# Unveiling the mitochondrial genome of *Salvia splendens* insights into the evolutionary traits within the genus *Salvia*

**DOI:** 10.1038/s41598-025-96637-9

**Published:** 2025-04-17

**Authors:** Heyu Yang, Yang Ni, Jingling Li, Haimei Chen, Chang Liu

**Affiliations:** Institute of Medicinal Plant Development, Chinese Academy of Medical Sciences and Peking Union Medical College, Beijing, 100193 People’s Republic of China

**Keywords:** *Salvia splendens*, Lamiales, Mitochondrial genome, Recombination, Intron markers, Comparative genomics, Evolutionary genetics

## Abstract

Previously, we resolved the complete sequences of the mitochondrial genomes (mitogenome) of two *Salvia* species (*S. miltiorrhiza* and *S. officinalis*). The major configurations of these two species were two circular chromosomes. In this study, we further studied the mitogenome of a floral species of *Salvia* (*Salvia splendens*) to understand the diversity and evolution of the *Salvia* mitogenomes. We sequenced the total DNAs of *S. splendens* using the Nanopore and Illumina platforms and assembled the mitogenome using a hybrid assembly strategy. The major configurations of the *S. splendens* were two circular chromosomes with lengths of 182,239 and 165,055 bp. There were 32 protein-coding genes (PCGs), three rRNA genes, and 18 tRNA genes annotated in the *S. splendens* mitogenome. We found 56 pairs of repetitive sequences in the *S. splendens* mitogenome. Three of them (R01, 04, and 07) could mediate recombination, whose products could be identified by the mapping of Nanopore reads, PCR amplifications, and Sanger sequencing of the PCR products. 457 RNA editing sites were identified in the *S. splendens* mitochondrial RNAs when comparing the RNA-seq data with their corresponding DNA templates. We showed that *S. splendens* was a sister taxon to *S. miltiorrhiza* based on the mitogenomes, consistent with the phylogeny determined with the plastome sequences. Crucially, we developed 12 mitochondrial markers sourced from mitochondrial intron regions to facilitate the identification of three *Salvia* species. Our study offers a comprehensive view of the structure of the *Salvia* mitogenomes and provides robust mitochondrial markers for *Salvia* species identification.

## Introduction

*Salvia splendens* Ker-Gawl, also called scarlet sage, is a perennial herbaceous plant from the Lamiaceae family. It was native to Brazil and was popular as a bedding plant all over the world. The phenolic metabolites and flavone triglycoside from the methanol extract of *S. splendens* leaves were shown to have significant hypoglycemic, anti-inflammatory effects, and in vitro antioxidant activity^1^. The extracts of *S. splendens* were found able to phytosynthesize AgNPs^2^. Moreover, the AgNPs derived from *S. splendens* had cytotoxicity against human lung cancer cell line A549^3^. Furthermore, the diterpenes found in *S. splendens* may be useful as neuropharmacological agents^4^.

Apart from these pharmacophylogenetic and synthetic studies on *S. splendens*, the genome resources of *S. splendens* have also been reported. Two versions of nuclear genome assemblies were published with genome sizes of 808 and 807 Mb, respectively^5,6^. The plastid genome (plastome) of *S. splendens* was also characterized by a length of 150,604 bp^7,8^. During the progress of this study, a mitochondrial genome sequence of *S. splendens* was released in the public database (GenBank: PNBA02000024.1). However, the genome is not even annotated, and we have not found any corresponding publication. As a result, the mitochondrial genome of *S. splendens* remains to be analyzed.

Mitochondria produce cellular ATP through oxidative phosphorylation in animal, fungi, and plant cells. The plant mitogenomes were unique compared to their counterparts of animals or fungi^9^. The size of plant mitogenomes ranged from 66 kb for *Viscum scurruloideum* to 11,000 kb for *Silene conica*, larger than those of animals or fungi^10,11^. Furthermore, many structural rearrangements were found in the plant mitogenomes resulting from intra- or inter-molecular homologous recombination mediated by repetitive sequences^12,13^. Specifically, the plant mitogenome was found to have complex gene expression regulation mechanisms demonstrated by cis- and trans-splicing, and RNA editing events^10,14^. While plant mitochondrial DNA (mtDNA) exhibits a slower nucleotide substitution rate^15^, mitochondrial markers have been developed for both phylogenetic analysis and species identification. These markers are derived from the less conserved regions of the mitochondrial genomes, including intron and intergenic sequences^16,17^.

By March 2023, the complete mitogenome sequences of 589 plant species have been deposited in the GenBank (https://www.ncbi.nlm.nih.gov/genome/browse/#!/organelles/). Structural differences and size variations were observed among the mitogenomes of these plant species as previously described^18,19^. Most genes were conserved except those lost in the mitogenomes and transferred to the nuclear genomes^20,21^.

Previously, we determined the complete mitogenome sequences of two *Salvia* species *S. miltiorrhiza*^22^ and *S. officinalis*^23^. To enhance our understanding of the structural differences, size variations, and divergence of gene contents and orders of *Salvia* mitogenomes, we here to study the mitogenome of *S. splendens*. We sequenced the *S. splendens* total DNAs using the Illumina and Nanopore sequencing platforms and assembled the *S. splendens* mitogenome using a hybrid strategy. The genome structure, gene contents, extent of RNA editing events, and repeat-mediated homologous recombination were compared among these three *Salvia* species, which shed light on the mechanisms driving the evolution of *Salvia* mitogenomes. Furthermore, we have identified 12 mitochondrial markers derived from intron regions of the mitochondrial genome. These polymorphic markers serve as reliable tools for distinguishing the three *Salvia* species.

## Materials and methods

### Plant materials and nucleic acid extraction

Young and fresh leaves of *S. splendens* were collected from a cultivated line (ss01) from Songjiang, Shanghai city (30^◦^56′49.5″N, 121^◦^15′23.3″E), China. *S. splendens* is neither an endangered nor protected species. The permission to collect *S. splendens* has been obtained through the Institute of Medicinal Plant Development, Chinese Academy of Medical Sciences and Peking Union Medical College. We deposited the voucher specimen under the accession number ss-vs01 at the Institute of Medicinal Plant Development, Chinese Academy of Medical Sciences, Peking Union Medical College, Beijing, China. We stored the leaves of *S. splendens* at −80 °C until use. The total DNA and RNA of 0.1 g leaves per sample were extracted using a plant genomic DNA kit (Tiangen Biotech, Beijing, Co., Ltd.) and an RNAprep Pure Plan Kit (Tiangen Biotech Beijing CO., LTD), respectively. We assessed the purity of DNA and RNA with electrophoresis using the 1.5% agarose gel and quantified the DNA and RNA concentration using the Nanodrop spectrophotometer 2000 (Thermo Fisher Scientific, America).

### DNA sequencing and mitogenome assembly

We generated the DNA sequencing data by Oxford Nanopore and Illumina sequencing technologies. In Nanopore sequencing, the DNA yield of ss01 was evaluated using a Qubit instrument, and DNA quality was evaluated via a Nanodrop instrument. Further DNA fragmentation and selection of DNA fragments with the size of 10–50 kb were conducted using the Blue-pippin system (Sage Science, MA, USA). For sequencing library preparation, we used the 1D ligation kit (SQK-LSK109) and then performed the sequencing on a PromethION Flow Cell (Oxford Nanopore Technologies, UK). Base calling was conducted using Guppy v3.2.2 18 with default parameters. The high-quality reads (Q-score ≥ 7) were obtained for the mitogenome assembly.

For Illumina sequencing, the sequencing libraries were prepared using a Truseq Nano DNA HT Sample Preparation Kit (Illumina) following the manufacturer’s instructions. Briefly, the DNA sample of the same line (ss-01) was fragmented to an average length of 350 bp by sonication, end polished, and ligated with the adapter for PCR amplification. Further PCR amplicons were purified using the AMPure XP system (Beckman Coulter, CA, USA) and the size distribution of PCR amplicons was analyzed using an Agilent 2100 Bioanalyzer (Agilent Technologies, Palo Alto, CA, USA). Finally, libraries were sequenced on an Illumina NovaSeq 6000 platform and 150 bp paired-end reads were obtained. The above steps of sequencing were conducted at Grandomics Biotechnology Co., Ltd (Wuhan, China).

For RNA sequencing, the rRNAs in the total RNA were depleted via a Ribo-Zero™ Magnetic Kit (Epicenter, Madison, WI, USA). The rRNA-depleted sequencing library was constructed using a VAHTS Universal V8 RNA-seq Library Prep Kit (Vazyme, Nanjing, China) following the manufacturer’s recommendations. The library sequencing was performed on an Illumina HiSeq 2500 sequencer (2 × 150 bp reads) at Grandomics Biotechnology Co., Ltd.

We assembled the mitogenome of *S. splendens* using a hybrid assembly strategy. We first extended and achieved the mitochondrial reads from Illumina sequencing data using GetOrganelle (v1.6.4)^24^. The extended reads were assembled into a unitig graph using the SPAdes packaged in the Unicycler (v0.4.9)^25^. Then the double-bifurcation structures (DBSs) in the unitig graph were resolved by the long Nanopore reads using the Unicycler software.

To validate the assembly results of the unicycler, we obtained the DBS sequences and their flanking sequences of 1000 bp long. These two sequences were named configurations 1 and 2 (c1 and c2 in short). Their flanking sequences were then switched to mimic the homologous recombination products. The resulting two sequences were named configurations 3 and 4 (c3 and c4 in short). Then the nanopore long reads were mapped to these sequences using BWA (v0.7.12-r1039)^26^ with default parameters. The number of reads mapped to each configuration was counted. Among the four configurations, the two configurations supported with more Nanopore long reads were selected in the final assembly of the *S. splendens* mitogenome.

Then we mapped the Nanopore and Illumina sequencing reads to the final assembly of the *S. splendens* mitogenome using BWA with default parameters. The coverage of every site in the mitogenome and the median coverage were calculated using the samtools (v1.3.1)^27^.

The collinearity analysis of mitogenomes assembled in this study and the one released in GenBank (PNBA02000024.1) were performed using the nucmer module in the Mummer package (v3)^28,29^. The sequences were aligned using the many-to-many model with an identity threshold of 85%. Then we visualized the collinearity results using the Rideogram package of R^30^. The variable sites between the two assemblies were identified using BLASTN (v 2.10.1 +)^31^ and the positions of variable sites were detected using the blastn2snp tool from JVarkit (v.1.0)^32^. Moreover, the collinearity of *S. splendens* mitogenome and those of *S. miltiorrhiza*^22^ and *S. officinalis*^23^ were performed using the method described above.

### Mitogenome annotation

We annotated the final assembly of the *S. splendens* mitogenome using the MGA pipeline (http://www.1kmpg.cn/mga). The tRNA genes were found via tRNAscan-SE^33^. The positions of the start and stop codons and intron/exon boundaries of each gene were manually checked using the Apollo program^34^. We drew the circular map of the final assembly using PMGView (http://www.1kmpg.cn/pmgview). The sequences of the *S. splendens* mitogenome were deposited in the GenBank under the accession numbers OQ675154 and OQ675155.

### Analysis of the repeat-mediated recombination in the mitogenome

To explore possible recombination events mediated by the repetitive sequences in the *S. splendens* mitogenome, we identified the repetitive sequences using ROUSfinder v2^35^. We then extracted the 1000 bp-long flanking sequences of each repetitive sequence and constructed the sequences corresponding to the four configurations c1, c2, c3, and c4. The Nanopore sequencing data were mapped to these four configurations and the numbers of repeat-spanning reads for each configuration were calculated. The Integrative Genomics Viewer (IGV) software (v 2.15.1)^36^ was used to visualize the mapping results.

We extracted the repetitive sequences and their 200 to 700 bp-long flanking sequences as template sequences. The primers were designed to amplify the s template sequences using the Primer-BLAST. PCR amplification was conducted in a reaction of 50 µl, including 23 µL water, 25 µL 2 × Taq PCR Master Mix, 1 µL of each primer, and 1 µL total DNA. We performed the PCR amplification on a Pro-Flex PCR system (Applied Biosystems, Waltham, MA, USA) under the following conditions: denaturation at 94 °C for 2 min, followed by 35 cycles of 94 °C for 30 s, 57 °C for 30 s, 72 °C for 60 s, and 72 °C for 2 min of the final extension. The long fragments of four configurations associated with R01 were amplified using KOD One™ PCR Master Mix (TOYOBO, Japan). All of the PCR amplicons were analyzed using electrophoresis on a 1.5% agarose gel. Amplicons of R04 and R07 of the expected size were sequenced using the Sanger sequencing methods.

### Analysis of repetitive sequences

We used the MISA web service^37^ to identify the SSRs in the *S. splendens* mitogenome with the thresholds of 10, 5, and 4 for the number of mono-, di-, trinucleotide repeat units, respectively, and 3 for the numbers of tetra-, penta-, and hexanucleotide repeat units. We detected the tandem repeats in the *S. splendens* mitogenome using the Tandem Repeats Finder (v 4.09) [70]. The parameters of the Tandem Repeats Finder were set as follows: 2 for matches, 7 for mismatches and indels, and 50 and 500 for the minimum alignment score and maximum period size, respectively.

### Identification of mitochondrial plastid sequences (MTPTs)

We assembled the plastome of the *S. splendens* using GetOrganelle with the parameters: “-R 15 -k 21,45,65,85,105 -F embplant_pt”. The plastome sequence obtained in this study was similar to the plastome sequence (OM617847.1) reported in our previous study^7^. We determined the MTPTs of *S. splendens* by searching the homologous sequences between the plastome and the mitogenome using BLASTn (v 2.10.1 +) with the parameters of e-value < 1*e*-6 and word size of 7^22^. Hits less than 100 bp long and having less than 80% sequence identity to the query sequences were excluded. Then, the sequences consisting of MTPTs and their 2000 bp long flanking regions were extracted. The Nanopore long reads were mapped to these sequences and the mapping results were visualized using the Integrative Genomics Viewer (IGV) software (v 2.15.1)^36^. All sequences of MTPTs were annotated using CPGAVAS2 (http://www.1kmpg.cn/cpgavas2) to identify the genes located in the MTPTs.

### Phylogenetic analysis of the twelve Lamiales species based on common mitochondrial genes

To resolve the phylogenetic relationship of the Lamiales species, we obtained the whole mitogenome sequences of twelve Lamiales species and two outgroup species. The twelve Lamiales included *Ajuga reptans* (NC_023103.1), *Rotheca serrata* (NC_049064.1), *Scutellaria tsinyunensis* (MW553042.1), *S. miltiorrhiza* (MN_585275/6.1), *S. officinalis* (OQ001564 and OQ001565), *S. splendens* (OQ675154 and OQ675155), *Erythranthe lutea* (NC_018041.1), *Castilleja paramensis* (NC_031806.1), *Utricularia reniformis* (NC_034982.1), *Dorcoceras hygrometricum* (NC_016741.1), *Osmanthus fragrans* (NC_060346.1), and *Hesperelaea palmeri* (NC_031323.1). The two outgroup species were *Nicotiana tabacum* (NC_006581.1) and *Solanum lycopersicum* (NC_035963.1).

Then we extracted the CDS of 26 PCGs (*atp*1, *atp*4, *atp*6, *atp*8, *atp*9, *ccm*B, *ccm*C, *ccm*Fc, *ccm*Fn, *cob*, *cox*1, *cox*2, *cox*3, *mat*R, *mtt*B, *nad*1, *nad*2, *nad*3, *nad*4, *nad*4L, *nad*5, *nad*6, *nad*7, *nad*9, *rps*12, and *rps*13) shared among all fourteen species via PhyloSuite (v1.2.1)^38^. We aligned these CDS with MAFFT (v7.450)^39^ and concatenated the aligned results into a data matrix using PhyloSuite. We established the ML tree using the data matrix and RAxML (v8.2.4)^40^ with the parameters “raxmlHPC-PTHREADS-SSE3 -f a -N 1000 -m PROTGAMMACPREV—× 551314260 -p 551314260 -o Nicotiana_tabacum, Solanum_lycopersicum -T 20.” We obtained the bootstrap support values of each branch in the ML tree after 1000 replicates. We also built the BI tree using MrBayes (v3.2.7)^41^ with the model TVM + I + G and the parameters calculated by jMdoleTest (v2.1.0)^42^. We visualized the two trees using the iTOL web server (https://itol.embl.de).

Moreover, we extracted the CDS of 56 PCGs (*atpI, atpF, rps15, atpE, rpl22, rpl16, psbK, psbF, petD, rps3, petA, psaC, rpl14, clpP, rbcL, rps2, rps16, psbH, petL, atpA, rpoB, psbJ, petN, rpl20, rps11, ycf4, accD, rpl2, psbA, psbM, rps4, psbD, rpoC2, petG, matK, rpoA, petB, ycf1, rpl23, psbL, rps8, ycf3, psbC, psbN, rps18, ycf2, rps14, rpl33, atpH, psbE, rpl36, psaB, psbT, psaA, rps7,* and *rpoC1*) shared among the chloroplast genomes of these fourteen species. The corresponding accession numbers are *Ajuga reptans* (NC_023102.1), *Rotheca serrata* (MN814867), *Scutellaria tsinyunensis* (NC_050161.1), *S. miltiorrhiza* (NC_023431.1), *S. officinalis* (NC_038165.1), *S. splendens* (NC_050901.1), *Erythranthe lutea* (NC_030212.1), *Castilleja paramensis* (NC_031805.1), *Utricularia reniformis* (NC_029719.1), *Dorcoceras hygrometricum* (NC_016468.1), *Osmanthus fragrans* (NC_042377.1), *Hesperelaea palmeri* (NC_025787.1), *Nicotiana tabacum* (NC_001879.1) and *Solanum lycopersicum* (NC_007898.1). The ML and BI trees were constructed and visualized using the same methods described above.

### Detection of RNA editing sites

To determine potential RNA editing sites in the *S. splendens* mitogenome, we extracted the sequences for each CDS and their 100 bp long flanking sequences as the reference sequences. We mapped the strand-specific RNA-seq reads to these reference sequences using HISAT2 (v 2.2.1)^43^. The parameters of the HISAT2 were “-rna-strandness RF -sensitive -no-mixed -no-discordant” as described previously^44^. The RNA editing sites were identified using the REDItools (v 2.0)^45^ with the parameters of coverage ≥ 5 and frequency ≥ 0.1^46^. We further checked and visualized the mapping results using the IGV software (v 2.15.1)^36^ with a minor variant frequency ≥ 0.1.

We identified the SNP sites in the CDS of *S. splendens* mitogenome using the same method described previously^23^. Briefly, we mapped the Illumina sequencing data to the template sequences described above using BWA with default parameters. The SNP sites were determined using REDItools (v 2.0) with parameters of coverage ≥ 5 and frequency ≥ 0.1.

### Development of genetic markers for interspecific identification based on mitochondrial introns

To develop the mitochondrial intron markers, we extracted the intron sequences of the mitochondrial genomes of *S. splendens* obtained in this study and *S. miltiorrhiza*^22^, *S. officinalis*^23^ using a custom Python script. Alignments of intron sequences were carried out using ClustalW2 and interspecific polymorphic sites of each intron were identified using custom Python script. Subsequently, we extracted the polymorphic sites and their 300 bp long flanking regions, employing them as target regions for primer design. Detailed methods of primer design, PCR amplification, and Sanger sequencing are elucidated above. We conducted the PCR amplification using the genomic DNA samples of three different individuals as templates for each species.

## Results

### Analysis of the *S. splendens* mitogenome structure

**Fig. 1:**
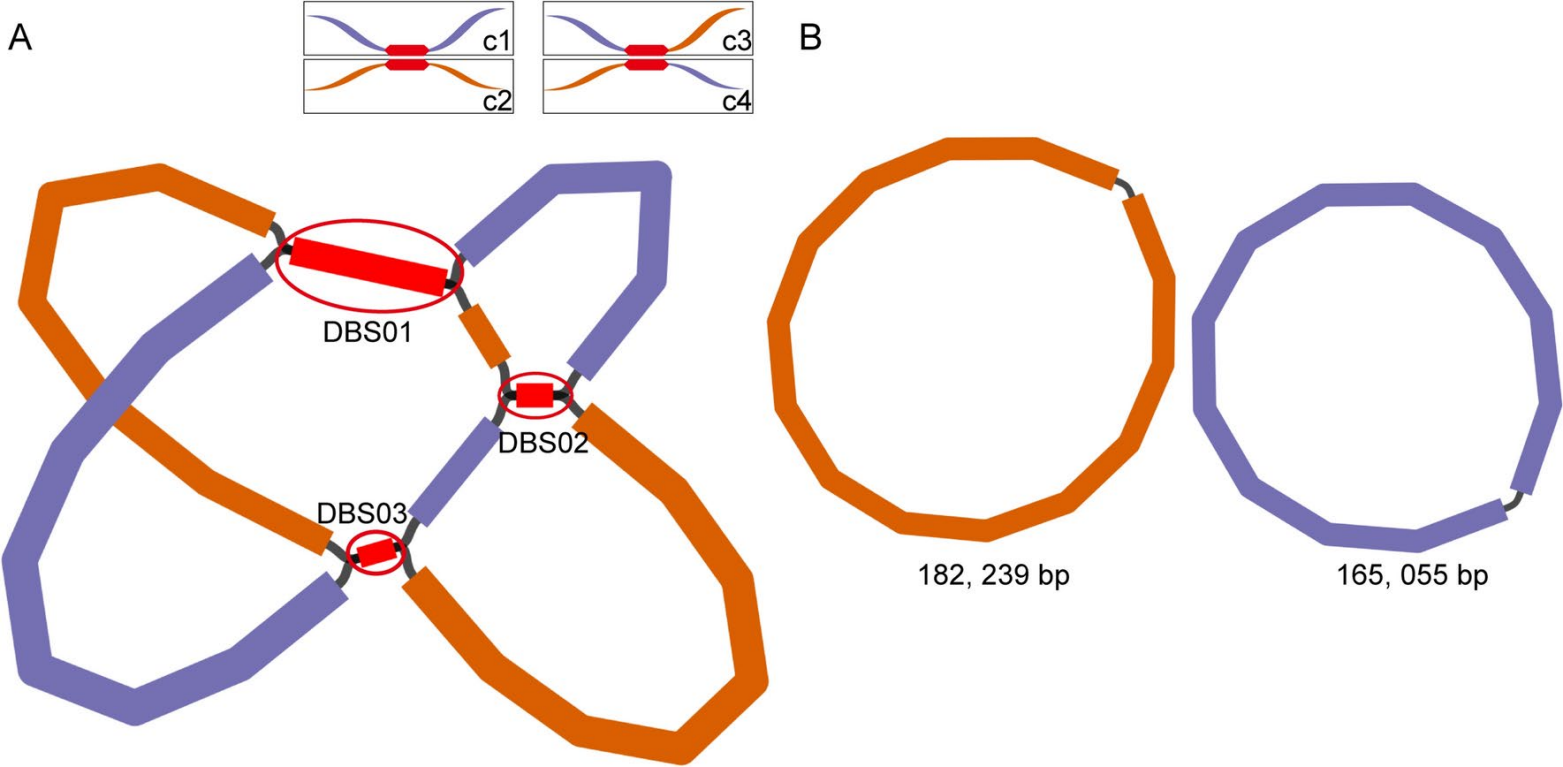
Schematic representation of the assembly steps of *S. splendens* mitogenome. (**A**) Unitig graph of the *S. splendens* mitogenome was obtained from the assembly of Illumina reads using Unicycler. The unitig graph contained nine contigs that formed three DBSs (DBS01–03, red circle). Each DBS has four configurations (c1, c2, c3, and c4), which were named and illustrated using the same method as the one in our previous report^22^. DBS01 was shown in the top left corner as an example. (**B**) Schematic graph of MC1 (orange circle) and MC2 (blue circle) of *S. splendens* after the DBSs were resolved by long reads.

We sequenced the S. *splendens* samples using the Nanopore and Illumina sequencing platforms. The statistical results of the sequencing data are shown in (Table S1). The Nanopore and Illumina reads were deposited in GenBank under the accession numbers: SRR23936021 and SRR23936021, respectively. The resulting sequences were assembled using a hybrid assembly strategy. The assembly steps are depicted in (Fig. 1A). Firstly, we assembled the Illumina reads using Spades and obtained a unitig graph. Three double-bifurcation structures (DBSs) were identified. Each DBS structure has four configurations, named c1–4. Here c1 and c2 represented the major configurations. And c3 and c4 represent the configurations resulting from c1 and c2’s recombinations. We then resolved the DBS structures using Unicycler, resulting in two circular chromosomes (Fig. 1B).

To confirm that the two circular chromosomes were assembled correctly, we mapped the long reads back to the core sequences of the DBSs and their 1000 bp long flanking sequences (Fig. S1). The mapping results were consistent with the two circular chromosomes as that resolved by Unicycler (Fig. S1). The location of these DBS structures on MC1 and MC2 is shown in (Table 1). The two circular chromosomes were named MC1 and MC2. MC stands for mitochondrial chromosome. MC1 and MC2 were 182, 239 and 165, 055 bp long with a GC content of 44.84 and 44.43%, respectively.

**Table 1:** Mapping results of Nanopore long reads to the four possible configurations associated with three DBS (DBS01–03) of the *S. splendens* mitogenome, which correspond to repetitive sequences (R01, R04, and R07).

Repetitive sequences ID	DBS ID	Query sequence	Subject sequence	Alignment length	Repeat copy 1			Repeat copy 2			Numbers of long reads mapped to each DBS				Recombination frequency (%)
					Start	End	Strand	Start	End	Strand	c1	c2	c3	c4	
R01	DBS01	MC1	MC2	13331	67353	80683	plus	91656	104986	plus	17	15	16	6	40.74
R04	DBS02	MC1	MC2	192	86255	86446	plus	155991	155800	minus	48	42	1	3	4.26
R07	DBS03	MC1	MC2	123	170622	170744	plus	1118	1239	plus	90	76	1	0	0.60

The percentage of minor DBS configurations was calculated as the number of reads mapped to the configurations having fewer mapped reads divided by those mapped to all configurations.

*DBS* double-bifurcation structure, *MC1/2* mitogenome chromosome 1/2.

^a^Validated successfully with PCR.

^b^all positions are based on MC1, except those marked with MC2.

We then mapped the short reads and long reads to the sequences of MC1 and MC2, simultaneously. We detected the median coverage to be 73 and 80 for MC1 and MC2, respectively (Fig. S2A,B). The short reads were mapped back to the MC1 and MC2 with the median coverage being 191 and 212 for MC1 and MC2, respectively (Fig. S2C,D).

**Fig. 2:**
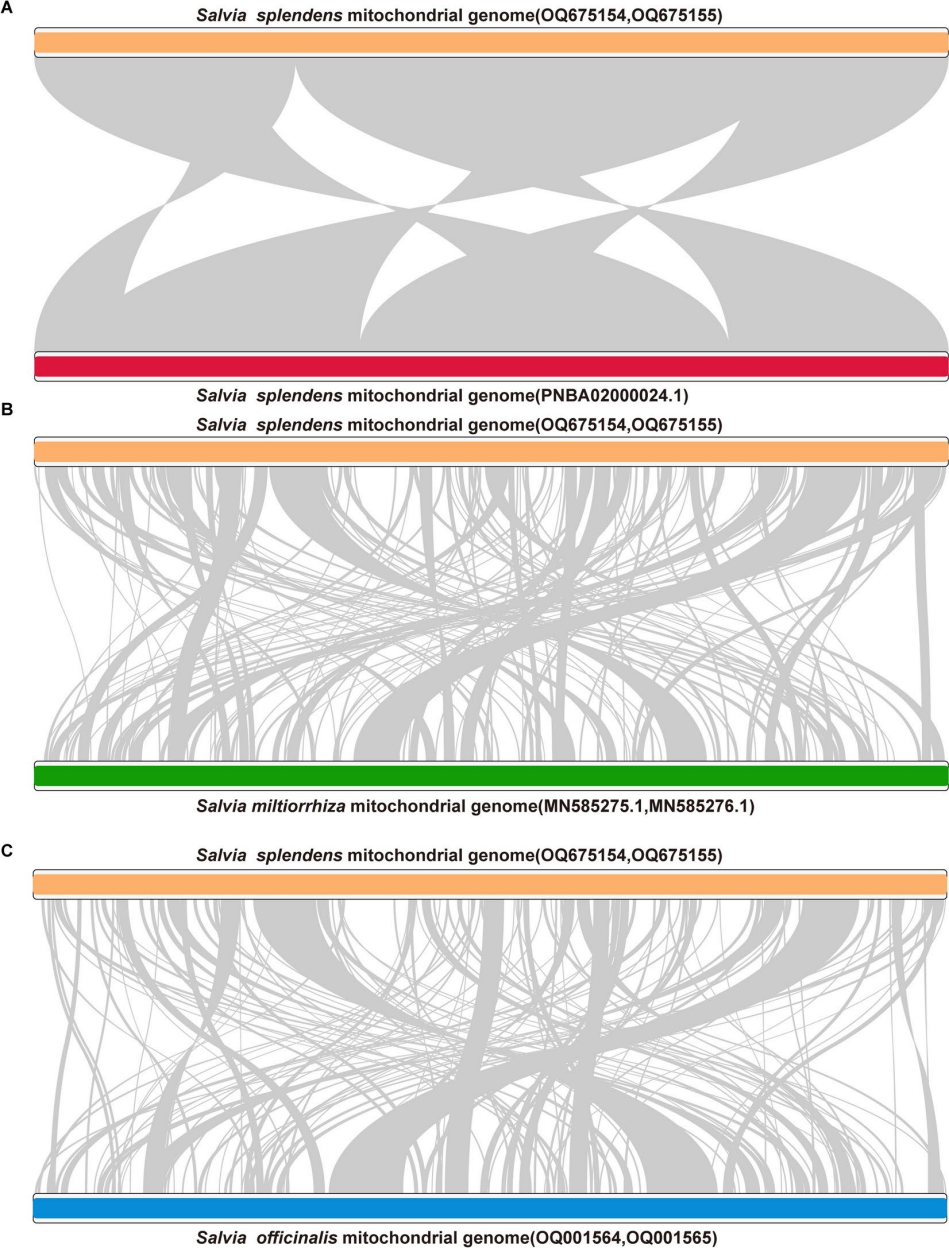
Syntenic analysis of the mitogenome sequences of *S. splendens* (OQ675154 and OQ675155) in this study and *S. splendens* (PNBA02000024.1) (**A**), *S. splendens* (OQ675154 and OQ675155) and *S. miltiorrhiza* (**B**), and *S. splendens*(OQ675154 and OQ675155) and *S. officinalis* (**C**). The mitogenomes of *S. splendens* (OQ675154 and OQ675155), *S. splendens* (PNBA02000024.1), *S. miltiorrhiza,* and *S. officinalis* are shown with orange, red, green, and blue bars, respectively. Regions that have an identity score of more than 85% are connected with grey arcs.

To determine the intra-specific and inter-specific variations of *Salvia* mitogenomes, we compared the mitogenome sequences of *S. splendens* in this study with those of *S. splendens* deposited in Genbank and *S. officinalis* and *S. miltiorrhiza*^22^ using Mummer (v3)^28^ with the identity threshold of 85% and the many-to-many model*.* For comparison of the sequences of *S. splendens* mitogenome assembled in this study and the one released in GenBank (PNBA02000024.1), the alignable regions covered all sequences of the two whole mitogenomes under the identity threshold (Fig. 2A). In total, there were five alignable regions between the two sequences with a length of 31,723 to 104,986 bp and identity of 99.81 to 99.99% (Table S2). We then detected 166 variable sites between these two assemblies using BLASTN (v 2.10.1 +) and Jvarkit (v.1.0) (Table S3).

We compared the sequences of *S. splendens* and *S. miltiorrhiza* mitogenomes, the lengths of the alignable regions were 205,371 bp (59.13% of the complete mitogenome sequences) and 204,958 bp (49.49% of the complete mitogenome sequences) of *S. splendens* and *S. miltiorrhiza* mitogenome*,* respectively (Fig. 2B). We then compared the sequences of *S. splendens* and *S. officinalis* mitogenomes, the length of the alignable regions was 169,505 bp (48.81% of the complete mitogenome sequences) and 169,816 bp (55.11% of the complete mitogenome sequences) of *S. splendens* and *S. officinalis* mitogenomes*,* respectively (Fig. 2C).

**Fig. 3:**
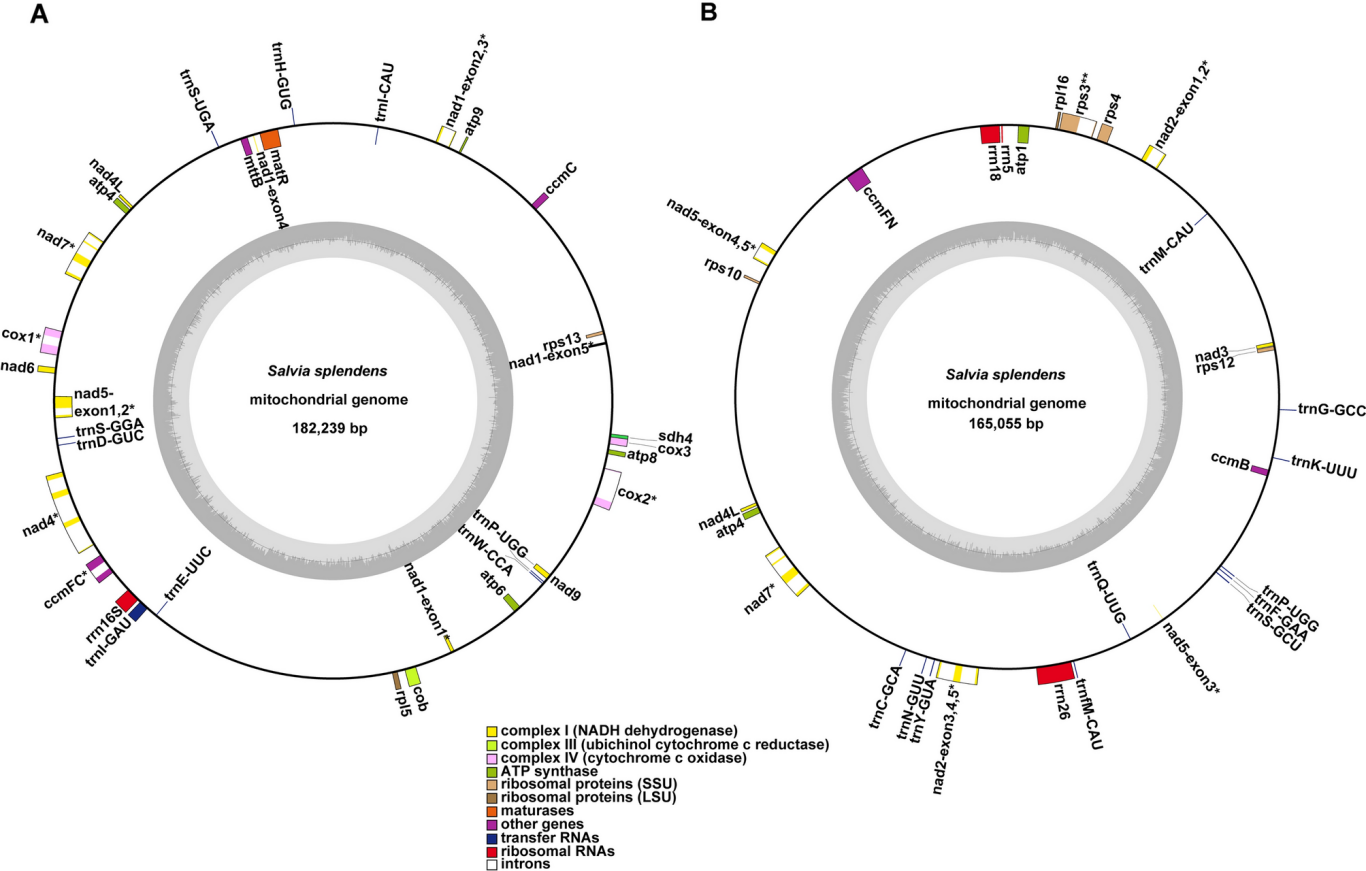
Schematic representation of the circular chromosomes MC1 (**A**) and MC2 (**B**) of *S. splendens* mitogenome*.* The graph was drawn using PMGView (http://www.1kmpg.cn/pmgview). Genes shown on the inside were on the negative strand, whereas those on the outside were on the positive strand. Genes with introns were highlighted using “*”. The gray circle represents the GC contents. The circle inside the GC content graph marks the 50% threshold. The colors indicate different functional categories shown in the legend.

**Fig. 4:**
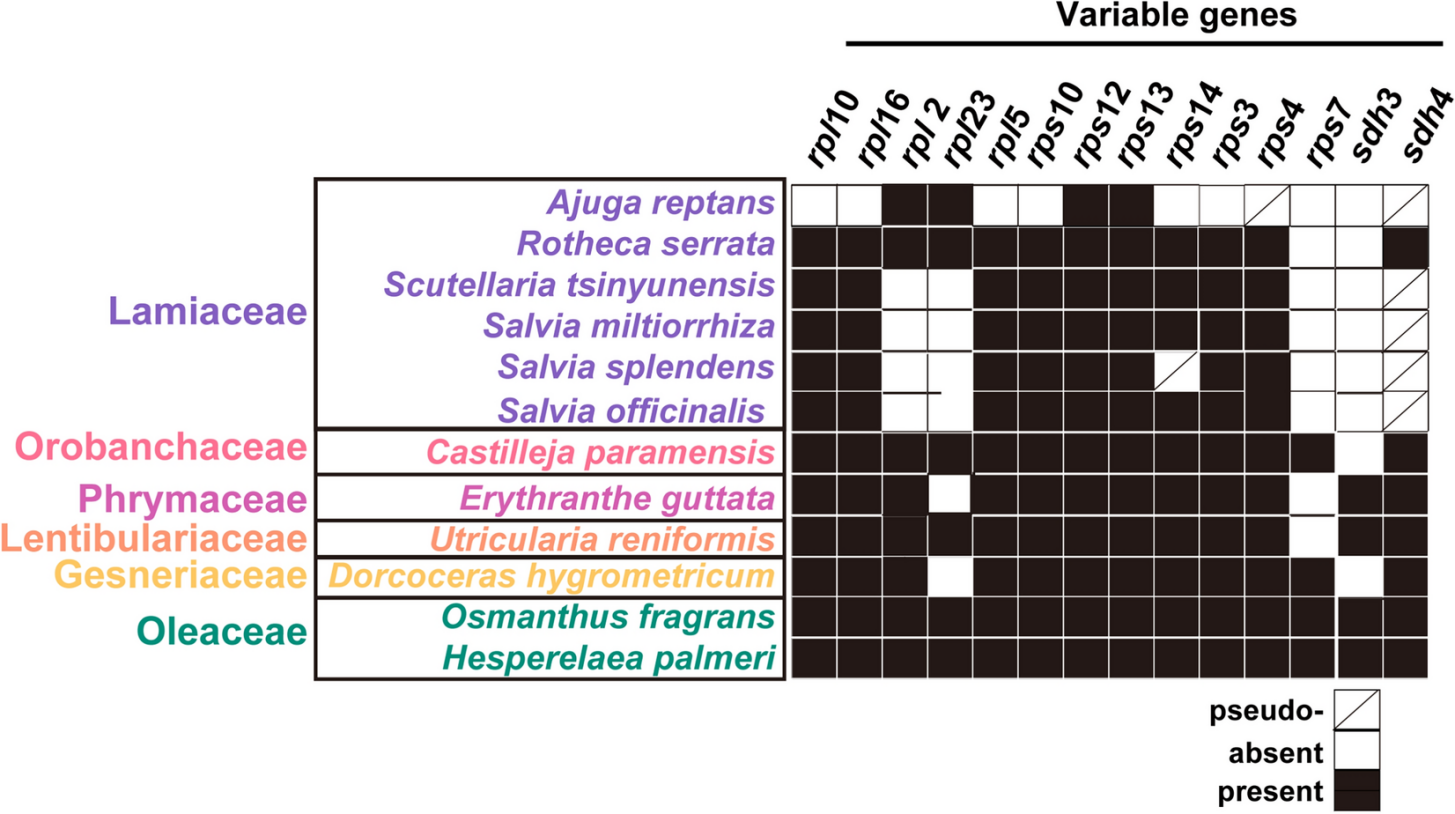
Variable gene contents in twelve sequenced Lamiales mitogenomes. The X-axis shows the names of core and variable genes.

We annotated 51 genes in the *S. splendens* mitogenome, including 32 PCGs, three rRNA genes, and 18 tRNA genes. Among the 32 PCGs, 24 PCGs were identified in most angiosperm plants and were classified as core genes in the previous report^47^. And 8 PCGs were found in some of the angiosperm plants and were classified as variable genes (Figs. 3, Fig. 4,Table 2). As shown in Fig. 4, the variable genes *rps7* and *sdh3* were both missing in the six mitogenomes of Lamiaceae. The total length of the coding sequences (CDS) of genes was 49,955 bp, accounting for 14.38% of the total length of the *S. splendens* mitogenome.

**Table 2:** Genes predicted in the mitogenome of *S. splendens.*

Group of genes		Name of genes
Core genes	ATP synthase	*atp*1*, atp*4, *atp*6*, atp*8*, atp*9
	Cytochrome c biogenesis	*ccm*B*, ccm*C*, ccm*FC^a^, *ccm*FN
	Ubichinol cytochrome c reductase	*cob*
	Cytochrome c oxidase	*cox*1^a^, *cox*2^a^, *cox*3
	Maturases	*mat*R
	Transport membrane protein	*mtt*B
	NADH dehydrogenase	*nad*1^c^, *nad*2^c^, *nad*3, *nad*4^b^, *nad*4L*, nad*5^c^*, nad*6, *nad*7^b^, *nad*9
Variable genes	Ribosomal protein large subunit	*rpl*5*, rpl*10*, rpl*16
	Ribosomal protein small subunit	*rps*3^a^*, rps*4*, rps*10^a^*, rps*12*, rps*13
rRNA genes	Ribosomal RNAs	*rrn*5*, rrn*18*, rrn*26
tRNA genes	Transfer RNA	*trn*C-GCA, *trn*M-CAU, *trn*E-UUC, *trn*H-GUG, *trn*I-CAU, *trn*P-UGG, *trn*S-UGA, *trn*I-CAU, *trn*P-CGG, *trn*C-GCA, *trn*F-GAA, *trn*G-GCC, *trn*K-UUU, *trn*fM-CAU, *trn*Q-UUG, *trn*S-GCU, *trn*Y-GUA, *trn*W-CCA

“^a^”, “^b^”, and “^c^”: genes with two, four, and five exons, respectively.

### Repetitive sequences in the *S. splendens* mitogenome and their involvement in recombination

We found 56 pairs of repetitive sequences in the *S. splendens* mitogenome using the ROUSFinder2.0.py and checked the recombination frequency of these repetitive sequences using the Nanopore long reads (Table 1, Table S4). Among them, we found the recombination products associated with three repetitive sequences (R01, 04, and 07) based on the mapping results of the long Nanopore reads (Table 1). The length of the longest repetitive sequence (R01) of *S. splendens* mitogenome was 13,331 bp and it had the highest recombination frequency (40.74%). The other two repetitive sequences (R04 and R07) had lower recombination frequencies of 4.26% and 0.60%, respectively.

**Fig. 5:**
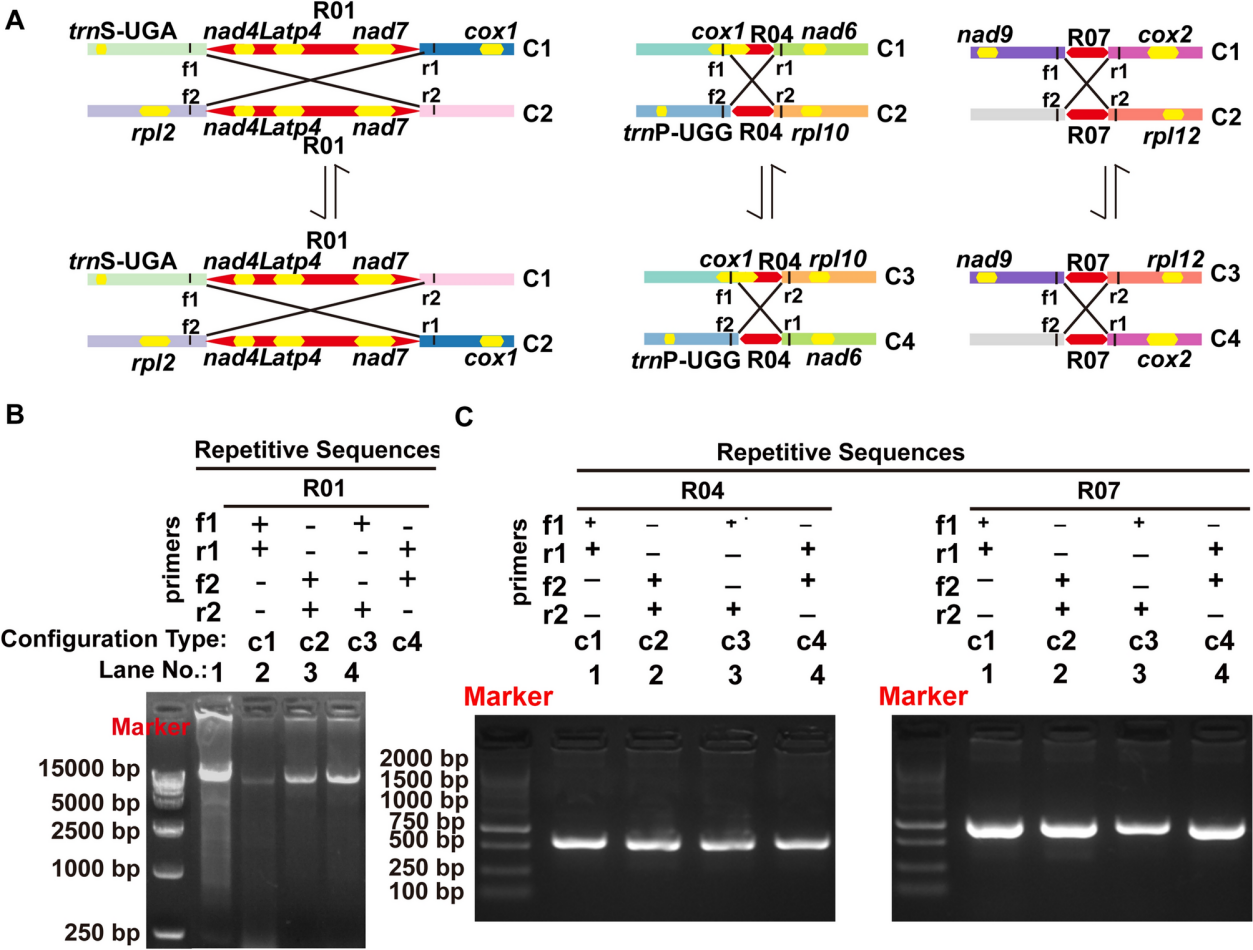
PCR verification of recombination products associated with the repetitive sequences (R01, R04, and R07) on MC1 and MC2. (**A**) Schematic representation of the four configurations (c1–c4) associated with each repetitive sequence. The regions corresponding to the primers are shown as red blocks. f1 and f2: forward primers. r1 and r2: reverse primers. (**B**) Electrophoretic gel plot of PCR products amplified with various combinations of forward and reverse primers to amplify the DNA molecules corresponding to configurations c1–c4. The name of the repetitive sequence, combinations of forward and reverse primers, expected configuration to be amplified, and the lane numbers are shown above the gel plot.

We used PCR amplification and Sanger sequencing to validate the presence of the four configurations associated with the repetitive sequences of R04 and R07. The presence of the four configurations associated with R01 was validated using PCR amplification. The primer pairs were designed to amplify sequences corresponding to the four configurations (c1–c4). The primer sequences are shown in (Table S5). The PCR amplicons had the expected sizes (Fig. 5). And the Sanger sequencing results of PCR amplicons associated with R04 and R07 were similar to those of the templates (Fig. S3).

**Fig. 6:**
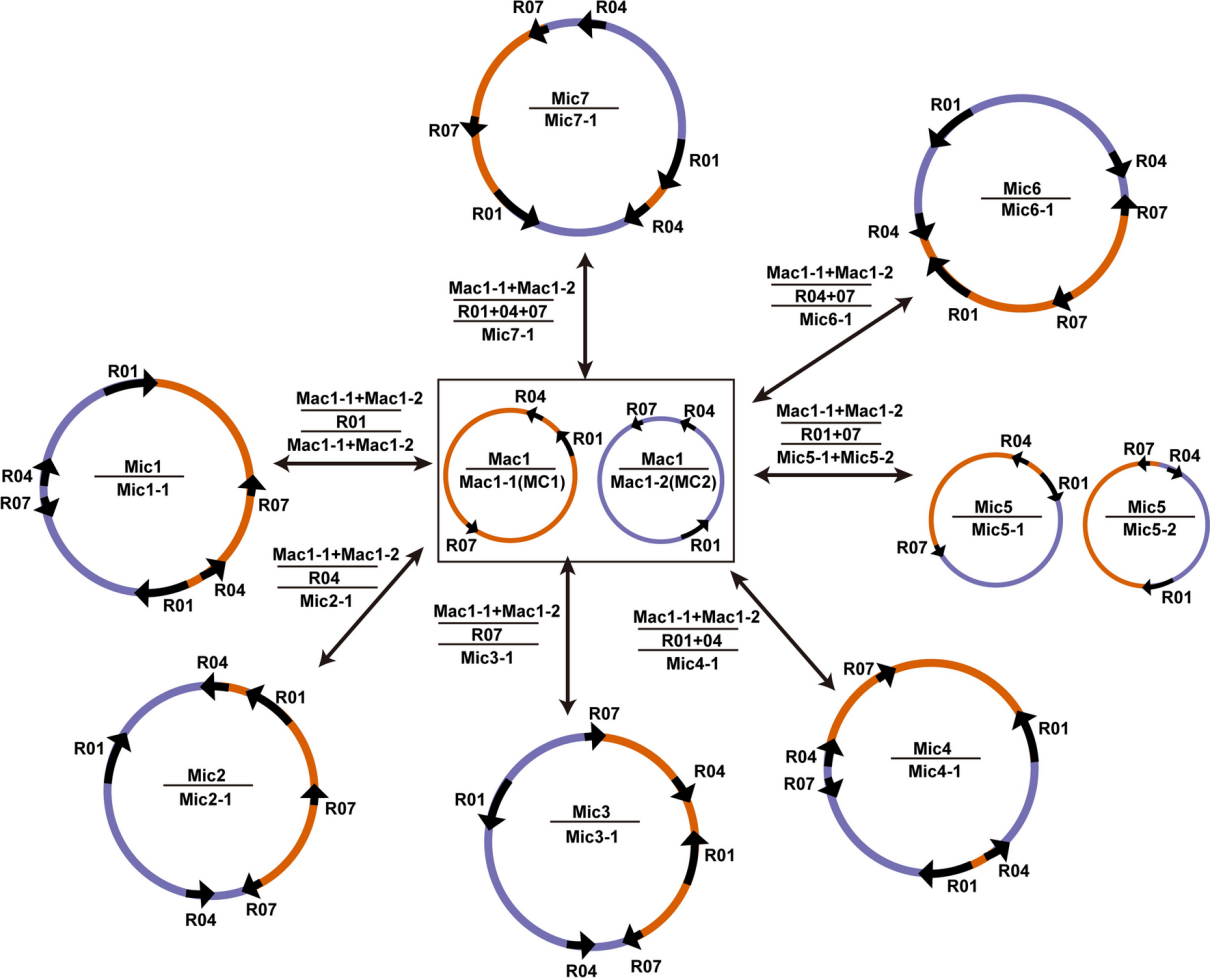
Products of homologous recombination mediated by one, two, and three of the repetitive sequences R01, R04, and R07. The repeat units of R01, R04, and R07 are represented by black arrows. Sequences around the repeat units are shown in different colors. The circles represent circular chromosomes. The genomic configuration is named “c” followed by the configuration number. In contrast, the circular chromosomes of a particular genomic configuration are named “c” followed by the configuration number, “-”, and the chromosome number. The double-headed arrows indicated the source circular chromosomes, the repetitive elements, and the product circular chromosomes, separated with horizontal lines. The genomic configuration name is prefixed with “Mac”, representing “major configuration” if it is the most abundant configuration. Otherwise, the genomic configuration name is prefixed with “Mic”, representing “minor configuration”. Each configuration can have a set of chromosomes. Mac1 is the genomic configuration containing chromosomes MC1 (Mac1-1) and MC2 (Mac1-2). Mac1-1 and Mac1-2 can undergo recombination mediated by R01, R04, R07 to form circular chromosomes Mic1-1, Mic2-1, and Mic3-1, respectively. Mac1-1 and Mac1-2 can undergo recombination mediated by repetitive sequences R01 and R04 to form a circular chromosome Mic4-1. Mac1-1 and Mac1-2 can undergo recombination mediated by repetitive sequences R01 and R07 to form two circular chromosomes: Mic5-1 and Mic5-2. Mac1-1 and Mac1-2 can undergo recombination mediated by repetitive sequences R04 and R07 to form two circular chromosomes: Mic6-1. Lastly, Mac1-1 and Mac1-2 can form Mic7 through recombinations mediated by R01, R04, and R07 together. Please note that we consider the Mac1 as the baseline configuration. Only newly formed circular chromosomes are shown for each newly formed genome configuration. By definition, it should also contain the circular chromosome in the source configuration that does not undergo recombination.

Using the major configuration as the baseline, we can infer possible minor configurations resulting from the recombination mediated by the three repetitive sequences. As shown in Fig. 6, the major configuration (Mac1) is placed in the middle. Seven configurations (Mic1-7) resulting from repeat-mediated recombination from Mac1 are shown around Mac1. Genome configurations Mac1 and Mic5 have two chromosomes. Each of the other six genome configurations (Mic1,2,3,4,6,7) has one chromosome only.

We also detected the tandem repeats and simple sequence repeats (SSRs) that can be used for species authentication, genetic variation, and evolution studies^48–52^ in the *S. splendens* mitogenome. Using the MISA web service and Linux version of the Tandem Repeats Finder (v4.09), we identified 89 SSRs and ten long tandem repeats (Tables S6–10). We identified 51 and 38 SSRs on the MC1 and MC2, respectively. The most abundant type of SSR was tetranucleotide repeats, accounting for 43.83% of all SSRs in the *S. splendens* mitogenome (Table S7). Ten long tandem repeats 8 and 2 tandem repetitive sequences were identified on the MC1 and MC2, respectively (Table S8)**.** Their repeat units ranged from 11–26 nt in size (Table S8).

### Identification of mitochondrial plastid sequences (MTPTs)

The mitochondrial plastid DNAs (MTPTs) are plastid DNAs (ptDNA) migrated into the mitochondrial DNA (mtDNA)^53,54^. We detected MTPTs in the *S. splendens* by comparing the complete mtDNA and ptDNA sequences of *S. splendens* using BLASTn with the parameters of e-value < 1*e*-6 and the word size of 7^22^. We found 28,175 bp of plastid-derived DNAs distributed among 15 different loci in the *S. splendens* mtDNAs (Table S9). All these 15 MTPTs occupied 8.11 and 18.71% of the complete ptDNA and mtDNA sequences of *S. splendens.* The length of these 15 different loci ranged from 103 (MTPT11) to 13,674 bp (MTPT12) (Table S9). The largest MTPTs (MTPT12) inserted the regions of MC2 from positions 77,995 to 91,668 (Table S9).

Most MTPTs represented noncoding DNA or fragments of the CDS, such as *rpo*B and *atp*B fragments in the MTPT02 and MTPT15 (Table S9). However, the MTPTs contained functional ptDNA-encoded genes such as five transfer RNA genes (*trnI*-GAU, *trn*D-GUC, *trn*S-GGA, *trn*V-GAC, and *trn*W-CCA) and the intact CDSs of 11 genes (*psb*B, *psb*H, *psb*N, *psb*T, *rpl*14, *rpl*2, *rpl*22, *rpo*A, *rps*11, *rps*19, *rps*8) (Table S9). We then mapped the long reads to the sequences containing the MTPTs and their 1000 bp of 5′ and 3′ flanking regions to confirm their presence. As a result, all 15 MTPTs including the longest MTPT (MTPT12) were supported by the mapping results of long reads (Fig. S4A–O).

### Identification of RNA editing sites

In angiosperms, RNA editing substitutes cytidines (C) for uridines (U) post-transcriptionally^55,56^, resulting in a change of the amino acid sequences^55^. These changes were likely to increase the overall diversity of the mitogenomic proteomes^57^, reshape the proteins’ physicochemical characteristics^58^, and modify proteins’ folding patterns^59^. Therefore, RNA editing plays an important role in the biological processes of plant mitochondria.

We identified the RNA editing events in the *S. splendens* mitochondrial RNAs based on the results of mapping the RNA-seq data to the reference genome. In total, 457 RNA sites were detected in the *S. splendens* mitochondrial RNAs. The detailed information for RNA editing sites is shown in (Table S10). The mapping results of the RNA-seq reads to the reference genome are shown in (Fig. S5A–Z). Among them, 46 (10.07%) and 411 (75.14%) RNA editing events resulted in synonymous and non-synonymous codon changes, respectively (Table S10). The non-synonymous codon changes mainly contained three amino acid alterations. There were 133 (29.10%) RNA editing events changing Pro to Leu, 89 (19.47%) editing events changing Ser to Leu, and 65 (14.22%) RNA editing events changing Ser to Phe (Table S10). According to the physicochemical properties, 35.89% of non-synonymous codon changes were from hydrophilic to hydrophobic amino acids. Editing events mainly occurred at the first and second codon positions of the CDS, which accounted for 33.04% (151 sites) and 56.46% (258 sites) of all RNA editing sites, respectively (Table S10). We compared the RNA editing sites between *S. splendens* and *S. officinalis*. We found that *S. splendens* and *S. officinalis* shared 387 RNA editing sites. In contrast, *S. splendens and A. thaliana* mitogenomes shared only 201 RNA editing sites. The difference is consistent with their phylogenetic relationship.

Two of the most dramatic RNA editing events are the creation of new start and stop codons. We define the stop-gain as a stop codon obtained as a result of RNA editing. Two stop-gain resulting from the RNA editing events *cox2*-615 and *nad9*-289 were identified in the *S. splendens* mitochondrial RNAs (Table S10). The codon changes of the *cox2*-615 and *nad9*-289 were CAA to TAA and CGA to TGA (Table S10, Fig. S5K,W). The frequencies of these two events were 0.5 and 0.35, respectively.

To exclude the interference of SNPs on the identification of the RNA editing sites, we also detected the SNPs in the CDS of *S. splendens* mitogenome using the Illumina short reads. We detected 37 SNP sites in the CDS of the *S. splendens* mitogenome (Table S11). None of these SNP sites overlapped with the predicted RNA editing sites.

### Phylogenetic analysis based on the mitogenomes and plastomes of the Liamiales

**Fig. 7:**
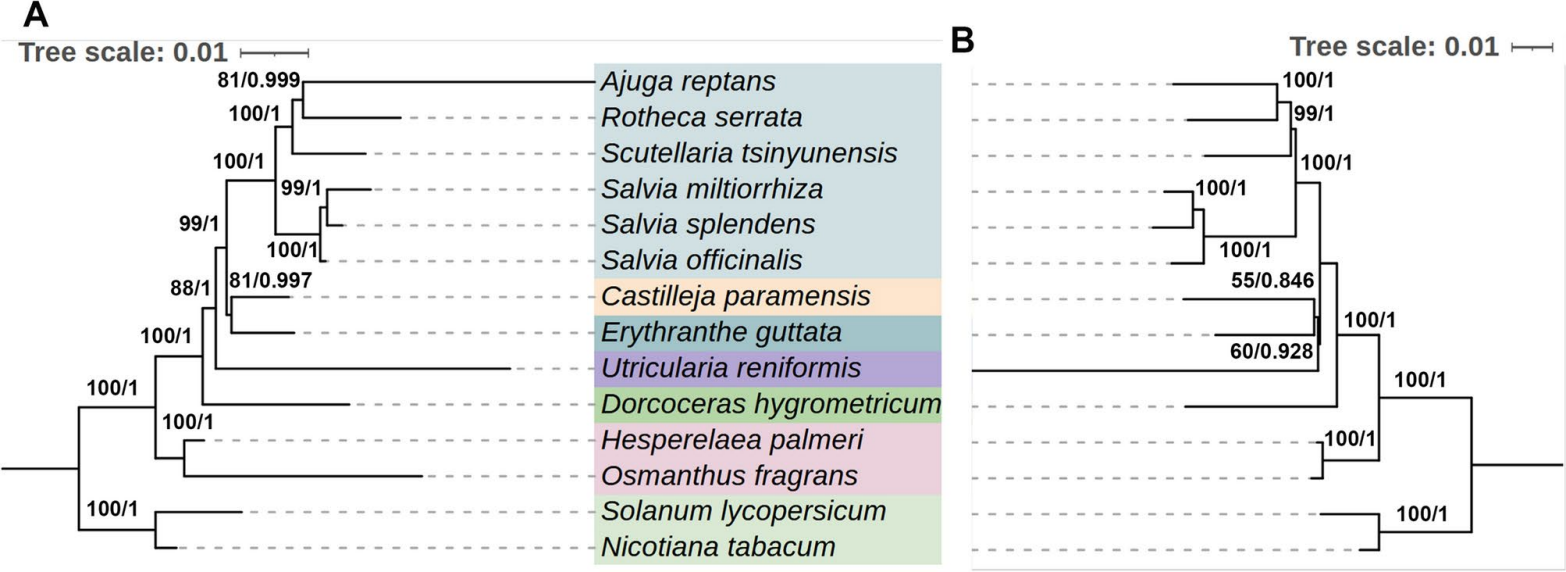
Molecular phylogenetic analysis based on the CDS of mitogenomes (**A**) and plastomes (**B**) in Lamiales. The tree was constructed using CDS of the 26 conserved genes of the mitogenomes of 12 Liamiales species and 2 outgroup species (**A**) and 56 CDS from the plastomes of these 14 species (**B**) via the ML and BI methods. The bootstrap score was obtained using 1000 replicates. The ML bootstrap support values and BI posterior probabilities were labeled at the corresponding nodes. Two Solanaceae species: *Nicotiana tabacum* and *Solanum lycopersicum*, were used as outgroups.

To infer the phylogeny of twelve Liamiales species based on mitogenomic sequences, we performed the phylogenetic analysis of twelve Lamiales species based on the CDS of 26 common mitogenomic genes in the 12 Lamiales mitogenomes. Based on the data matrix of the CDS, the maximum likelihood (ML) and the Bayesian inference (BI) trees were constructed by RAxML (v8.2.4) and MrBayes (v3.2.7)^41^, respectively. As shown, *S. splendens* was sister to *S. miltiorrhiza* and they were clustered in a branch with a bootstrap support value of 99 and a posterior probability of 1.00 for the ML and BI analyses, respectively (Fig. 7A). The branch of the *S. splendens* and *S. miltiorrhiza* was then clustered with the *S. officinalis* to form a branch with a bootstrap support value of 100 and a posterior probability of 1.00 (Fig. 7A). In parallel, we constructed the phylogenetic trees using the CDS of 56 plastid genes with the same methods. The phylogeny of 12 Liamiales species based on the mitogenomic sequences was consistent with that based on the plastome sequences (Fig. 7B).

### Genetic markers for interspecific identification based on mitochondrial introns

Initially, through the alignment of mitochondrial intron sequences of *S. miltiorrhiza*, *S. officinalis*, and *S. splendens*, multiple polymorphic loci were identified. Among them, 48 loci exhibiting variation between two or three species in the 12 introns (cox1i12, cox2i12, nad1i23, nad2i12, nad2i34, nad2i45, nad4i12, nad4i34, nad5i45, nad7i12, nad7i23, and nad7i34) were selected (Table S12). The naming convention for the introns follows the obvious study^60^. Out of these, 25 loci were single nucleotide polymorphisms (SNPs), while 23 were insertion-deletion type loci. Remarkably, one of the insertion/deletion polymorphic loci exhibited a substantial base number alteration of 65 bp, hence was not listed in the table.

**Fig. 8:**
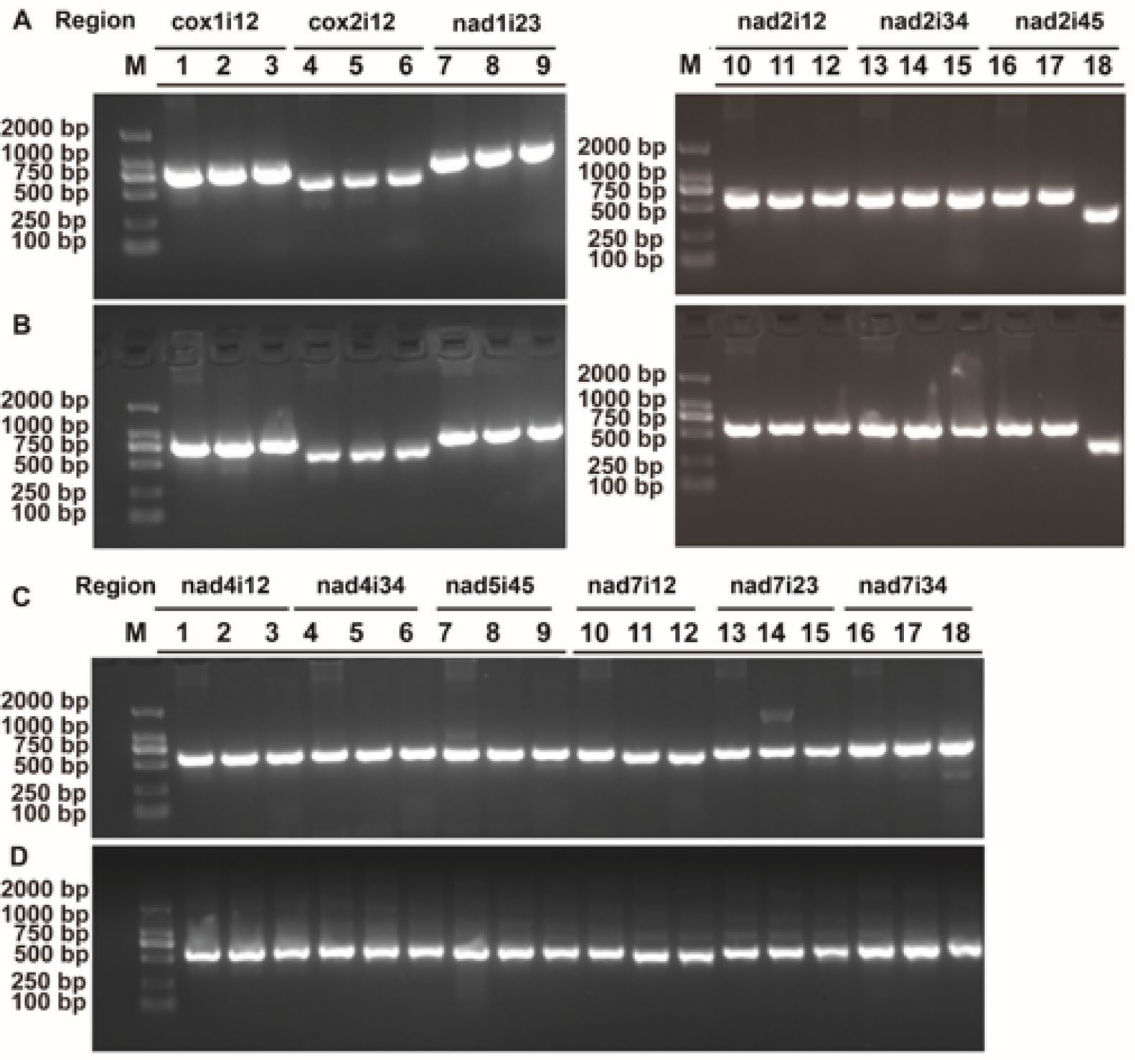
Agarose gel electrophoresis results of PCR products obtained from 12 molecular markers located in the intron regions of *S. miltiorrhiza*, *S. officinalis*, and *S. splendens*. Panel A: Lanes 1–18 display the PCR product bands for markers cox1i12, cox2i12, nad1i23, nad2i12, nad2i34, and nad2i45, corresponding to the first individual from each of the three species. Panel B: Lanes 1–18 display the bands for the six markers from panel A, but for the second individual of each species. Panel C: Lanes 1–18 display the PCR product bands for markers nad4i12, nad4i34, nad5i45, nad7i12, nad7i23, and nad7i34, corresponding to the first individual from each species. Panel D: Lanes 1–18 present the bands for the six markers from Panel C, corresponding to the second individual of each species.

**Fig. 9:**
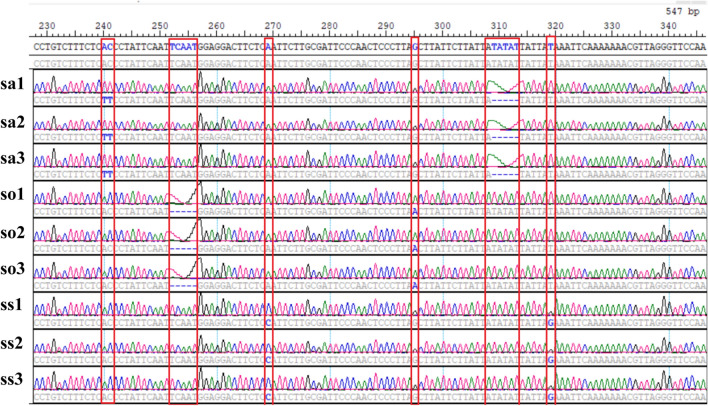
Detection of 7 polymorphic sites within the cox2i12 marker for three individuals of *S. miltiorrhiza*, *S. officinalis*, and *S. splendens* using Sanger sequencing.

To further explore these polymorphic loci, 12 primer pairs were designed to amplify these polymorphic loci (Table S13), and the resulting amplicons were sequenced employing Sanger sequencing. Through PCR amplification and Sanger sequencing, the variation loci were discerned within DNA samples obtained from three individuals of each species. Figure 8 shows the electrophoretic gel plot of amplicons generated from all 12 primer pairs, represented by two individuals from each of the three species. Sanger sequencing succeeded in revealing all the detected loci within the 12 introns (Fig. S6). All the variation loci within an intron of these 12 introns enabled three species to be distinguished. As an example, intron cox2i12 revealed nine polymorphic loci, enabling three species to be distinguished through two or more loci among these nine loci (Fig. 9, Fig. S6).

## Discussions

Investigations on the genome size, structure, gene content and orders, and intron content of plant mtDNA were crucial for understanding the diversity and evolution of plant mitogenomes. We recently analyzed the mitogenomes of two *Salvia* species: *S. miltiorrhiza* and *S. officinalis*^22,23^. To further determine the characteristics of the *Salvia* mitogenomes, we report here on the structure, gene contents, homologous recombination mediated by the repetitive sequences, and RNA editing events of the *S. splendens* mitogenome. In addition, a comparative analysis was conducted on the three mitogenomes to determine genetic diversity among the *Salvia* genus.

### All three *Salvia* species’ mitogenomes contained two circular molecules

We found that the major mitogenome configuration of *S. miltiorrhiza*, *S. officinalis,* and *S. splendens* contained two circular chromosomes. Moreover, we identified 9, 3, and 3 repetitive sequences in the three genomes that can mediate the homologous recombination The length of the longest repetitive sequence of *S. splendens* mitogenome was 13,331 bp (R01), which was more than two folds of the longest repetitive sequence of *S. miltiorrhiza* (5835 bp)^22^ and nearly fifteen folds of the longest repetitive sequence of *S. officinalis* (892 bp)^23^. All these long repetitive sequences were found to be able to mediate the homologous recombination, supported by the mapping results of Pacbio and Nanopore long reads. However, sequence comparison identified little sequence similarity among these repetitive sequences. In the future, they should be examined for possible conservation of high-level structure. We validated the homologous recombination mediated by the long repetitive sequence (13,331 bp) in the *S. splendens* mitogenome using PCR. Similarly, the recombination mediated by a ~ 16 kp repeat pair was also validated using PCR in the mitogenome of a holoparasitic plant *Aeginetia indica*. These two results showed that the long repetitive sequences also mediated the homologous recombination in plant mitogenome.

### MTPTs were found to be most abundant in the complete *S. splendens *mitogenome among the three *Salvia* species

Previous studies showed that MTPTs abundance correlates with both mitochondrial genome size and mtDNA noncoding content^61^. Here, we compared the MTPTs in the three *Salvia* species and found the total length of the MTPTs, ranging from 12,583 bp of *S. miltiorrhiza*^62^ to 28,175 bp of *S. splendens*. The total length of the MTPTs was 14,495 bp of *S. officinalis*^23^. However, the mitochondrial genome size of *S. miltiorrhiza* was 414,114 bp, larger than the 347,294 bp of *S. splendens* and 308,168 bp of *S. officinalis*. In these three *Salvia* species, the lengths of the MTPTs did not correlate with the mitochondrial genome size. For example, the *Carica papaya* mitogenome was 476,890 bp, larger than the *S. splendens*. However, the total MTPT length of *Carica papaya* was 15.1 kb, shorter than that of the *S. splendens*. Moreover, the length of the longest MTPT of *S. splendens* mitogenome was 13,674 bp (MTPT12), more than three folds of the longest MTPTs of *S. miltiorrhiza* (4,261 bp), and *S. officinalis* (4,987 bp).

Previous studies have suggested that the primary insertions of cpDNAs were large and then diverged and fragmented over evolutionary time^63^. Thus, it is possible that the MTPT12 was the primary insertion of cpDNA and didn’t fragment in the evolutionary process. Future studies are needed to determine the exact mechanism leading to the long MTPT in *S. splendens* and size variations in the congeneric mitogenomes.

### RNA editing events were conserved among two *Salvia* species

To gain a better understanding of RNA editing in *Salvia* mitochondria, we identified the editing sites of mitochondrial RNAs of *S. splendens* and compared them with those found in other *Salvia* species (*S. officinalis*) and *Arabidopsis thaliana*. 386 C-to-U modifications were shared between the CDS regions of the mitogenomes of *S. splendens* and *S. officinalis*. 201 C-to-U modifications were shared between the CDS regions of the mitogenomes of *S. splendens* and *A. thaliana*. Therefore, we can conclude that the RNA editing sites were more conserved on the intra-species level than the inter-species level. 201 C-to-U modifications were shared by CDS regions of the mitogenomes of two *Salvia* species (*S. splendens* and *S. officinalis*) and *A. thaliana*. This suggested that the inter-genus species hold nearly 50% of conserved RNA editing sites in their CDS regions of mitogenomes, in contrast, cogeneric species share 84% of conserved RNA editing sites.

### Mitochondrial intron markers for interspecific identification

Compared to nuclear DNA, organelle DNA offers advantages like multiple copies and uniparental inheritance, making sequences like the chloroplast barcode combination (*rbc*L + *mat*K + *trn*H-*psb*A) popular as molecular markers. However, DNA migration events in plant organelles cause some chloroplast DNAs to move to mitochondrial genomes, forming fragments known as MTPTs. The similarity between MTPTs and corresponding chloroplast DNA can be as high as 99.6%^64^. This leads to the co-amplification of chloroplast barcodes with MTPTs, resulting in unexpected marker sequences and potential plant misidentification^64^.

Recent advancements in plant mitochondrial genome research have spurred the development of molecular markers for interspecific identification based on polymorphisms in mitochondrial intron sequences. These markers can eliminate misidentification risks associated with chloroplast genome barcodes. For instance, one mitochondrial marker was developed to identify seven *Acer* species using a 33 bp insertion-deletion in the *nad*1 intron^65^. Additionally, two other markers were generated based on variant sites in the introns of the *nad*2 and *nad*4 genes to differentiate five *Amorphophallus* species^17^. In this study, we developed 12 intron markers derived from the polymorphisms in mitochondrial intron sequences, tailored for the identification of *S. miltiorrhiza*, *S. officinalis*, and *S. splendens*. These markers are superior to traditional chloroplast markers and may also be used for the identification of more species in the *Salvia* genus in the future.

## Conclusion

Our study showed that the major configuration of the *S. splendens* mitogenome contains two circular chromosomes. Multiple configurations are likely to coexist, which are converted from the major configuration through repeat-mediated recombination. 457 RNA editing sites including two stop codon gain sites were identified in the *S. splendens* mitogenome resulting from RNA editing. We developed 12 intron markers derived from the polymorphisms in mitochondrial intron sequences for the identification of *S. miltiorrhiza*, *S. officinalis*, and *S. splendens*.

## Supplementary Information

Supplementary Information 1.

Supplementary Information 2.

Supplementary Information 3.

Supplementary Information 4.

## Data Availability

The raw sequencing data from the Illumina and Nanopore platforms generated during the current study are available in GenBank. The associated BioProject, BioSample, and SRA numbers and the associated link are PRJNA947342, SAMN33848326, SRR23935050 for Illumina sequencing reads, and SRR23936021 for Nanopore sequencing reads. The mitogenome sequences along with the annotation information of them have been deposited in GenBank (https://www.ncbi.nlm.nih.gov/) with accession numbers: OQ675154 and OQ675155. The plant sample has been stored at the Herbarium of the Institute of Medicinal Plant Development, Beijing, China (Voucher Numbers: Implad20230116).
