# Supplementary material for: Unveiling the mitochondrial genome of Salvia splendens insights into the evolutionary traits within the genus Salvia
Source: Sci Rep. 2025 Apr 17;15:13344. doi: 10.1038/s41598-025-96637-9 (PMC12006378; doi:10.1038/s41598-025-96637-9)
Supplement: Supplementary file 1 — Supplementary Information 1. [file 41598_2025_96637_MOESM1_ESM.docx]

**SUPPLEMENTARY MATERIALS**

**Unveiling the mitochondrial genome of *Salvia splendens* insights into the evolutionary traits within the genus *Salvia***

Heyu Yang^1^, Yang Ni^1^, Jingling Li^1^, Haimei Chen^1*^, Chang Liu^1*^

^1^Institute of Medicinal Plant Development, Chinese Academy of Medical Sciences and Peking Union Medical College, Beijing 100193, PR China

*Correspondence: Chang Liu: cliu@implad.ac.cn; Tel: +86-10-57833111; Fax: +86-10- 62899715; Orcid-ID: 0000-0003-3879-7302; Haimei Chen: [hmchen@implad.ac.cn](mailto:hmchen@implad.ac.cn); Tel: +86-10-57833114

E-mails:

HYY: heyuyang@tju.edu.cn

YN: [ny_work@126.com](mailto:ny_work@126.com)

JLL: [lijingling1997@163.com](mailto:lijingling1997@163.com)

HMC: [hmchen@implad.ac.cn](mailto:hmchen@implad.ac.cn)

CL: cliu@implad.ac.cn

Table S1 Statistic summary of the sequencing data generated by Nanopore and Illumina platforms.

| Sequencing descriptors | Sequencing platform | |
| --- | --- | --- |
|  | Nanopore | Illumina |
| Total number of nucleotides  (raw data, bp) | 10,292,887,985 | 12,282,949,800 |
| Total Number of Reads | 389,320 | 81,886,332 |
| Mean Read Length (bp) | 26,438 | 150 |
| Total Number of Mapped Reads | 28,222 (MC1), 30,632 (MC2) | 619,287 (MC1), 857,276 (MC2) |
| Average Coverage Depth | 83.34 (MC1), 94.88 (MC2) | 469.77 (MC1), 705.60 (MC2) |

Table S2 The alignable regions of the *S. splendens* mitogenome assembled in this study and the one released in GenBank (PNBA02000024.1). “POS”: position.

| query | hit | query-POS | | hit-POS | | query-length | hit-length | identity(%) |
| --- | --- | --- | --- | --- | --- | --- | --- | --- |
| PNBA02000024.1 | this study | 264122 | 183457 | 1 | 80683 | 80666 | 80683 | 99.95 |
| PNBA02000024.1 | this study | 31723 | 1 | 67353 | 99079 | 31723 | 31727 | 99.99 |
| PNBA02000024.1 | this study | 347308 | 264123 | 99080 | 182239 | 83186 | 83160 | 99.83 |
| PNBA02000024.1 | this study | 123378 | 18393 | 182240 | 287225 | 104986 | 104986 | 99.99 |
| PNBA02000024.1 | this study | 196787 | 123379 | 273895 | 347294 | 73409 | 73400 | 99.81 |

Table S3 Variable sites between the *S. splendens* mitogenome released in GenBank (query, PNBA02000024.1) and assembled in this study (hit). “POS”: position, “REF: Reference allele, “ALT”: Alternative allele.

| query-POS | hit-POS | REF(hit) | ALT(query) |
| --- | --- | --- | --- |
| 35661 | 269957 | C | A |
| 106584 | 199034 | T | G |
| 284700 | 161662 | TCTTG | CCCTC |
| 284709 | 161653 | T | C |
| 284722 | 161640 | ATA | GTC |
| 284733 | 161629 | T | G |
| 284742 | 161620 | T |  |
| 284750 | 161613 | C | T |
| 284768 | 161595 | C | G |
| 284773 | 161590 | TGTACCCAAAA | CCT |
| 284785 | 161570 | T | C |
| 284793 | 161562 | TTG |  |
| 284817 | 161541 | TCAAAGGGTCAGT | CCAGAGGAGAAG |
| 284841 | 161516 | TCGTTGATT | CAATGGATC |
| 284856 | 161501 | C | T |
| 284924 | 161433 | AG |  |
| 284937 | 161422 | AAAGAA | TACGAC |
| 284947 | 161412 | GAA | CAG |
| 284963 | 161396 | G | A |
| 285011 | 161348 | C | T |
| 330836 | 115523 | AATTGGTT |  |
| 331998 | 114353 | T | G |
| 333419 | 112932 | T | G |
| 333990 | 112361 | T | G |
| 334042 | 112309 | C | T |
| 334153 | 112198 | AG |  |
| 334564 | 111789 | ATTCG |  |
| 335110 | 111248 | T | A |
| 338436 | 107922 | T | G |
| 338445 | 107913 | T | C |
| 338504 | 107854 | GC | TA |
| 338529 | 107829 | T | C |
| 338571 | 107787 | A | C |
| 338623 | 107735 | GATTT | AATGG |
| 338663 | 107695 | T | C |
| 342146 | 104212 | T |  |
| 345535 | 100822 | T |  |
| 345557 | 100801 | C |  |
| 345624 | 100735 | G |  |
| 345714 | 100644 | TTCC |  |
| 346136 | 100226 | A |  |
| 346211 | 100152 | C |  |
| 346235 | 100129 | T |  |
| 346355 | 100008 | G |  |
| 346391 | 99973 | CACCT | ACAACAC |
| 346414 | 99952 | T | G |
| 346425 | 99941 | G |  |
| 346439 | 99928 | A | G |
| 346515 | 99852 | TGCG | CTGACGT |
| 346537 | 99833 | TGCA | GGCCAG |
| 346552 | 99820 | TA | AC |
| 346572 | 99800 | AG | TAGA |
| 346586 | 99788 | A |  |
| 346624 | 99749 | GA |  |
| 346647 | 99728 | A | C |
| 346656 | 99719 | TGG | AGGC |
| 346664 | 99712 | G |  |
| 346676 | 99701 | AG | GAGA |
| 346692 | 99687 | AG | GAT |
| 346706 | 99674 | TAGA | TTAGG |
| 346716 | 99665 | T |  |
| 346724 | 99658 | GGAT | AGGAG |
| 346749 | 99634 | TTGCT | GTTTGCCTA |
| 346774 | 99613 | G | T |
| 346798 | 99589 | C |  |
| 346803 | 99585 | TCCT | CGCTC |
| 346822 | 99567 | A | C |
| 346827 | 99562 | C |  |
| 346901 | 99489 | G |  |
| 346926 | 99463 | A |  |
| 346971 | 99417 | G | T |
| 346984 | 99404 | CAG | TCAGT |
| 347032 | 99358 | T |  |
| 347036 | 99353 | TGTGTGT | GTGTTG |
| 221422 | 42718 | A | C |
| 261119 | 3021 | G | A |
| 261245 | 2895 | T | G |
| 261254 | 2886 | G | A |
| 261263 | 2877 | T | A |
| 261317 | 2823 | T | C |
| 261364 | 2776 | GATCCTATAG |  |
| 261415 | 2715 | C | T |
| 261435 | 2695 | C | T |
| 261456 | 2674 | C | A |
| 261469 | 2661 | A | G |
| 261545 | 2585 | ATTAATTT | TTTCATAA |
| 261573 | 2557 | AAATT |  |
| 261591 | 2534 | C | T |
| 261629 | 2496 | A | G |
| 261770 | 2355 | A | G |
| 261823 | 2302 | A | G |
| 261904 | 2221 | T | G |
| 261928 | 2197 | A | G |
| 262034 | 2091 | TG |  |
| 262082 | 2041 | A | C |
| 262088 | 2035 | A | C |
| 262154 | 1969 | C | T |
| 262173 | 1950 | C | T |
| 136716 | 333965 | C | T |
| 159306 | 311375 | TCTGGGCA | CTGCCCCG |
| 159321 | 311360 | AGGC | TGGT |
| 159332 | 311349 | C | T |
| 159338 | 311343 | TC | AG |
| 159345 | 311336 | GG | CA |
| 159374 | 311307 | G | T |
| 159379 | 311302 | A | C |
| 159386 | 311295 | C | A |
| 159396 | 311285 | G | A |
| 159404 | 311277 | G | A |
| 159562 | 311119 | T |  |
| 159589 | 311093 | AG | GA |
| 159608 | 311074 | CAACTCTCTCT | GAACCATGTCG |
| 159623 | 311059 | TT |  |
| 159631 | 311053 | CTAG | TA |
| 159637 | 311045 | A | T |
| 159644 | 311038 | G | A |
| 196788 | 273894 | TC | CG |
| 3906 | 95174 | CGATTC | AT |
| 31724 | 67352 | CG | TC |
| 133139 | 337534 | C | T |
| 133697 | 336976 | CATT |  |
| 133861 | 336808 | T | C |
| 133925 | 336744 | C | T |
| 133976 | 336693 | A |  |
| 134037 | 336631 | A |  |
| 134105 | 336564 | C | T |
| 134138 | 336531 | T | G |
| 134145 | 336524 | C | A |
| 134237 | 336432 | C | A |
| 134283 | 336386 | A | G |
| 134358 | 336311 | C | A |
| 134367 | 336302 | T | G |
| 134400 | 336269 | A |  |
| 134513 | 336157 | A | G |
| 134526 | 336144 | A | G |
| 134535 | 336135 | TC | CT |
| 134586 | 336084 | C | T |
| 134694 | 335976 | ATATT |  |
| 134738 | 335937 | G | A |
| 134868 | 335807 | G | A |
| 135054 | 335621 | A | G |
| 135105 | 335570 | T | G |
| 135223 | 335452 | ACGTAGAGC |  |
| 135244 | 335422 | AC | GG |
| 135290 | 335376 | G | A |
| 135498 | 335168 | ATTTA | CTTTC |
| 135573 | 335093 | TC | GG |
| 135585 | 335081 | AAAT |  |
| 135591 | 335071 | C | A |
| 135617 | 335045 | G | A |
| 135641 | 335021 | A | G |
| 135652 | 335010 | G | C |
| 135774 | 334888 | G | A |
| 135798 | 334864 | C | A |
| 135876 | 334786 | C | T |
| 135888 | 334774 | T | G |
| 135910 | 334752 | AATCTTGAG |  |
| 136077 | 334576 | C | G |
| 136093 | 334560 | A | T |
| 136153 | 334500 | G | C |
| 136203 | 334450 | T | G |
| 136272 | 334381 | C | G |
| 136626 | 334027 | T | G |
| 136662 | 333991 | C | T |
| 136667 | 333986 | T | C |
| 136682 | 333971 | A |  |

Table S4 Results of Nanopore long reads mapped to the four possible configurations associated with 53 repetitive sequences (R02,03,05,06,07-56). MC1/2: mitogenome chromosome 1/2. The percentage of minor configurations was calculated as the number of reads mapped to them divided by those mapped to all configurations.

| Repetitive Sequences ID | Query Sequence | Subject Sequence | Alignment Length | Repeat Copy 1 | | | Repeat Copy 2 | | | Numbers of Long Reads Mapped to Each DBS | | | | Recombination Frequency (%) |
| --- | --- | --- | --- | --- | --- | --- | --- | --- | --- | --- | --- | --- | --- | --- |
|  |  |  |  | Start | End | Strand | Start | End | Strand | c1 | c2 | c3 | c4 |  |
| R02 | MC1 | MC2 | 887 | 112992 | 113855 | plus | 43631 | 42778 | minus | 240 | 79 | 0 | 0 | 0.00% |
| R03 | MC1 | MC2 | 466 | 137051 | 137516 | plus | 40164 | 40629 | plus | 58 | 89 | 0 | 0 | 0.00% |
| R05 | MC1 | MC2 | 371 | 68220 | 68585 | plus | 15378 | 15743 | plus | 144 | 75 | 1 | 2 | 1.35% |
| R06 | MC1 | MC2 | 134 | 115453 | 115585 | plus | 131702 | 131578 | minus | 0 | 84 | 0 | 0 | 0.00% |
| R08 | MC1 | MC2 | 113 | 79372 | 79484 | plus | 9715 | 9603 | minus | 122 | 82 | 0 | 0 | 0.00% |
| R09 | MC2 | MC2 | 113 | 103675 | 103787 | plus | 9715 | 9603 | minus | 122 | 82 | 0 | 0 | 0.00% |
| R10 | MC2 | MC2 | 110 | 2327 | 2433 | plus | 59607 | 59712 | plus | 83 | 77 | 0 | 0 | 0.00% |
| R11 | MC2 | MC2 | 77 | 18278 | 18354 | plus | 36065 | 36141 | plus | 84 | 75 | 1 | 0 | 0.00% |
| R12 | MC2 | MC2 | 71 | 118087 | 118017 | minus | 56119 | 56189 | plus | 93 | 78 | 1 | 0 | 0.00% |
| R13 | MC2 | MC2 | 68 | 36699 | 36766 | plus | 147555 | 147622 | plus | 71 | 87 | 0 | 0 | 0.00% |
| R14 | MC1 | MC2 | 64 | 133141 | 133204 | plus | 36094 | 36157 | plus | 60 | 84 | 0 | 0 | 0.00% |
| R15 | MC1 | MC2 | 62 | 92220 | 92281 | plus | 33643 | 33582 | minus | 60 | 88 | 0 | 0 | 0.00% |
| R16 | MC2 | MC2 | 60 | 41882 | 41941 | plus | 147224 | 147165 | minus | 72 | 83 | 1 | 0 | 0.00% |
| R17 | MC1 | MC2 | 53 | 32019 | 32071 | plus | 15716 | 15768 | plus | 65 | 75 | 0 | 0 | 0.00% |
| R18 | MC1 | MC1 | 52 | 170939 | 170888 | minus | 157139 | 157190 | plus | 89 | 88 | 0 | 2 | 0.00% |
| R19 | MC1 | MC2 | 51 | 14322 | 14372 | plus | 63488 | 63538 | plus | 80 | 70 | 0 | 0 | 0.00% |
| R20 | MC1 | MC2 | 48 | 133141 | 133188 | plus | 18307 | 18354 | plus | 61 | 75 | 0 | 0 | 0.00% |
| R21 | MC1 | MC2 | 45 | 86698 | 86742 | plus | 39479 | 39435 | minus | 50 | 88 | 0 | 0 | 0.00% |
| R22 | MC2 | MC2 | 45 | 39120 | 39164 | plus | 59964 | 59920 | minus | 84 | 84 | 1 | 0 | 0.00% |
| R23 | MC1 | MC2 | 43 | 87531 | 87573 | plus | 147583 | 147625 | plus | 55 | 71 | 0 | 0 | 0.00% |
| R24 | MC1 | MC2 | 43 | 5973 | 6015 | plus | 144096 | 144138 | plus | 76 | 83 | 0 | 0 | 0.00% |
| R25 | MC1 | MC1 | 42 | 81229 | 81270 | plus | 162686 | 162727 | plus | 92 | 47 | 0 | 0 | 0.00% |
| R26 | MC1 | MC1 | 41 | 136957 | 136917 | minus | 32253 | 32293 | plus | 61 | 64 | 0 | 0 | 0.00% |
| R27 | MC1 | MC1 | 41 | 129650 | 129690 | plus | 130432 | 130472 | plus | 62 | 61 | 0 | 0 | 0.00% |
| R28 | MC1 | MC1 | 40 | 145470 | 145509 | plus | 157171 | 157132 | minus | 88 | 80 | 0 | 0 | 0.00% |
| R29 | MC1 | MC2 | 40 | 87531 | 87570 | plus | 36727 | 36766 | plus | 55 | 87 | 0 | 0 | 0.00% |
| R30 | MC1 | MC1 | 39 | 92777 | 92815 | plus | 141958 | 141996 | plus | 75 | 58 | 0 | 0 | 0.00% |
| R31 | MC1 | MC2 | 38 | 14321 | 14358 | plus | 68425 | 68462 | plus | 80 | 70 | 0 | 0 | 0.00% |
| R32 | MC2 | MC2 | 38 | 39137 | 39100 | minus | 36659 | 36696 | plus | 84 | 87 | 0 | 0 | 0.00% |
| R33 | MC2 | MC2 | 38 | 120154 | 120191 | plus | 150690 | 150653 | minus | 67 | 83 | 0 | 0 | 0.00% |
| R34 | MC1 | MC1 | 37 | 13241 | 13277 | plus | 69008 | 69044 | plus | 81 | 150 | 0 | 0 | 0.00% |
| R35 | MC1 | MC2 | 37 | 157426 | 157462 | plus | 37703 | 37667 | minus | 88 | 85 | 0 | 0 | 0.00% |
| R36 | MC1 | MC2 | 37 | 13241 | 13277 | plus | 93311 | 93347 | plus | 81 | 150 | 0 | 0 | 0.00% |
| R37 | MC2 | MC2 | 37 | 43571 | 43607 | plus | 141060 | 141024 | minus | 82 | 87 | 0 | 0 | 0.00% |
| R38 | MC1 | MC2 | 36 | 34449 | 34484 | plus | 27303 | 27338 | plus | 67 | 78 | 0 | 0 | 0.00% |
| R39 | MC1 | MC1 | 35 | 152263 | 152297 | plus | 175443 | 175477 | plus | 91 | 86 | 0 | 0 | 0.00% |
| R40 | MC1 | MC2 | 35 | 166724 | 166758 | plus | 46788 | 46822 | plus | 92 | 87 | 0 | 0 | 0.00% |
| R41 | MC1 | MC2 | 35 | 159760 | 159794 | plus | 15180 | 15146 | minus | 96 | 82 | 0 | 0 | 0.00% |
| R42 | MC1 | MC2 | 34 | 14632 | 14665 | plus | 129456 | 129423 | minus | 78 | 85 | 0 | 0 | 0.00% |
| R43 | MC1 | MC2 | 33 | 155353 | 155385 | plus | 64856 | 64824 | minus | 90 | 70 | 0 | 0 | 0.00% |
| R44 | MC1 | MC1 | 33 | 145470 | 145502 | plus | 170907 | 170939 | plus | 89 | 79 | 0 | 0 | 0.00% |
| R45 | MC1 | MC1 | 32 | 49062 | 49093 | plus | 166464 | 166433 | minus | 92 | 82 | 0 | 0 | 0.00% |
| R46 | MC1 | MC1 | 32 | 144146 | 144177 | plus | 157131 | 157100 | minus | 77 | 88 | 0 | 0 | 0.00% |
| R47 | MC1 | MC2 | 32 | 92825 | 92856 | plus | 36883 | 36852 | minus | 58 | 87 | 0 | 0 | 0.00% |
| R48 | MC1 | MC2 | 32 | 156565 | 156596 | plus | 56087 | 56118 | plus | 91 | 78 | 0 | 0 | 0.00% |
| R49 | MC1 | MC1 | 32 | 32086 | 32117 | plus | 176670 | 176701 | plus | 92 | 65 | 0 | 0 | 0.00% |
| R50 | MC1 | MC1 | 31 | 57984 | 58014 | plus | 142440 | 142410 | minus | 76 | 78 | 0 | 0 | 0.00% |
| R51 | MC2 | MC2 | 31 | 39050 | 39080 | plus | 39943 | 39973 | plus | 88 | 84 | 0 | 0 | 0.00% |
| R52 | MC2 | MC2 | 31 | 145090 | 145060 | minus | 117522 | 117552 | plus | 79 | 93 | 0 | 0 | 0.00% |
| R53 | MC1 | MC2 | 30 | 170909 | 170938 | plus | 25374 | 25403 | plus | 89 | 73 | 0 | 0 | 0.00% |
| R54 | MC2 | MC1 | 30 | 25404 | 25375 | minus | 162647 | 162676 | plus | 92 | 74 | 0 | 0 | 0.00% |
| R55 | MC1 | MC2 | 30 | 145472 | 145501 | plus | 25374 | 25403 | plus | 79 | 73 | 0 | 0 | 0.00% |
| R56 | MC1 | MC2 | 30 | 133079 | 133108 | plus | 43355 | 43384 | plus | 62 | 82 | 0 | 0 | 0.00% |

Table S5 PCR primers used to detect the recombination products of the three repetitive sequences in the *S. splendens* mitogenome*.*

| Primer Name | DBS ID | Repetitive sequence ID | Primer Sequence (5’->3’) |
| --- | --- | --- | --- |
| R01-F1 | DBS01 | R01 | GGCTTTCCCCTTTCGCTACT |
| R01-R1 | DBS01 | R01 | AGTTGAGCTGCCTGAGCTTG |
| R01-F2 | DBS01 | R01 | GCTTTCAATTCGCCTCTGACC |
| R01-R2 | DBS01 | R01 | ACGAGGAAGACAACTCAGCG |
| R04-F1 | DBS02 | R04 | TTTTGTGCCGTAGCTTGCG |
| R04-R1 | DBS02 | R04 | GAGGATTCCCCGAATACCTGAAA |
| R04-F2 | DBS02 | R04 | CCCGGACCTCCTTCTTTACC |
| R04-R2 | DBS02 | R04 | CCGAGGGTACAAACCCTATTTTC |
| R07-F1 | DBS03 | R07 | TAGGGCTTTCGGGTATGCCA |
| R07-R1 | DBS03 | R07 | GCCATCAGGAGCGCTAGAAT |
| R07-F2 | DBS03 | R07 | TGCGCTACCTTCTGTGAGTG |
| R07-R2 | DBS03 | R07 | CGTAGCAAGAGCGCGAAAAA |

Table S6 Summary of the simple sequence repeats found in the *S. splendens* mitogenome*.* “MC1/2”: mitogenome chromosome 1/2.

| Chromosomes | Repeat Unit | Number of Repeat Units | | | | | | | | | | | | | | | |
| --- | --- | --- | --- | --- | --- | --- | --- | --- | --- | --- | --- | --- | --- | --- | --- | --- | --- |
|  |  | 3 | 4 | 5 | 6 | 7 | 8 | 9 | 10 | 11 | 12 | 13 | 14 | 15 | 16 | 17 | total |
| MC1 | A/T | - | - | - | - | - | - | - | 4 | 1 | 1 | - | - | - | - | 1 | 7 |
| MC1 | AG/CT | - | - | 5 | 2 | - | - | - | - | - | - | - | - | - | - | - | 7 |
| MC1 | AT/AT | - | - | 2 | - | 1 | 1 | - | - | - | - | - | - | - | - | - | 4 |
| MC1 | AAG/CTT | - | 1 |  | - | - | - | - | - | - | - | - | - | - | - | - | 1 |
| MC1 | AAT/ATT | - | 3 | 1 | - | - | - | - | - | - | - | - | - | - | - | - | 4 |
| MC1 | ACG/CGT | - | 1 | - | - | - | - | - | - | - | - | - | - | - | - | - | 1 |
| MC1 | ACT/AGT | - | 2 | 1 | - | - | - | - | - | - | - | - | - | - | - | - | 3 |
| MC1 | AAAG/CTTT | 10 | - | - | - | - | - | - | - | - | - | - | - | - | - | - | 10 |
| MC1 | AAAT/ATTT | 1 | - | - | - | - | - | - | - | - | - | - | - | - | - | - | 1 |
| MC1 | AACG/CGTT | 1 | - | - | - | - | - | - | - | - | - | - | - | - | - | - | 1 |
| MC1 | AAGG/CCTT | 1 | - | - | - | - | - | - | - | - | - | - | - | - | - | - | 1 |
| MC1 | AATC/ATTG | 1 | - | - | - | - | - | - | - | - | - | - | - | - | - | - | 1 |
| MC1 | ACTC/AGTG | 1 | - | - | - | - | - | - | - | - | - | - | - | - | - | - | 1 |
| MC1 | AGAT/ATCT | 1 | - | - | - | - | - | - | - | - | - | - | - | - | - | - | 1 |
| MC1 | AGCC/CTGG | 1 | - | - | - | - | - | - | - | - | - | - | - | - | - | - | 1 |
| MC1 | AGCG/CGCT | 1 | - | - | - | - | - | - | - | - | - | - | - | - | - | - | 1 |
| MC1 | AGCT/AGCT | 1 | - | - | - | - | - | - | - | - | - | - | - | - | - | - | 1 |
| MC1 | AGGC/CCTG | 1 | - | - | - | - | - | - | - | - | - | - | - | - | - | - | 1 |
| MC1 | AAAAG/CTTTT | 1 | - | - | - | - | - | - | - | - | - | - | - | - | - | - | 1 |
| MC1 | AAGAC/CTTGT | 1 | - | - | - | - | - | - | - | - | - | - | - | - | - | - | 1 |
| MC1 | AATAC/ATTGT | 1 | - | - | - | - | - | - | - | - | - | - | - | - | - | - | 1 |
| MC1 | ACTAG/AGTCT | 1 | - | - | - | - | - | - | - | - | - | - | - | - | - | - | 1 |
| MC1 | AAATAT/ATATTT | 1 | - | - | - | - | - | - | - | - | - | - | - | - | - | - | 1 |
| MC1 | AATAGT/ACTATT | 1 | - | - | - | - | - | - | - | - | - | - | - | - | - | - | 1 |
| MC2 | A/T | - | - | - | - | - | - | - | 10 | 4 | - | - | - | - | - | - | 14 |
| MC2 | AG/CT | - | - | 6 |  | - | - | - | - | - | - | - | - | - | - | - | 6 |
| MC2 | AT/AT | - | - | - | 1 | 1 | - | - | - | - | - | - | - | - | - | - | 2 |
| MC2 | AAT/ATT | - | 1 | 1 | - | - | - | - | - | - | - | - | - | - | - | - | 2 |
| MC2 | ACT/AGT | - | 1 | - | - | - | - | - | - | - | - | - | - | - | - | - | 1 |
| MC2 | AAAC/GTTT | 1 | - | - | - | - | - | - | - | - | - | - | - | - | - | - | 1 |
| MC2 | AAAG/CTTT | 6 | - | - | - | - | - | - | - | - | - | - | - | - | - | - | 6 |
| MC2 | AAGG/CCTT | 1 | - | - | - | - | - | - | - | - | - | - | - | - | - | - | 1 |
| MC2 | AATC/ATTG | 2 | - | - | - | - | - | - | - | - | - | - | - | - | - | - | 2 |
| MC2 | AATG/ATTC | 2 | - | - | - | - | - | - | - | - | - | - | - | - | - | - | 2 |
| MC2 | AAGCT/AGCTT | 1 | - | - | - | - | - | - | - | - | - | - | - | - | - | - | 1 |
| MC2 | AATAC/ATTGT | 1 | - | - | - | - | - | - | - | - | - | - | - | - | - | - | 1 |
| MC2 | ACAGC/CTGTG | 1 | - | - | - | - | - | - | - | - | - | - | - | - | - | - | 1 |

Table S7 The detailed information of the simple sequence repeats in the *S. splendens* mitogenome*.* “MC1/2”: mitogenome chromosome 1/2.

| Chromosome | ID | Repeat type | Repeat unit | Repeat type | Start | End | Repeat type |
| --- | --- | --- | --- | --- | --- | --- | --- |
| MC1 | 1 | mononucleotide | (T)10 | 10 | 10594 | 10603 | 1 |
| MC1 | 2 | mononucleotide | (A)12 | 12 | 21533 | 21544 | 1 |
| MC1 | 3 | mononucleotide | (A)10 | 10 | 54004 | 54013 | 1 |
| MC1 | 4 | mononucleotide | (T)11 | 11 | 108169 | 108179 | 1 |
| MC1 | 5 | mononucleotide | (T)10 | 10 | 123672 | 123681 | 1 |
| MC1 | 6 | mononucleotide | (T)10 | 10 | 126770 | 126779 | 1 |
| MC1 | 7 | mononucleotide | (A)17 | 17 | 134260 | 134276 | 1 |
| MC1 | 8 | dinucleotide | (AT)5 | 10 | 1645 | 1654 | 2 |
| MC1 | 9 | dinucleotide | (AT)5 | 10 | 41073 | 41082 | 2 |
| MC1 | 10 | dinucleotide | (TC)6 | 12 | 44405 | 44416 | 2 |
| MC1 | 11 | dinucleotide | (CT)6 | 12 | 50210 | 50221 | 2 |
| MC1 | 12 | dinucleotide | (AG)5 | 10 | 54348 | 54357 | 2 |
| MC1 | 13 | dinucleotide | (CT)5 | 10 | 93161 | 93170 | 2 |
| MC1 | 14 | dinucleotide | (CT)5 | 10 | 99972 | 99981 | 2 |
| MC1 | 15 | dinucleotide | (TA)8 | 16 | 123456 | 123471 | 2 |
| MC1 | 16 | dinucleotide | (AG)5 | 10 | 130185 | 130194 | 2 |
| MC1 | 17 | dinucleotide | (AG)5 | 10 | 147787 | 147796 | 2 |
| MC1 | 18 | trinucleotide | (AGA)4 | 12 | 3468 | 3479 | 3 |
| MC1 | 19 | trinucleotide | (GTC)4 | 12 | 6390 | 6401 | 3 |
| MC1 | 20 | trinucleotide | (TTA)4 | 12 | 66238 | 66249 | 3 |
| MC1 | 21 | trinucleotide | (ATT)5 | 15 | 72517 | 72531 | 3 |
| MC1 | 22 | trinucleotide | (TAG)4 | 12 | 110888 | 110899 | 3 |
| MC1 | 23 | trinucleotide | (AAT)4 | 12 | 163410 | 163421 | 3 |
| MC1 | 24 | trinucleotide | (TAG)4 | 12 | 181778 | 181789 | 3 |
| MC1 | 25 | tetranucleotide | (TGCC)3 | 12 | 5798 | 5809 | 4 |
| MC1 | 26 | tetranucleotide | (TTCT)3 | 12 | 9697 | 9708 | 4 |
| MC1 | 27 | tetranucleotide | (TCTT)3 | 12 | 29144 | 29155 | 4 |
| MC1 | 28 | tetranucleotide | (TTTC)3 | 12 | 38509 | 38520 | 4 |
| MC1 | 29 | tetranucleotide | (AATC)3 | 12 | 45825 | 45836 | 4 |
| MC1 | 30 | tetranucleotide | (AATA)3 | 12 | 59024 | 59035 | 4 |
| MC1 | 31 | tetranucleotide | (TTTC)3 | 12 | 59748 | 59759 | 4 |
| MC1 | 32 | tetranucleotide | (AGAT)3 | 12 | 61619 | 61630 | 4 |
| MC1 | 33 | tetranucleotide | (CCAG)3 | 12 | 97274 | 97285 | 4 |
| MC1 | 34 | tetranucleotide | (GAAA)3 | 12 | 98137 | 98148 | 4 |
| MC1 | 35 | tetranucleotide | (CGAA)3 | 12 | 137724 | 137735 | 4 |
| MC1 | 36 | tetranucleotide | (TGAG)3 | 12 | 139569 | 139580 | 4 |
| MC1 | 37 | tetranucleotide | (GCTA)3 | 12 | 140098 | 140109 | 4 |
| MC1 | 38 | tetranucleotide | (AAGA)3 | 12 | 141591 | 141602 | 4 |
| MC1 | 39 | tetranucleotide | (TCCT)3 | 12 | 142448 | 142459 | 4 |
| MC1 | 40 | tetranucleotide | (AGAA)3 | 12 | 148401 | 148412 | 4 |
| MC1 | 41 | tetranucleotide | (AAGA)3 | 12 | 156663 | 156674 | 4 |
| MC1 | 42 | tetranucleotide | (AAGA)3 | 12 | 157890 | 157901 | 4 |
| MC1 | 43 | tetranucleotide | (GCTC)3 | 12 | 175229 | 175240 | 4 |
| MC1 | 44 | tetranucleotide | (TTTC)3 | 12 | 182079 | 182090 | 4 |
| MC1 | 45 | pentanucleotide | (ACTAG)3 | 15 | 51359 | 51373 | 5 |
| MC1 | 46 | pentanucleotide | (TATTG)3 | 15 | 98509 | 98523 | 5 |
| MC1 | 47 | pentanucleotide | (CTTTT)3 | 15 | 145265 | 145279 | 5 |
| MC1 | 48 | pentanucleotide | (TCTTG)3 | 15 | 172697 | 172711 | 5 |
| MC1 | 49 | hexanucleotide | (TTTATA)3 | 18 | 34935 | 34952 | 6 |
| MC1 | 50 | compound | NA | 92 | 70609 | 70700 | compound |
| MC1 | 51 | compound | NA | 28 | 102350 | 102377 | compound |
| MC2 | 52 | mononucleotide | (T)11 | 11 | 2022 | 2032 | 1 |
| MC2 | 53 | mononucleotide | (A)10 | 10 | 11874 | 11883 | 1 |
| MC2 | 54 | mononucleotide | (T)10 | 10 | 29082 | 29091 | 1 |
| MC2 | 55 | mononucleotide | (T)11 | 11 | 33699 | 33709 | 1 |
| MC2 | 56 | mononucleotide | (T)10 | 10 | 81171 | 81180 | 1 |
| MC2 | 57 | mononucleotide | (A)10 | 10 | 81387 | 81396 | 1 |
| MC2 | 58 | mononucleotide | (T)10 | 10 | 86477 | 86486 | 1 |
| MC2 | 59 | mononucleotide | (A)10 | 10 | 130090 | 130099 | 1 |
| MC2 | 60 | mononucleotide | (T)10 | 10 | 143267 | 143276 | 1 |
| MC2 | 61 | mononucleotide | (T)11 | 11 | 144289 | 144299 | 1 |
| MC2 | 62 | mononucleotide | (A)10 | 10 | 144557 | 144566 | 1 |
| MC2 | 63 | mononucleotide | (A)10 | 10 | 154454 | 154463 | 1 |
| MC2 | 64 | mononucleotide | (A)10 | 10 | 157199 | 157208 | 1 |
| MC2 | 65 | dinucleotide | (CT)5 | 10 | 54707 | 54716 | 2 |
| MC2 | 66 | dinucleotide | (TC)5 | 10 | 59244 | 59253 | 2 |
| MC2 | 67 | dinucleotide | (AG)5 | 10 | 68678 | 68687 | 2 |
| MC2 | 68 | dinucleotide | (TA)6 | 12 | 90586 | 90597 | 2 |
| MC2 | 69 | dinucleotide | (GA)5 | 10 | 115592 | 115601 | 2 |
| MC2 | 70 | dinucleotide | (CT)5 | 10 | 135987 | 135996 | 2 |
| MC2 | 71 | dinucleotide | (TC)5 | 10 | 152588 | 152597 | 2 |
| MC2 | 72 | trinucleotide | (CTA)4 | 12 | 45127 | 45138 | 3 |
| MC2 | 73 | trinucleotide | (ATT)5 | 15 | 96820 | 96834 | 3 |
| MC2 | 74 | tetranucleotide | (GAAA)3 | 12 | 13224 | 13235 | 4 |
| MC2 | 75 | tetranucleotide | (TTTC)3 | 12 | 20343 | 20354 | 4 |
| MC2 | 76 | tetranucleotide | (CATT)3 | 12 | 34715 | 34726 | 4 |
| MC2 | 77 | tetranucleotide | (TTTC)3 | 12 | 42448 | 42459 | 4 |
| MC2 | 78 | tetranucleotide | (TTGA)3 | 12 | 47760 | 47771 | 4 |
| MC2 | 79 | tetranucleotide | (GGAA)3 | 12 | 53203 | 53214 | 4 |
| MC2 | 80 | tetranucleotide | (TTCT)3 | 12 | 55644 | 55655 | 4 |
| MC2 | 81 | tetranucleotide | (AATC)3 | 12 | 61849 | 61860 | 4 |
| MC2 | 82 | tetranucleotide | (GAAA)3 | 12 | 62656 | 62667 | 4 |
| MC2 | 83 | tetranucleotide | (CATT)3 | 12 | 73604 | 73615 | 4 |
| MC2 | 84 | tetranucleotide | (AACA)3 | 12 | 106846 | 106857 | 4 |
| MC2 | 85 | pentanucleotide | (ACAGC)3 | 15 | 42678 | 42692 | 5 |
| MC2 | 86 | pentanucleotide | (AGCTA)3 | 15 | 123962 | 123976 | 5 |
| MC2 | 87 | pentanucleotide | (TATTG)3 | 15 | 124771 | 124785 | 5 |
| MC2 | 88 | compound | NA | 92 | 94912 | 95003 | compound |
| MC2 | 89 | compound | NA | 27 | 135185 | 135211 | compound |

Table S8 The detailed information of long tandem repeats in the mitogenome of *S. splendens.*

| Chromosomes | ID | Indices | Period size(bp) | Copy Number | Consensus Size (bp) | Percent Matches | Percent Indels | Score | Bases number | | | | Entropy |
| --- | --- | --- | --- | --- | --- | --- | --- | --- | --- | --- | --- | --- | --- |
|  |  |  |  |  |  |  |  |  | A | C | G | T | (0–2) |
| MC1 | 1 | 41069-41103 | 17 | 2.1 | 17 | 89 | 10 | 54 | 51 | 0 | 0 | 48 | 1 |
| MC1 | 2 | 62581-62639 | 26 | 2.3 | 26 | 79 | 8 | 66 | 44 | 15 | 38 | 1 | 1.56 |
| MC1 | 3 | 111756-111823 | 11 | 6.5 | 11 | 73 | 20 | 58 | 30 | 2 | 17 | 48 | 1.62 |
| MC1 | 4 | 111761-111825 | 18 | 3.8 | 17 | 88 | 9 | 80 | 30 | 3 | 18 | 47 | 1.64 |
| MC1 | 5 | 132851-132886 | 12 | 3 | 12 | 100 | 0 | 72 | 41 | 0 | 8 | 50 | 1.33 |
| MC1 | 6 | 132851-132894 | 12 | 3.7 | 11 | 93 | 6 | 61 | 40 | 0 | 11 | 47 | 1.39 |
| MC1 | 7 | 134806-134831 | 13 | 2 | 13 | 100 | 0 | 52 | 53 | 0 | 0 | 46 | 1 |
| MC1 | 8 | 141519-141548 | 16 | 1.9 | 15 | 93 | 6 | 51 | 83 | 0 | 16 | 0 | 0.65 |
| MC2 | 9 | 32285-32324 | 15 | 2.5 | 16 | 88 | 8 | 55 | 70 | 2 | 17 | 10 | 1.27 |
| MC2 | 10 | 51920-51972 | 23 | 2.3 | 22 | 81 | 9 | 70 | 26 | 20 | 22 | 30 | 1.98 |

Table S9 List of Mitochondrial Plastid DNAs (MTPT) in the *S. splendens* mitogenome. “MC1/2”: chromosome 1/2.

| MTPT ID | Name of Circular Molecule | Identity（%） | Alignment Length (bp) | Number of | Number of Gap Openings | Positions on the Chloroplast Genome | | Positions on the Mitochondrial Genome | | Genes Located in the MTPT Fragments | E value | Score |
| --- | --- | --- | --- | --- | --- | --- | --- | --- | --- | --- | --- | --- |
|  |  |  |  | Mismatches |  | Start | End | Start | End |  |  |  |
| MTPT01 | MC1 | 100 | 1220 | 0 | 0 | 21851 | 23070 | 3078 | 1859 |  | 0 | 2254 |
| MTPT02 | MC1 | 100 | 1918 | 0 | 0 | 23127 | 25044 | 42050 | 43967 | *rpoB* fragment | 0 | 3542 |
| MTPT03 | MC1 | 88.764 | 178 | 10 | 5 | 44949 | 45126 | 95011 | 94844 |  | 3.67E-53 | 209 |
| MTPT04 | MC1 | 91.111 | 180 | 16 | 0 | 44211 | 44390 | 95196 | 95017 | *trn*S-GGA | 1.01E-63 | 244 |
| MTPT05 | MC1 | 95.192 | 104 | 5 | 0 | 29497 | 29600 | 95738 | 95841 | *trn*D-GUC | 8.06E-40 | 165 |
| MTPT06 | MC1 | 100 | 589 | 0 | 0 | 63276 | 63864 | 107374 | 107962 | *psb*E | 0 | 1088 |
| MTPT07 | MC1 | 100 | 4675 | 0 | 0 | 96652 | 101326 | 111241 | 115915 | *trn*V-GAC, *trn*I-GAU | 0 | 8634 |
| MTPT08 | MC1 | 100 | 136 | 0 | 0 | 64811 | 64946 | 161308 | 161443 | *trn*W-CCA | 6.01E-66 | 252 |
| MTPT09 | MC1 | 97.895 | 190 | 3 | 1 | 65021 | 65209 | 161492 | 161681 |  | 9.71E-89 | 327 |
| MTPT10 | MC2 | 100 | 503 | 0 | 0 | 137043 | 137545 | 16316 | 16818 |  | 0 | 929 |
| MTPT11 | MC2 | 93.204 | 103 | 7 | 0 | 86883 | 86985 | 75653 | 75755 |  | 6.27E-36 | 152 |
| MTPT12 | MC2 | 100 | 13674 | 0 | 0 | 70219 | 83892 | 77995 | 91668 | *psb*B, *psb*H, *psb*N, *psb*T, *rpl*14, *rpl*2, *rpl*22, *rpo*A, *rps*11, *rps*19, *rps*8 | 0 | 25252 |
| MTPT13 | MC2 | 82.42 | 438 | 49 | 16 | 103738 | 104163 | 128770 | 129191 |  | 1.24E-97 | 357 |
| MTPT14 | MC2 | 91.045 | 134 | 2 | 3 | 131790 | 131922 | 131578 | 131702 |  | 4.82E-42 | 172 |
| MTPT15 | MC2 | 100 | 4133 | 0 | 0 | 51815 | 55947 | 155811 | 151679 | *atp*B fragment | 0 | 7633 |

Table S10 Summary of RNA editing sites detected in the PCGs of *S. splendens* mitogenome. “Nt Pos” and “AA Pos” means the position of the RNA editing sites in the nucleotide and amino acid sequences of the PCGs. Position: the sites of the codon in which RNA editing occurred. In the column of “Sp_So”, the 387 sites of *S. splendens* mitogenome that was homologous with *S. officinalis* were shown with “Yes”. In the column of “Sp_Ar”, the 201 sites of *S. splendens* mitogenome that was homologous with *Arabidopsis* were shown with “Yes”. In the column of “Stop_Gain”, the two stop-gain sites of *S. splendens* mitogenome were shown with “Yes”.

| Gene | Nt Pos | Sp_So | Sp_Ar | Stop_Gain | AA Pos | Reference Nt | Nt Coverage | Nt Base Count [A, C, G, T] | Nt Allsubs | Nt Frequency | Effect | Codon Position |
| --- | --- | --- | --- | --- | --- | --- | --- | --- | --- | --- | --- | --- |
| atp4 | 59 | Yes | No | No | 20 | C | 112 | [0, 0, 0, 112] | CT | 1 | TCT (S) => TTT (F) | 2 |
| atp4 | 71 | Yes | No | No | 24 | C | 127 | [0, 0, 0, 127] | CT | 1 | TCA (S) => TTA (L) | 2 |
| atp4 | 89* | Yes | Yes | No | 30 | C | 118 | [0, 1, 0, 117] | CT | 0.99 | TCA (S) => TTA (L) | 2 |
| atp4 | 118 | Yes | No | No | 40 | C | 130 | [0, 0, 0, 130] | CT | 1 | CGT (R) => TGT (C) | 1 |
| atp4 | 144 | No | No | No | 48 | C | 300 | [0, 118, 0, 182] | CT | 0.61 | TTC (F) => TTT (F) | 3 |
| atp4 | 215* | Yes | Yes | No | 72 | C | 481 | [0, 1, 0, 480] | CT | 1 | TCG (S) => TTG (L) | 2 |
| atp4 | 227 | Yes | No | No | 76 | C | 498 | [0, 135, 0, 363] | CT | 0.73 | CCC (P) => CTC (L) | 2 |
| atp4 | 248* | Yes | Yes | No | 83 | C | 438 | [0, 4, 0, 434] | CT | 0.99 | CCT (P) => CTT (L) | 2 |
| atp4 | 251* | Yes | Yes | No | 84 | C | 470 | [0, 5, 0, 465] | CT | 0.99 | CCG (P) => CTG (L) | 2 |
| atp4 | 395* | Yes | Yes | No | 132 | C | 94 | [0, 1, 0, 93] | CT | 0.99 | TCA (S) => TTA (L) | 2 |
| atp4 | 407 | Yes | No | No | 136 | C | 92 | [0, 0, 0, 92] | CT | 1 | CCA (P) => CTA (L) | 2 |
| atp4 | 416* | No | Yes | No | 139 | C | 76 | [0, 15, 0, 61] | CT | 0.8 | ACT (T) => ATT (I) | 2 |
| atp6 | 218 | Yes | No | No | 73 | C | 1132 | [0, 17, 0, 1115] | CT | 0.98 | CCG (P) => CTG (L) | 2 |
| atp6 | 287 | Yes | No | No | 96 | C | 918 | [0, 16, 0, 902] | CT | 0.98 | TCG (S) => TTG (L) | 2 |
| atp6 | 305 | Yes | No | No | 102 | C | 997 | [0, 16, 0, 981] | CT | 0.98 | TCG (S) => TTG (L) | 2 |
| atp6 | 313 | Yes | No | No | 105 | C | 795 | [0, 15, 0, 780] | CT | 0.98 | CGT (R) => TGT (C) | 1 |
| atp6 | 452 | Yes | No | No | 151 | C | 665 | [0, 14, 0, 651] | CT | 0.98 | TCA (S) => TTA (L) | 2 |
| atp6 | 511 | Yes | No | No | 171 | C | 530 | [0, 7, 0, 523] | CT | 0.99 | CCT (P) => TCT (S) | 1 |
| atp6 | 514 | Yes | No | No | 172 | C | 519 | [0, 5, 0, 514] | CT | 0.99 | CAT (H) => TAT (Y) | 1 |
| atp6 | 639 | No | No | No | 213 | C | 160 | [0, 142, 0, 18] | CT | 0.11 | TTC (F) => TTT (F) | 3 |
| atp8 | 30 | Yes | No | No | 10 | C | 2501 | [0, 1200, 0, 1301] | CT | 0.52 | TTC (F) => TTT (F) | 3 |
| atp8 | 58 | Yes | No | No | 20 | C | 2150 | [0, 107, 0, 2043] | CT | 0.95 | CTC (L) => TTC (F) | 1 |
| atp9 | 212 | Yes | No | No | 71 | C | 412 | [0, 8, 0, 404] | CT | 0.98 | TCA (S) => TTA (L) | 2 |
| ccmB | 28* | Yes | Yes | No | 10 | C | 291 | [0, 30, 0, 261] | CT | 0.9 | CAT (H) => TAT (Y) | 1 |
| ccmB | 45 | No | No | No | 15 | C | 265 | [0, 126, 0, 139] | CT | 0.52 | TCC (S) => TCT (S) | 3 |
| ccmB | 80 | Yes | No | No | 27 | C | 100 | [0, 31, 0, 69] | CT | 0.69 | TCG (S) => TTG (L) | 2 |
| ccmB | 87 | Yes | No | No | 29 | C | 102 | [0, 46, 0, 56] | CT | 0.55 | ATC (I) => ATT (I) | 3 |
| ccmB | 128 | Yes | No | No | 43 | C | 51 | [0, 36, 0, 15] | CT | 0.29 | TCA (S) => TTA (L) | 2 |
| ccmB | 137 | No | No | No | 46 | C | 37 | [0, 18, 0, 19] | CT | 0.51 | TCC (S) => TTC (F) | 2 |
| ccmB | 160 | No | No | No | 54 | C | 34 | [0, 30, 0, 4] | CT | 0.12 | CCT (P) => TCT (S) | 1 |
| ccmB | 172 | No | No | No | 58 | C | 26 | [0, 8, 0, 18] | CT | 0.69 | CCT (P) => TCT (S) | 1 |
| ccmB | 193 | No | No | No | 65 | C | 30 | [0, 13, 0, 17] | CT | 0.57 | CCT (P) => TCT (S) | 1 |
| ccmB | 194 | No | No | No | 65 | C | 32 | [0, 13, 0, 19] | CT | 0.59 | CCT (P) => TTT (S) | 2 |
| ccmB | 286* | Yes | Yes | No | 96 | C | 81 | [0, 28, 0, 53] | CT | 0.65 | CGG (R) => TGG (W) | 1 |
| ccmB | 304* | Yes | Yes | No | 102 | C | 60 | [0, 26, 0, 34] | CT | 0.57 | CGT (R) => TGT (C) | 1 |
| ccmB | 313 | Yes | No | No | 105 | C | 72 | [0, 9, 0, 63] | CT | 0.88 | CGT (R) => TGT (C) | 1 |
| ccmB | 338* | Yes | Yes | No | 113 | C | 72 | [0, 29, 0, 43] | CT | 0.6 | CCG (P) => CTG (L) | 2 |
| ccmB | 367 | Yes | No | No | 123 | C | 40 | [0, 15, 0, 25] | CT | 0.62 | CGG (R) => TGG (W) | 1 |
| ccmB | 380 | Yes | No | No | 127 | C | 30 | [0, 18, 0, 12] | CT | 0.4 | CCA (P) => CTA (L) | 2 |
| ccmB | 424* | Yes | Yes | No | 142 | C | 22 | [0, 11, 0, 11] | CT | 0.5 | CGT (R) => TGT (C) | 1 |
| ccmB | 428 | Yes | No | No | 143 | C | 22 | [0, 8, 0, 14] | CT | 0.64 | TCG (S) => TTG (L) | 2 |
| ccmB | 467 | No | No | No | 156 | C | 14 | [0, 9, 0, 5] | CT | 0.36 | TCG (S) => TTG (L) | 2 |
| ccmB | 512 | No | No | No | 171 | C | 6 | [0, 2, 0, 4] | CT | 0.67 | TCT (S) => TTT (F) | 2 |
| ccmB | 551* | Yes | Yes | No | 184 | C | 4 | [0, 2, 0, 2] | CT | 0.5 | TCA (S) => TTA (L) | 2 |
| ccmB | 554 | No | No | No | 185 | C | 4 | [0, 2, 0, 2] | CT | 0.5 | TCG (S) => TTG (L) | 2 |
| ccmB | 566 | No | No | No | 189 | C | 4 | [0, 0, 0, 4] | CT | 1 | TCC (S) => TTC (L) | 2 |
| ccmB | 572 | No | No | No | 191 | C | 4 | [0, 0, 0, 4] | CT | 1 | CCG (P) => CTG (L) | 2 |
| ccmB | 596 | No | No | No | 199 | C | 3 | [0, 0, 0, 3] | CT | 1 | TCG (S) => TTG (L) | 2 |
| ccmB | 611 | No | No | No | 204 | C | 2 | [0, 1, 0, 1] | CT | 0.5 | TCA (S) => TTA (L) | 2 |
| ccmC | 38 | Yes | No | No | 13 | C | 225 | [0, 54, 0, 171] | CT | 0.76 | TCA (S) => TTA (L) | 2 |
| ccmC | 76 | Yes | No | No | 26 | C | 193 | [0, 19, 0, 174] | CT | 0.9 | CGG (R) => TGG (W) | 1 |
| ccmC | 103* | Yes | Yes | No | 35 | C | 180 | [0, 23, 0, 157] | CT | 0.87 | CAT (H) => TAT (Y) | 1 |
| ccmC | 115 | Yes | No | No | 39 | C | 169 | [0, 13, 0, 156] | CT | 0.92 | CGG (R) => TGG (W) | 1 |
| ccmC | 133* | Yes | Yes | No | 45 | C | 129 | [0, 19, 0, 110] | CT | 0.85 | CTT (L) => TTT (F) | 1 |
| ccmC | 161 | Yes | No | No | 54 | C | 86 | [0, 13, 0, 73] | CT | 0.85 | CCC (P) => CTC (L) | 2 |
| ccmC | 179* | Yes | Yes | No | 60 | C | 74 | [0, 15, 0, 59] | CT | 0.8 | GCG (A) => GTG (V) | 2 |
| ccmC | 184* | Yes | Yes | No | 62 | C | 76 | [0, 17, 0, 59] | CT | 0.78 | CGG (R) => TGG (W) | 1 |
| ccmC | 281 | Yes | No | No | 94 | C | 48 | [0, 19, 0, 29] | CT | 0.6 | ACA (T) => ATA (I) | 2 |
| ccmC | 299 | Yes | No | No | 100 | C | 42 | [0, 21, 0, 21] | CT | 0.5 | TCT (S) => TTT (F) | 2 |
| ccmC | 331* | Yes | Yes | No | 111 | C | 26 | [0, 14, 0, 12] | CT | 0.46 | CGG (R) => TGG (W) | 1 |
| ccmC | 399 | Yes | No | No | 133 | C | 22 | [0, 14, 0, 8] | CT | 0.36 | TTC (F) => TTT (F) | 3 |
| ccmC | 400* | Yes | Yes | No | 134 | C | 21 | [0, 17, 0, 4] | CT | 0.19 | CTT (L) => TTT (F) | 1 |
| ccmC | 436 | Yes | No | No | 146 | C | 24 | [0, 16, 0, 8] | CT | 0.33 | CCT (P) => TCT (S) | 1 |
| ccmC | 446 | Yes | No | No | 149 | C | 23 | [0, 13, 0, 10] | CT | 0.43 | CCG (P) => CTG (L) | 2 |
| ccmC | 458* | Yes | Yes | No | 153 | C | 22 | [0, 16, 0, 6] | CT | 0.27 | TCA (S) => TTA (L) | 2 |
| ccmC | 497 | No | No | No | 166 | C | 24 | [0, 18, 0, 6] | CT | 0.25 | TCT (S) => TTT (F) | 2 |
| ccmC | 521 | No | No | No | 174 | C | 14 | [0, 7, 0, 7] | CT | 0.5 | TCG (S) => TTG (L) | 2 |
| ccmC | 575* | Yes | Yes | No | 192 | C | 5 | [0, 3, 0, 2] | CT | 0.4 | CCC (P) => CTC (L) | 2 |
| ccmC | 605 | Yes | No | No | 202 | C | 5 | [0, 1, 0, 4] | CT | 0.8 | TCC (S) => TTC (F) | 2 |
| ccmC | 608* | Yes | Yes | No | 203 | C | 5 | [0, 1, 0, 4] | CT | 0.8 | CCC (P) => CTC (L) | 2 |
| ccmC | 619 | No | No | No | 207 | C | 4 | [0, 0, 0, 4] | CT | 1 | CCT (P) => TCT (S) | 1 |
| ccmC | 656 | No | No | No | 219 | C | 3 | [0, 1, 0, 2] | CT | 0.67 | CCA (P) => CTA (L) | 2 |
| ccmFc | 38 | Yes | No | No | 13 | C | 41 | [0, 0, 0, 41] | CT | 1 | TCC (S) => TTC (F) | 2 |
| ccmFc | 39 | No | No | No | 13 | C | 34 | [0, 21, 0, 13] | CT | 0.38 | TCC (S) => TTT (F) | 3 |
| ccmFc | 50* | Yes | Yes | No | 17 | C | 33 | [0, 0, 0, 33] | CT | 1 | CCT (P) => CTT (L) | 2 |
| ccmFc | 52 | Yes | No | No | 18 | C | 33 | [0, 1, 0, 32] | CT | 0.97 | CGT (R) => TGT (C) | 1 |
| ccmFc | 103* | Yes | Yes | No | 35 | C | 43 | [0, 2, 0, 41] | CT | 0.95 | CCC (P) => TCC (S) | 1 |
| ccmFc | 122* | Yes | Yes | No | 41 | C | 50 | [0, 1, 0, 49] | CT | 0.98 | TCC (S) => TTC (F) | 2 |
| ccmFc | 146* | Yes | Yes | No | 49 | C | 60 | [0, 12, 0, 48] | CT | 0.8 | CCT (P) => CTT (L) | 2 |
| ccmFc | 151 | No | No | No | 51 | C | 61 | [0, 3, 0, 58] | CT | 0.95 | CCT (P) => TCT (S) | 1 |
| ccmFc | 155* | Yes | Yes | No | 52 | C | 59 | [0, 4, 0, 55] | CT | 0.93 | TCA (S) => TTA (L) | 2 |
| ccmFc | 310 | Yes | No | No | 104 | C | 51 | [0, 3, 0, 48] | CT | 0.94 | CGT (R) => TGT (C) | 1 |
| ccmFc | 334* | Yes | Yes | No | 112 | C | 46 | [0, 6, 0, 40] | CT | 0.87 | CTT (L) => TTT (F) | 1 |
| ccmFc | 390 | No | No | No | 130 | C | 53 | [0, 45, 0, 8] | CT | 0.15 | CTC (L) => CTT (L) | 3 |
| ccmFc | 406* | Yes | Yes | No | 136 | C | 57 | [0, 2, 0, 55] | CT | 0.96 | CGT (R) => TGT (C) | 1 |
| ccmFc | 700 | No | No | No | 234 | C | 2 | [0, 0, 0, 2] | CT | 1 | CCA (P) => TCA (S) | 1 |
| ccmFc | 701 | No | No | No | 234 | C | 2 | [0, 0, 0, 2] | CT | 1 | CCA (P) => TTA (S) | 2 |
| ccmFc | 955 | No | No | No | 319 | C | 166 | [0, 138, 0, 28] | CT | 0.17 | CGT (R) => TGT (C) | 1 |
| ccmFc | 1211 | No | No | No | 404 | C | 15 | [0, 13, 0, 2] | CT | 0.13 | CCT (P) => CTT (L) | 2 |
| ccmFc | 1228* | Yes | Yes | No | 410 | C | 15 | [0, 0, 0, 15] | CT | 1 | CGG (R) => TGG (W) | 1 |
| ccmFc | 1233 | Yes | No | No | 411 | C | 13 | [0, 11, 0, 2] | CT | 0.15 | ATC (I) => ATT (I) | 3 |
| ccmFn | 38 | Yes | No | No | 13 | C | 493 | [0, 7, 0, 486] | CT | 0.99 | CCG (P) => CTG (L) | 2 |
| ccmFn | 98 | Yes | No | No | 33 | C | 397 | [0, 30, 0, 367] | CT | 0.92 | CCT (P) => CTT (L) | 1 |
| ccmFn | 137 | Yes | No | No | 46 | C | 208 | [0, 73, 0, 135] | CT | 0.65 | TCG (S) => TTG (L) | 2 |
| ccmFn | 142 | Yes | No | No | 48 | C | 231 | [0, 5, 0, 226] | CT | 0.98 | CGT (R) => TGT (C) | 1 |
| ccmFn | 151 | Yes | No | No | 51 | C | 209 | [0, 10, 0, 199] | CT | 0.95 | CCT (P) => TCT (S) | 1 |
| ccmFn | 165 | Yes | No | No | 55 | C | 215 | [0, 160, 0, 55] | CT | 0.26 | TCC (S) => TCT (S) | 3 |
| ccmFn | 248 | Yes | No | No | 83 | C | 321 | [0, 9, 0, 312] | CT | 0.97 | TCA (S) => TTA (L) | 2 |
| ccmFn | 256 | Yes | No | No | 86 | C | 370 | [0, 19, 0, 351] | CT | 0.95 | CGG (R) => TGG (W) | 1 |
| ccmFn | 283 | Yes | No | No | 95 | C | 343 | [0, 27, 0, 316] | CT | 0.92 | CTT (L) => TTT (F) | 1 |
| ccmFn | 371* | Yes | Yes | No | 124 | C | 299 | [0, 33, 0, 266] | CT | 0.89 | TCG (S) => TTG (L) | 2 |
| ccmFn | 378* | Yes | Yes | No | 126 | C | 288 | [0, 47, 0, 241] | CT | 0.84 | TTC (F) => TTT (F) | 3 |
| ccmFn | 406 | No | No | No | 136 | C | 308 | [0, 273, 0, 35] | CT | 0.11 | CGT (R) => TGT (C) | 1 |
| ccmFn | 713* | Yes | Yes | No | 238 | C | 217 | [0, 7, 0, 210] | CT | 0.97 | CCT (P) => CTT (L) | 2 |
| ccmFn | 722 | Yes | No | No | 241 | C | 213 | [0, 112, 0, 101] | CT | 0.47 | TCA (S) => TTA (L) | 2 |
| ccmFn | 732 | Yes | No | No | 244 | C | 205 | [0, 159, 0, 46] | CT | 0.22 | TCC (S) => TCT (S) | 3 |
| ccmFn | 760 | Yes | No | No | 254 | C | 252 | [0, 18, 0, 234] | CT | 0.93 | CGT (R) => TGT (C) | 1 |
| ccmFn | 782* | Yes | Yes | No | 261 | C | 288 | [0, 20, 0, 268] | CT | 0.93 | TCA (S) => TTA (L) | 2 |
| ccmFn | 794* | Yes | Yes | No | 265 | C | 316 | [0, 26, 0, 290] | CT | 0.92 | CCA (P) => CTA (L) | 2 |
| ccmFn | 809* | Yes | Yes | No | 279 | C | 305 | [0, 27, 0, 278] | CT | 0.91 | TCA (S) => TTA (L) | 2 |
| ccmFn | 958* | Yes | Yes | No | 320 | C | 407 | [0, 3, 0, 404] | CT | 0.99 | CGC (R) => TGC (C) | 1 |
| ccmFn | 1008 | No | No | No | 336 | C | 375 | [0, 290, 0, 85] | CT | 0.23 | CTC (L) => CTT (L) | 3 |
| ccmFn | 1276 | Yes | No | No | 426 | C | 285 | [0, 20, 0, 265] | CT | 0.93 | CGG (R) => TGG (W) | 1 |
| ccmFn | 1304 | Yes | No | No | 435 | C | 314 | [0, 25, 0, 289] | CT | 0.92 | CCA (P) => CTA (L) | 2 |
| ccmFn | 1321 | Yes | No | No | 441 | C | 322 | [0, 12, 0, 310] | CT | 0.96 | CAT (H) => TAT (Y) | 1 |
| ccmFn | 1336 | Yes | No | No | 446 | C | 312 | [0, 19, 0, 293] | CT | 0.94 | CGG (R) => TGG (W) | 1 |
| ccmFn | 1354 | Yes | No | No | 452 | C | 401 | [0, 16, 0, 385] | CT | 0.96 | CGG (R) => TGG (W) | 1 |
| ccmFn | 1387 | Yes | No | No | 463 | C | 477 | [0, 52, 0, 425] | CT | 0.89 | CGG (R) => TGG (W) | 1 |
| ccmFn | 1429 | Yes | No | No | 477 | C | 629 | [0, 549, 0, 80] | CT | 0.13 | CTT (L) => TTT (F) | 1 |
| ccmFn | 1448 | Yes | No | No | 483 | C | 644 | [0, 13, 0, 631] | CT | 0.98 | TCC (S) => TTC (F) | 2 |
| ccmFn | 1472 | Yes | No | No | 491 | C | 715 | [0, 17, 0, 698] | CT | 0.98 | CCA (P) => CTA (L) | 2 |
| ccmFn | 1484 | Yes | No | No | 495 | C | 690 | [0, 120, 0, 570] | CT | 0.83 | TCA (S) => TTA (L) | 2 |
| ccmFn | 1519 | Yes | No | No | 507 | C | 503 | [0, 10, 0, 493] | CT | 0.98 | CCC (P) => TCC (S) | 1 |
| ccmFn | 1747 | No | No | No | 583 | C | 22 | [0, 3, 0, 19] | CT | 0.86 | CCT (P) => TCT (S) | 1 |
| cob | 298 | Yes | No | No | 100 | C | 2186 | [0, 68, 0, 2118] | CT | 0.97 | CAC (H) => TAC (Y) | 1 |
| cob | 325* | Yes | Yes | No | 109 | C | 1963 | [0, 16, 0, 1947] | CT | 0.99 | CAT (H) => TAT (Y) | 1 |
| cob | 358 | Yes | No | No | 120 | C | 1989 | [0, 13, 0, 1976] | CT | 0.99 | CGG (R) => TGG (W) | 1 |
| cob | 568* | Yes | Yes | No | 190 | C | 2001 | [0, 14, 0, 1987] | CT | 0.99 | CAT (H) => TAT (Y) | 1 |
| cob | 853* | Yes | Yes | No | 285 | C | 2908 | [0, 19, 0, 2889] | CT | 0.99 | CAT (H) => TAT (Y) | 1 |
| cob | 908* | Yes | Yes | No | 303 | C | 2325 | [0, 64, 0, 2261] | CT | 0.97 | TCC (S) => TTC (F) | 2 |
| cob | 982* | Yes | Yes | No | 328 | C | 1328 | [0, 11, 0, 1317] | CT | 0.99 | CAC (H) => TAC (Y) | 1 |
| cob | 1015 | Yes | No | No | 339 | C | 1024 | [0, 9, 0, 1015] | CT | 0.99 | CGC (R) => TGC (C) | 1 |
| cob | 1084* | Yes | Yes | No | 362 | C | 478 | [0, 8, 0, 470] | CT | 0.98 | CCT (P) =>TCT (S) | 1 |
| cob | 1101 | No | No | No | 367 | C | 323 | [0, 253, 0, 70] | CT | 0.22 | TTC (F) => TTT (F) | 3 |
| cob | 1160* | Yes | Yes | No | 387 | C | 109 | [0, 97, 0, 12] | CT | 0.11 | ACG (P) =>ATG (M) | 2 |
| cox1 | 242 | Yes | No | No | 81 | C | 1453 | [0, 13, 1, 1439] | CT | 0.99 | TCT (S) => TTT (F) | 2 |
| cox1 | 254* | Yes | Yes | No | 85 | C | 1711 | [0, 23, 0, 1688] | CT | 0.99 | TCT (S) => TTT (F) | 2 |
| cox1 | 265 | No | No | No | 89 | C | 1729 | [0, 1416, 0, 313] | CT | 0.18 | CTG (L) => TTG (L) | 1 |
| cox1 | 452* | Yes | Yes | No | 151 | C | 619 | [0, 7, 0, 612] | CT | 0.99 | TCT (S) => TTT (F) | 2 |
| cox1 | 515 | Yes | No | No | 172 | C | 335 | [0, 41, 0, 294] | CT | 0.88 | TCC (S) => TTC (F) | 2 |
| cox1 | 551 | Yes | No | No | 184 | C | 197 | [0, 2, 0, 195] | CT | 0.99 | TCA (S) => TTA (L) | 2 |
| cox1 | 590 | Yes | No | No | 197 | C | 115 | [0, 2, 0, 113] | CT | 0.98 | CCA (P) => CTA (L) | 2 |
| cox1 | 715 | Yes | No | No | 239 | C | 5 | [0, 0, 0, 5] | CT | 1 | CGG (R) => TGG (W) | 1 |
| cox1 | 761 | Yes | No | No | 254 | C | 503 | [0, 10, 0, 493] | CT | 0.98 | TCC (S) => TTC (F) | 2 |
| cox1 | 1078 | Yes | No | No | 360 | C | 1468 | [0, 1060, 0, 408] | CT | 0.28 | CTG (L) => TTG (L) | 1 |
| cox1 | 1186 | Yes | No | No | 396 | C | 912 | [0, 56, 0, 856] | CT | 0.94 | CAC (H) => TAC (Y) | 1 |
| cox1 | 1296 | No | No | No | 432 | C | 2004 | [0, 1327, 0, 677] | CT | 0.34 | TTC (F) => TTT (F) | 3 |
| cox1 | 1405 | Yes | No | No | 469 | C | 1049 | [0, 33, 0, 1016] | CT | 0.97 | CGT (R) => TGT (C) | 1 |
| cox1 | 1433 | Yes | No | No | 478 | C | 783 | [0, 16, 0, 767] | CT | 0.98 | TCA (S) => TTA (L) | 2 |
| cox1 | 1489 | Yes | No | No | 497 | C | 331 | [0, 8, 0, 323] | CT | 0.98 | CCA (P) => TCA (S) | 1 |
| cox1 | 1499 | Yes | No | No | 500 | C | 302 | [0, 10, 0, 292] | CT | 0.97 | CCA (P) => CTA (L) | 2 |
| cox2 | 32 | Yes | No | No | 11 | C | 6771 | [0, 185, 0, 6586] | CT | 0.97 | TCT (S) => TTT (F) | 2 |
| cox2 | 404 | Yes | No | No | 135 | C | 3919 | [0, 108, 0, 3811] | CT | 0.97 | ACG (P) =>ATG (M) | 2 |
| cox2 | 422 | Yes | No | No | 141 | C | 3393 | [0, 103, 0, 3290] | CT | 0.97 | CCA (P) => CTA (L) | 2 |
| cox2 | 437 | Yes | No | No | 146 | C | 2949 | [0, 119, 0, 2830] | CT | 0.96 | TCA (S) => TTA (L) | 2 |
| cox2 | 505 | Yes | No | No | 169 | C | 788 | [0, 38, 0, 750] | CT | 0.95 | CCT (P) => TCT (S) | 1 |
| cox2 | 518 | Yes | No | No | 173 | C | 550 | [0, 46, 0, 504] | CT | 0.92 | CCT (P) => CTT (L) | 2 |
| cox2 | 580 | Yes | No | No | 194 | C | 7 | [5, 2, 0, 0] | CA | 0.71 | CAG (Q) => AAG (K) | 1 |
| cox2 | 584 | No | No | No | 195 | C | 11 | [2, 9, 0, 0] | CA | 0.18 | ACC (T) => AAC (N) | 2 |
| cox2 | 586 | No | No | No | 196 | T | 79 | [22, 0, 2, 55] | TA | 0.28 | TCT (S) => ACT (T) | 1 |
| cox2 | 593 | Yes | No | No | 198 | C | 37 | [0, 0, 0, 37] | CT | 1 | TCG (S) => TTT (F) | 2 |
| cox2 | 594 | No | No | No | 198 | G | 4 | [0, 0, 3, 1] | GT | 0.25 | TCG (S) => TTT (F) | 3 |
| cox2 | 598 | No | No | No | 200 | C | 5 | [2, 3, 0, 0] | CA | 0.4 | CAA (Q) => AAA (K) | 1 |
| cox2 | 615 | No | No | Yes | 205 | C | 2 | [1, 1, 0, 0] | CA | 0.5 | TAC (Y) => TAA (*) | 3 |
| cox2 | 682* | Yes | Yes | No | 228 | C | 78 | [0, 2, 0, 76] | CT | 0.97 | CCT (P) => TCT (S) | 1 |
| cox2 | 703* | Yes | Yes | No | 235 | C | 55 | [0, 1, 0, 54] | CT | 0.98 | CGG (R) => TGG (W) | 1 |
| cox3 | 174 | Yes | No | No | 58 | C | 3502 | [0, 3100, 0, 402] | CT | 0.11 | TCT (S) => TTT (F) | 2 |
| cox3 | 245* | Yes | Yes | No | 82 | C | 2892 | [0, 41, 0, 2851] | CT | 0.99 | CCT (P) => CTT (L) | 2 |
| cox3 | 304 | Yes | No | No | 102 | C | 2063 | [0, 48, 1, 2014] | CT | 0.98 | CGG (R) => TGG (W) | 1 |
| cox3 | 311* | Yes | Yes | No | 104 | C | 2014 | [0, 117, 0, 1897] | CT | 0.94 | TCT (S) => TTT (F) | 2 |
| cox3 | 314* | Yes | Yes | No | 105 | C | 1986 | [0, 71, 1, 1914] | CT | 0.96 | TCT (S) => TTT (F) | 2 |
| cox3 | 419 | Yes | No | No | 140 | C | 4026 | [0, 25, 0, 4001] | CT | 0.99 | CCC (P) => CTC (L) | 2 |
| cox3 | 422* | Yes | Yes | No | 141 | C | 3821 | [0, 91, 0, 3730] | CT | 0.98 | CCT (P) => CTT (L) | 2 |
| cox3 | 566 | Yes | No | No | 189 | C | 5154 | [0, 94, 0, 5060] | CT | 0.98 | TCC (S) => TTC (F) | 2 |
| cox3 | 567 | Yes | No | No | 189 | C | 5313 | [0, 4200, 0, 1113] | CT | 0.21 | TCC (S) => TTT (F) | 3 |
| cox3 | 651 | No | No | No | 217 | C | 1366 | [0, 1052, 0, 314] | CT | 0.23 | TTC (F) => TTT (F) | 3 |
| cox3 | 754 | Yes | No | No | 252 | C | 298 | [0, 6, 0, 292] | CT | 0.98 | CGG (R) => TGG (W) | 1 |
| cox3 | 764 | Yes | No | No | 255 | C | 230 | [0, 14, 0, 216] | CT | 0.94 | CCA (P) => CTA (L) | 2 |
| matR | 32 | Yes | No | No | 11 | C | 934 | [0, 93, 0, 841] | CT | 0.9 | TCC (S) => TTC (F) | 2 |
| matR | 43 | Yes | No | No | 15 | C | 907 | [0, 83, 0, 824] | CT | 0.91 | TCC (S) => TTC (F) | 2 |
| matR | 166 | No | No | No | 56 | C | 393 | [0, 313, 0, 80] | CT | 0.2 | CAC (H) => TAC (Y) | 1 |
| matR | 237 | Yes | No | No | 79 | C | 282 | [0, 189, 0, 93] | CT | 0.33 | TCC (S) => TCT (S) | 3 |
| matR | 326* | Yes | Yes | No | 109 | C | 332 | [0, 19, 0, 313] | CT | 0.94 | CCA (P) => CTA (L) | 2 |
| matR | 413* | Yes | Yes | No | 138 | C | 307 | [0, 16, 0, 291] | CT | 0.95 | TCG (S) => TTG (L) | 2 |
| matR | 474 | No | No | No | 158 | C | 283 | [0, 229, 0, 54] | CT | 0.19 | TTC (F) => TTT (F) | 3 |
| matR | 482 | No | No | No | 161 | C | 269 | [0, 165, 0, 104] | CT | 0.39 | CCC (P) => CTC (L) | 2 |
| matR | 1215 | No | No | No | 405 | C | 474 | [0, 367, 0, 107] | CT | 0.23 | ATC (I) => ATT (I) | 3 |
| matR | 1531 | Yes | No | No | 511 | C | 989 | [0, 708, 0, 281] | CT | 0.28 | CCC (P) => TCC (S) | 1 |
| matR | 1533 | Yes | No | No | 511 | C | 997 | [0, 731, 0, 266] | CT | 0.27 | CCC (P) => TCT (S) | 3 |
| matR | 1667* | Yes | Yes | No | 556 | C | 1045 | [0, 51, 0, 994] | CT | 0.95 | TCC (S) => TTC (F) | 2 |
| matR | 1688* | Yes | Yes | No | 563 | C | 919 | [0, 93, 0, 826] | CT | 0.9 | CCT (P) => CTT (L) | 2 |
| matR | 1708* | Yes | Yes | No | 570 | C | 760 | [0, 56, 0, 704] | CT | 0.93 | CGC (R) => TGC (C) | 1 |
| matR | 1722 | Yes | No | No | 574 | C | 822 | [0, 594, 0, 228] | CT | 0.28 | TAC (Y) => TAT (Y) | 3 |
| matR | 1744* | Yes | Yes | No | 582 | C | 705 | [0, 49, 0, 656] | CT | 0.93 | CAC (H) => TAC (Y) | 1 |
| matR | 1775 | Yes | No | No | 592 | C | 562 | [0, 98, 0, 464] | CT | 0.83 | CCG (P) => CTG (L) | 2 |
| matR | 1814 | Yes | No | No | 605 | C | 482 | [0, 29, 0, 453] | CT | 0.94 | CCA (P) => CTA (L) | 2 |
| matR | 1832* | Yes | Yes | No | 611 | C | 365 | [0, 57, 0, 308] | CT | 0.84 | TCA (S) => TTA (L) | 2 |
| mttB | 16 | Yes | No | No | 6 | C | 241 | [0, 38, 0, 203] | CT | 0.84 | CAT (H) => TAT (Y) | 1 |
| mttB | 25 | No | No | No | 9 | C | 228 | [0, 195, 0, 33] | CT | 0.14 | CCG (P) => TCG (S) | 1 |
| mttB | 26* | Yes | Yes | No | 9 | C | 249 | [0, 58, 0, 191] | CT | 0.77 | CCG (P) =>TTG (L) | 2 |
| mttB | 64* | Yes | Yes | No | 22 | C | 226 | [0, 75, 0, 151] | CT | 0.67 | CGG (R) => TGG (W) | 1 |
| mttB | 100 | Yes | No | No | 34 | C | 149 | [0, 128, 0, 21] | CT | 0.14 | CGT (R) => TGT (C) | 1 |
| mttB | 112* | Yes | Yes | No | 38 | C | 118 | [0, 81, 0, 37] | CT | 0.31 | CCG (P) => TCG (S) | 1 |
| mttB | 128* | Yes | Yes | No | 43 | C | 114 | [0, 33, 0, 81] | CT | 0.71 | TCT (S) => TTT (F) | 2 |
| mttB | 178 | Yes | No | No | 60 | C | 119 | [0, 55, 0, 64] | CT | 0.54 | CGT (R) => TGT (C) | 1 |
| mttB | 188 | Yes | No | No | 63 | C | 113 | [0, 8, 0, 105] | CT | 0.93 | TCA (S) => TTA (L) | 2 |
| mttB | 202 | Yes | No | No | 68 | C | 112 | [0, 33, 0, 79] | CT | 0.71 | CCG (P) => TCG (S) | 1 |
| mttB | 236 | Yes | No | No | 79 | C | 118 | [0, 80, 0, 38] | CT | 0.32 | TCT (S) => TTT (F) | 2 |
| mttB | 262 | Yes | No | No | 88 | C | 111 | [0, 18, 0, 93] | CT | 0.84 | CAT (H) => TAT (Y) | 1 |
| mttB | 328* | Yes | Yes | No | 110 | C | 76 | [0, 32, 0, 44] | CT | 0.58 | CTC (L) => TTC (F) | 1 |
| mttB | 331* | Yes | Yes | No | 111 | C | 79 | [0, 29, 0, 50] | CT | 0.63 | CAT (H) => TAT (Y) | 1 |
| mttB | 344 | Yes | No | No | 115 | C | 77 | [0, 47, 0, 30] | CT | 0.39 | TCT (S) => TTT (F) | 2 |
| mttB | 346* | Yes | Yes | No | 116 | C | 79 | [0, 55, 0, 24] | CT | 0.3 | CGC (R) => TGC (C) | 1 |
| mttB | 373 | Yes | No | No | 125 | C | 68 | [0, 22, 0, 46] | CT | 0.68 | CTT (L) => TTT (F) | 1 |
| mttB | 376* | Yes | Yes | No | 126 | C | 69 | [0, 54, 0, 15] | CT | 0.22 | CCC (P) => TCC (S) | 1 |
| mttB | 379 | Yes | No | No | 127 | C | 83 | [0, 74, 0, 9] | CT | 0.11 | CGG (R) => TGG (W) | 1 |
| mttB | 407* | Yes | Yes | No | 136 | C | 158 | [0, 49, 0, 109] | CT | 0.69 | CCA (P) => CTA (L) | 2 |
| mttB | 472* | Yes | Yes | No | 158 | C | 203 | [0, 153, 0, 50] | CT | 0.25 | CAT (H) => TAT (Y) | 1 |
| mttB | 497* | Yes | Yes | No | 166 | C | 212 | [0, 47, 0, 165] | CT | 0.78 | TCG (S) => TTG (L) | 2 |
| mttB | 505* | Yes | Yes | No | 169 | C | 211 | [0, 23, 0, 188] | CT | 0.89 | CCA (P) => TCA (S) | 1 |
| mttB | 541 | Yes | No | No | 181 | C | 149 | [0, 30, 0, 119] | CT | 0.8 | CGT (R) => TGT (C) | 1 |
| mttB | 554* | Yes | Yes | No | 185 | C | 117 | [0, 63, 0, 54] | CT | 0.46 | CCA (P) => CTA (L) | 2 |
| mttB | 578 | Yes | No | No | 193 | C | 81 | [0, 18, 0, 63] | CT | 0.78 | TCC (S) => TTC (F) | 2 |
| mttB | 610* | Yes | Yes | No | 204 | C | 9 | [1, 2, 0, 6] | CT | 0.75 | CCG (P) => TCG (S) | 1 |
| mttB | 616* | Yes | Yes | No | 206 | C | 31 | [0, 1, 0, 30] | CT | 0.97 | CTC (L) => TTC (F) | 1 |
| mttB | 667* | Yes | Yes | No | 223 | C | 28 | [0, 6, 0, 22] | CT | 0.79 | CGT (R) => TGT (C) | 1 |
| mttB | 672* | Yes | Yes | No | 224 | C | 28 | [0, 24, 0, 4] | CT | 0.14 | TTC (F) => TTT (F) | 3 |
| mttB | 713* | Yes | Yes | No | 238 | C | 28 | [0, 9, 0, 19] | CT | 0.68 | TCG (S) => TTG (L) | 2 |
| nad1 | 215 | Yes | No | No | 72 | C | 294 | [0, 9, 0, 285] | CT | 0.97 | TCC (S) => TTC (F) | 2 |
| nad1 | 265* | Yes | Yes | No | 89 | C | 152 | [0, 9, 0, 143] | CT | 0.94 | CGG (R) => TGG (W) | 1 |
| nad1 | 307* | Yes | Yes | No | 103 | C | 36 | [0, 23, 0, 13] | CT | 0.36 | CCG (P) => TTG (S) | 1 |
| nad1 | 308* | Yes | Yes | No | 103 | C | 62 | [0, 21, 0, 41] | CT | 0.66 | CCG (P) => TTG (L) | 2 |
| nad1 | 376* | Yes | Yes | No | 126 | C | 14 | [0, 1, 0, 13] | CT | 0.93 | CGG (R) => TGG (W) | 1 |
| nad1 | 401 | Yes | No | No | 134 | C | 189 | [0, 65, 0, 124] | CT | 0.66 | TCT (S) => TTT (F) | 2 |
| nad1 | 436 | Yes | No | No | 146 | C | 154 | [0, 6, 0, 148] | CT | 0.96 | CCT (P) => TCT (S) | 1 |
| nad1 | 490* | Yes | Yes | No | 164 | C | 212 | [0, 46, 0, 166] | CT | 0.78 | CCC (P) => TCT (S) | 1 |
| nad1 | 493* | Yes | Yes | No | 164 | C | 218 | [0, 145, 0, 73] | CT | 0.33 | CCC (P) => TCT (S) | 3 |
| nad1 | 500 | Yes | No | No | 167 | C | 231 | [0, 147, 0, 84] | CT | 0.36 | TCG (S) => TTG (L) | 2 |
| nad1 | 536* | Yes | Yes | No | 179 | C | 232 | [0, 4, 0, 228] | CT | 0.98 | TCC (S) => TTC (F) | 2 |
| nad1 | 635* | Yes | Yes | No | 212 | C | 115 | [0, 3, 0, 112] | CT | 0.97 | TCA (S) => TTA (L) | 2 |
| nad1 | 725* | Yes | Yes | No | 242 | C | 337 | [0, 41, 0, 296] | CT | 0.88 | CCA (P) => CTA (L) | 2 |
| nad1 | 734 | Yes | No | No | 245 | C | 360 | [0, 65, 0, 295] | CT | 0.82 | TCG (S) => TTG (L) | 2 |
| nad1 | 740 | Yes | No | No | 247 | C | 356 | [0, 47, 0, 309] | CT | 0.87 | TCT (S) => TTT (F) | 2 |
| nad1 | 743* | Yes | Yes | No | 248 | C | 363 | [0, 54, 0, 309] | CT | 0.85 | CCA (P) => CTA (L) | 2 |
| nad1 | 755* | Yes | Yes | No | 252 | C | 609 | [0, 70, 0, 539] | CT | 0.89 | CCG (P) => CTG (L) | 2 |
| nad1 | 779 | Yes | No | No | 260 | C | 693 | [0, 63, 0, 630] | CT | 0.91 | TCC (S) => TTC (F) | 2 |
| nad1 | 792 | Yes | No | No | 264 | C | 874 | [0, 612, 0, 262] | CT | 0.3 | CCC (P) => CCT (P) | 3 |
| nad1 | 823* | Yes | Yes | No | 275 | C | 1021 | [0, 52, 0, 969] | CT | 0.95 | CTC (L) => TTC (F) | 1 |
| nad1 | 898* | Yes | Yes | No | 300 | C | 1037 | [0, 2, 0, 1035] | CT | 1 | CGG (R) => TGG (W) | 1 |
| nad1 | 909 | Yes | No | No | 303 | C | 852 | [0, 703, 0, 149] | CT | 0.17 | TTC (F) => TTT (F) | 3 |
| nad1 | 928* | Yes | Yes | No | 310 | C | 627 | [0, 8, 0, 619] | CT | 0.99 | CGG (R) => TGG (W) | 1 |
| nad2 | 26* | Yes | Yes | No | 9 | C | 120 | [0, 44, 0, 76] | CT | 0.63 | TCC (S) => TTC (F) | 2 |
| nad2 | 89 | No | No | No | 30 | C | 50 | [19, 31, 0, 0] | CA | 0.38 | ACT (T) => ATT (I) | 2 |
| nad2 | 92 | No | No | No | 31 | C | 4 | [1, 3, 0, 0] | CA | 0.25 | TCT (S) => TTT (F) | 2 |
| nad2 | 109 | No | No | No | 37 | C | 4 | [1, 3, 0, 0] | CA | 0.25 | CGG (R) => TGG (W) | 1 |
| nad2 | 223 | Yes | No | No | 75 | C | 532 | [0, 22, 0, 510] | CT | 0.96 | CTT (L) => TTT (F) | 1 |
| nad2 | 252 | Yes | No | No | 84 | C | 522 | [0, 390, 0, 132] | CT | 0.25 | TTC (F) => TTT (F) | 3 |
| nad2 | 303 | Yes | No | No | 101 | C | 323 | [0, 270, 0, 53] | CT | 0.16 | TTC (F) => TTT (F) | 3 |
| nad2 | 308* | Yes | Yes | No | 103 | C | 299 | [0, 31, 0, 268] | CT | 0.9 | TCT (S) => TTT (F) | 2 |
| nad2 | 311* | Yes | Yes | No | 104 | C | 316 | [0, 31, 0, 285] | CT | 0.9 | TCC (S) => TTC (F) | 2 |
| nad2 | 356* | Yes | Yes | No | 119 | C | 155 | [0, 59, 0, 96] | CT | 0.62 | CCA (P) => CTA (L) | 2 |
| nad2 | 361* | Yes | Yes | No | 121 | C | 154 | [0, 25, 0, 129] | CT | 0.84 | CCT (P) => TCT (S) | 1 |
| nad2 | 367* | Yes | Yes | No | 123 | C | 150 | [0, 75, 0, 75] | CT | 0.5 | CGC (R) => TGC (C) | 1 |
| nad2 | 401 | Yes | No | No | 134 | C | 95 | [0, 18, 0, 77] | CT | 0.81 | TCA (S) => TTA (L) | 2 |
| nad2 | 428* | Yes | Yes | No | 143 | C | 60 | [0, 9, 0, 51] | CT | 0.85 | CCT (P) => CTT (L) | 2 |
| nad2 | 497* | Yes | Yes | No | 166 | C | 19 | [0, 4, 0, 15] | CT | 0.79 | TCG (S) => TTG (L) | 2 |
| nad2 | 741 | No | No | No | 247 | C | 783 | [0, 685, 0, 98] | CT | 0.13 | CCC (P) => CCT (P) | 3 |
| nad2 | 788* | Yes | Yes | No | 263 | C | 1333 | [0, 38, 0, 1295] | CT | 0.97 | TCT (S) => TTT (F) | 2 |
| nad2 | 800 | Yes | No | No | 267 | C | 1457 | [0, 55, 0, 1402] | CT | 0.96 | TCA (S) => TTA (L) | 2 |
| nad2 | 809* | Yes | Yes | No | 270 | C | 1486 | [0, 237, 0, 1249] | CT | 0.84 | TCT (S) => TTT (F) | 2 |
| nad2 | 928* | Yes | Yes | No | 310 | C | 1148 | [0, 67, 0, 1081] | CT | 0.94 | CAT (H) => TAT (Y) | 1 |
| nad2 | 958* | Yes | Yes | No | 320 | C | 1013 | [0, 95, 0, 918] | CT | 0.91 | CGT (R) => TGT (C) | 1 |
| nad2 | 1028 | Yes | No | No | 343 | C | 656 | [0, 26, 0, 630] | CT | 0.96 | TCA (S) => TTA (L) | 2 |
| nad2 | 1058* | Yes | Yes | No | 353 | C | 530 | [0, 64, 0, 466] | CT | 0.88 | TCA (S) => TTA (L) | 2 |
| nad2 | 1276 | No | No | No | 426 | C | 30 | [0, 0, 0, 30] | CT | 1 | CGT (R) => TGT (C) | 1 |
| nad2 | 1298 | Yes | No | No | 433 | C | 267 | [0, 16, 0, 251] | CT | 0.94 | GCG (A) => GTG (V) | 2 |
| nad2 | 1400* | Yes | Yes | No | 467 | C | 181 | [0, 5, 0, 176] | CT | 0.97 | TCA (S) => TTA (L) | 2 |
| nad2 | 1408 | Yes | No | No | 470 | C | 148 | [0, 11, 0, 137] | CT | 0.93 | CCA (P) => TTA (S) | 1 |
| nad2 | 1409 | Yes | No | No | 470 | C | 154 | [0, 10, 0, 144] | CT | 0.94 | CCA (P) => TTA (L) | 2 |
| nad2 | 1416 | Yes | No | No | 472 | C | 140 | [0, 11, 0, 129] | CT | 0.92 | CCC (P) => CCT (P) | 3 |
| nad2 | 1457* | No | Yes | No | 486 | C | 36 | [0, 0, 0, 36] | CT | 1 | TCA (S) => TTA (L) | 2 |
| nad3 | 44 | Yes | No | No | 15 | C | 153 | [0, 20, 0, 133] | CT | 0.87 | TCG (S) => TTG (L) | 2 |
| nad3 | 62 | Yes | No | No | 21 | C | 144 | [0, 46, 0, 98] | CT | 0.68 | CCA (P) => CTA (L) | 2 |
| nad3 | 79 | Yes | No | No | 27 | C | 139 | [0, 10, 0, 129] | CT | 0.93 | CCA (P) => TTA (S) | 1 |
| nad3 | 80* | Yes | Yes | No | 27 | C | 133 | [0, 5, 0, 128] | CT | 0.96 | CCA (P) => TTA (L) | 2 |
| nad3 | 124 | Yes | No | No | 42 | C | 90 | [0, 5, 0, 85] | CT | 0.94 | CAC (H) => TAC (Y) | 1 |
| nad3 | 146* | Yes | Yes | No | 49 | C | 70 | [0, 8, 0, 62] | CT | 0.89 | TCC (S) => TTC (F) | 2 |
| nad3 | 208* | Yes | Yes | No | 70 | C | 16 | [0, 3, 0, 13] | CT | 0.81 | CCT (P) => TTT (F) | 1 |
| nad3 | 209* | Yes | Yes | No | 70 | C | 19 | [0, 5, 0, 14] | CT | 0.74 | CCT (P) => TTT (F) | 2 |
| nad3 | 215 | Yes | No | No | 72 | C | 17 | [0, 7, 0, 10] | CT | 0.59 | CCG (P) => CTG (L) | 2 |
| nad3 | 230 | Yes | No | No | 77 | C | 6 | [0, 2, 0, 4] | CT | 0.67 | TCC (S) => TTC (F) | 2 |
| nad3 | 251* | Yes | Yes | No | 84 | C | 8 | [0, 7, 0, 1] | CT | 0.12 | CCC (P) => CTC (L) | 2 |
| nad3 | 266 | Yes | No | No | 89 | C | 8 | [0, 3, 0, 5] | CT | 0.62 | CCC (P) => CTC (L) | 2 |
| nad3 | 275 | Yes | No | No | 92 | C | 9 | [0, 4, 0, 5] | CT | 0.56 | TCT (S) => TTT (F) | 2 |
| nad3 | 317 | Yes | No | No | 106 | C | 11 | [0, 2, 0, 9] | CT | 0.82 | TCT (S) => TTT (F) | 2 |
| nad3 | 344* | Yes | Yes | No | 115 | C | 8 | [0, 1, 0, 7] | CT | 0.88 | TCG (S) => TTG (L) | 2 |
| nad3 | 349* | Yes | Yes | No | 117 | C | 9 | [0, 2, 0, 7] | CT | 0.78 | CGG (R) => TGG (W) | 1 |
| nad4 | 65* | Yes | Yes | No | 22 | C | 519 | [0, 79, 0, 440] | CT | 0.85 | ACT (T) => ATT (I) | 2 |
| nad4 | 68 | Yes | No | No | 23 | C | 527 | [0, 5, 0, 522] | CT | 0.99 | CCT (P) => CTT (L) | 2 |
| nad4 | 98* | Yes | Yes | No | 33 | C | 428 | [0, 7, 0, 421] | CT | 0.98 | CCG (P) => CTG (L) | 2 |
| nad4 | 149* | Yes | Yes | No | 50 | C | 264 | [0, 8, 0, 256] | CT | 0.97 | CCT (P) => CTT (L) | 2 |
| nad4 | 157* | Yes | Yes | No | 53 | C | 300 | [0, 8, 0, 292] | CT | 0.97 | CGG (R) => TGG (W) | 1 |
| nad4 | 188* | Yes | Yes | No | 63 | C | 193 | [0, 12, 0, 181] | CT | 0.94 | TCT (S) => TTT (F) | 2 |
| nad4 | 353* | Yes | Yes | No | 118 | C | 20 | [0, 2, 0, 18] | CT | 0.9 | ACA (T) => ATA (I) | 2 |
| nad4 | 359 | Yes | No | No | 120 | C | 18 | [0, 3, 0, 15] | CT | 0.83 | TCT (S) => TTT (F) | 2 |
| nad4 | 367* | Yes | Yes | No | 123 | C | 19 | [0, 3, 0, 16] | CT | 0.84 | CGT (R) => TGT (C) | 1 |
| nad4 | 407 | Yes | No | No | 136 | C | 4 | [0, 0, 0, 4] | CT | 1 | CCT (P) => CTT (L) | 2 |
| nad4 | 427* | Yes | Yes | No | 143 | C | 3 | [0, 2, 0, 1] | CT | 0.33 | CCC (P) => TTC (S) | 1 |
| nad4 | 440* | Yes | Yes | No | 147 | C | 3 | [0, 0, 0, 3] | CT | 1 | CCA (P) => CTA (L) | 2 |
| nad4 | 599* | Yes | Yes | No | 200 | C | 1169 | [0, 5, 0, 1164] | CT | 1 | TCA (S) => TTA (L) | 2 |
| nad4 | 650* | Yes | Yes | No | 217 | C | 886 | [0, 32, 0, 854] | CT | 0.96 | TCT (S) => TTT (F) | 2 |
| nad4 | 758* | Yes | Yes | No | 253 | C | 182 | [0, 2, 0, 180] | CT | 0.99 | CCT (P) => CTT (L) | 2 |
| nad4 | 847 | Yes | No | No | 283 | C | 45 | [0, 18, 0, 27] | CT | 0.6 | CCA (P) => TTA (S) | 1 |
| nad4 | 848 | Yes | No | No | 283 | C | 44 | [0, 1, 0, 43] | CT | 0.98 | CCA (P) => TTA (L) | 2 |
| nad4 | 878 | Yes | No | No | 293 | C | 27 | [0, 1, 0, 26] | CT | 0.96 | TCG (S) => TTG (L) | 2 |
| nad4 | 909 | No | No | No | 303 | G | 12 | [0, 2, 10, 0] | GC | 0.17 | AAG (K) => AAC (N) | 3 |
| nad4 | 997 | Yes | No | No | 333 | C | 299 | [0, 214, 0, 85] | CT | 0.28 | CTA (L) => TTA (L) | 1 |
| nad4 | 1001* | Yes | Yes | No | 334 | C | 268 | [0, 40, 0, 228] | CT | 0.85 | CCG (P) => CTG (L) | 2 |
| nad4 | 1007 | Yes | No | No | 336 | C | 302 | [0, 53, 0, 249] | CT | 0.82 | TCA (S) => TTA (L) | 2 |
| nad4 | 1100 | Yes | No | No | 367 | C | 532 | [0, 49, 0, 483] | CT | 0.91 | TCA (S) => TTA (L) | 2 |
| nad4 | 1120* | Yes | Yes | No | 374 | C | 453 | [0, 62, 0, 391] | CT | 0.86 | CTC (L) => TTC (F) | 1 |
| nad4 | 1123 | Yes | No | No | 375 | C | 472 | [0, 23, 0, 449] | CT | 0.95 | CCT (P) => TCT (S) | 1 |
| nad4 | 1142 | Yes | No | No | 381 | C | 423 | [0, 92, 0, 331] | CT | 0.78 | TCC (S) => TTC (F) | 2 |
| nad4 | 1163* | Yes | Yes | No | 388 | C | 385 | [0, 20, 0, 365] | CT | 0.95 | TCA (S) => TTA (L) | 2 |
| nad4 | 1298 | Yes | No | No | 433 | C | 38 | [0, 3, 0, 35] | CT | 0.92 | GCG (A) => GTG (V) | 2 |
| nad4 | 1346* | Yes | Yes | No | 449 | C | 5 | [0, 2, 0, 3] | CT | 0.6 | CCA (P) => CTA (L) | 2 |
| nad4 | 1364 | Yes | No | No | 455 | C | 2 | [0, 0, 0, 2] | CT | 1 | TCC (S) => TTC (F) | 2 |
| nad4 | 1408* | Yes | Yes | No | 470 | C | 14 | [0, 4, 0, 10] | CT | 0.71 | CAC (H) => TAC (Y) | 1 |
| nad4 | 1424* | Yes | Yes | No | 475 | C | 15 | [0, 6, 0, 9] | CT | 0.6 | CCG (P) => CTG (L) | 2 |
| nad4L | 11* | Yes | Yes | No | 4 | C | 51 | [0, 5, 0, 46] | CT | 0.9 | TCT (S) => TTT (F) | 2 |
| nad4L | 17 | Yes | No | No | 6 | C | 60 | [0, 8, 0, 52] | CT | 0.87 | TCA (S) => TTA (L) | 2 |
| nad4L | 25* | Yes | Yes | No | 9 | C | 58 | [0, 7, 0, 51] | CT | 0.88 | CGG (R) => TGG (W) | 1 |
| nad4L | 56* | Yes | Yes | No | 19 | C | 15 | [0, 0, 0, 15] | CT | 1 | CCT (P) => CTT (L) | 2 |
| nad4L | 65* | Yes | Yes | No | 22 | C | 8 | [0, 1, 0, 7] | CT | 0.88 | TCA (S) => TTA (L) | 2 |
| nad4L | 70* | Yes | Yes | No | 24 | C | 5 | [0, 1, 0, 4] | CT | 0.8 | CCA (P) => TCA (S) | 1 |
| nad4L | 80* | Yes | Yes | No | 27 | C | 5 | [0, 0, 0, 5] | CT | 1 | TCA (S) => TTA (L) | 2 |
| nad4L | 101* | Yes | Yes | No | 34 | C | 109 | [0, 55 0, 54] | CT | 0.49 | TCG (S) => TTG (L) | 2 |
| nad4L | 128* | Yes | Yes | No | 43 | C | 5 | [0, 0, 0, 5] | CT | 1 | TCG (S) => TTG (L) | 2 |
| nad4L | 149 | Yes | No | No | 50 | C | 5 | [0, 0, 0, 5] | CT | 1 | TCA (S) => TTA (L) | 2 |
| nad4L | 158* | Yes | Yes | No | 53 | C | 5 | [0, 2, 0, 3] | CT | 0.6 | TCA (S) => TTA (L) | 2 |
| nad4L | 167* | Yes | Yes | No | 56 | C | 4 | [0, 1, 0, 3] | CT | 0.75 | TCA (S) => TTA (L) | 2 |
| nad4L | 251 | Yes | No | No | 84 | C | 3 | [0, 0, 0, 3] | CT | 1 | TCT (S) => TTT (F) | 2 |
| nad5 | 155* | Yes | Yes | No | 52 | C | 34 | [0, 10, 0, 24] | CT | 0.71 | CCG (P) => CTG (L) | 2 |
| nad5 | 242* | Yes | Yes | No | 81 | C | 138 | [0, 16, 0, 122] | CT | 0.88 | CCG (P) => CTG (L) | 2 |
| nad5 | 359 | Yes | No | No | 120 | C | 200 | [0, 13, 0, 187] | CT | 0.94 | TCT (S) => TTT (F) | 2 |
| nad5 | 374* | Yes | Yes | No | 125 | C | 361 | [0, 10, 0, 351] | CT | 0.97 | CCA (P) => CTA (L) | 2 |
| nad5 | 398* | Yes | Yes | No | 133 | C | 354 | [0, 55, 0, 299] | CT | 0.84 | TCT (S) => TTT (F) | 2 |
| nad5 | 539 | Yes | No | No | 180 | C | 422 | [0, 12, 0, 410] | CT | 0.97 | CCT (P) => CTT (L) | 2 |
| nad5 | 548* | Yes | Yes | No | 183 | C | 465 | [0, 42, 1, 422] | CT | 0.91 | TCG (S) => TTG (L) | 2 |
| nad5 | 608* | Yes | Yes | No | 203 | C | 445 | [0, 4, 0, 441] | CT | 0.99 | GCC (A) => GTC (V) | 2 |
| nad5 | 609* | Yes | Yes | No | 203 | C | 427 | [0, 361, 0, 66] | CT | 0.15 | GCC (A) => GTT (V) | 2 |
| nad5 | 629* | Yes | Yes | No | 210 | C | 429 | [0, 19, 0, 410] | CT | 0.96 | TCT (S) => TTT (F) | 2 |
| nad5 | 676* | Yes | Yes | No | 226 | C | 360 | [0, 4, 0, 356] | CT | 0.99 | CTT (L) => TTT (F) | 1 |
| nad5 | 713* | Yes | Yes | No | 238 | C | 418 | [0, 26, 0, 392] | CT | 0.94 | TCG (S) => TTG (L) | 2 |
| nad5 | 725* | Yes | Yes | No | 242 | C | 440 | [0, 5, 0, 435] | CT | 0.99 | TCA (S) => TTA (L) | 2 |
| nad5 | 835* | Yes | Yes | No | 279 | C | 433 | [0, 13, 0, 420] | CT | 0.97 | CCG (P) => TCG (S) | 1 |
| nad5 | 1310 | Yes | No | No | 437 | C | 66 | [0, 19, 0, 47] | CT | 0.71 | TCA (S) => TTA (L) | 2 |
| nad5 | 1490* | Yes | Yes | No | 497 | C | 404 | [0, 6, 0, 398] | CT | 0.99 | CCC (P) => CTC (L) | 2 |
| nad5 | 1550* | Yes | Yes | No | 517 | C | 787 | [0, 55, 0, 732] | CT | 0.93 | ACC (T) => ATC (I) | 2 |
| nad5 | 1580* | Yes | Yes | No | 527 | C | 764 | [0, 33, 0, 731] | CT | 0.96 | ACA (T) => ATA (I) | 2 |
| nad5 | 1589 | Yes | No | No | 530 | C | 739 | [0, 150, 0, 589] | CT | 0.8 | TCT (S) => TTT (F) | 2 |
| nad5 | 1610* | Yes | Yes | No | 537 | C | 881 | [0, 55, 0, 826] | CT | 0.94 | TCC (S) => TTC (F) | 2 |
| nad5 | 1731 | No | No | No | 577 | C | 186 | [0, 159, 0, 27] | CT | 0.15 | TTC (F) => TTT (F) | 3 |
| nad5 | 1895* | Yes | Yes | No | 632 | C | 50 | [0, 14, 0, 36] | CT | 0.72 | TCA (S) => TTA (L) | 2 |
| nad5 | 1916* | Yes | Yes | No | 639 | C | 30 | [0, 7, 0, 23] | CT | 0.77 | TCT (S) => TTT (F) | 2 |
| nad5 | 1918* | Yes | Yes | No | 640 | C | 32 | [0, 7, 0, 25] | CT | 0.78 | CGT (R) => TGT (C) | 1 |
| nad5 | 1958* | Yes | Yes | No | 653 | C | 22 | [0, 2, 0, 20] | CT | 0.91 | TCG (S) => TTG (L) | 2 |
| nad5 | 1981 | Yes | No | No | 661 | C | 14 | [0, 0, 0, 14] | CT | 1 | CGT (R) => TGT (C) | 1 |
| nad6 | 26* | Yes | Yes | No | 9 | C | 894 | [0, 15, 0, 879] | CT | 0.98 | CCT (P) => CTT (L) | 2 |
| nad6 | 88* | Yes | Yes | No | 30 | C | 891 | [1, 135, 0, 755] | CT | 0.85 | CCC (P) => TTC (F) | 1 |
| nad6 | 89* | Yes | Yes | No | 30 | C | 870 | [0, 317, 0, 553] | CT | 0.64 | CCC (P) => TTC (F) | 2 |
| nad6 | 95* | Yes | Yes | No | 32 | C | 889 | [0, 242, 0, 647] | CT | 0.73 | CCA (P) => CTA (L) | 2 |
| nad6 | 103* | Yes | Yes | No | 35 | C | 925 | [0, 139, 0, 786] | CT | 0.85 | CGC (R) => TGC (C) | 1 |
| nad6 | 161* | Yes | Yes | No | 54 | C | 1125 | [0, 87, 0, 1038] | CT | 0.92 | CCA (P) => CTA (L) | 2 |
| nad6 | 169* | Yes | Yes | No | 57 | C | 1175 | [0, 32, 0, 1143] | CT | 0.97 | CAT (H) => TAT (Y) | 1 |
| nad6 | 191* | Yes | Yes | No | 64 | C | 1390 | [0, 3, 0, 1387] | CT | 1 | TCA (S) => TTA (L) | 2 |
| nad6 | 360 | No | No | No | 120 | C | 1632 | [0, 756, 0, 876] | CT | 0.54 | ACC (T) => ACT (T) | 3 |
| nad6 | 463* | Yes | Yes | No | 155 | C | 405 | [0, 5, 0, 400] | CT | 0.99 | CCT (P) => TCT (S) | 1 |
| nad6 | 569 | Yes | No | No | 190 | C | 26 | [0, 2, 0, 24] | CT | 0.92 | TCT (S) => TTT (F) | 2 |
| nad7 | 244* | Yes | Yes | No | 82 | C | 400 | [0, 2, 0, 398] | CT | 0.99 | CAT (H) => TAT (Y) | 1 |
| nad7 | 251* | Yes | Yes | No | 84 | C | 407 | [0, 4, 0, 403] | CT | 0.99 | TCA (S) => TTA (L) | 2 |
| nad7 | 316* | Yes | Yes | No | 106 | C | 430 | [0, 1, 0, 429] | CT | 1 | CGT (R) => TGT (C) | 1 |
| nad7 | 335* | Yes | Yes | No | 112 | C | 416 | [0, 0, 0, 416] | CT | 1 | TCA (S) => TTA (L) | 2 |
| nad7 | 344* | Yes | Yes | No | 115 | C | 392 | [0, 1, 0, 391] | CT | 1 | TCA (S) => TTA (L) | 2 |
| nad7 | 383 | Yes | No | No | 128 | C | 152 | [0, 0, 0, 152] | CT | 1 | TCA (S) => TTA (L) | 2 |
| nad7 | 531 | Yes | No | No | 177 | C | 12 | [0, 7, 0, 5] | CT | 0.42 | TCC (S) => TCT (S) | 3 |
| nad7 | 578* | Yes | Yes | No | 193 | C | 12 | [0, 0, 0, 12] | CT | 1 | TCA (S) => TTA (L) | 2 |
| nad7 | 724* | Yes | Yes | No | 242 | C | 9 | [0, 0, 0, 9] | CT | 1 | CAT (H) => TAT (Y) | 1 |
| nad7 | 739* | Yes | Yes | No | 247 | C | 10 | [0, 0, 0, 10] | CT | 1 | CCT (P) => TTT (F) | 1 |
| nad7 | 740 | Yes | No | No | 247 | C | 9 | [0, 0, 0, 9] | CT | 1 | CCT (P) => TTT (F) | 2 |
| nad7 | 769* | Yes | Yes | No | 257 | C | 6 | [0, 0, 0, 6] | CT | 1 | CGC (R) => TGC (C) | 1 |
| nad7 | 944 | Yes | No | No | 315 | C | 43 | [0, 2, 0, 41] | CT | 0.95 | CCT (P) => CTT (L) | 2 |
| nad7 | 963 | No | No | No | 321 | C | 112 | [0, 43, 0, 69] | CT | 0.62 | TCC (S) => TCT (S) | 3 |
| nad7 | 1050 | Yes | No | No | 350 | C | 150 | [0, 0, 0, 150] | CT | 1 | CCC (P) => CCT (P) | 3 |
| nad7 | 1057* | Yes | Yes | No | 353 | C | 134 | [0, 0, 0, 134] | CT | 1 | CGT (R) => TGT (C) | 1 |
| nad7 | 1103* | Yes | Yes | No | 368 | C | 48 | [0, 0, 0, 48] | CT | 1 | TCT (S) => TTT (F) | 2 |
| nad7 | 1124* | Yes | Yes | No | 375 | C | 27 | [0, 0, 0, 27] | CT | 1 | CCA (P) => CTA (L) | 2 |
| nad7 | 1137 | Yes | No | No | 379 | C | 411 | [0, 312, 0, 99] | CT | 0.24 | GTC (V) => GTT (V) | 3 |
| nad7 | 1166 | Yes | No | No | 389 | C | 4 | [0, 0, 0, 4] | CT | 1 | TCT (S) => TTT (F) | 2 |
| nad9 | 92* | Yes | Yes | No | 31 | C | 3260 | [0, 163, 0, 3097] | CT | 0.95 | TCT (S) => TTT (F) | 2 |
| nad9 | 167* | Yes | Yes | No | 56 | C | 5301 | [0, 59, 0, 5242] | CT | 0.99 | TCG (S) => TTG (L) | 2 |
| nad9 | 289 | No | No | Yes | 97 | C | 8730 | [0, 5633, 0, 3097] | CT | 0.35 | CGA (R) => TGA (*) | 1 |
| nad9 | 298* | Yes | Yes | No | 100 | C | 7855 | [0, 79, 0, 7776] | CT | 0.99 | CCG (P) => TCG (S) | 1 |
| nad9 | 310 | No | No | No | 104 | C | 7968 | [0, 5130, 0, 2838] | CT | 0.36 | CTA (L) => TTA (L) | 1 |
| nad9 | 328* | Yes | Yes | No | 110 | C | 6124 | [0, 29, 1, 6094] | CT | 1 | CGG (R) => TGG (W) | 1 |
| nad9 | 368 | Yes | No | No | 123 | C | 5024 | [0, 20, 0, 5004] | CT | 1 | TCC (S) => TTC (F) | 2 |
| nad9 | 398* | Yes | Yes | No | 133 | C | 3369 | [0, 23, 0, 3346] | CT | 0.99 | TCA (S) => TTA (L) | 2 |
| nad9 | 439* | Yes | Yes | No | 147 | C | 1804 | [0, 21, 0, 1783] | CT | 0.99 | CTT (L) => TTT (F) | 1 |
| rpl10 | 9 | No | No | No | 3 | C | 1058 | [0, 815, 0, 243] | CT | 0.23 | TTC (F) => TTT (F) | 3 |
| rpl10 | 101 | Yes | No | No | 34 | C | 956 | [0, 50, 0, 906] | CT | 0.95 | TCA (S) => TTA (L) | 2 |
| rpl10 | 134 | Yes | No | No | 45 | C | 1166 | [0, 29, 0, 1137] | CT | 0.98 | CCA (P) => CTA (L) | 2 |
| rpl10 | 174 | No | No | No | 58 | C | 1396 | [0, 874, 0, 522] | CT | 0.37 | TTC (F) => TTT (F) | 3 |
| rpl10 | 180 | No | No | No | 60 | C | 1312 | [0, 221, 0, 1091] | CT | 0.83 | ACC (T) => ATC (I) | 2 |
| rpl10 | 330 | No | No | No | 110 | C | 763 | [0, 492, 0, 271] | CT | 0.36 | TAC (Y) => TAT (Y) | 3 |
| rpl10 | 371 | No | No | No | 124 | C | 519 | [0, 391, 0, 128] | CT | 0.25 | TCA (S) => TTA (L) | 2 |
| rpl16 | 221* | Yes | Yes | No | 74 | C | 158 | [0, 4, 0, 154] | CT | 0.97 | TCG (S) => TTG (L) | 2 |
| rpl5 | 35* | Yes | Yes | No | 12 | C | 1188 | [0, 77, 0, 1111] | CT | 0.94 | TCA (S) => TTA (L) | 2 |
| rpl5 | 47* | Yes | Yes | No | 16 | C | 1279 | [0, 66, 0, 1213] | CT | 0.95 | CCG (P) => CTG (L) | 2 |
| rpl5 | 160 | Yes | No | No | 54 | C | 1287 | [0, 455, 0, 832] | CT | 0.65 | CCG (P) => TCG (S) | 1 |
| rpl5 | 414 | No | No | No | 138 | C | 455 | [0, 129, 0, 326] | CT | 0.72 | TCC (S) => TCT (S) | 2 |
| rpl5 | 441 | Yes | No | No | 147 | C | 333 | [0, 54, 0, 279] | CT | 0.84 | ATC (I) => ATT (I) | 3 |
| rpl5 | 509* | Yes | Yes | No | 170 | C | 82 | [0, 1, 0, 81] | CT | 0.99 | CCA (P) => CTA (L) | 2 |
| rpl5 | 512 | Yes | No | No | 171 | C | 79 | [0, 3, 0, 76] | CT | 0.96 | CCG (P) => CTG (L) | 2 |
| rps12 | 104* | Yes | Yes | No | 35 | C | 979 | [0, 12, 0, 967] | CT | 0.99 | CCG (P) => CTG (L) | 2 |
| rps12 | 159 | Yes | No | No | 53 | C | 455 | [0, 111, 0, 344] | CT | 0.76 | GCC (A) => GCT (A) | 3 |
| rps12 | 196* | Yes | Yes | No | 66 | C | 289 | [0, 1, 0, 288] | CT | 1 | CAC (H) => TAC (Y) | 1 |
| rps12 | 221* | Yes | Yes | No | 74 | C | 199 | [0, 5, 0, 194] | CT | 0.97 | TCG (S) => TTG (L) | 2 |
| rps12 | 284* | Yes | Yes | No | 95 | C | 71 | [0, 0, 0, 71] | CT | 1 | TCC (S) => TTC (F) | 2 |
| rps13 | 56 | Yes | No | No | 19 | C | 1113 | [0, 16, 0, 1097] | CT | 0.99 | TCA (S) => TTA (L) | 2 |
| rps13 | 100 | Yes | No | No | 34 | C | 830 | [0, 19, 0, 811] | CT | 0.98 | CGT (R) => TGT (C) | 1 |
| rps13 | 287 | Yes | No | No | 96 | C | 202 | [0, 3, 0, 199] | CT | 0.99 | TCG (S) => TTG (L) | 2 |
| rps3 | 92 | No | No | No | 31 | C | 140 | [0, 22, 0, 118] | CT | 0.84 | TCA (S) => TTA (L) | 2 |
| rps3 | 512 | No | No | No | 171 | C | 773 | [0, 27, 0, 746] | CT | 0.97 | TCA (S) => TTA (L) | 2 |
| rps3 | 713 | No | No | No | 238 | C | 862 | [1, 51, 0, 810] | CT | 0.94 | TCG (S) => TTG (L) | 2 |
| rps3 | 1152 | No | No | No | 384 | C | 1050 | [0, 816, 0, 234] | CT | 0.22 | TTC (F) => TTT (F) | 3 |
| rps3 | 1355 | Yes | No | No | 452 | C | 1005 | [0, 26, 0, 97] | CT | 0.97 | CCG (P) => CTG (L) | 2 |
| rps3 | 1413 | No | No | No | 471 | C | 810 | [0, 693, 0, 117] | CT | 0.14 | TCC (S) => TCT (S) | 3 |
| rps3 | 1496 | Yes | No | No | 499 | C | 442 | [0, 15, 0, 427] | CT | 0.97 | TCA (S) => TTA (L) | 2 |
| rps3 | 1582 | Yes | No | No | 528 | C | 254 | [0, 8, 0, 246] | CT | 0.97 | CCT (P) => TCT (S) | 1 |
| rps4 | 176 | Yes | No | No | 59 | C | 556 | [0, 57, 0, 499] | CT | 0.9 | TCA (S) => TTA (L) | 2 |
| rps4 | 205* | Yes | Yes | No | 69 | C | 484 | [0, 14, 0, 470] | CT | 0.97 | CAT (H) => TAT (Y) | 1 |
| rps4 | 219 | Yes | No | No | 73 | C | 474 | [0, 338, 0, 136] | CT | 0.29 | CCC (P) => CCT (P) | 3 |
| rps4 | 275 | Yes | No | No | 92 | C | 507 | [0, 30, 0, 477] | CT | 0.94 | CCA (P) => CTA (L) | 2 |
| rps4 | 287 | Yes | No | No | 96 | C | 491 | [0, 20, 0, 471] | CT | 0.96 | TCG (S) => TTG (L) | 2 |
| rps4 | 299 | Yes | No | No | 100 | C | 538 | [0, 15, 0, 523] | CT | 0.97 | CCG (P) => CTG (L) | 2 |
| rps4 | 316 | Yes | No | No | 106 | C | 510 | [0, 323, 0, 187] | CT | 0.37 | CGT (R) => TGT (C) | 1 |
| rps4 | 344 | Yes | No | No | 115 | C | 525 | [0, 20, 0, 505] | CT | 0.96 | CCG (P) => CTG (L) | 2 |
| rps4 | 449 | No | No | No | 150 | C | 532 | [0, 420, 0, 112] | CT | 0.21 | ACC (T) => ATC (I) | 2 |
| rps4 | 491 | Yes | No | No | 164 | C | 531 | [0, 22, 0, 509] | CT | 0.96 | TCA (S) => TTA (L) | 2 |
| rps4 | 504 | No | No | No | 168 | C | 562 | [0, 308, 0, 254] | CT | 0.45 | TTC (F) => TTT (F) | 3 |
| rps4 | 791 | No | No | No | 264 | C | 439 | [0, 345, 0, 94] | CT | 0.21 | CCT (P) => CTT (L) | 2 |
| rps4 | 935* | Yes | Yes | No | 312 | C | 173 | [0, 14, 0, 159] | CT | 0.92 | TCG (S) => TTG (L) | 2 |
| rps4 | 946* | Yes | Yes | No | 316 | C | 182 | [0, 11, 0, 171] | CT | 0.94 | CAT (H) => TAT (Y) | 1 |
| rps4 | 956 | Yes | No | No | 319 | C | 148 | [0, 116, 0, 32] | CT | 0.22 | CCA (P) => CTA (L) | 2 |
| rps4 | 971* | Yes | Yes | No | 324 | C | 117 | [0, 22, 0, 95] | CT | 0.81 | TCT (S) => TTT (F) | 2 |
| rps4 | 1022* | Yes | Yes | No | 341 | C | 39 | [0, 0, 0, 39] | CT | 1 | CCA (P) => CTA (L) | 2 |
| rps4 | 1036* | Yes | Yes | No | 346 | C | 33 | [0, 2, 0, 31] | CT | 0.94 | CGG (R) => TGG (W) | 1 |

#### Table S11 Summary of single nucleotide polymorphisms (SNPs) detected in the PCGs of *S. splendens* mitogenome. “Nt Pos” means the position of the SNPs in the nucleotide sequences of the PCGs.

| Gene | Nt Pos | Nt Reference | Nt Coverage | Nt Base Count [A,C,G,T] | Nt Allsubs | Nt Frequency |
| --- | --- | --- | --- | --- | --- | --- |
| atp1 | 65 | A | 206 | [132, 0, 0, 74] | AT | 0.36 |
| atp1 | 69 | G | 202 | [0, 0, 131, 71] | GT | 0.35 |
| atp1 | 71 | A | 201 | [130, 0, 71, 0] | AG | 0.35 |
| atp1 | 79 | G | 210 | [0, 0, 135, 75] | GT | 0.36 |
| atp1 | 102 | C | 217 | [0, 134, 83, 0] | CG | 0.38 |
| atp1 | 126 | G | 225 | [0, 0, 139, 86] | GT | 0.38 |
| atp1 | 183 | G | 244 | [0, 97, 147, 0] | GC | 0.4 |
| atp1 | 198 | A | 264 | [159, 0, 105, 0] | AG | 0.4 |
| atp1 | 219 | A | 248 | [147, 0, 101, 0] | AG | 0.41 |
| atp1 | 222 | G | 251 | [98, 0, 153, 0] | GA | 0.39 |
| atp1 | 246 | T | 264 | [0, 107, 0, 157] | TC | 0.41 |
| atp1 | 261 | A | 287 | [169, 0, 118, 0] | AG | 0.41 |
| atp1 | 273 | G | 271 | [109, 0, 162, 0] | GA | 0.4 |
| atp1 | 336 | C | 277 | [0, 166, 111, 0] | CG | 0.4 |
| atp1 | 342 | A | 261 | [167, 0, 94, 0] | AG | 0.36 |
| atp1 | 374 | C | 261 | [91, 170, 0, 0] | CA | 0.35 |
| atp1 | 411 | A | 283 | [190, 0, 93, 0] | AG | 0.33 |
| atp1 | 453 | G | 274 | [0, 0, 184, 90] | GT | 0.33 |
| atp1 | 568 | C | 186 | [27, 159, 0, 0] | CA | 0.15 |
| atp1 | 581 | A | 181 | [157, 2, 22, 0] | AG AC | 0.12 |
| atp1 | 906 | C | 766 | [0, 193, 0, 573] | CT | 0.75 |
| atp1 | 912 | A | 749 | [188, 0, 561, 0] | AG | 0.75 |
| atp1 | 918 | C | 742 | [556, 176, 0, 10] | CA CT | 0.76 |
| atp1 | 921 | T | 778 | [0, 570, 0, 208] | TC | 0.73 |
| atp1 | 924 | C | 648 | [0, 200, 0, 448] | CT | 0.69 |
| atp1 | 936 | G | 684 | [0, 493, 191, 0] | GC | 0.72 |
| atp1 | 996 | A | 185 | [158, 0, 0, 27] | AT | 0.15 |
| atp1 | 1000 | G | 185 | [0, 0, 153, 32] | GT | 0.17 |
| atp1 | 1002 | A | 191 | [152, 0, 39, 0] | AG | 0.2 |
| atp1 | 1023 | T | 1156 | [1, 1002, 0, 153] | TC TA | 0.87 |
| atp1 | 1032 | T | 1139 | [0, 988, 0, 151] | TC | 0.87 |
| atp1 | 1059 | C | 189 | [20, 169, 0, 0] | CA | 0.11 |
| atp9 | 231 | T | 210 | [47, 0, 0, 163] | TA | 0.22 |
| ccmFn | 1712 | C | 182 | [29, 153, 0, 0] | CA | 0.16 |
| rps13 | 5 | T | 132 | [0, 16, 0, 116] | TC | 0.12 |
| rps13 | 8 | A | 132 | [117, 15, 0, 0] | AC | 0.11 |
| rps3 | 1510 | T | 206 | [0, 29, 0, 177] | TC | 0.14 |

#### Table S12 Variation sites within the intron regions of 12 mitochondrial genes in *S. miltiorrhiza*, *S. officinalis*, and *S. splendens*.

| Name of intron | Position | Loci of *S. miltiorrhiza* | Loci of *S. officinalis* | Loci of *S. splendens* |
| --- | --- | --- | --- | --- |
| cox1i12 | 341 | T | A | T |
| cox1i12 | 749 | TATGAT | T | T |
| cox2i12 | 698 | T | T | TCTTGT |
| cox2i12 | 825 | T | A | A |
| cox2i12 | 826 | T | C | C |
| cox2i12 | 832 | TCAAT | T | TCAAT |
| cox2i12 | 854 | A | A | C |
| cox2i12 | 880 | G | A | G |
| cox2i12 | 895 | A | ATATAT | ATATAT |
| cox2i12 | 904 | T | T | G |
| cox2i12 | 1083 | C | A | C |
| nad1i23 | 611 | ACTAGA | A | A |
| nad1i23 | 698 | T | TATTATATTCT | TATTATATTCT |
| nad2i12 | 583 | A | G | A |
| nad2i12 | 669 | A | A | C |
| nad2i12 | 689 | ATAGAGCTA | ATAGAGCTA | A |
| nad2i34 | 737 | A | ATTGGA | ATTGGA |
| nad2i34 | 880 | C | CCGGGCGGCGGGC | CCGGGCGGCGGGC |
| nad2i34 | 932 | A | C | A |
| nad2i34 | 1023 | A | G | G |
| nad2i45 | 713 | TTTGGAT | TTTGGAT | T |
| nad2i45 | 778 | CC | C | C |
| nad4i12 | 782 | T | T | TTATGT |
| nad4i12 | 868 | C | T | C |
| nad4i12 | 897 | A | G | G |
| nad4i34 | 2417 | AGCCCAAAACAA | A | AGCCCA |
| nad4i34 | 2432 | A | A | C |
| nad4i34 | 2433 | G | G | A |
| nad4i34 | 2434 | G | G | A |
| nad4i34 | 2438 | G | G | A |
| nad4i34 | 2439 | T | T | G |
| nad4i34 | 2440 | T | T | G |
| nad4i34 | 2472 | CGCGAACATAC | CGCGAACATAC | C |
| nad5i45 | 425 | T | G | A |
| nad5i45 | 473 | TTCGCGAGCCTTCACT | TTCGCGAGCCTTCACT | T |
| nad7i12 | 226 | G | G | GAG |
| nad7i12 | 422 | C | T | C |
| nad7i12 | 525 | GCATAGCATAGCATAG | G | G |
| nad7i23 | 575 | A | C | A |
| nad7i23 | 586 | C | CGCTCCCC | CGCTCCCC |
| nad7i23 | 700 | A | AGGGA | AGGGA |
| nad7i23 | 777 | T | T | TTTGTCT |
| nad7i23 | 874 | TCTTT | T | T |
| nad7i23 | 904 | C | A | C |
| nad7i34 | 146 | A | C | C |
| nad7i34 | 379 | A | A | G |
| nad7i34 | 610 | AA | A | AA |

#### Table S13 Primer sequences used for amplifying intron polymorphism molecular markers in *S. miltiorrhiza*, *S. officinalis*, and *S. splendens*.

| Primer Name | Primer Sequence (5’->3’) |
| --- | --- |
| cox1i12-F | AGTGGTTTTCTACCCCGATCT |
| cox1i12-R | AGCTAGCCATGCTTTATGGTGA |
| cox2i12-F | GCGCCACTCTCTTTCTGAGT |
| cox2i12-R | GGTTGGCCACTCTGCTATGT |
| nad1i23-F | CTGTTCCCCTAACCCAACCC |
| nad1i23-R | CGTCCCGTCTCATCGTGATT |
| nad2i12-F | CTTCTTAGAGGAGGGTTGTTTGC |
| nad2i12-R | GAGAAAGCTCGCCGGAGAAT |
| nad2i34-F | CCTCCTTCTTTGCTTTCTTCCAA |
| nad2i34-R | TCCGCTCTCGGCCATTCC |
| nad2i45-F | AGGAGAGCAAAGCGCCAAT |
| nad2i45-R | TGAAGAGGGGCCAAGGATCT |
| nad4i12-F | TCGTCCAGGTCTTATTGTGAAA |
| nad4i12-R | CAACGCCCGTCTTCCAAATC |
| nad4i34-F | TGAGAAAAAGGGGTCGGTCA |
| nad4i34-R | TTCCTGCCGGATATCAAAACA |
| nad5i45-F | GCGAGTGAAGTGCTTACGCC |
| nad5i45-R | AGTTTTCTCCCTTTCTCTCGCT |
| nad7i12-F | GCAACTCAAGCGAACCGC |
| nad7i12-R | TATAGGCTCGCTCCCTCTTT |
| nad7i23-F | TGAGAACCGTGTGAACGGAG |
| nad7i23-R | GCAAAAGGAAGGTTGGGTGC |
| nad7i34-F | AGGAGTAAAGCATCCCGAGG |
| nad7i34-R | TGATTAAAAAGGGAAAGGGCTCA |

Fig. S1 The alignment of the Nanopore reads to the four configurations of the three double bifurcating structures (DBS), named DBS01-DBS03 found in the unitig graph. The unitig graph was generated using Unicycler from Illumina reads that were filtered with GetOrganelle for mitochondrial reads. The major configurations (Mac) refer to the configurations of the DBS structure supported with more long reads. The minor configurations (Mic) refer to the configurations of the DBS structure supported with less long reads. It should be emphasized that the repetitive sequences affiliated with DBS01, DBS02, and DBS03 are identical to the repetitive sequences: R01, R04, and R07, respectively. Each figure can be divided into three parts from top to bottom. The top part shows the reference sequences with their coordinates. The middle part shows the read coverage bar chart in blue. The name and length of the repeat and the name of the configuration are shown above the coverage plot. The bottom part shows the reads' alignment to the reference sequences. The repeat region is shown as a double-headed arrow. The boundaries of the repeat regions of the DBS structure are indicated with red vertical lines.

A Mapping of long reads to the major configuration 1 (Mac1) of the DBS01 affiliated with the R01.


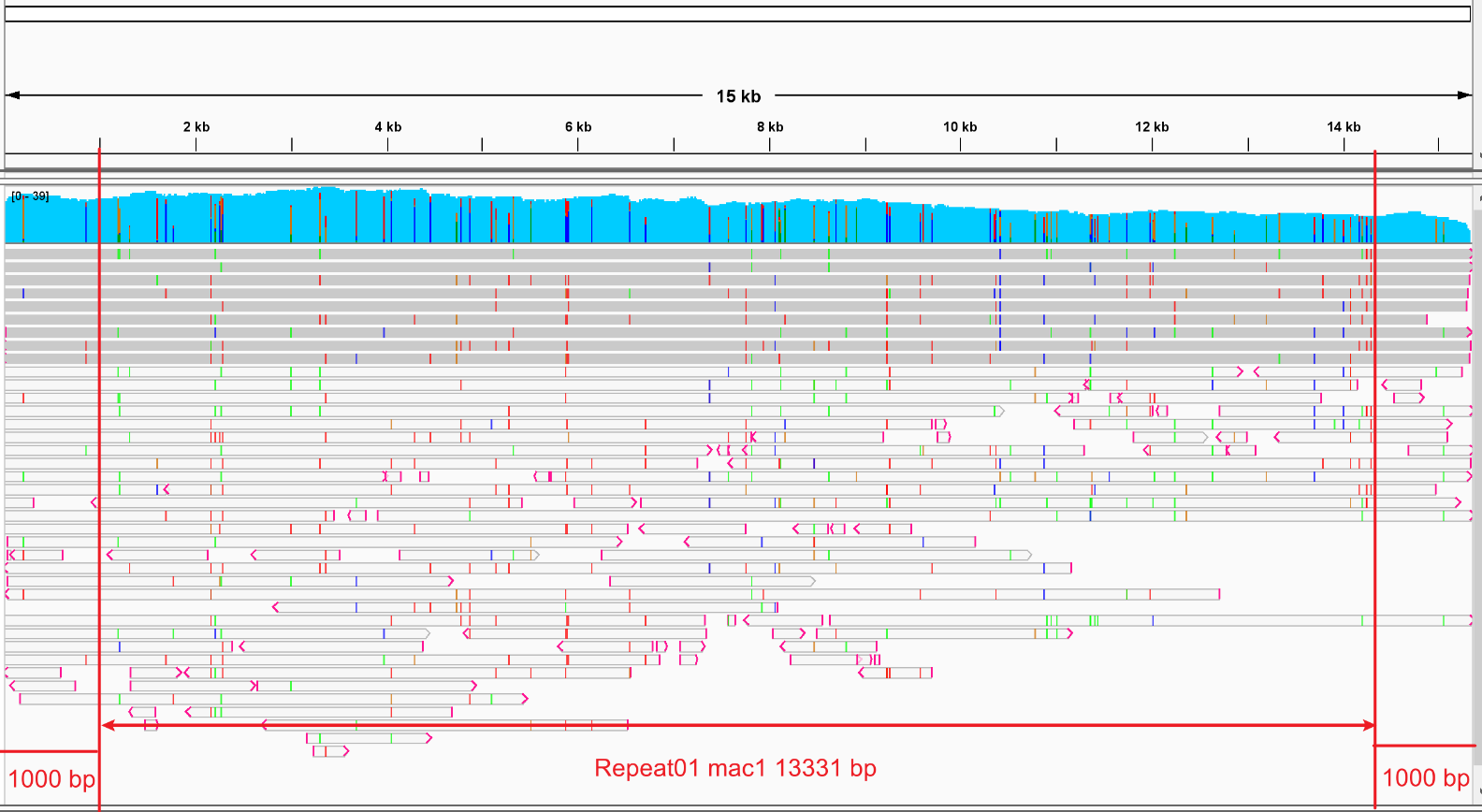


B Mapping of long reads to the major configuration 2 (Mac2) of the DBS01 affiliated with the R01.


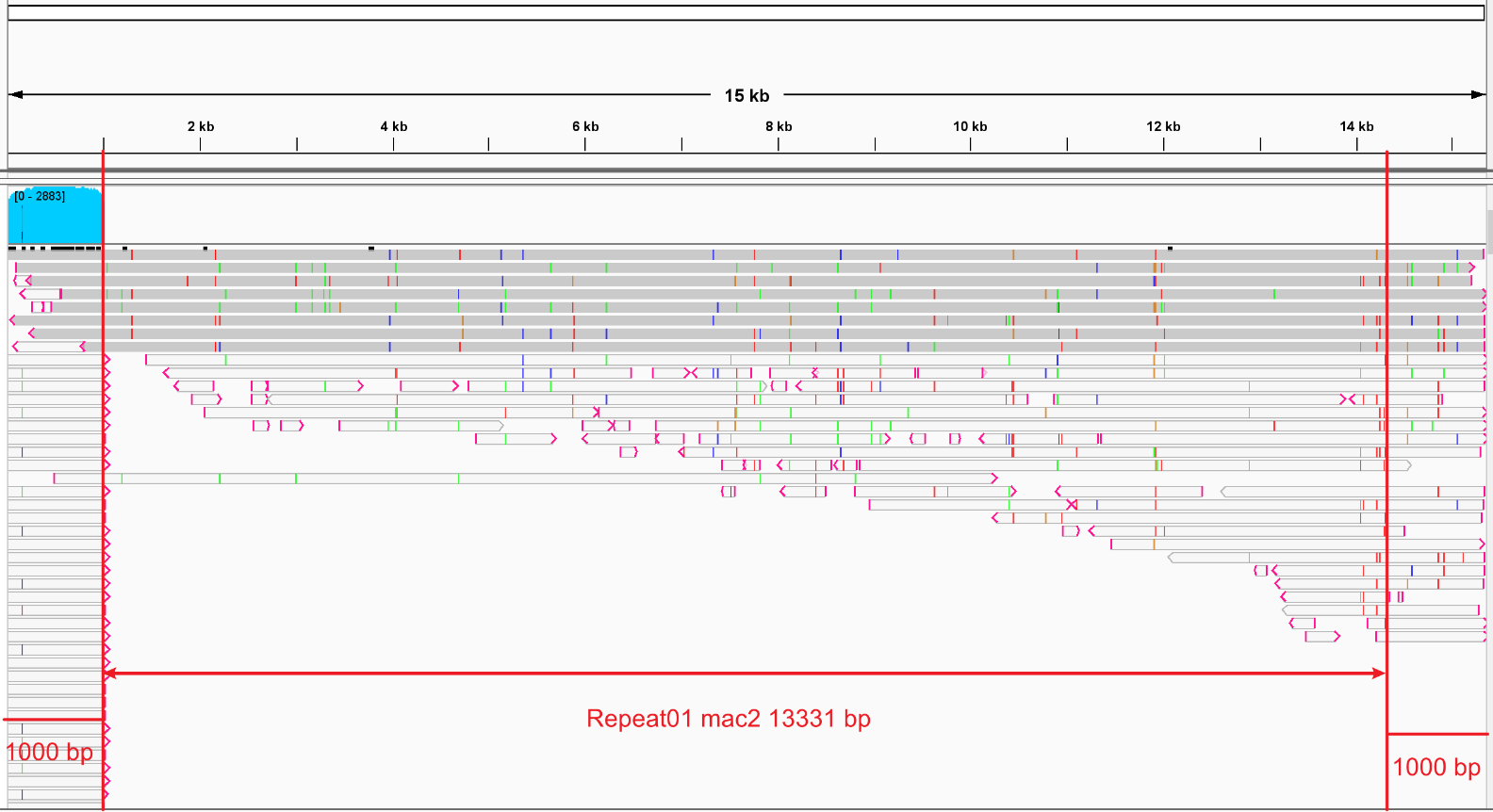


C Mapping of long reads to the minor configuration 1 (Mic1) of the DBS01 affiliated with the R01.


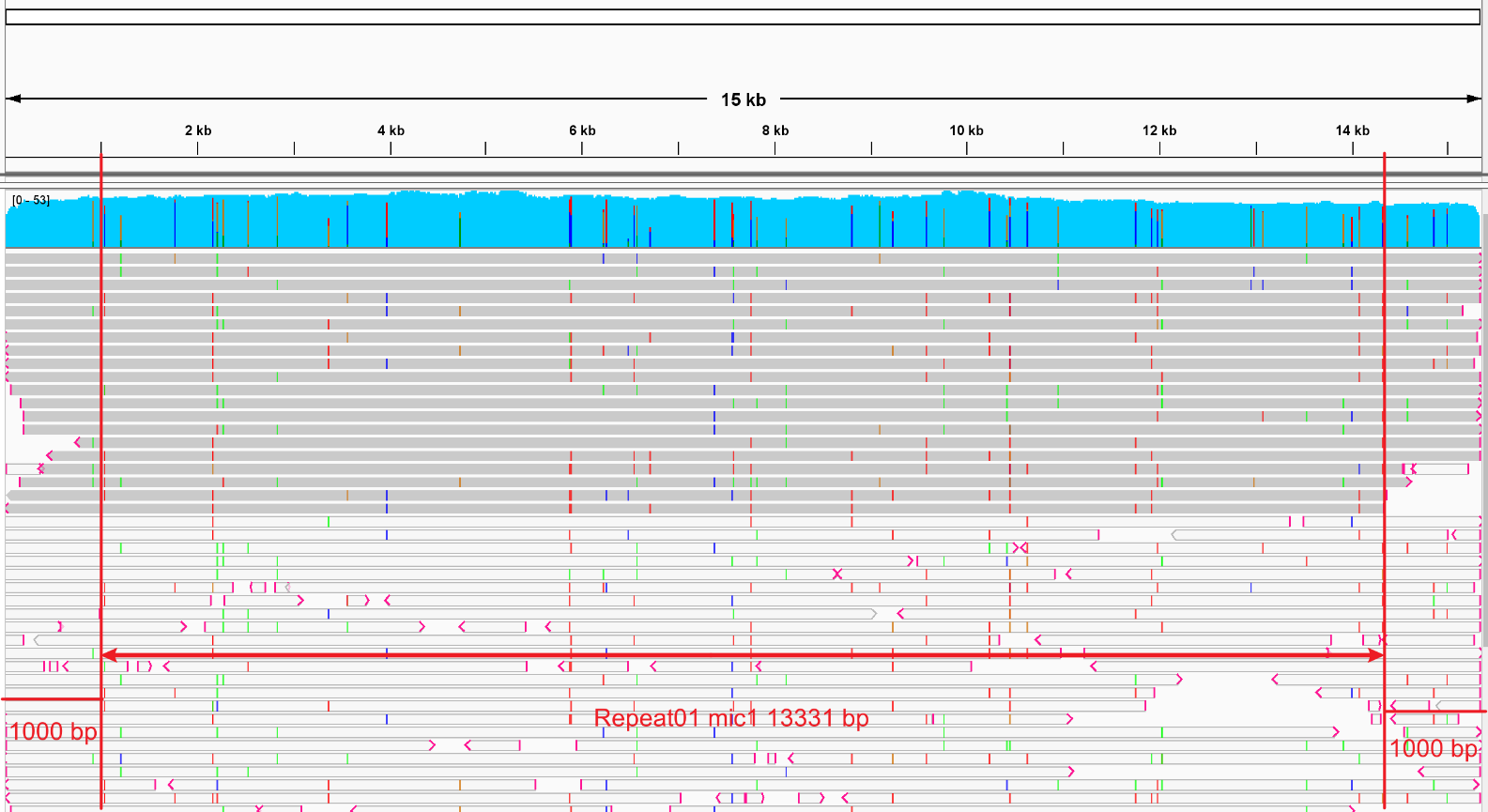


D Mapping of long reads to the minor configuration 2 (Mic2) of the DBS01 affiliated with the R01.


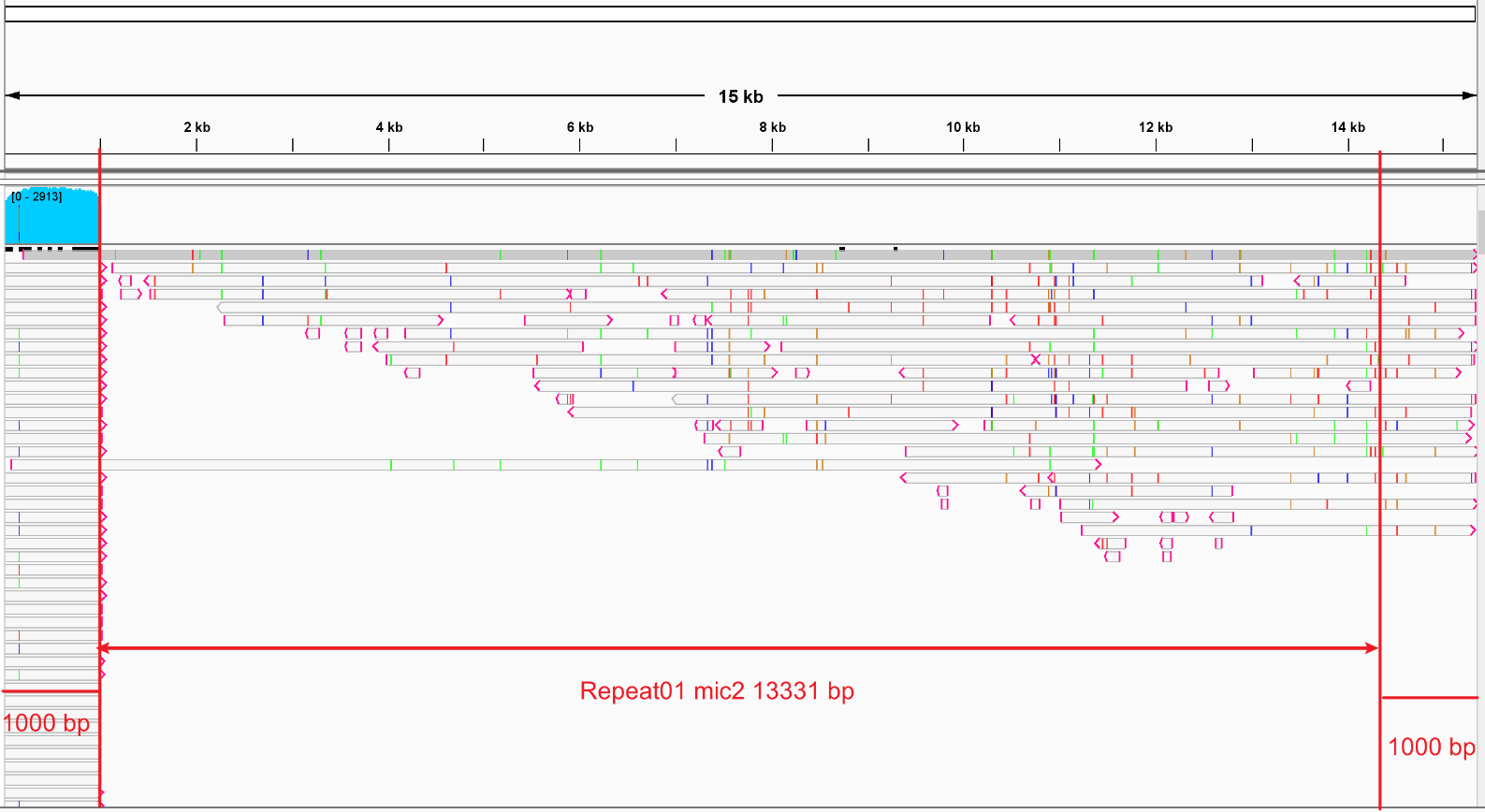


E Mapping of long reads to the major configuration 1 (Mac1) of the DBS02 affiliated with the R04.


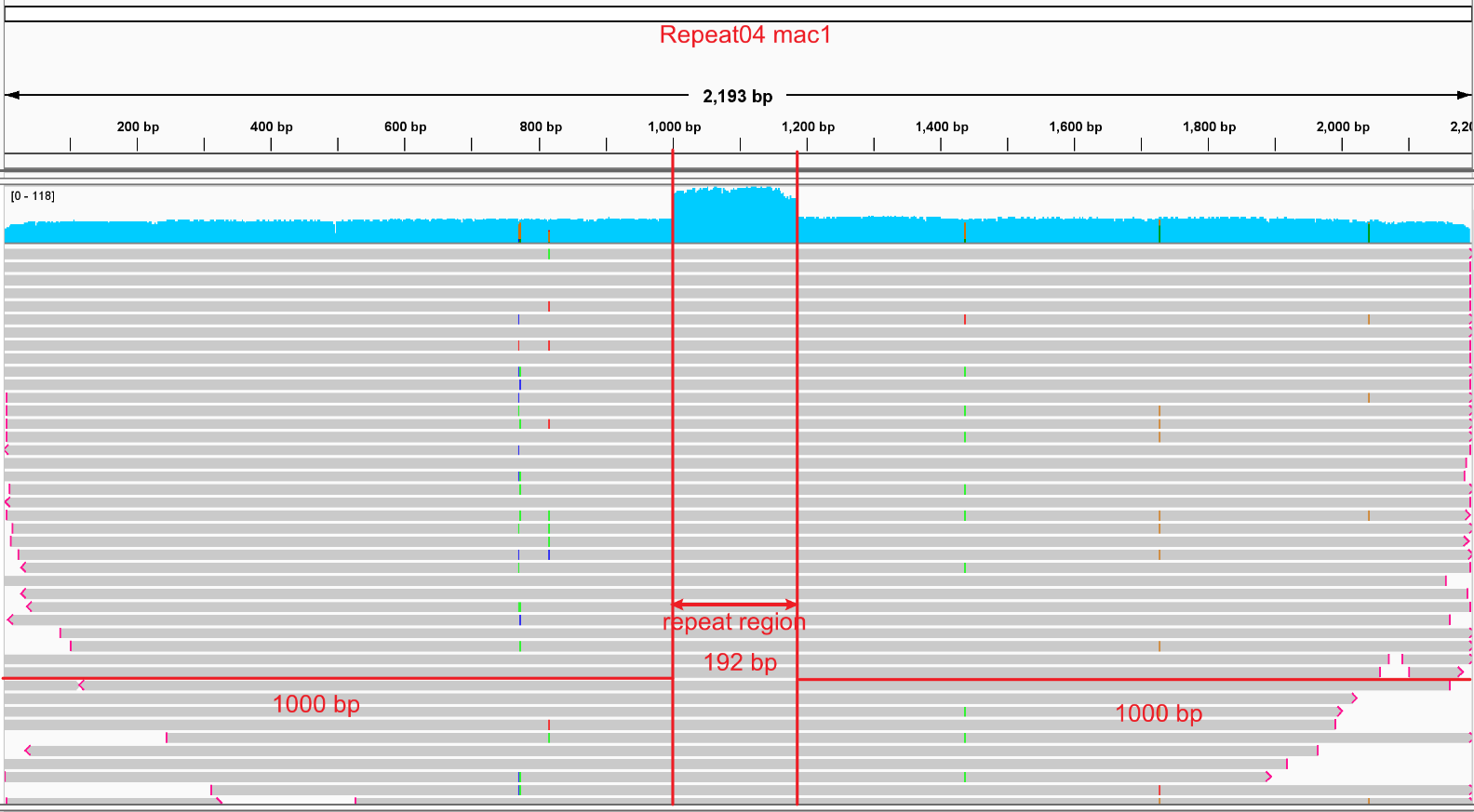


F Mapping of long reads to the major configuration 2 (Mac2) of the DBS02 affiliated with the R04.


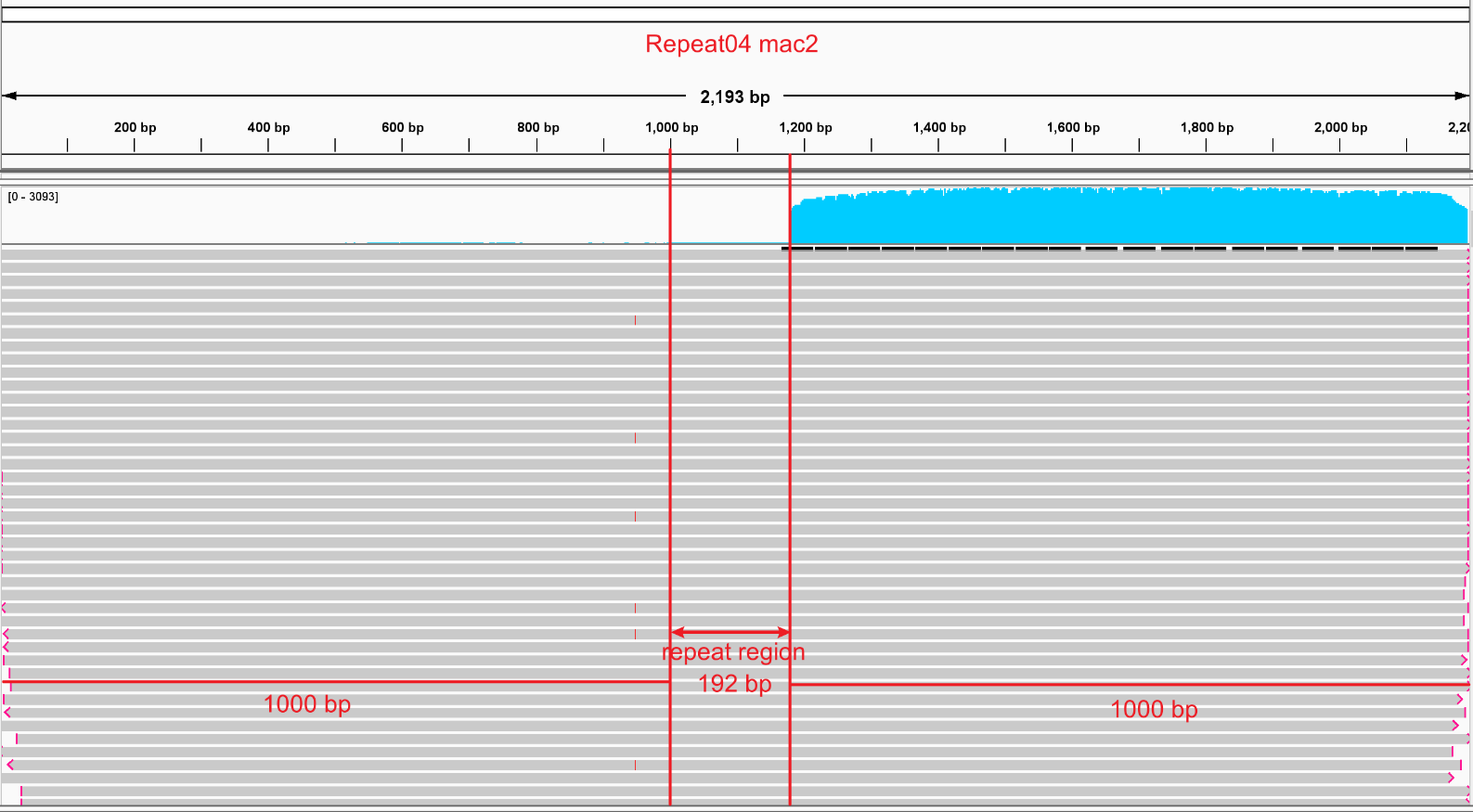


G Mapping of long reads to the minor configuration 1 (Mic1) of the DBS02 affiliated with the R04.


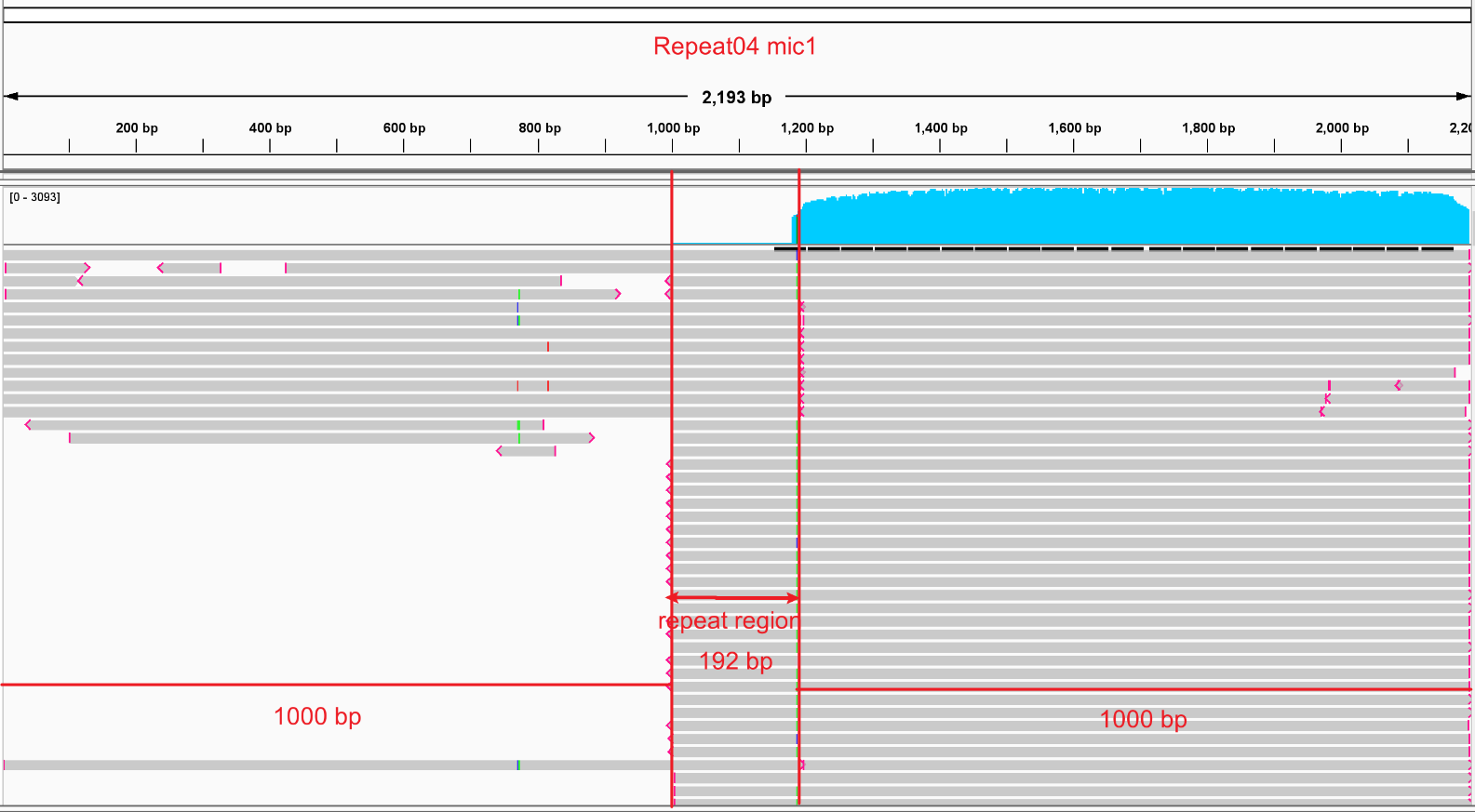


H Mapping of long reads to the minor configuration 1 (Mic1) of the DBS02 affiliated with the R04.


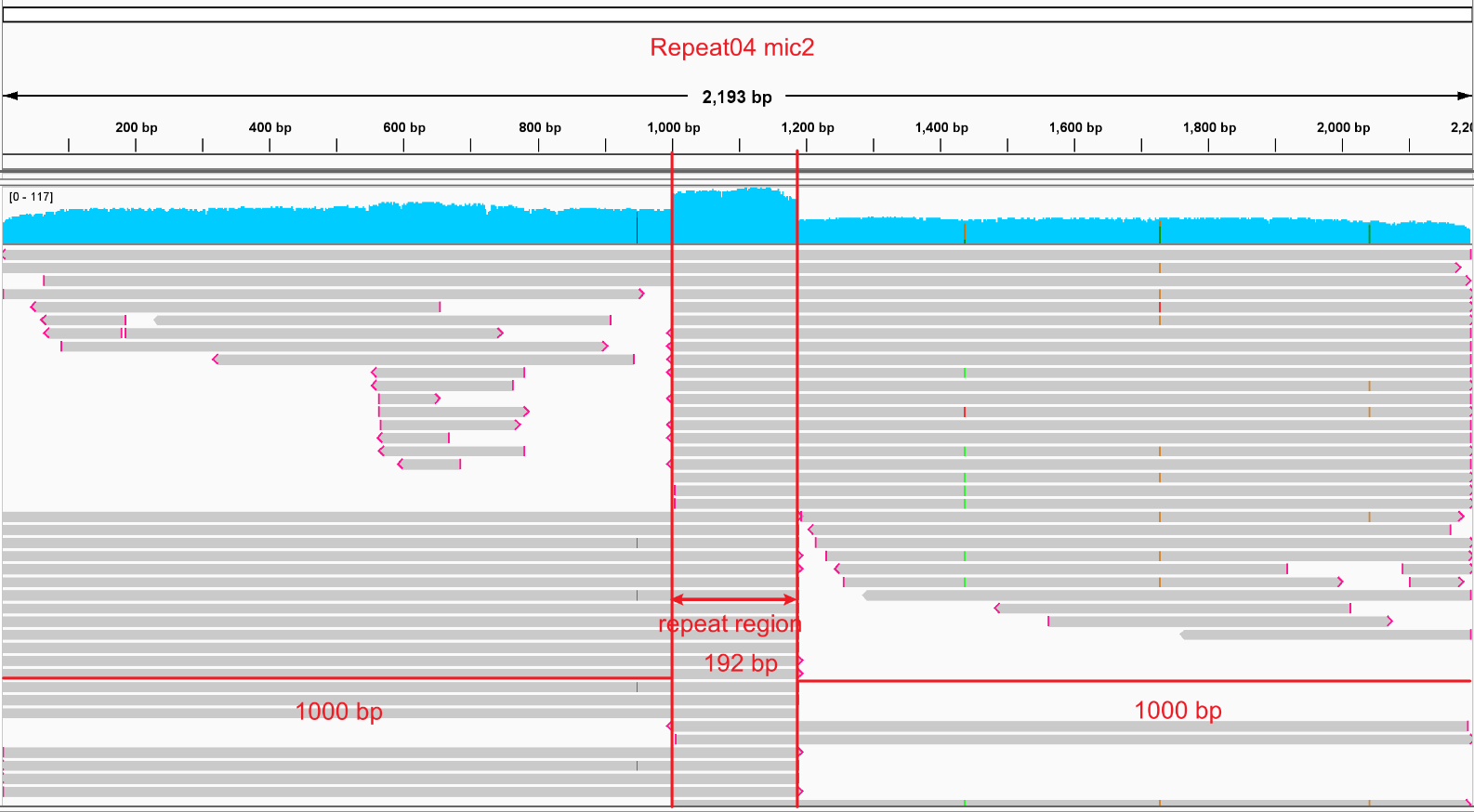


I Mapping of long reads to the major configuration 1 (Mac1) of the DBS03 affiliated with the R07.


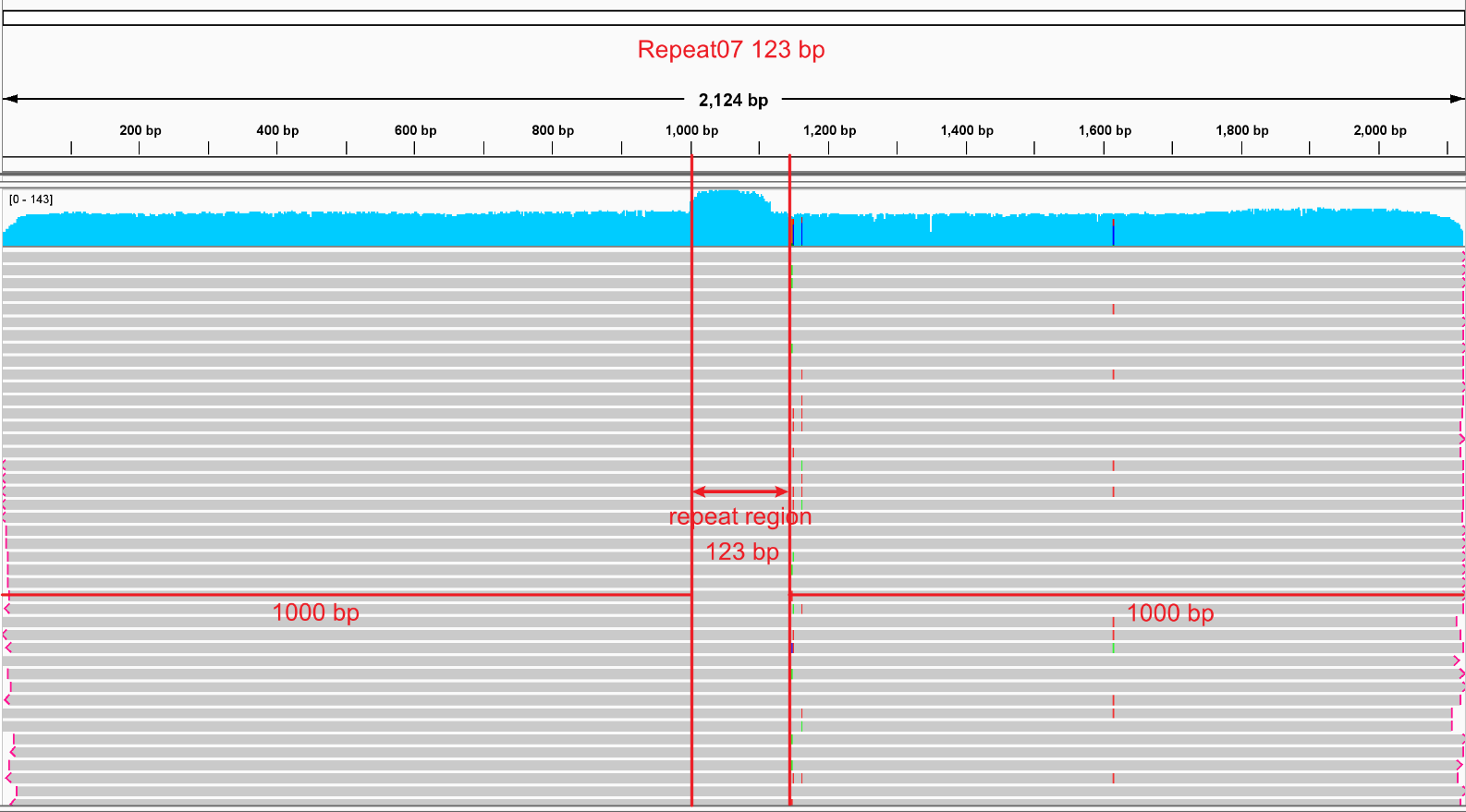


J Mapping of long reads to the major configuration 2 (Mac2) of the DBS03 affiliated with the R07.


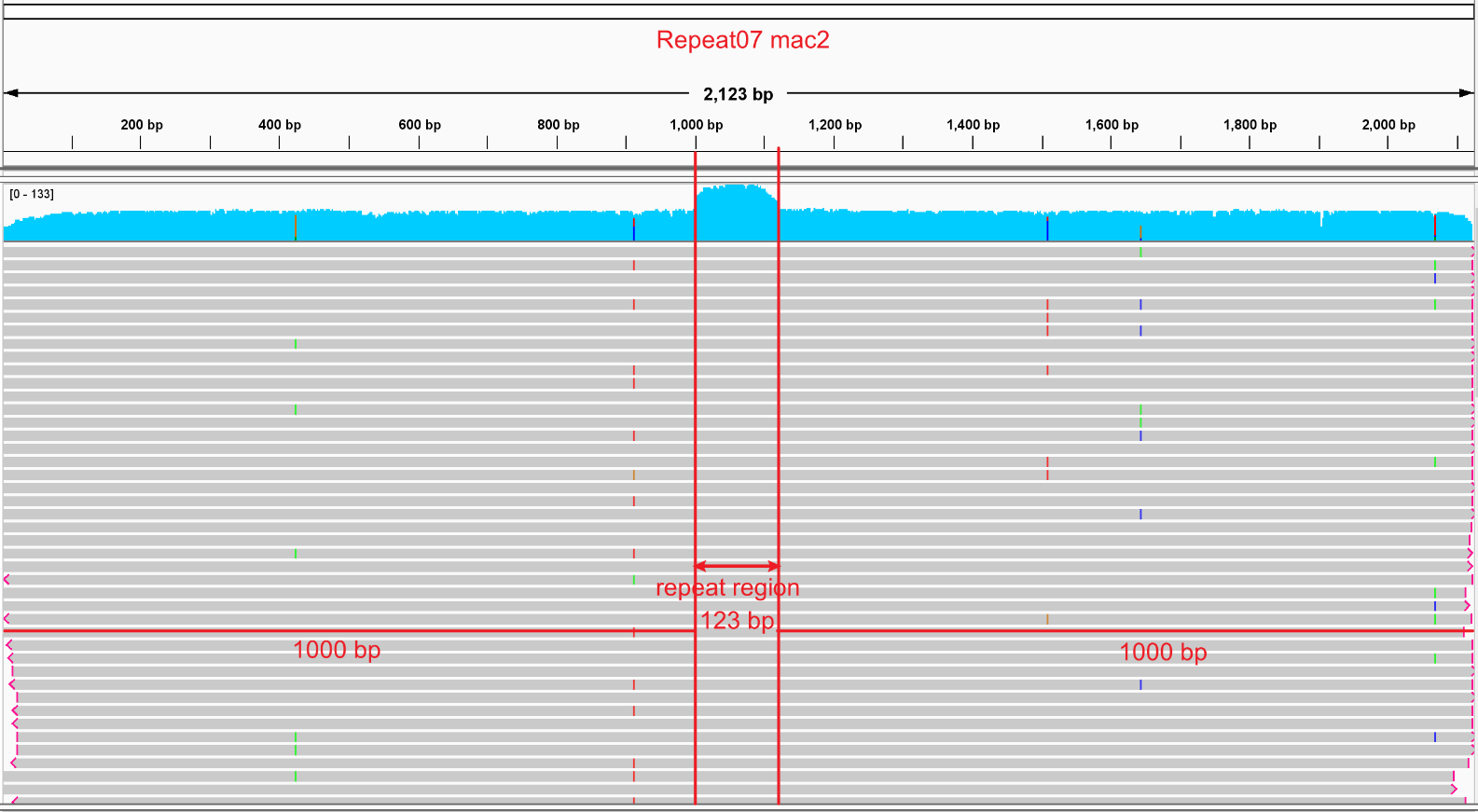


K Mapping of long reads to the minor configuration 1 (Mic1) of the DBS03 affiliated with the R07.


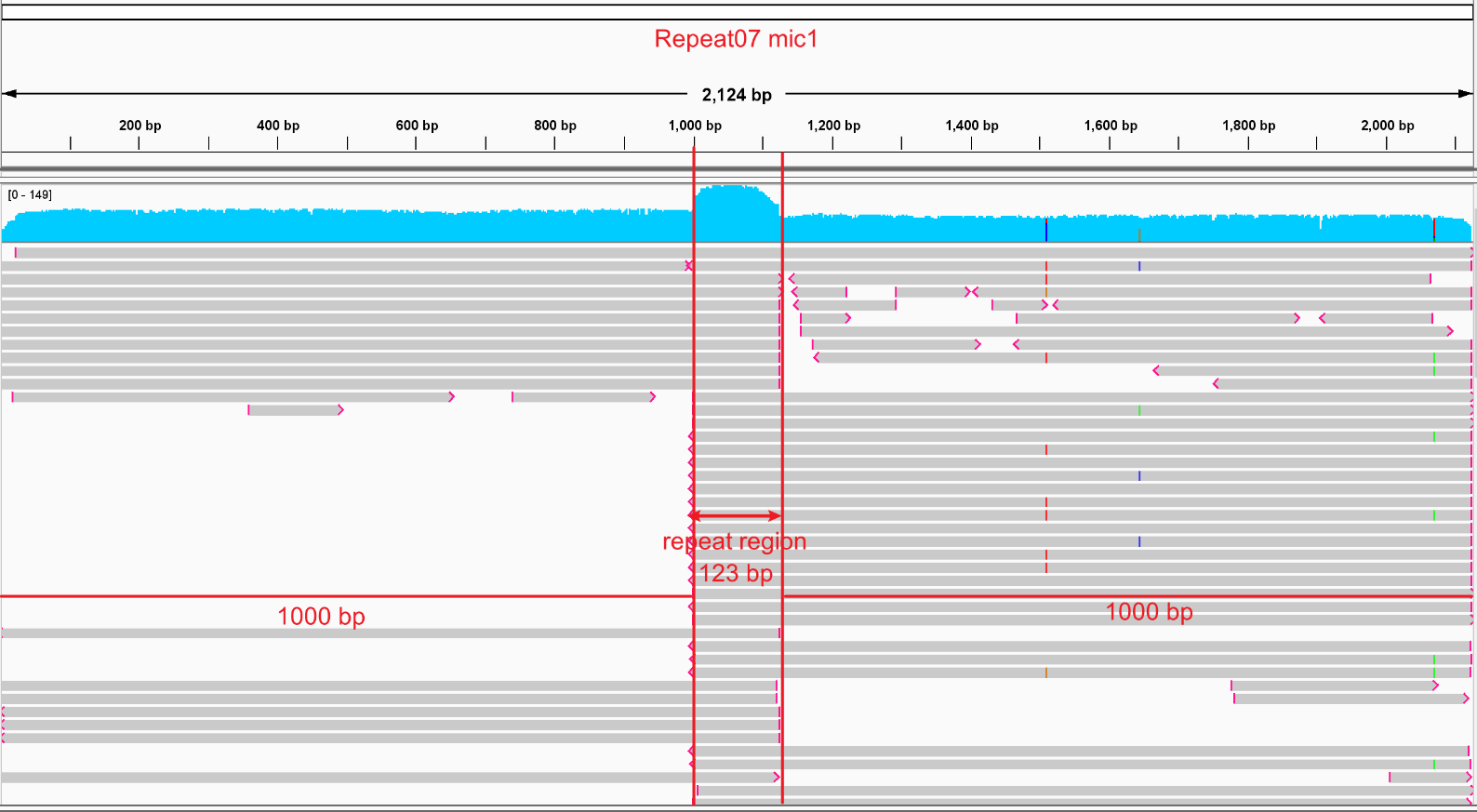


L Mapping of long reads to the minor configuration 2 (Mic2) of the DBS03 affiliated with the R07.


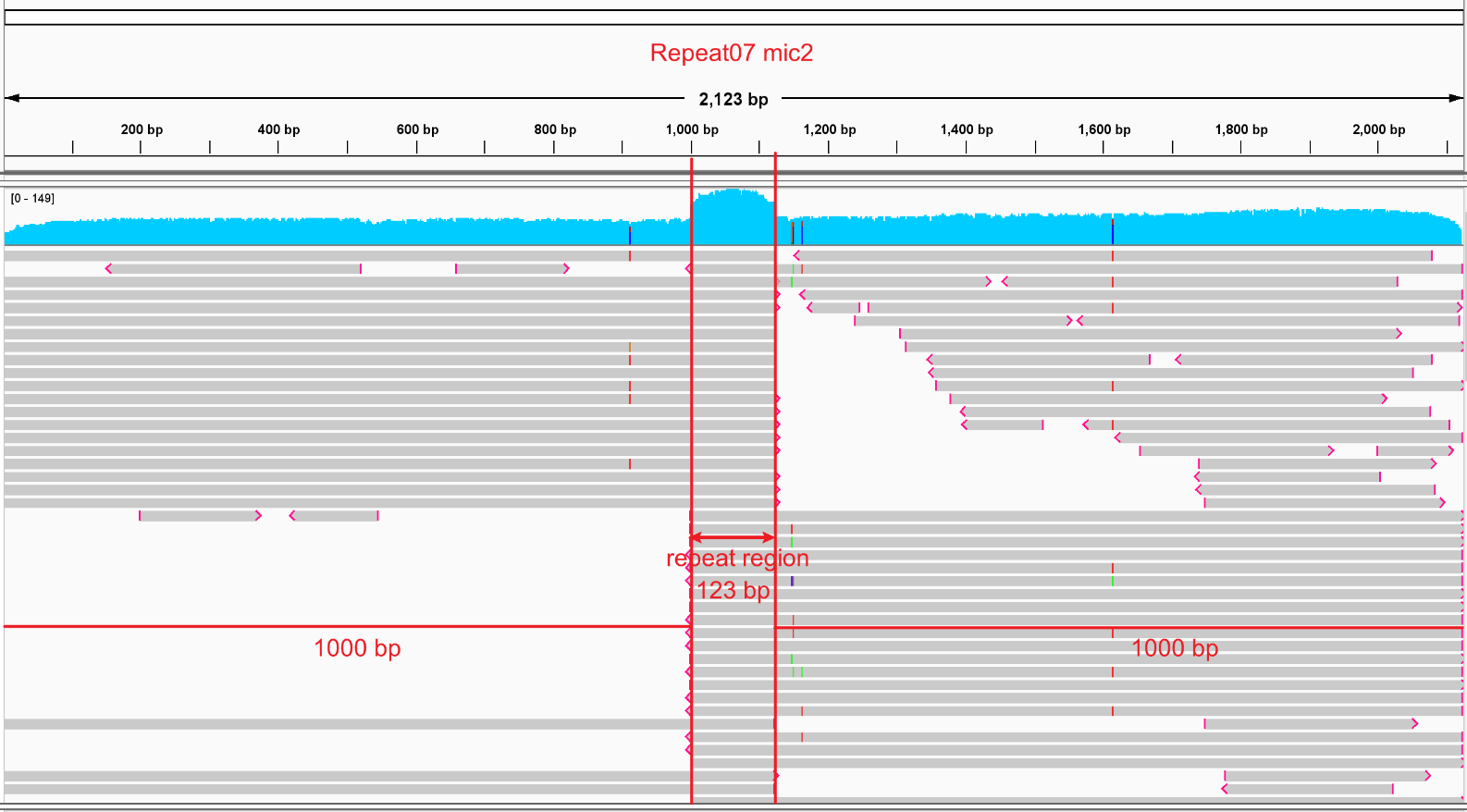


Fig. S2 Results of Nanopore and Illumina reads mapped to the mitochondrial chromosomes 1 (MC1) and 2 (MC2) of *S. splendens*. Panels A and B show the mapping results of Nanopore reads to MC1 and MC2, respectively. Panels C and D show the mapping results of all Illumina reads to the MC1 and MC2, respectively. The X-axis shows the nucleotide position and the Y-axis shows the corresponding coverage depth.


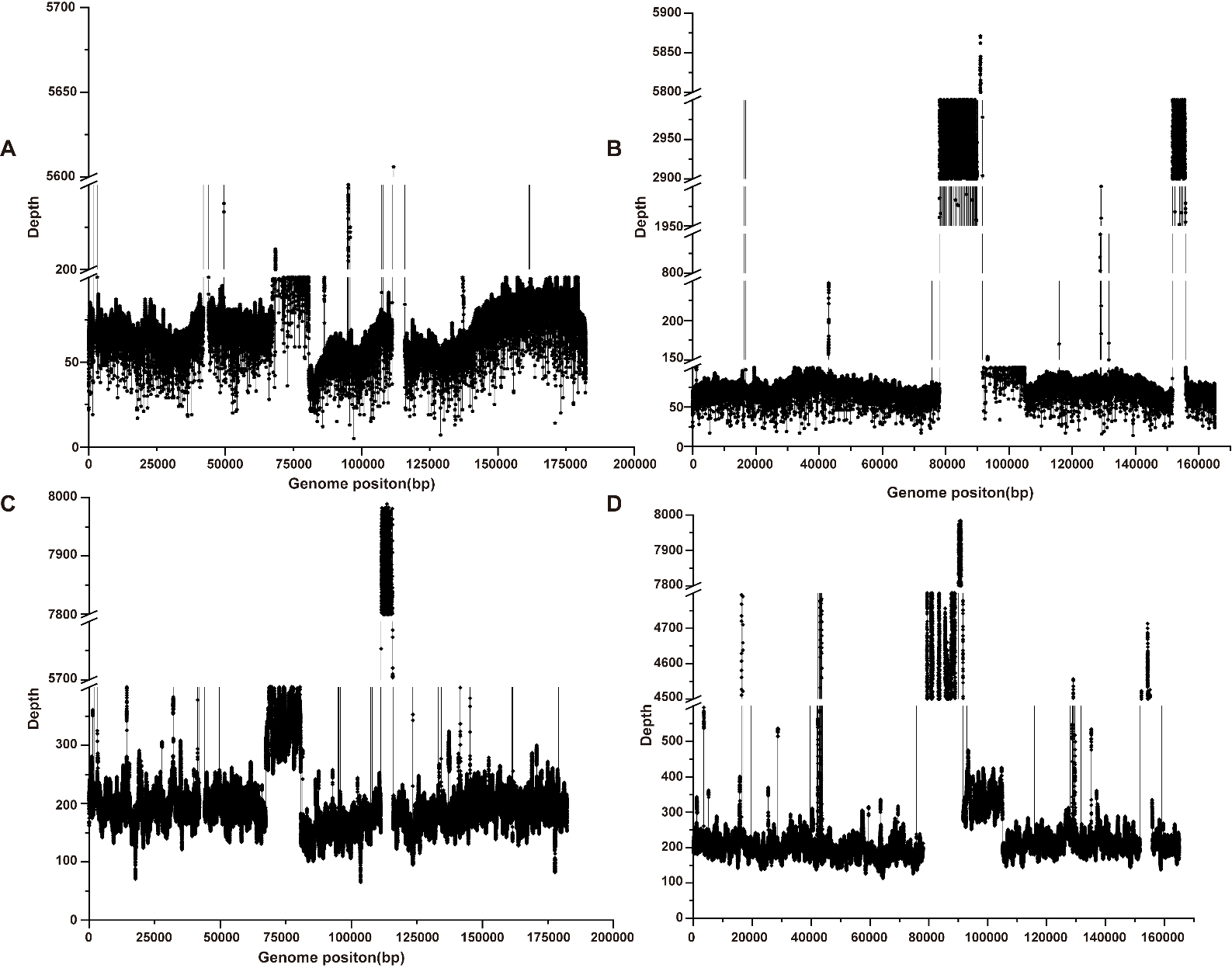


Fig. S3 Validation of the presence of the four configurations c1-4 of R04 and 07, respectively. PCR primers were designed based on the sequences corresponding to the four configurations. The genomic DNAs were then amplified by PCR and the PCR products were subject to Sanger sequencing. The sequencing chromatograms, the Sanger sequencing results (labeled with “PCR” and configuration number), the expected sequences (labeled with repeat id and configuration number), and the consensus sequences are shown at the top of the chromatograms and below the chromatograms. Panels A-I correspond to the repetitive sequences R04 and 07. The sequences of R04 and R07 were highlighted in bold red.

A comparison of the sequences obtained from PCR amplification and Sanger sequencing, and the expected sequence for R04-C1.


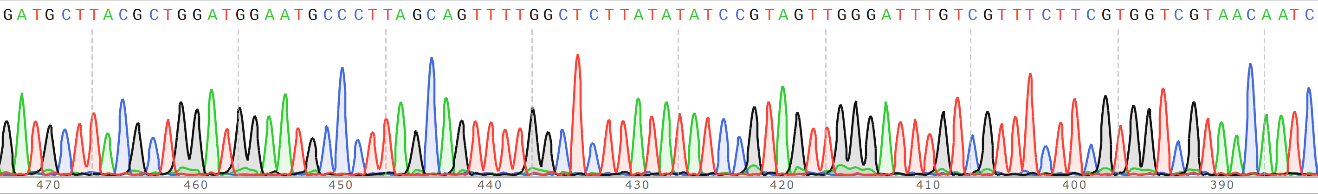

PCR_C1 : gatgcttacgctggatggaatgcccttagcagttttggctcttatatatccgtagttgggatttgtcgtttcttcgtggtcgtaacaatc : 90
R04_C1 : gatgcttacgctggatggaatgcccttagcagttttggctcttatatatccgtagttgggatttgtcgtttcttcgtggtcgtaacaatc : 90
 GATGCTTACGCTGGATGGAATGCCCTTAGCAGTTTTGGCTCTTATATATCCGTAGTTGGGATTTGTCGTTTCTTCGTGGTCGTAACAATC


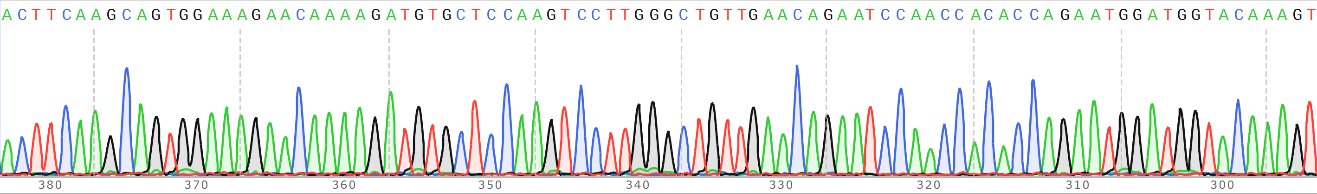

PCR_C1 : acttcaagcagtggaaagaacaaaagatgtgctccaagtccttgggctgttgaacagaatccaaccacaccagaatggatggtacaaagt : 180
R04_C1 : acttcaagcagtggaaagaacaaaagatgtgctccaagtccttgggctgttgaacagaatccaaccacaccagaatggatggtacaaagt : 180
 ACTTCAAGCAGTGGAAAGAACAAAAGATGTGCTCCAAGTCCTTGGGCTGTTGAACAGAATCCAA**CCACACCAGAATGGATGGTACAAAGT**


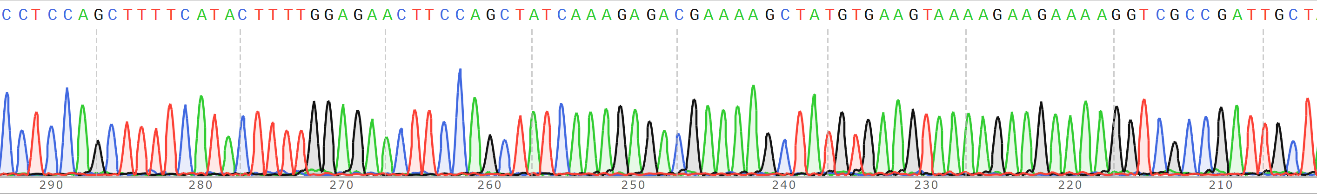

PCR_C1 : cctccagcttttcatacttttggagaacttccagctatcaaagagacgaaaagctatgtgaagtaaaagaagaaaaggtcgccgattgct : 270
R04_C1 : cctccagcttttcatacttttggagaacttccagctatcaaagagacgaaaagctatgtgaagtaaaagaagaaaaggtcgccgattgct : 270
 **CCTCCAGCTTTTCATACTTTTGGAGAACTTCCAGCTATCAAAGAGACGAAAAGCTATGTGAAGTAAAAGAAGAAAAGGTCGCCGATTGCT**


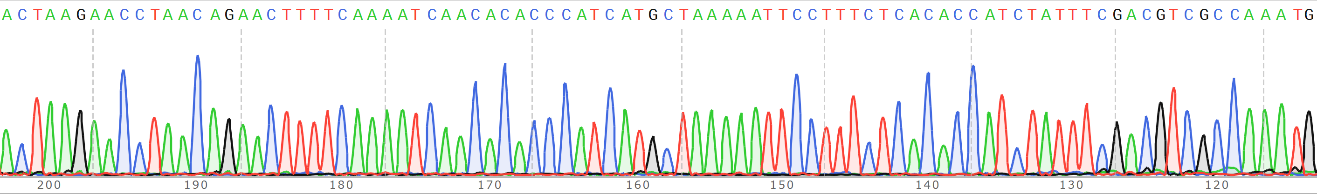

PCR_C1 : actaagaacctaacagaacttttcaaaatcaacacacccatcatgctaaaaattcctttctcacaccatctatttcgacgtcgccaaatg : 360
R04_C1 : actaagaacctaacagaacttttcaaaatcaacacacccatcatgctaaaaattcctttctcacaccatctatttcgacgtcgccaaatg : 360
 **ACTAAGAACCTAACAGAACTTTTCAAAATCAACACACCCATCATGCTAAAAATTCCTTTCTCACACCATCTATTTC**GACGTCGCCAAATG


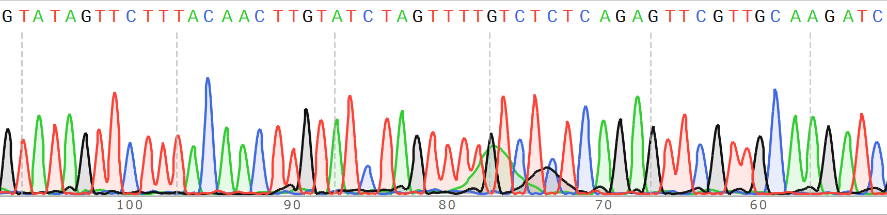

PCR_C1 : gtatggtatagttctttacaacttgtatctagttttgtctctcagagttcgttgcaagatc : 421
R04_C1 : gtatggtatagttctttacaacttgtatctagttttgtctctcagagttcgttgcaagatc : 421
 GTATGGTATAGTTCTTTACAACTTGTATCTAGTTTTGTCTCTCAGAGTTCGTTGCAAGATC

B comparison of the sequence obtained from PCR amplification and Sanger sequencing, and the expected sequence for R04-C2.


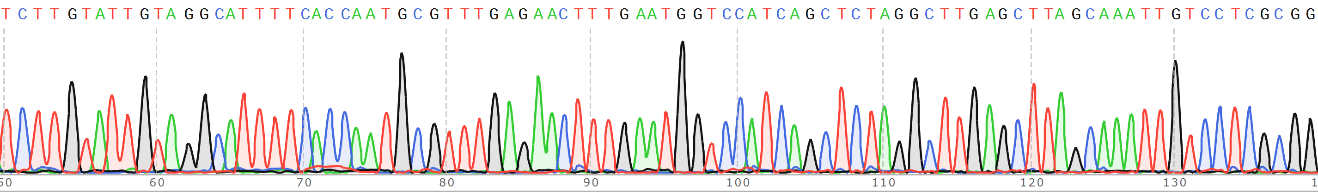

PCR_C2 : tcttgtattgtaggcattttcaccaatgcgtttgagaactttgaatggtccatcagctctaggcttgagcttagcaaattgtcctcgcgg : 90
R04_C2 : tcttgtattgtaggcattttcaccaatgcgtttgagaactttgaatggtccatcagctctaggcttgagcttagcaaattgtcctcgcgg : 90
 TCTTGTATTGTAGGCATTTTCACCAATGCGTTTGAGAACTTTGAATGGTCCATCAGCTCTAGGCTTGAGCTTAGCAAATTGTCCTCGCGG


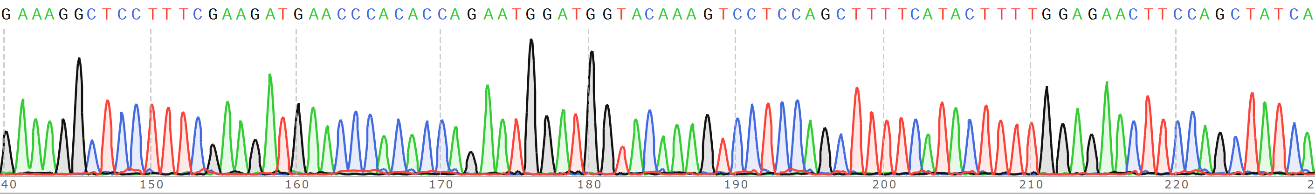

PCR_C2 : gaaaggctcctttcgaagatgaacccacaccagaatggatggtacaaagtcctccagcttttcatacttttggagaacttccagctatca : 180
R04_C2 : gaaaggctcctttcgaagatgaacccacaccagaatggatggtacaaagtcctccagcttttcatacttttggagaacttccagctatca : 180
 GAAAGGCTCCTTTCGAAGATGAAC**CCACACCAGAATGGATGGTACAAAGTCCTCCAGCTTTTCATACTTTTGGAGAACTTCCAGCTATCA**


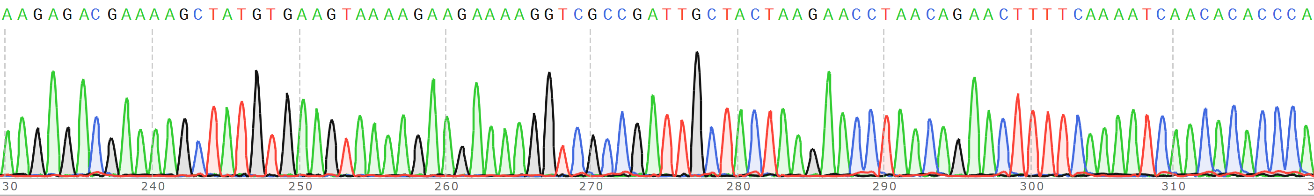

PCR_C2 : aagagacgaaaagctatgtgaagtaaaagaagaaaaggtcgccgattgctactaagaacctaacagaacttttcaaaatcaacacaccca : 270
R04_C2 : aagagacgaaaagctatgtgaagtaaaagaagaaaaggtcgccgattgctactaagaacctaacagaacttttcaaaatcaacacaccca : 270
 **AAGAGACGAAAAGCTATGTGAAGTAAAAGAAGAAAAGGTCGCCGATTGCTACTAAGAACCTAACAGAACTTTTCAAAATCAACACACCCA**


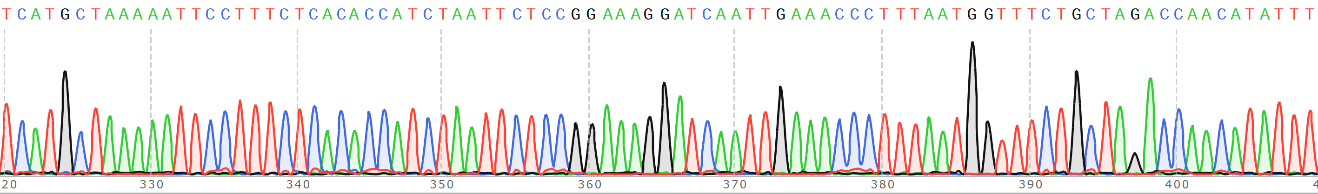

PCR_C2 : tcatgctaaaaattcctttctcacaccatctaattctccggaaaggatcaattgaaaccctttaatggtttctgctagaccaacatattt : 360
R04_C2 : tcatgctaaaaattcctttctcacaccatctaattctccggaaaggatcaattgaaaccctttaatggtttctgctagaccaacatattt : 360
 **TCATGCTAAAAATTCCTTTCTCACACCATCTAATTC**TCCGGAAAGGATCAATTGAAACCCTTTAATGGTTTCTGCTAGACCAACATATTT


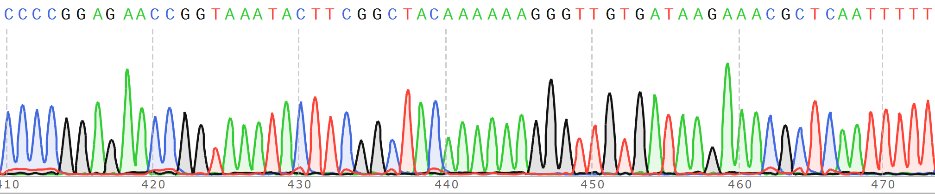

PCR_C2 : ccccggagaaccggtaaatacttcggctacaaaaaagggttgtgataagaaacgctcaattttt : 424
R04_C2 : ccccggagaaccggtaaatacttcggctacaaaaaagggttgtgataagaaacgctcaattttt : 424
 CCCCGGAGAACCGGTAAATACTTCGGCTACAAAAAAGGGTTGTGATAAGAAACGCTCAATTTTT

C comparison of the sequence obtained from PCR amplification and Sanger sequencing, and the expected sequence for R04-C3.


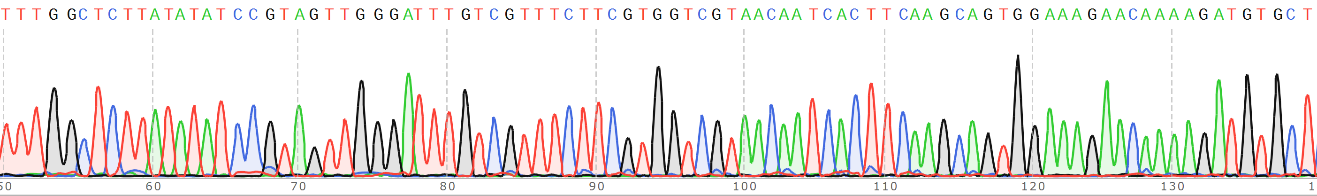

PCR_C3 : tttggctcttatatatccgtagttgggatttgtcgtttcttcgtggtcgtaacaatcacttcaagcagtggaaagaacaaaagatgtgct : 90
R04_C3 : tttggctcttatatatccgtagttgggatttgtcgtttcttcgtggtcgtaacaatcacttcaagcagtggaaagaacaaaagatgtgct : 90
 TTTGGCTCTTATATATCCGTAGTTGGGATTTGTCGTTTCTTCGTGGTCGTAACAATCACTTCAAGCAGTGGAAAGAACAAAAGATGTGCT


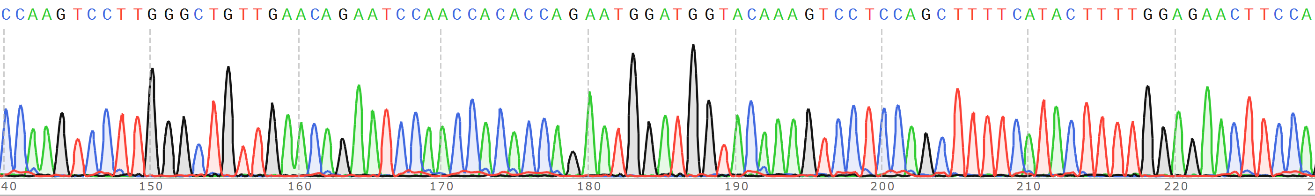

PCR_C3 : ccaagtccttgggctgttgaacagaatccaaccacaccagaatggatggtacaaagtcctccagcttttcatacttttggagaacttcca : 180
R04_C3 : ccaagtccttgggctgttgaacagaatccaaccacaccagaatggatggtacaaagtcctccagcttttcatacttttggagaacttcca : 180
 CCAAGTCCTTGGGCTGTTGAACAGAATCCAA**CCACACCAGAATGGATGGTACAAAGTCCTCCAGCTTTTCATACTTTTGGAGAACTTCCA**


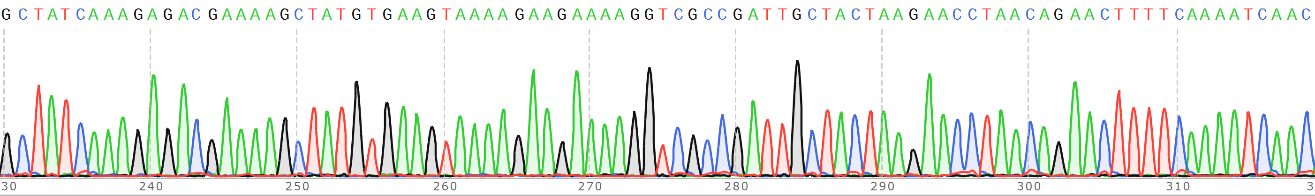

PCR_C3 : gctatcaaagagacgaaaagctatgtgaagtaaaagaagaaaaggtcgccgattgctactaagaacctaacagaacttttcaaaatcaac : 270
R04_C3 : gctatcaaagagacgaaaagctatgtgaagtaaaagaagaaaaggtcgccgattgctactaagaacctaacagaacttttcaaaatcaac : 270
 **GCTATCAAAGAGACGAAAAGCTATGTGAAGTAAAAGAAGAAAAGGTCGCCGATTGCTACTAAGAACCTAACAGAACTTTTCAAAATCAAC**


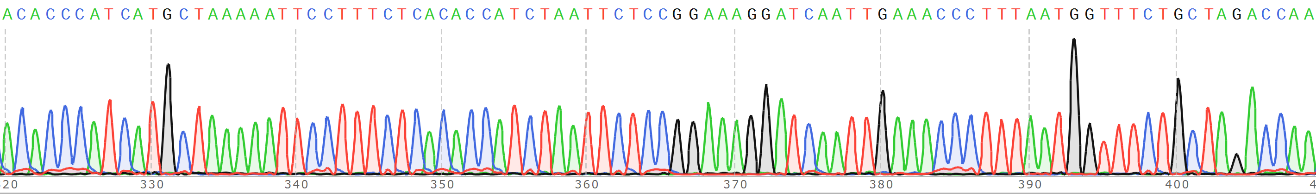

PCR_C3 : acacccatcatgctaaaaattcctttctcacaccatctaattctccggaaaggatcaattgaaaccctttaatggtttctgctagaccaa : 360
R04_C3 : acacccatcatgctaaaaattcctttctcacaccatctatttctccggaaaggatcaattgaaaccctttaatggtttctgctagaccaa : 360
 **ACACCCATCATGCTAAAAATTCCTTTCTCACACCATCTA TTC**TCCGGAAAGGATCAATTGAAACCCTTTAATGGTTTCTGCTAGACCAA


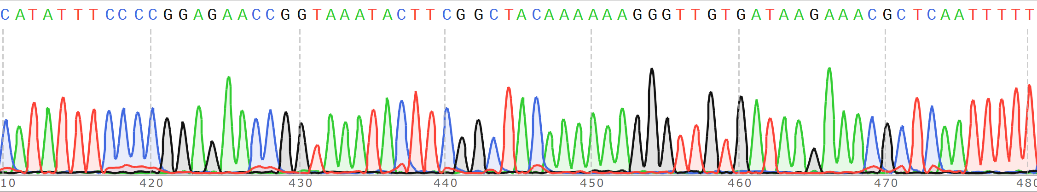

PCR_C3 : catatttccccggagaaccggtaaatacttcggctacaaaaaagggttgtgataagaaacgctcaattttt : 431
R04_C3 : catatttccccggagaaccggtaaatacttcggctacaaaaaagggttgtgataagaaacgctcaattttt : 431
 CATATTTCCCCGGAGAACCGGTAAATACTTCGGCTACAAAAAAGGGTTGTGATAAGAAACGCTCAATTTTT

D comparison of the sequence obtained from PCR amplification and Sanger sequencing, and the expected sequence for R01-C4.


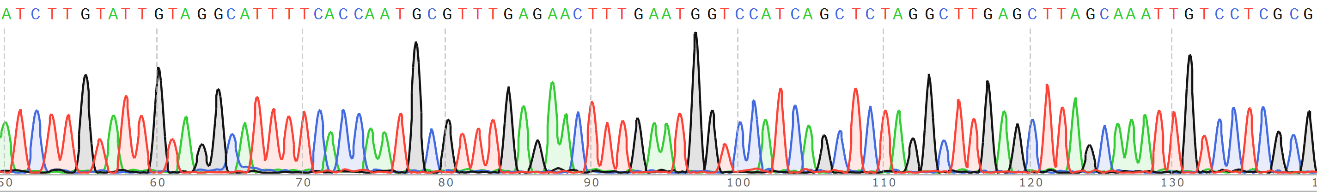

PCR_C4 : atcttgtattgtaggcattttcaccaatgcgtttgagaactttgaatggtccatcagctctaggcttgagcttagcaaattgtcctcgcg : 90
R04_C4 : atcttgtattgtaggcattttcaccaatgcgtttgagaactttgaatggtccatcagctctaggcttgagcttagcaaattgtcctcgcg : 90
 ATCTTGTATTGTAGGCATTTTCACCAATGCGTTTGAGAACTTTGAATGGTCCATCAGCTCTAGGCTTGAGCTTAGCAAATTGTCCTCGCG


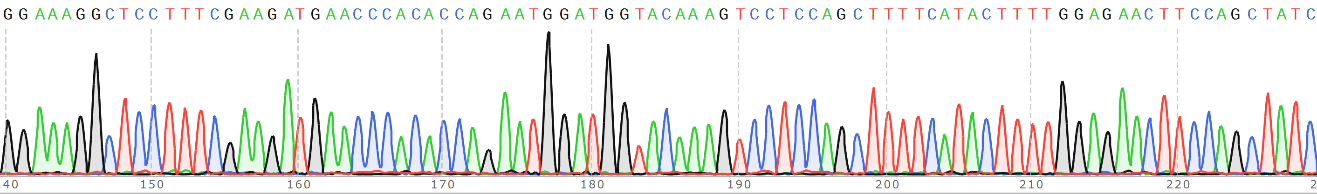

PCR_C4 : ggaaaggctcctttcgaagatgaacccacaccagaatggatggtacaaagtcctccagcttttcatacttttggagaacttccagctatc : 180
R04_C4 : ggaaaggctcctttcgaagatgaacccacaccagaatggatggtacaaagtcctccagcttttcatacttttggagaacttccagctatc : 180
 GGAAAGGCTCCTTTCGAAGATGAAC**CCACACCAGAATGGATGGTACAAAGTCCTCCAGCTTTTCATACTTTTGGAGAACTTCCAGCTATC**


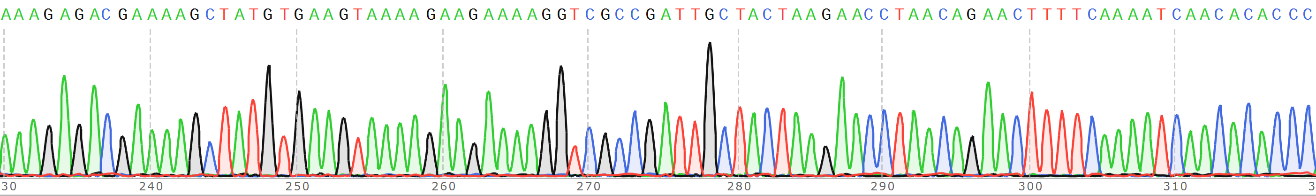

PCR_C4 : aaagagacgaaaagctatgtgaagtaaaagaagaaaaggtcgccgattgctactaagaacctaacagaacttttcaaaatcaacacaccc : 270
R04_C4 : aaagagacgaaaagctatgtgaagtaaaagaagaaaaggtcgccgattgctactaagaacctaacagaacttttcaaaatcaacacaccc : 270
 **AAAGAGACGAAAAGCTATGTGAAGTAAAAGAAGAAAAGGTCGCCGATTGCTACTAAGAACCTAACAGAACTTTTCAAAATCAACACACCC**


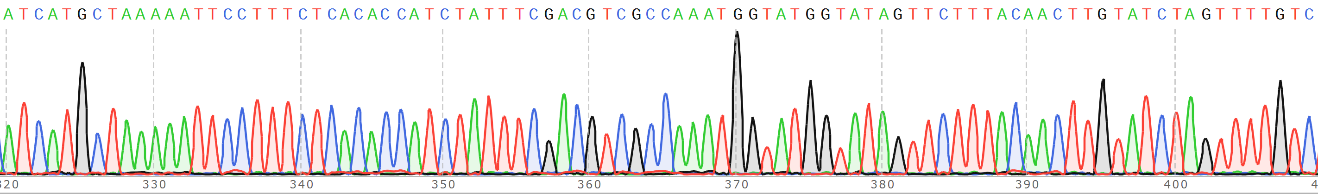

PCR_C4 : atcatgctaaaaattcctttctcacaccatctatttcgacgtcgccaaatggtatggtatagttctttacaacttgtatctagttttgtc : 360
R04_C4 : atcatgctaaaaattcctttctcacaccatctatttcgacgtcgccaaatggtatggtatagttctttacaacttgtatctagttttgtc : 360
 **ATCATGCTAAAAATTCCTTTCTCACACCATCTATTTC**GACGTCGCCAAATGGTATGGTATAGTTCTTTACAACTTGTATCTAGTTTTGTC


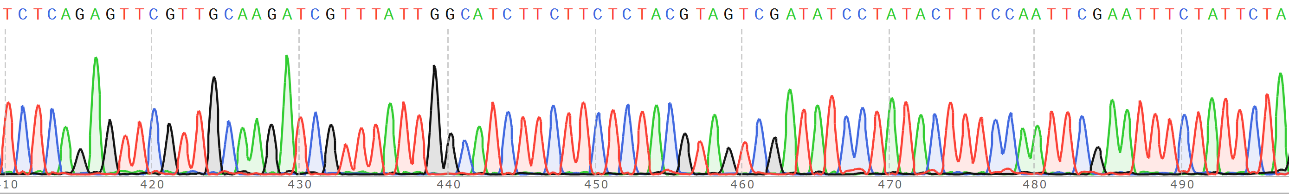

PCR_C4 : tctcagagttcgttgcaagatcgtttattggcatcttcttctctacgtagtcgatatcctatactttccaattcgaatttctattcta : 448
R04_C4 : tctcagagttcgttgcaagatcgtttattggcatcttcttctctacgtagtcgatatcctatactttccaattcgaatttctattcta : 448
 TCTCAGAGTTCGTTGCAAGATCGTTTATTGGCATCTTCTTCTCTACGTAGTCGATATCCTATACTTTCCAATTCGAATTTCTATTCTA

E comparison of the sequence obtained from PCR amplification and Sanger sequencing, and the expected sequence for R07-C1.


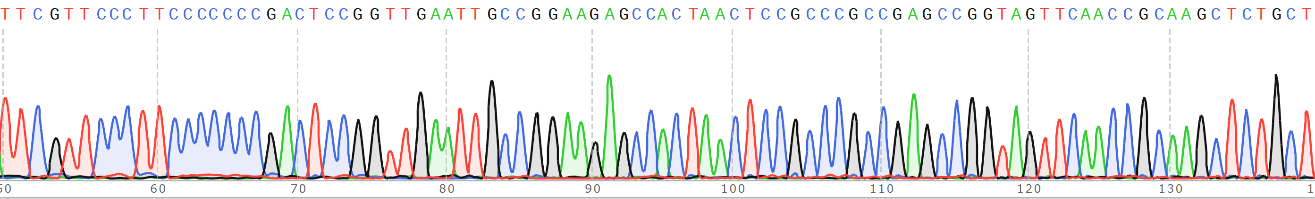

PCR_C1 : ttcgttcccttcccccccgactccggttgaattgccggaagagccactaactccgcccgccgagccggtagttcaaccgcaagctctgct : 90
R07_c1 : ttcgttcccttcccccccgactccggttgaattgccggaagagccactaactccgcccgccgagccggtagttcaaccgcaagctctgct : 90
 TTCGTTCCCTTCCCCCCCGACTCCGGTTGAATTGCCGGAAGAGCCACTAACTCCGCCCGCCGAGCCGGTAGTTCAACCGCAAGCTCTGCT


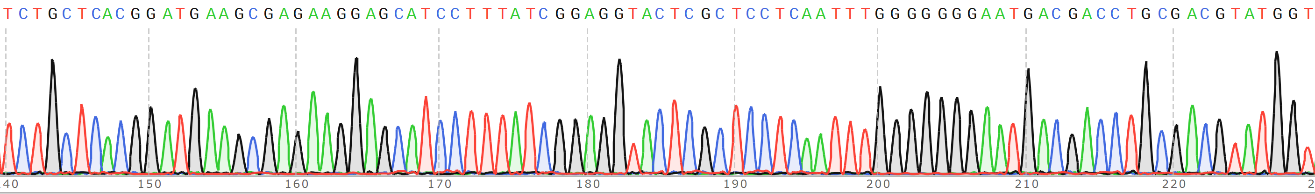

PCR_C1 : tctgctcacggatgaagcgagaaggagcatcctttatcggaggtactcgctcctcaatttgggggggaatgacgacctgcgacgtatggt : 180
R07_c1 : tctgctcacggatgaagcgagaaggagcatcctttatcggaggtactcgctcctcaatttgggggggaatgacgacctgcgacgtatggt : 180
 TCTGCTCACGGATGAAGCGAGAAGG**AGCATCCTTTATCGGAGGTACTCGCTCCTCAATTTGGGGGGGAATGACGACCTGCGACGTATGGT**


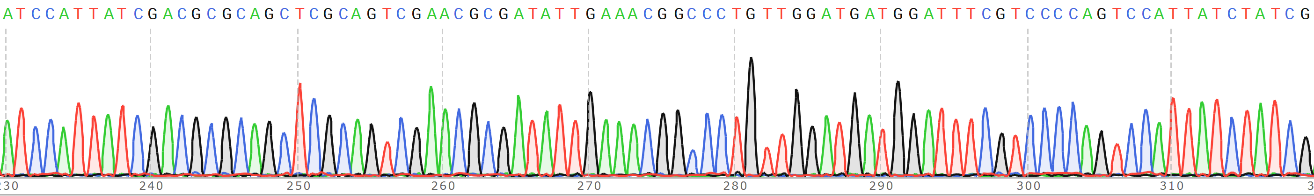

PCR_C1 : atccattatcgacgcgcagctcgcagtcgaacgcgatattgaaacggccctgttggatgatggatttcgtccccagtccattatctatcg : 270
R07_c1 : atccattatcgacgcgcagctcgcagtcgaacgcgatattgaaacggccctgttggatgatggatttcgtccccagtccattatctatcg : 270
 **ATCCATTATCGACGCGCAGCTCGCAGTCGAACGCGATATTGAAACGGCCCTGTTGGATGATGGATTTCGTCCCCAGTCCATTATCTATCG**


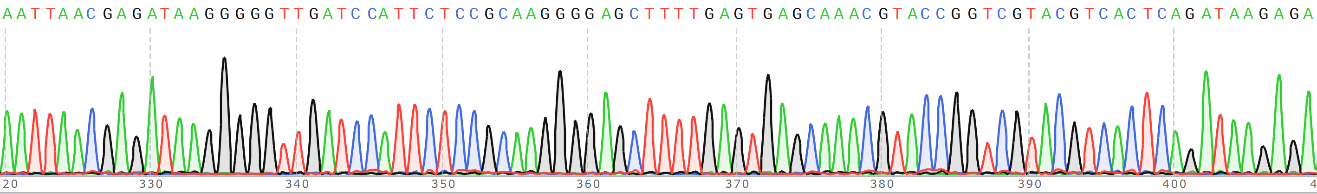

PCR_C1 : aattaacgagataagggggttgatccattctccgcaaggggagcttttgagtgagcaaacgtaccggtcgtacgtcactcagataagaga : 360
R07_c1 : aattaacgagataagggggttgatccattctccgcaaggggagcttttgagtgagcaaacgtaccggtcgtacgtcactcagataagaga : 360
 **AATTAACGAGATAAGGGGGTTGATCCATTCTCCGCAAGGGGAGCTTTTGAGTGAGCAAACGTACCGGTCGTACGTCACTCAGATAAGAGA**


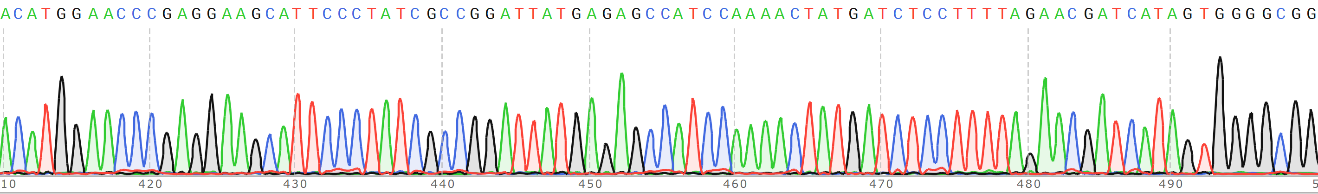

PCR_C1 : acatggaacccgaggaagcattccctatcgccggattatgagagccatccaaaactatgatctccttttagaacgatcatagtggggcgg : 450
R07_c1 : acatggaacccgaggaagcattccctatcgccggattatgagagccatccaaaactatgatctccttttagaacgatcatagtggggcgg : 450
 **ACATGGAACCCGAGGAAGCATTCCCTATCGCCGGATTATGAGAGCCATCCAAAACTATGATCTCCTTTTAGAACGATCATAGTGGGGCGG**


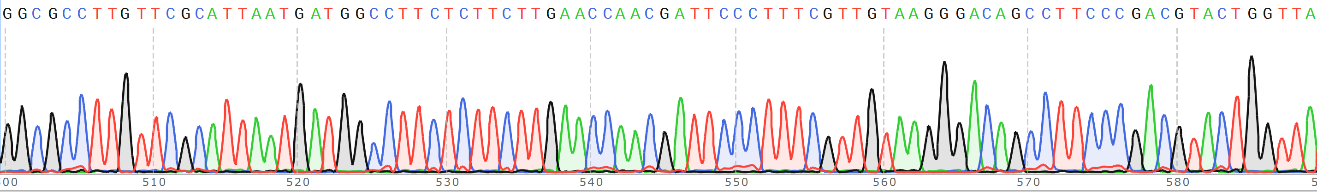

PCR_C1 : ggcgccttgttcgcattaatgatggccttctcttcttgaaccaacgattccctttcgttgtaagggacagccttcccgacgtactggtta : 540
R07_c1 : ggcgccttgttcgcattaatgatggccttctcttcttgaaccaacgattccctttcgttgtaagggacagccttcccgacgtactggtta : 540
 **GGCGCCTTGTTCGCATTAATGATGGCCTTCTCTTCT**TGAACCAACGATTCCCTTTCGTTGTAAGGGACAGCCTTCCCGACGTACTGGTTA


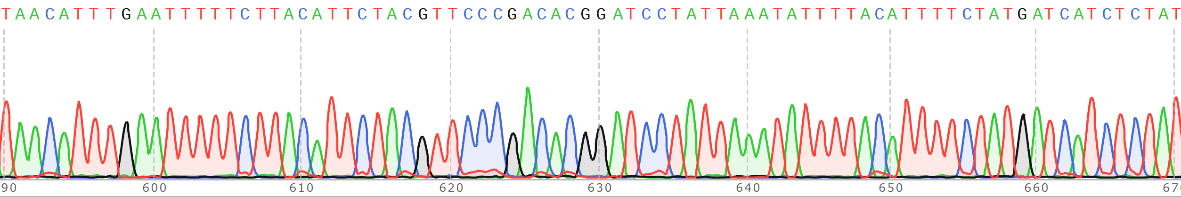

PCR_C1 : taacatttgaatttttcttacattctacgttcccgacacggatcctattaaatattttacattttctatgatcatctctat : 621
R07_c1 : taacatttgaatttttcttacattctacgttcccgacacggatcctattaaatattttacattttctatgatcatctctat : 621
 TAACATTTGAATTTTTCTTACATTCTACGTTCCCGACACGGATCCTATTAAATATTTTACATTTTCTATGATCATCTCTAT

F comparison of the sequence obtained from PCR amplification and Sanger sequencing, and the expected sequence for R07-C2.


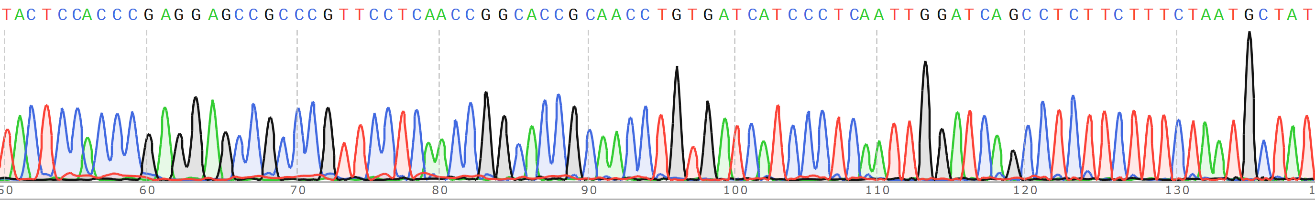

PCR_C2 : tactccacccgaggagccgcccgttcctcaaccggcaccgcaacctgtgatcatccctcaattggatcagcctcttctttctaatgctat : 90
R07_c2 : tactccacccgaggagccgcccgttcctcaaccggcaccgcaacctgtgatcatccctcaattggatcagcctcttctttctaatgctat : 90
 TACTCCACCCGAGGAGCCGCCCGTTCCTCAACCGGCACCGCAACCTGTGATCATCCCTCAATTGGATCAGCCTCTTCTTTCTAATGCTAT


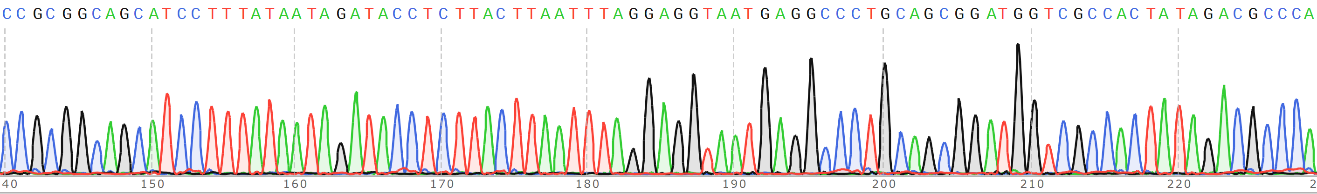

PCR_C2 : ccgcggcagcatcctttataatagatacctcttacttaatttaggaggtaatgaggccctgcagcggatggtcgccactatagacgccca : 180
R07_c2 : ccgcggcagcatcctttataatagatacctcttacttaatttaggaggtaatgaggccctgcagcggatggtcgccactatagacgccca : 180
 CCGCGGC**AGCATCCTTTATAATAGATACCTCTTACTTAATTTAGGAGGTAATGAGGCCCTGCAGCGGATGGTCGCCACTATAGACGCCCA**


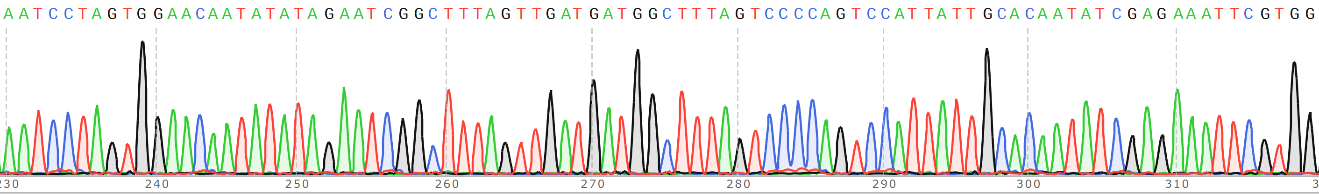

PCR_C2 : aatcctagtggaacaatatatagaatcggctttagttgatgatggctttagtccccagtccattattgcacaatatcgagaaattcgtgg : 270
R07_c2 : aatcctagtggaacaatatatagaatcggctttagttgatgatggctttagtccccagtccattattgcacaatatcgagaaattcgtgg : 270
 **AATCCTAGTGGAACAATATATAGAATCGGCTTTAGTTGATGATGGCTTTAGTCCCCAGTCCATTATTGCACAATATCGAGAAATTCGTGG**


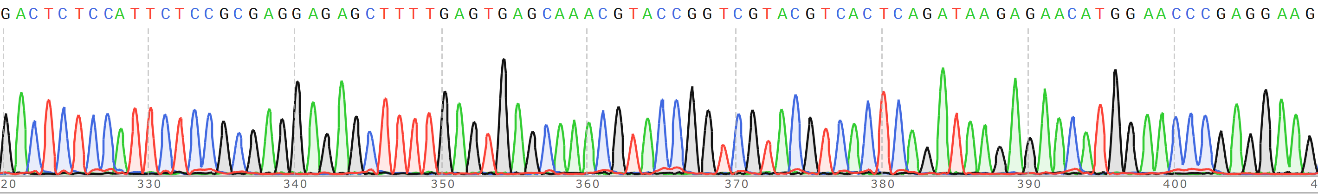

PCR_C2 : gactctccattctccgcgaggagagcttttgagtgagcaaacgtaccggtcgtacgtcactcagataagagaacatggaacccgaggaag : 360
R07_c2 : gactctccattctccgcgaggagagcttttgagtgagcaaacgtaccggtcgtacgtcactcagataagagaacatggaacccgaggaag : 360
 **GACTCTCCATTCTCCGCGAGGAGAGCTTTTGAGTGAGCAAACGTACCGGTCGTACGTCACTCAGATAAGAGAACATGGAACCCGAGGAAG**


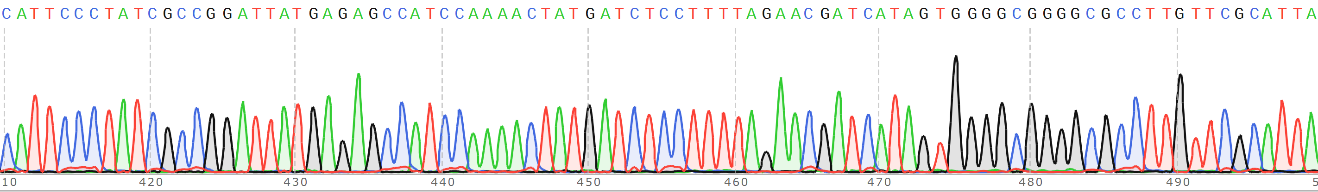

PCR_C2 : cattccctatcgccggattatgagagccatccaaaactatgatctccttttagaacgatcatagtggggcggggcgccttgttcgcatta : 450
R07_c2 : cattccctatcgccggattatgagagccatccaaaactatgatctccttttagaacgatcatagtggggcggggcgccttgttcgcatta : 450
 **CATTCCCTATCGCCGGATTATGAGAGCCATCCAAAACTATGATCTCCTTTTAGAACGATCATAGTGGGGCGGGGCGCCTTGTTCGCATTA**


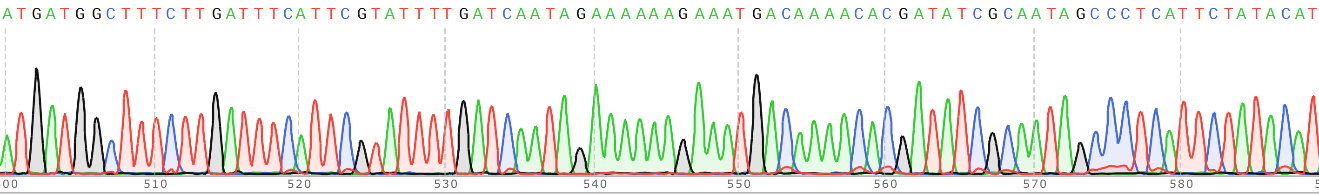

PCR_C2 : atgatggctttcttgatttcattcgtattttgatcaatagaaaaaagaaatgacaaaacacgatatcgcaatagccctcattctatacat : 540
R07_c2 : atgatggctttcttgatttcattcgtattttgatcaatagaaaaaagaaatgacaaaacacgatatcgcaatagccctcattctatacat : 540
 **ATGATGGCTTTCTTGATT**TCATTCGTATTTTGATCAATAGAAAAAAGAAATGACAAAACACGATATCGCAATAGCCCTCATTCTATACAT


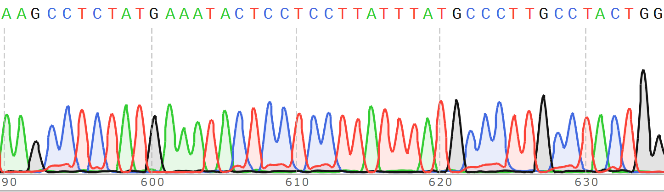

PCR_C2 : aagcctctatgaaatactcctccttatttatgcccttgcctactgg : 586
R07_c2 : aagcctctatgaaatactcctccttatttatgcccttgcctactgg : 586
 AAGCCTCTATGAAATACTCCTCCTTATTTATGCCCTTGCCTACTGG

G comparison of the sequence obtained from PCR amplification and Sanger sequencing, and the expected sequence for R07-C3.


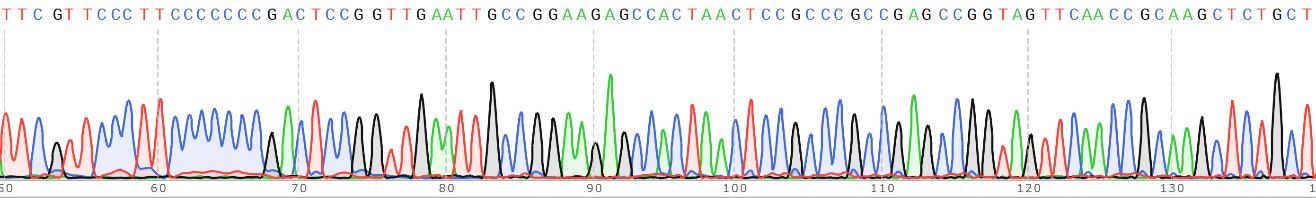

PCR_C3 : ttcgttcccttcccccccgactccggttgaattgccggaagagccactaactccgcccgccgagccggtagttcaaccgcaagctctgct : 90
R07_C3 : ttcgttcccttcccccccgactccggttgaattgccggaagagccactaactccgcccgccgagccggtagttcaaccgcaagctctgct : 90
 TTCGTTCCCTTCCCCCCCGACTCCGGTTGAATTGCCGGAAGAGCCACTAACTCCGCCCGCCGAGCCGGTAGTTCAACCGCAAGCTCTGCT


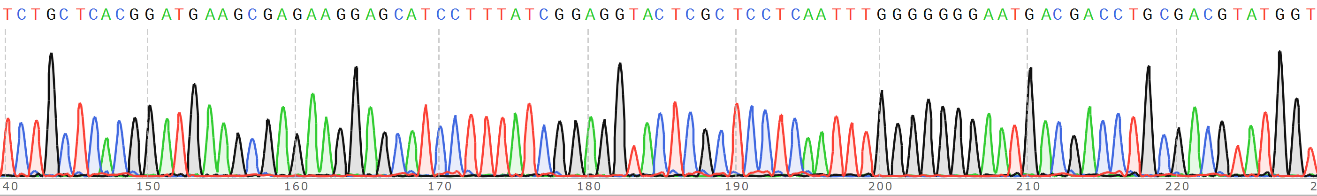

PCR_C3 : tctgctcacggatgaagcgagaaggagcatcctttatcggaggtactcgctcctcaatttgggggggaatgacgacctgcgacgtatggt : 180
R07_C3 : tctgctcacggatgaagcgagaaggagcatcctttatcggaggtactcgctcctcaatttgggggggaatgacgacctgcgacgtatggt : 180
 TCTGCTCACGGATGAAGCGAGAAGG**AGCATCCTTTATCGGAGGTACTCGCTCCTCAATTTGGGGGGGAATGACGACCTGCGACGTATGGT**


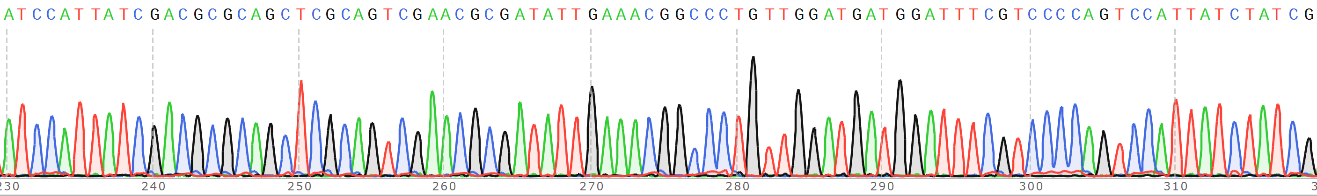

PCR_C3 : atccattatcgacgcgcagctcgcagtcgaacgcgatattgaaacggccctgttggatgatggatttcgtccccagtccattatctatcg : 270
R07_C3 : atccattatcgacgcgcagctcgcagtcgaacgcgatattgaaacggccctgttggatgatggatttcgtccccagtccattatctatcg : 270
 **ATCCATTATCGACGCGCAGCTCGCAGTCGAACGCGATATTGAAACGGCCCTGTTGGATGATGGATTTCGTCCCCAGTCCATTATCTATCG**


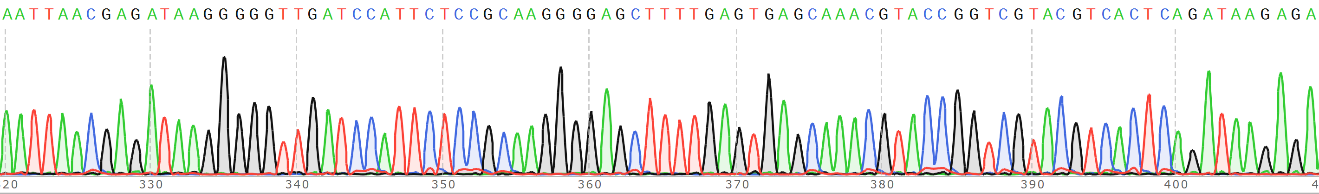

PCR_C3 : aattaacgagataagggggttgatccattctccgcaaggggagcttttgagtgagcaaacgtaccggtcgtacgtcactcagataagaga : 360
R07_C3 : aattaacgagataagggggttgatccattctccgcaaggggagcttttgagtgagcaaacgtaccggtcgtacgtcactcagataagaga : 360
 **AATTAACGAGATAAGGGGGTTGATCCATTCTCCGCAAGGGGAGCTTTTGAGTGAGCAAACGTACCGGTCGTACGTCACTCAGATAAGAGA**


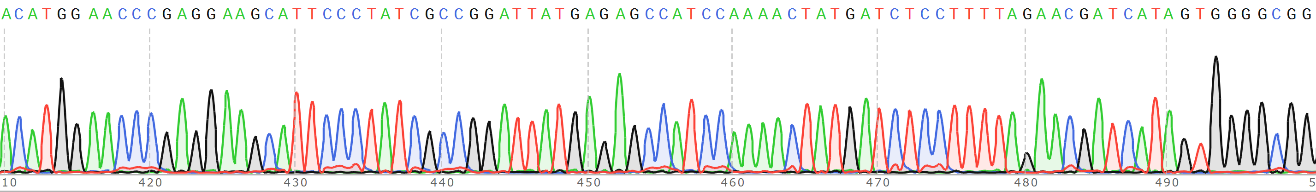

PCR_C3 : acatggaacccgaggaagcattccctatcgccggattatgagagccatccaaaactatgatctccttttagaacgatcatagtggggcgg : 450
R07_C3 : acatggaacccgaggaagcattccctatcgccggattatgagagccatccaaaactatgatctccttttagaacgatcatagtggggcgg : 450
 **ACATGGAACCCGAGGAAGCATTCCCTATCGCCGGATTATGAGAGCCATCCAAAACTATGATCTCCTTTTAGAACGATCATAGTGGGGCGG**


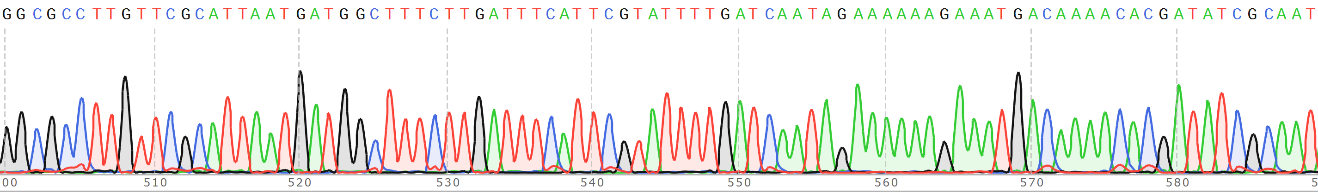

PCR_C3 : ggcgccttgttcgcattaatgatggctttcttgatttcattcgtattttgatcaatagaaaaaagaaatgacaaaacacgatatcgcaat : 540
R07_C3 : ggcgccttgttcgcattaatgatggccttcttgatttcattcgtattttgatcaatagaaaaaagaaatgacaaaacacgatatcgcaat : 540
 **GGCGCCTTGTTCGCATTAATGATGGC TTCTTGATT**TCATTCGTATTTTGATCAATAGAAAAAAGAAATGACAAAACACGATATCGCAAT


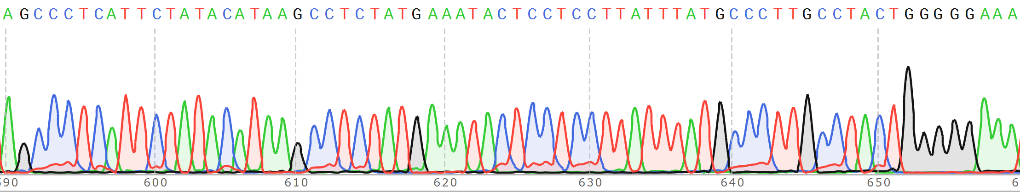

PCR_C3 : agccctcattctatacataagcctctatgaaatactcctccttatttatgcccttgcctactgggggaaa : 610
R07_C3 : agccctcattctatacataagcctctatgaaatactcctccttatttatgcccttgcctactgggggaaa : 610
 AGCCCTCATTCTATACATAAGCCTCTATGAAATACTCCTCCTTATTTATGCCCTTGCCTACTGGGGGAAA

H comparison of the sequence obtained from PCR amplification and Sanger sequencing, and the expected sequence for R07-C4.


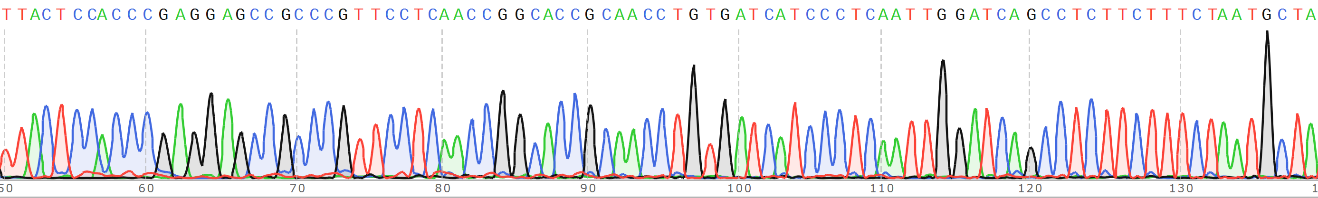

PCR_C4 : ttactccacccgaggagccgcccgttcctcaaccggcaccgcaacctgtgatcatccctcaattggatcagcctcttctttctaatgcta : 90
R07_C4 : ttactccacccgaggagccgcccgttcctcaaccggcaccgcaacctgtgatcatccctcaattggatcagcctcttctttctaatgcta : 90
 TTACTCCACCCGAGGAGCCGCCCGTTCCTCAACCGGCACCGCAACCTGTGATCATCCCTCAATTGGATCAGCCTCTTCTTTCTAATGCTA


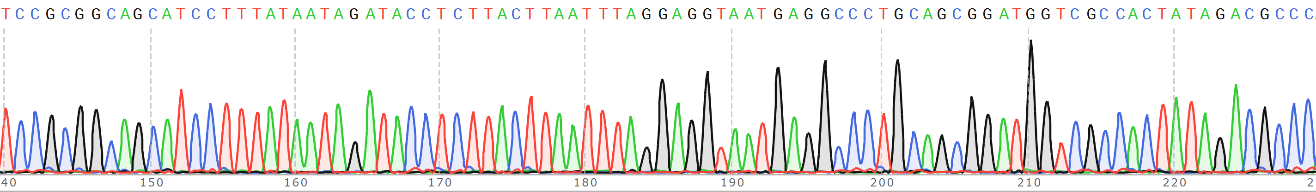

PCR_C4 : tccgcggcagcatcctttataatagatacctcttacttaatttaggaggtaatgaggccctgcagcggatggtcgccactatagacgccc : 180
R07_C4 : tccgcggcagcatcctttataatagatacctcttacttaatttaggaggtaatgaggccctgcagcggatggtcgccactatagacgccc : 180
 TCCGCGGC**AGCATCCTTTATAATAGATACCTCTTACTTAATTTAGGAGGTAATGAGGCCCTGCAGCGGATGGTCGCCACTATAGACGCCC**


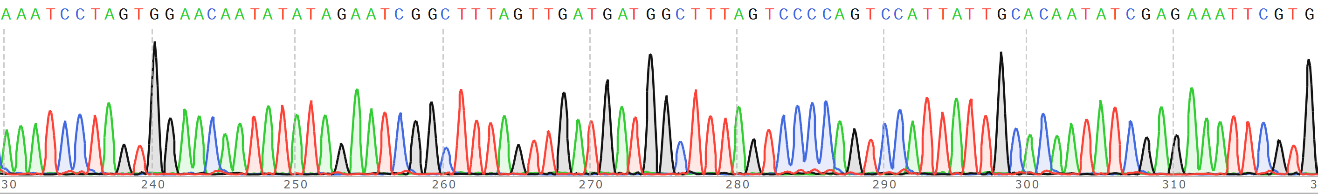

PCR_C4 : aaatcctagtggaacaatatatagaatcggctttagttgatgatggctttagtccccagtccattattgcacaatatcgagaaattcgtg : 270
R07_C4 : aaatcctagtggaacaatatatagaatcggctttagttgatgatggctttagtccccagtccattattgcacaatatcgagaaattcgtg : 270
 **AAATCCTAGTGGAACAATATATAGAATCGGCTTTAGTTGATGATGGCTTTAGTCCCCAGTCCATTATTGCACAATATCGAGAAATTCGTG**


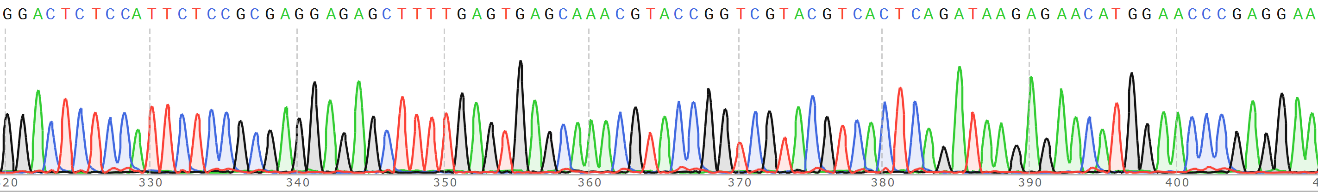

PCR_C4 : ggactctccattctccgcgaggagagcttttgagtgagcaaacgtaccggtcgtacgtcactcagataagagaacatggaacccgaggaa : 360
R07_C4 : ggactctccattctccgcgaggagagcttttgagtgagcaaacgtaccggtcgtacgtcactcagataagagaacatggaacccgaggaa : 360
 **GGACTCTCCATTCTCCGCGAGGAGAGCTTTTGAGTGAGCAAACGTACCGGTCGTACGTCACTCAGATAAGAGAACATGGAACCCGAGGAA**


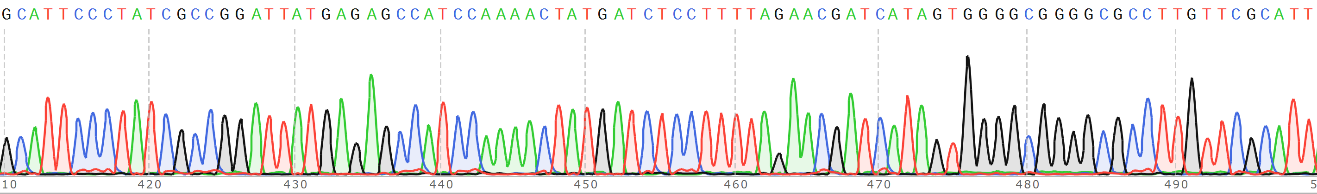

PCR_C4 : gcattccctatcgccggattatgagagccatccaaaactatgatctccttttagaacgatcatagtggggcggggcgccttgttcgcatt : 450
R07_C4 : gcattccctatcgccggattatgagagccatccaaaactatgatctccttttagaacgatcatagtggggcggggcgccttgttcgcatt : 450
 **GCATTCCCTATCGCCGGATTATGAGAGCCATCCAAAACTATGATCTCCTTTTAGAACGATCATAGTGGGGCGGGGCGCCTTGTTCGCATT**


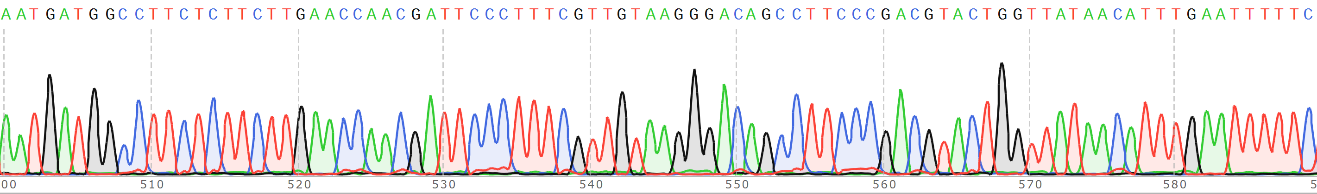

PCR_C4 : aatgatggccttctcttcttgaaccaacgattccctttcgttgtaagggacagccttcccgacgtactggttataacatttgaatttttc : 540
R07_C4 : aatgatggctttctcttcttgaaccaacgattccctttcgttgtaagggacagccttcccgacgtactggttataacatttgaatttttc : 540
 **AATGATGGC TTCTCTTCT**TGAACCAACGATTCCCTTTCGTTGTAAGGGACAGCCTTCCCGACGTACTGGTTATAACATTTGAATTTTTC


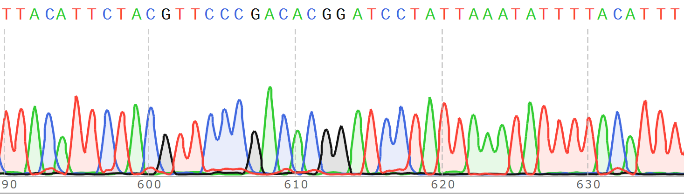

PCR_C4 : ttacattctacgttcccgacacggatcctattaaatattttacattt : 587
R07_C4 : ttacattctacgttcccgacacggatcctattaaatattttacattt : 587
 TTACATTCTACGTTCCCGACACGGATCCTATTAAATATTTTACATTT

Fig. S4 Alignment of the Nanopore long reads to the MTPTs and their flanking sequences in the mitogenome of *S. splendens*. Panels A-I show the alignment of the Nanopore long reads to the 9 MTPT sequences (mtpt01 to mtpt09) on the MC1 and panels J-O shows the alignment of the Nanopore long reads to the six MTPT sequences (mtpt10 to mtpt15) on the MC2. The figures were generated using IGV. The MTPT sequence was shown on the top of each figure with its coordinates. The coverage depth was shown as blue bars. The reads were represented with gray lines. The regions in gray represent a match. The purple regions represent small indels. Regions shown with other colors represent mismatched bases. The MTPT sequences are indicated with red lines having arrowheads at each end. The flanking sequences of 2000 bp long are indicated with the red line without arrowheads.

A alignment of Nanopore long reads to the sequence of mtpt01.


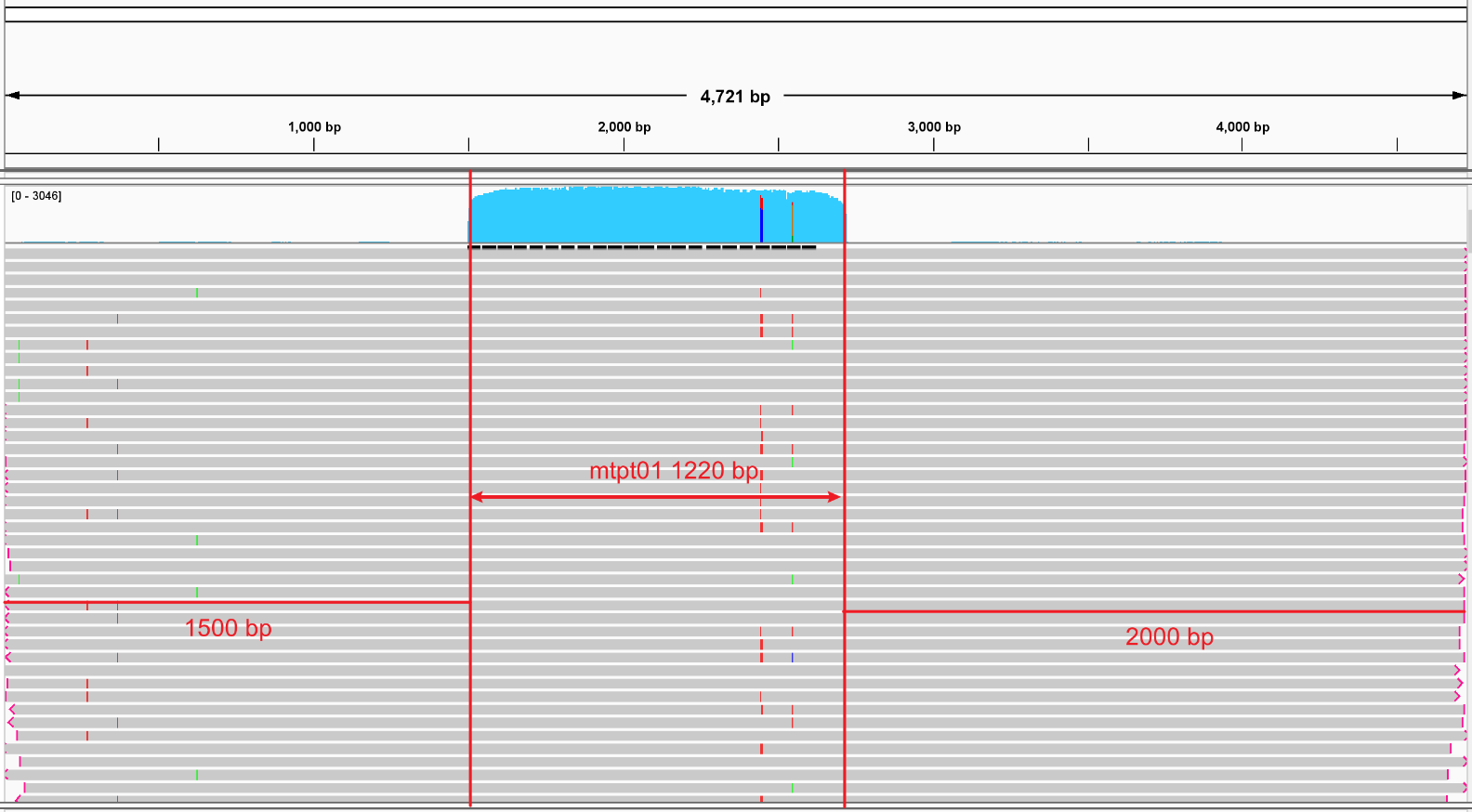


B alignment of Nanopore long reads to the sequence of mtpt02.


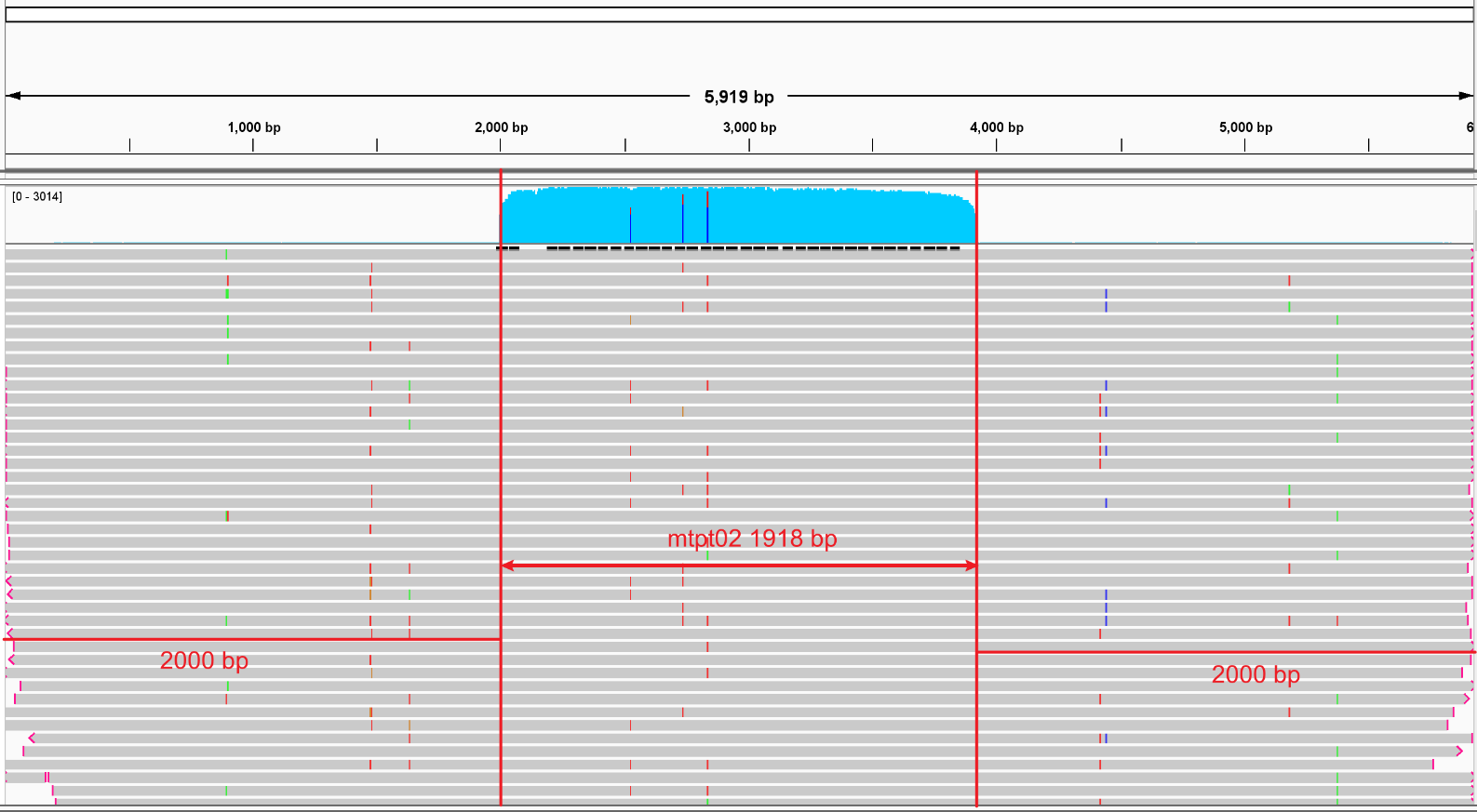


C alignment of Nanopore long reads to the sequence of mtpt03.


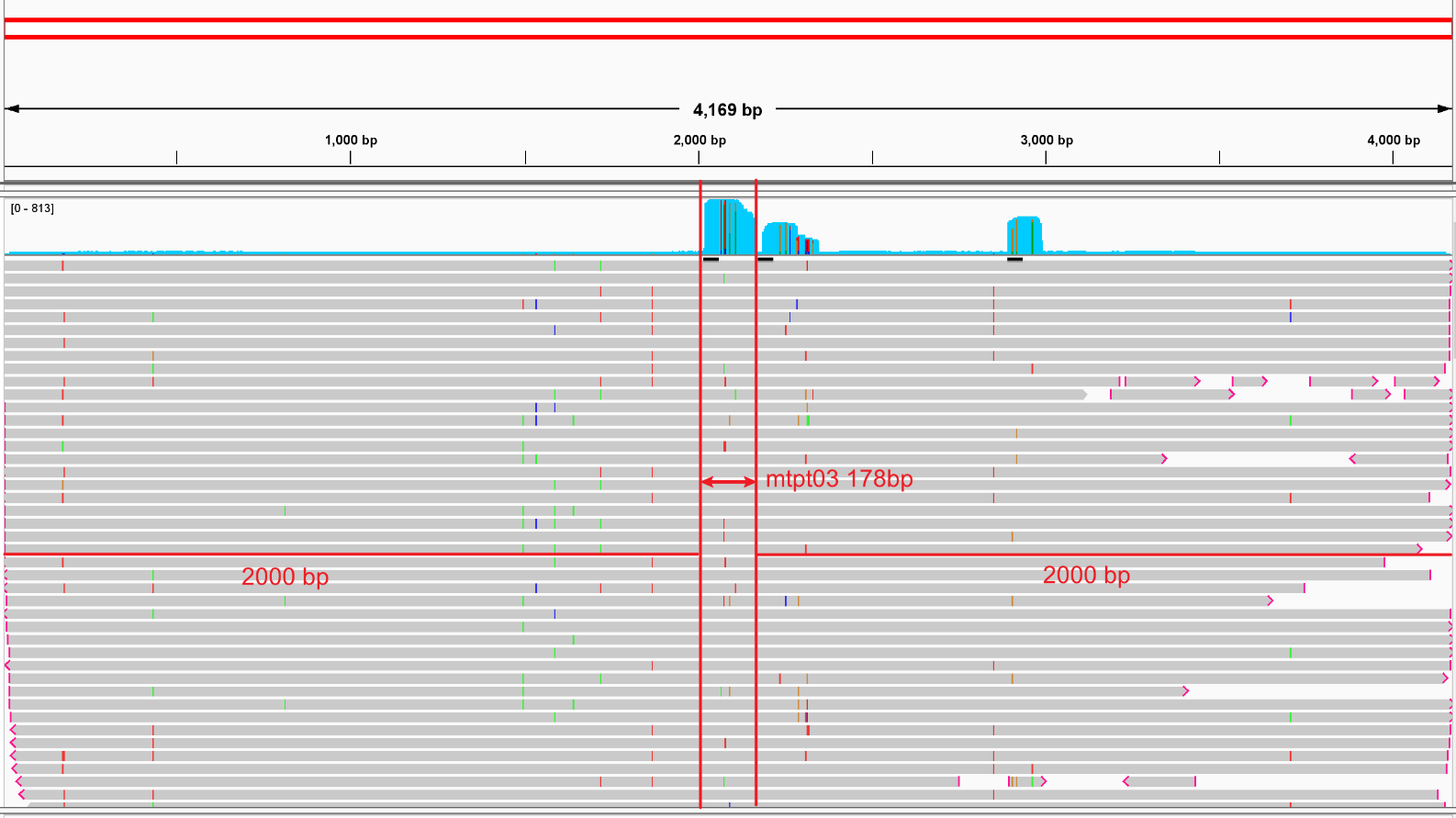


D alignment of Nanopore long reads to the sequence of mtpt04.


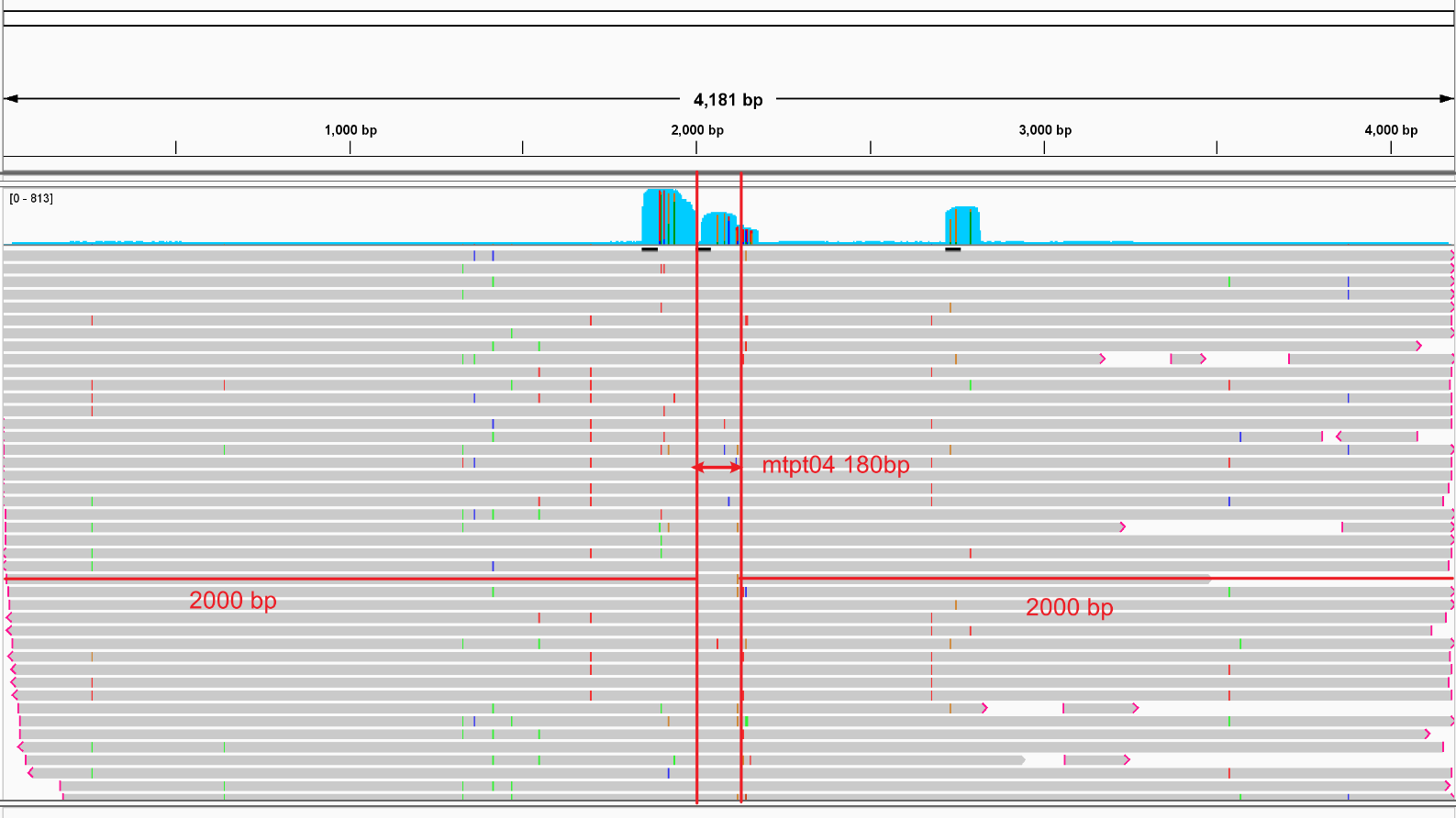


E alignment of Nanopore long reads to the sequence of mtpt05.


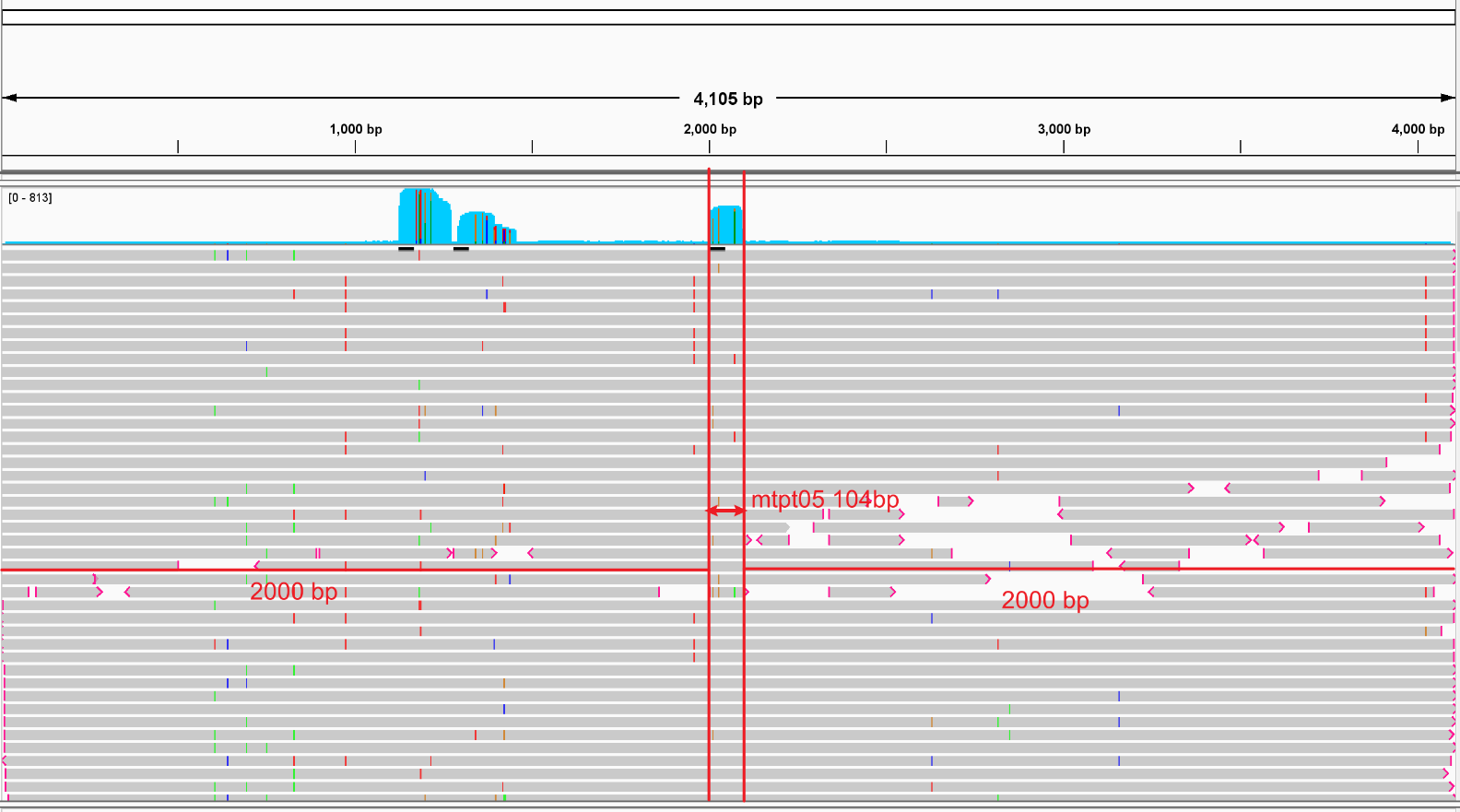


F alignment of Nanopore long reads to the sequence of mtpt06.


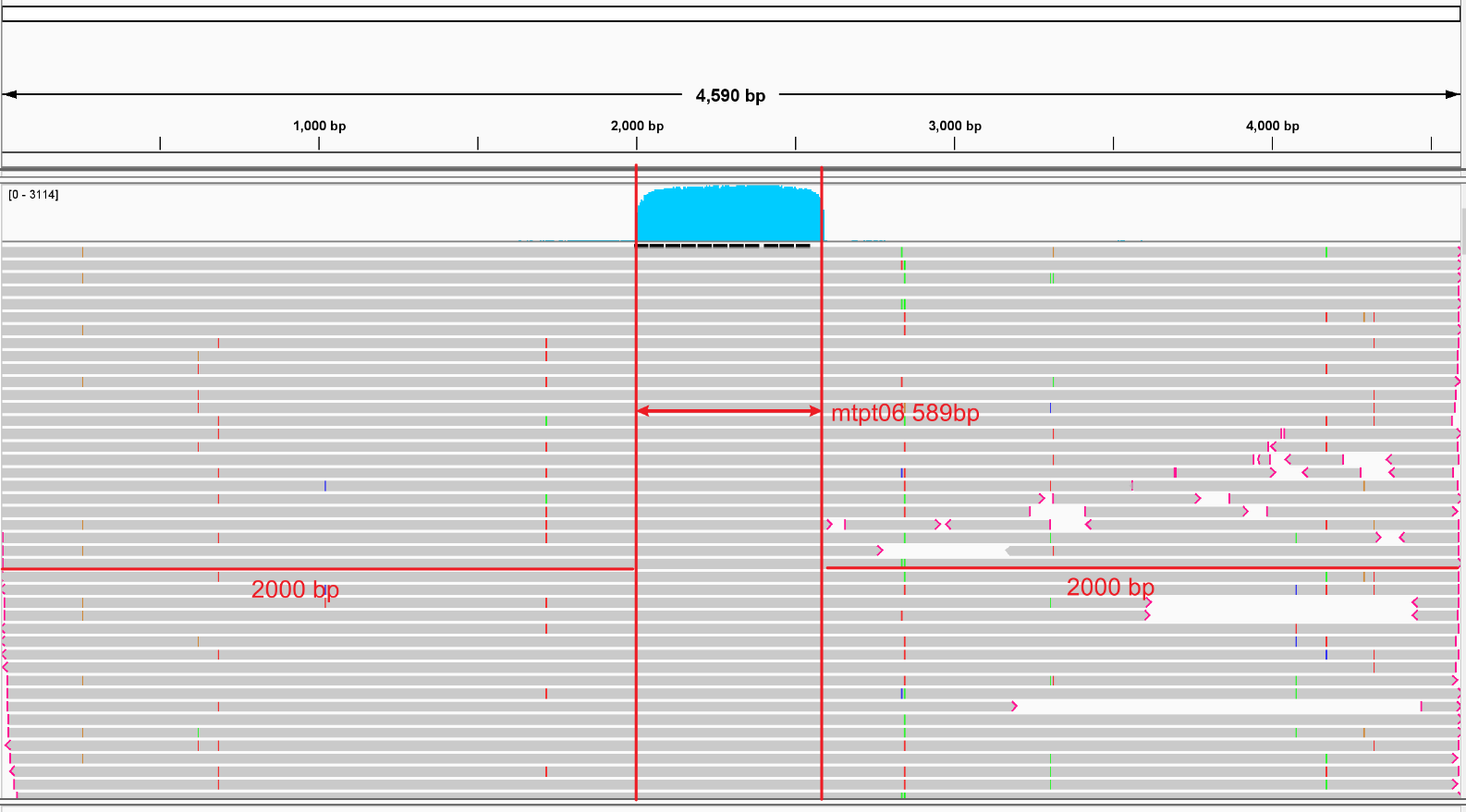


G alignment of Nanopore long reads to the sequence of mtpt07.


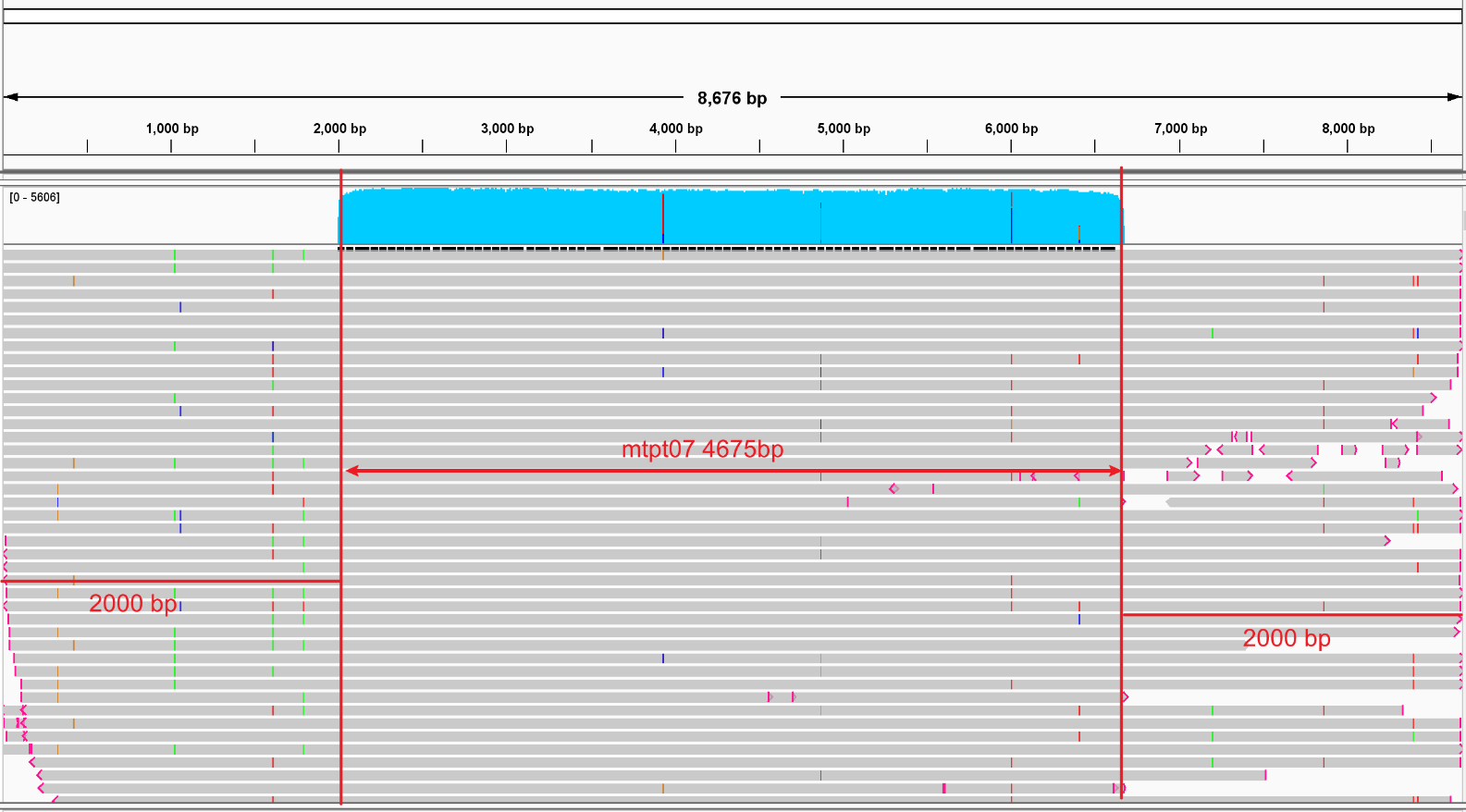


H alignment of Nanopore long reads to the sequence of mtpt08.


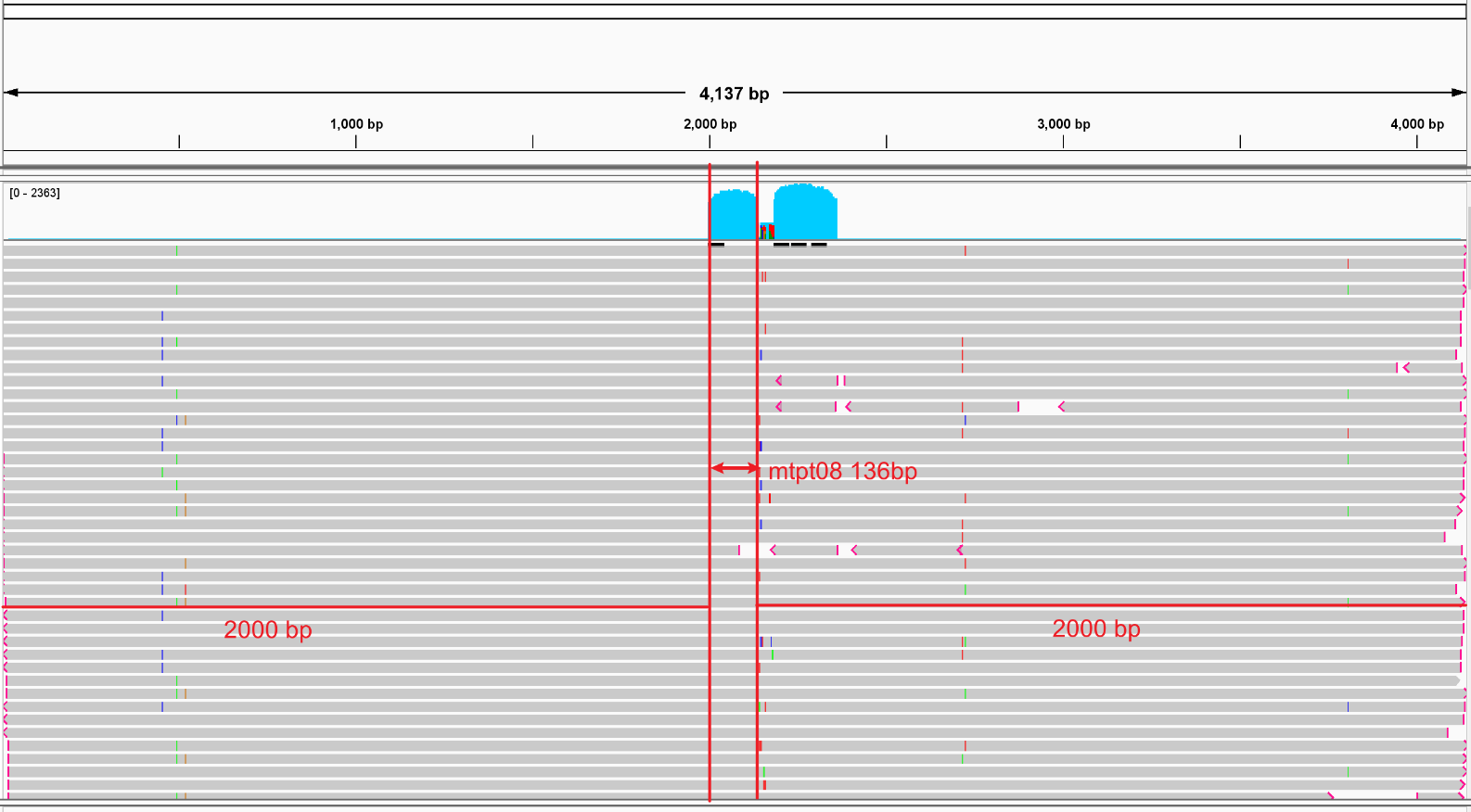


I alignment of Nanopore long reads to the sequence of mtpt09.


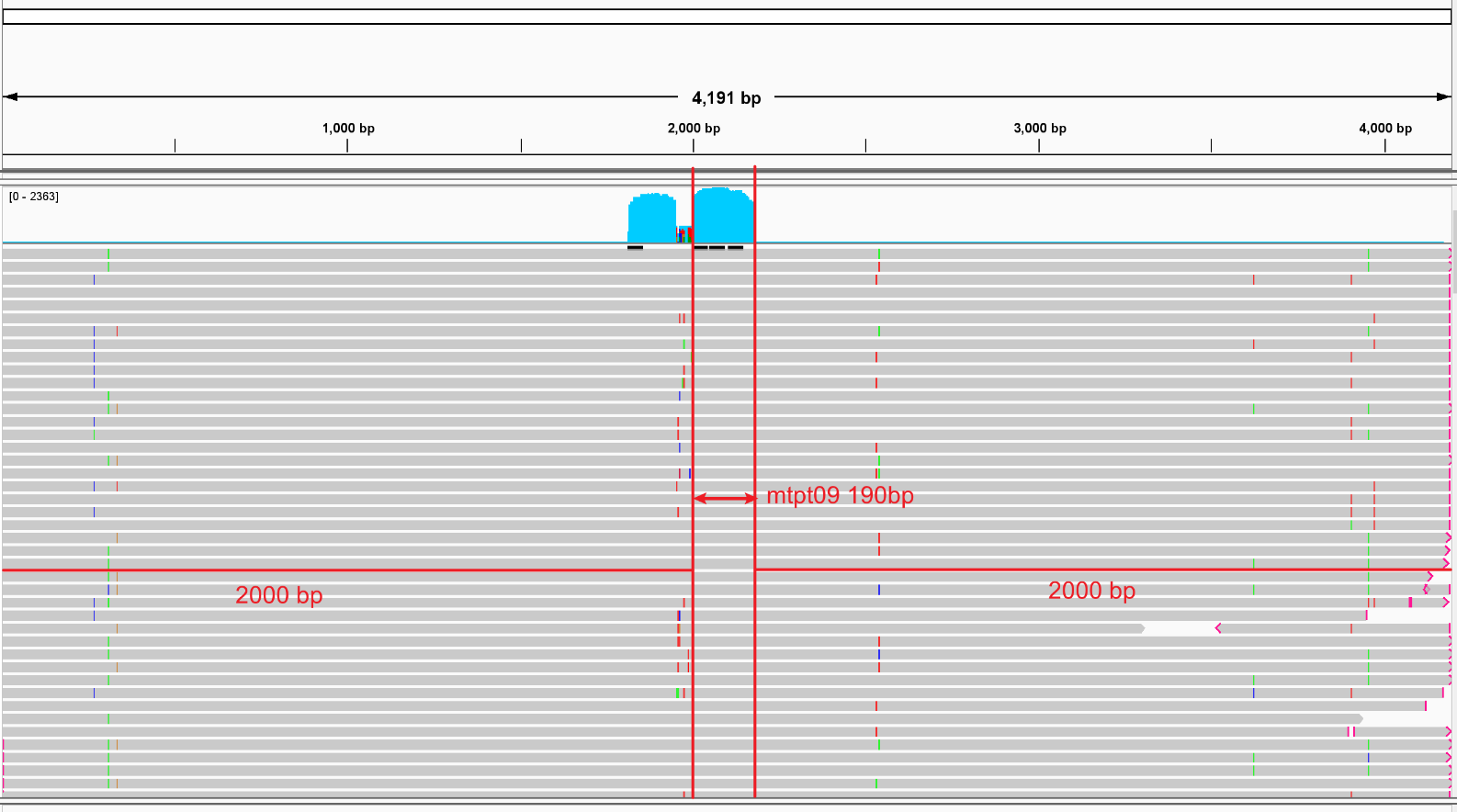


J alignment of Nanopore long reads to the sequence of mtpt10.


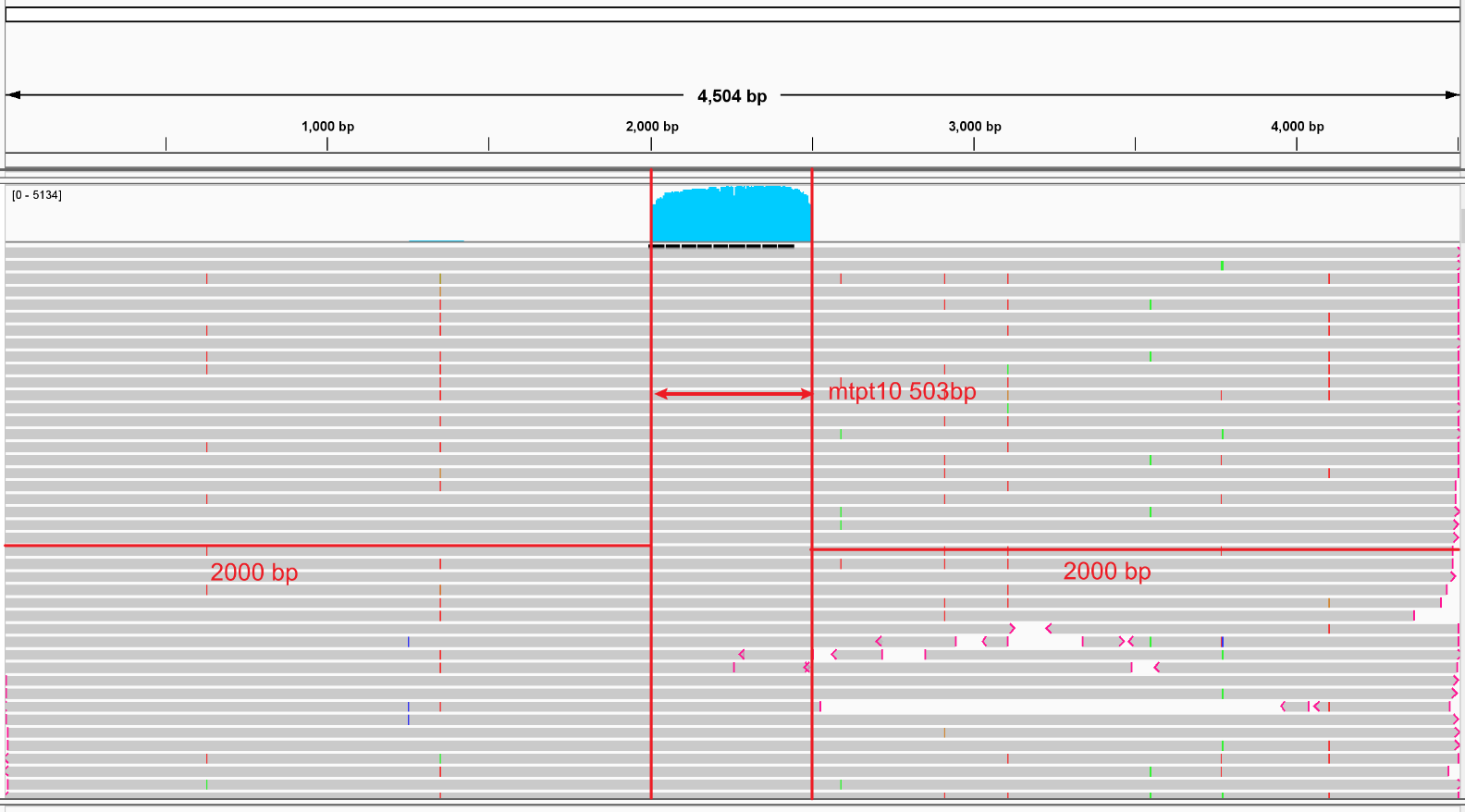


K alignment of Nanopore long reads to the sequence of mtpt11.


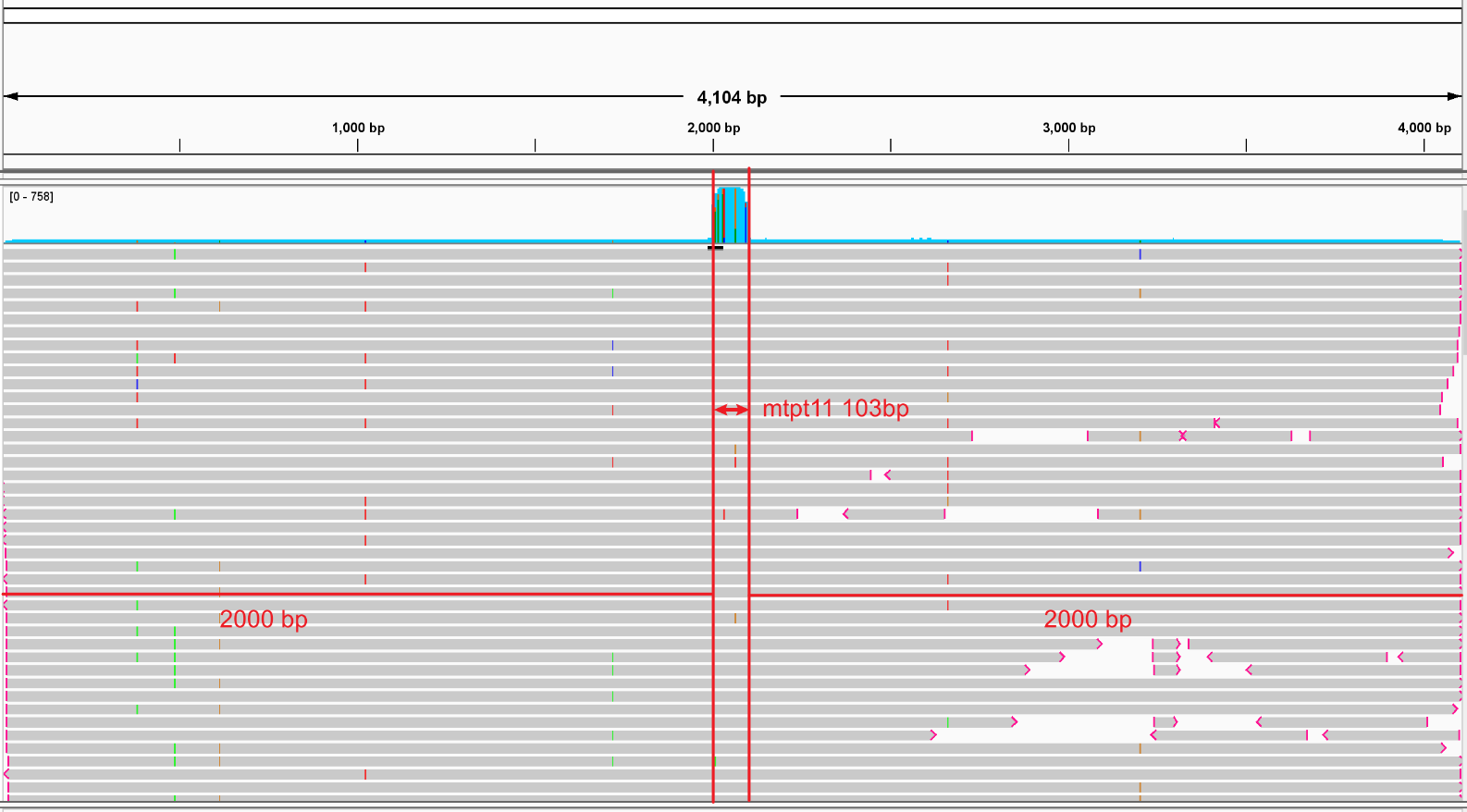


L alignment of Nanopore long reads to the sequence of mtpt12.


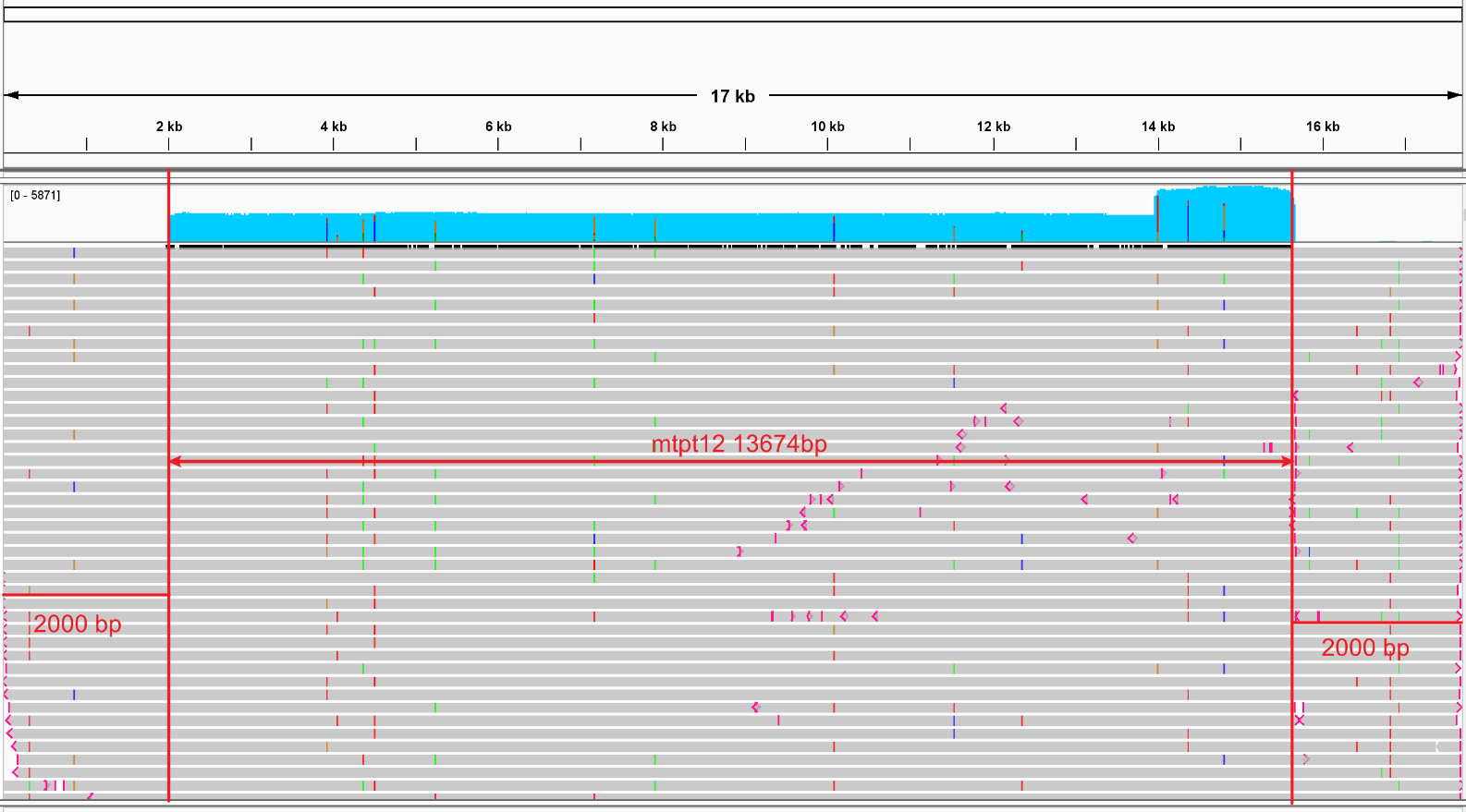


M alignment of Nanopore long reads to the sequence of mtpt13.


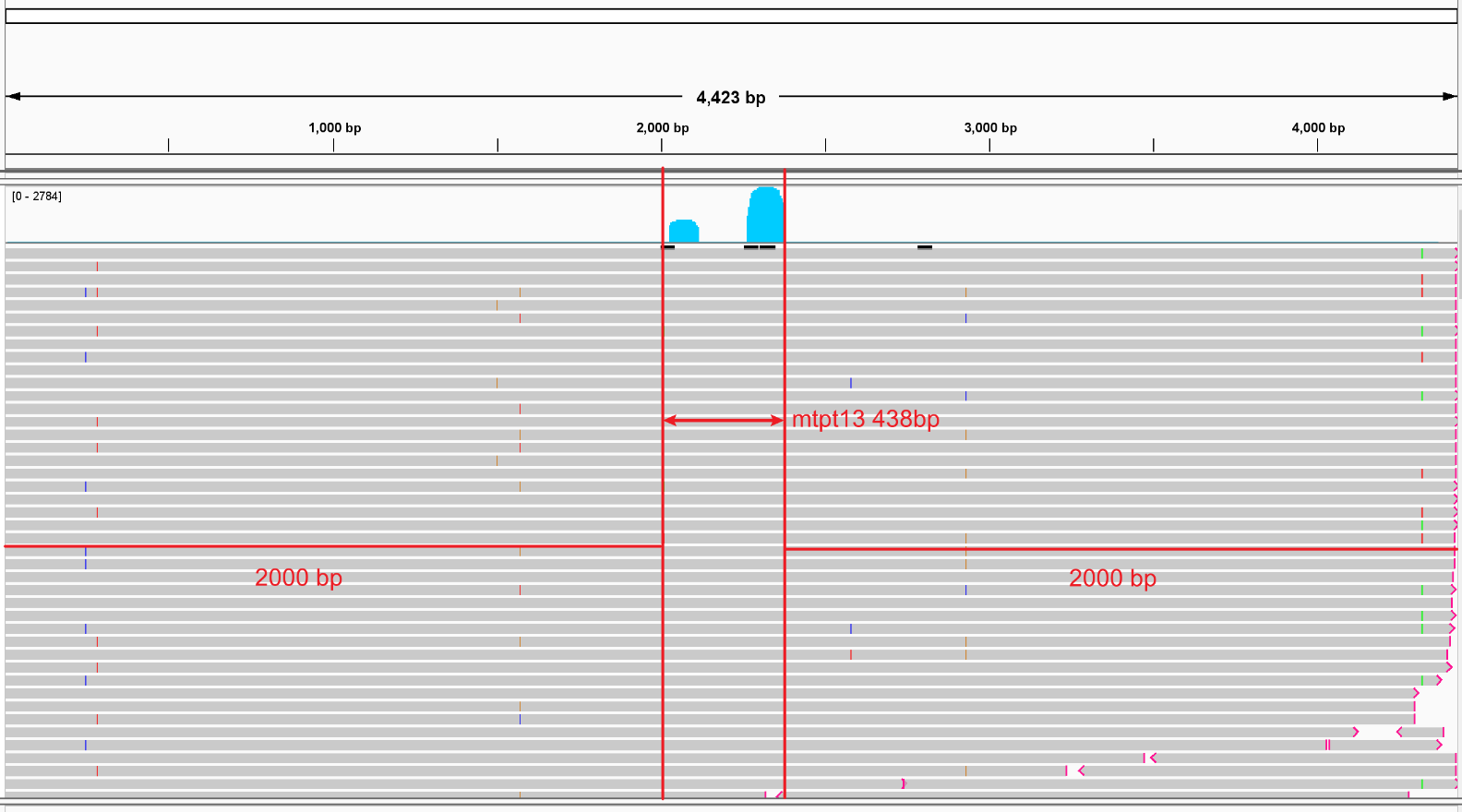


N alignment of Nanopore long reads to the sequence of mtpt14.


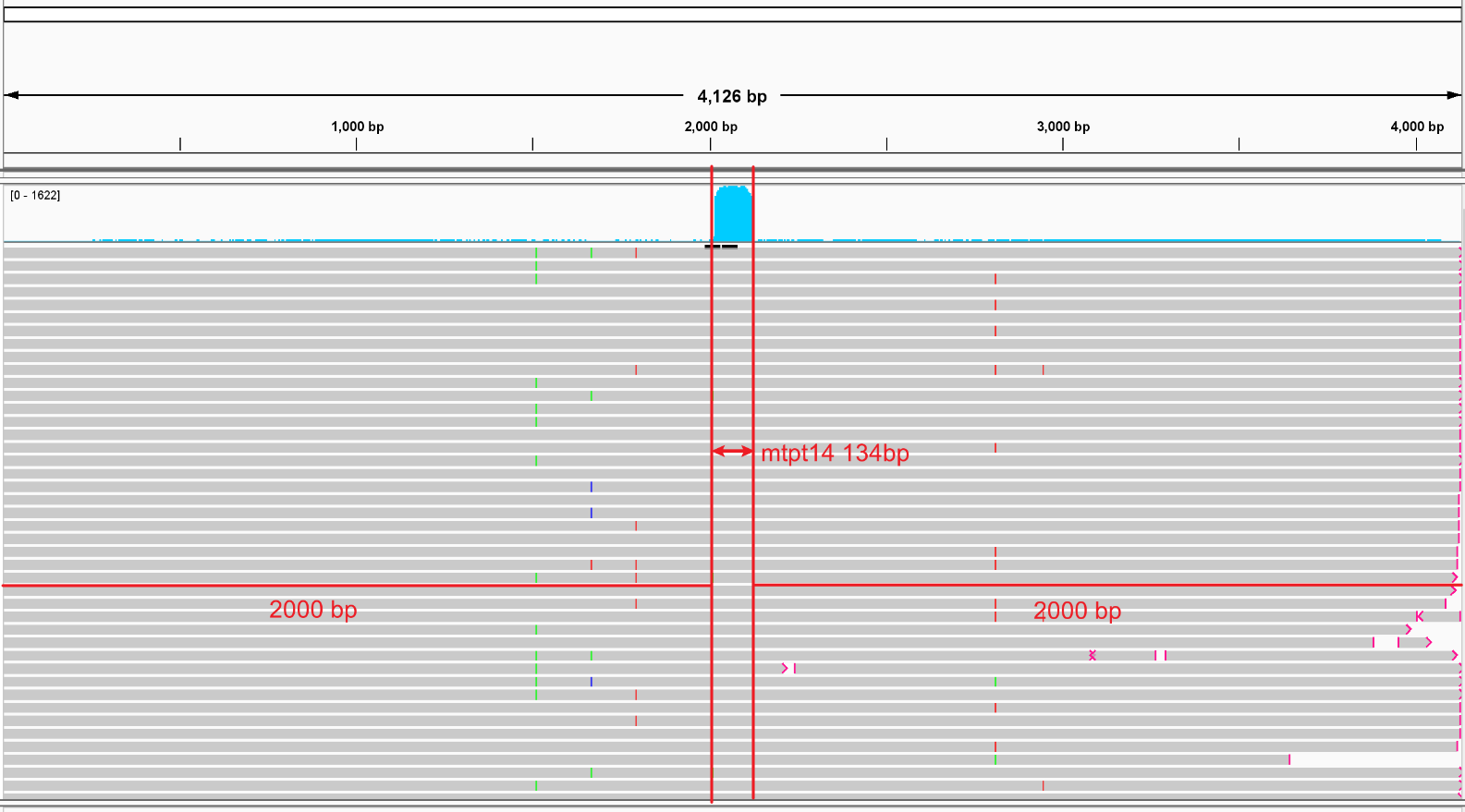


O alignment of Nanopore long reads to the sequence of mtpt15.


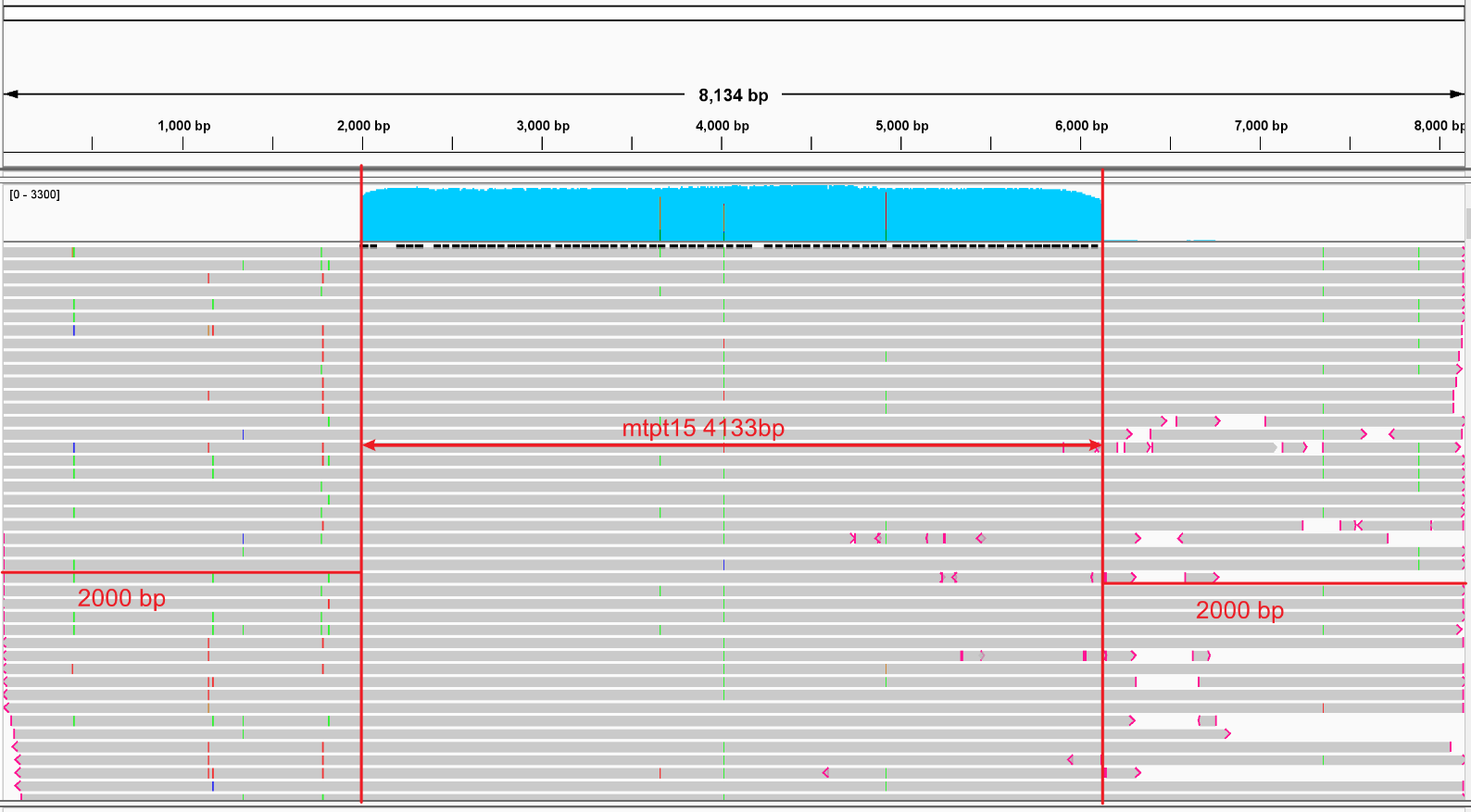


Fig. S5 Alignments of the RNA-seq reads to the coding sequences of twenty PCGs in the mitogenome of *S. splendens*. The reference sequences were shown at the bottom. The horizontal bar on the top of the figure represents the mitogenome sequence. The region highlighted in the red square is zoomed in and shown below. The gray lines represent the reads mapped to the reference sequence. Bases matching those in the reference sequences are shaded in gray. Bases not matching those in the reference sequences are shown in green for “A”, brown for “G”, blue for “C” and red for “T”. The RNA editing sites are shown in red squares.

A alignment of RNA-seq reads to the coding sequence of *atp4*. 12 RNA-seq editing sites: atp4-59, 71, 89, 118, 138, 144, 215, 227, 248, 251, 395, 407, and 416 are highlighted in red squares.


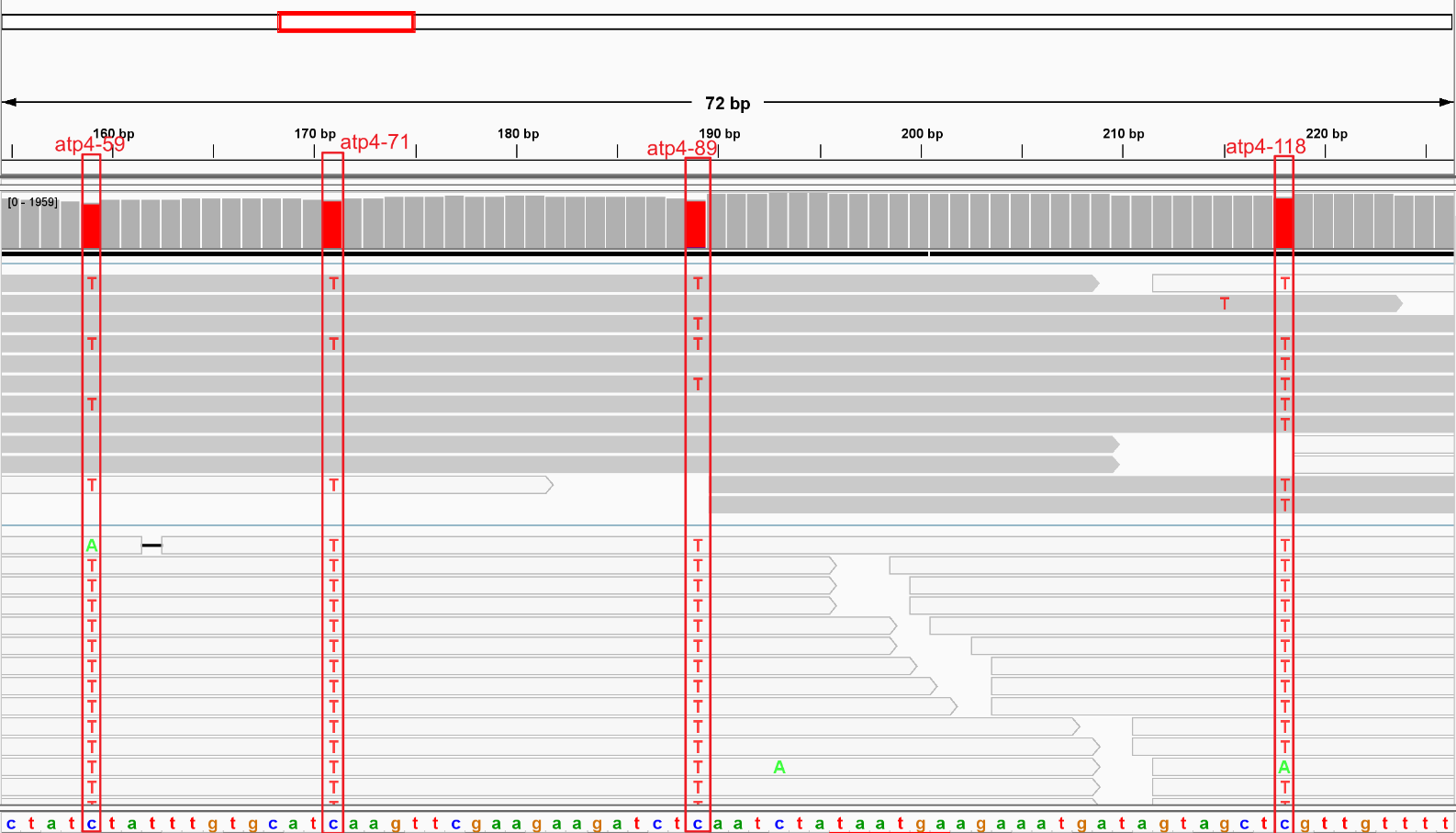

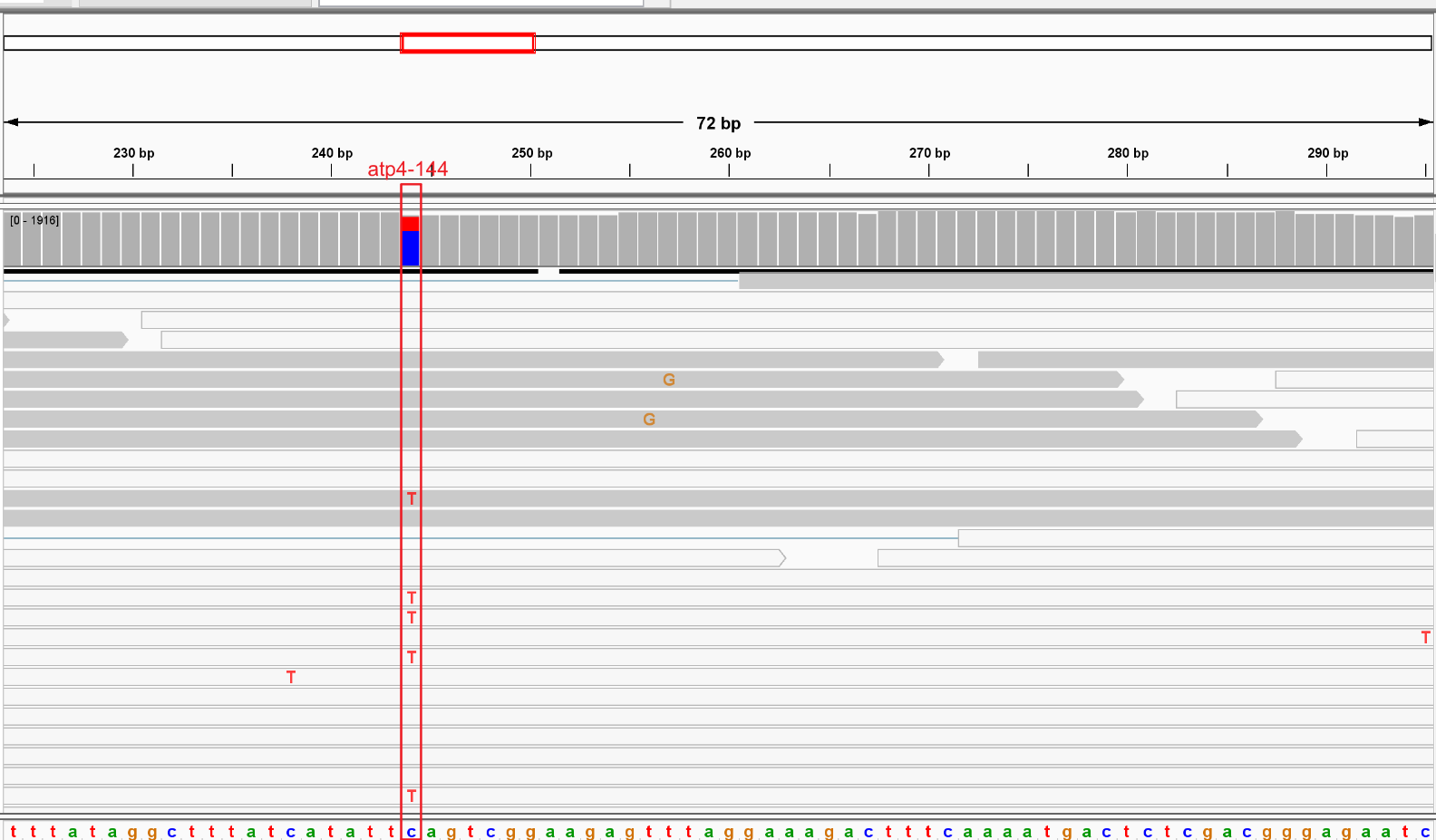

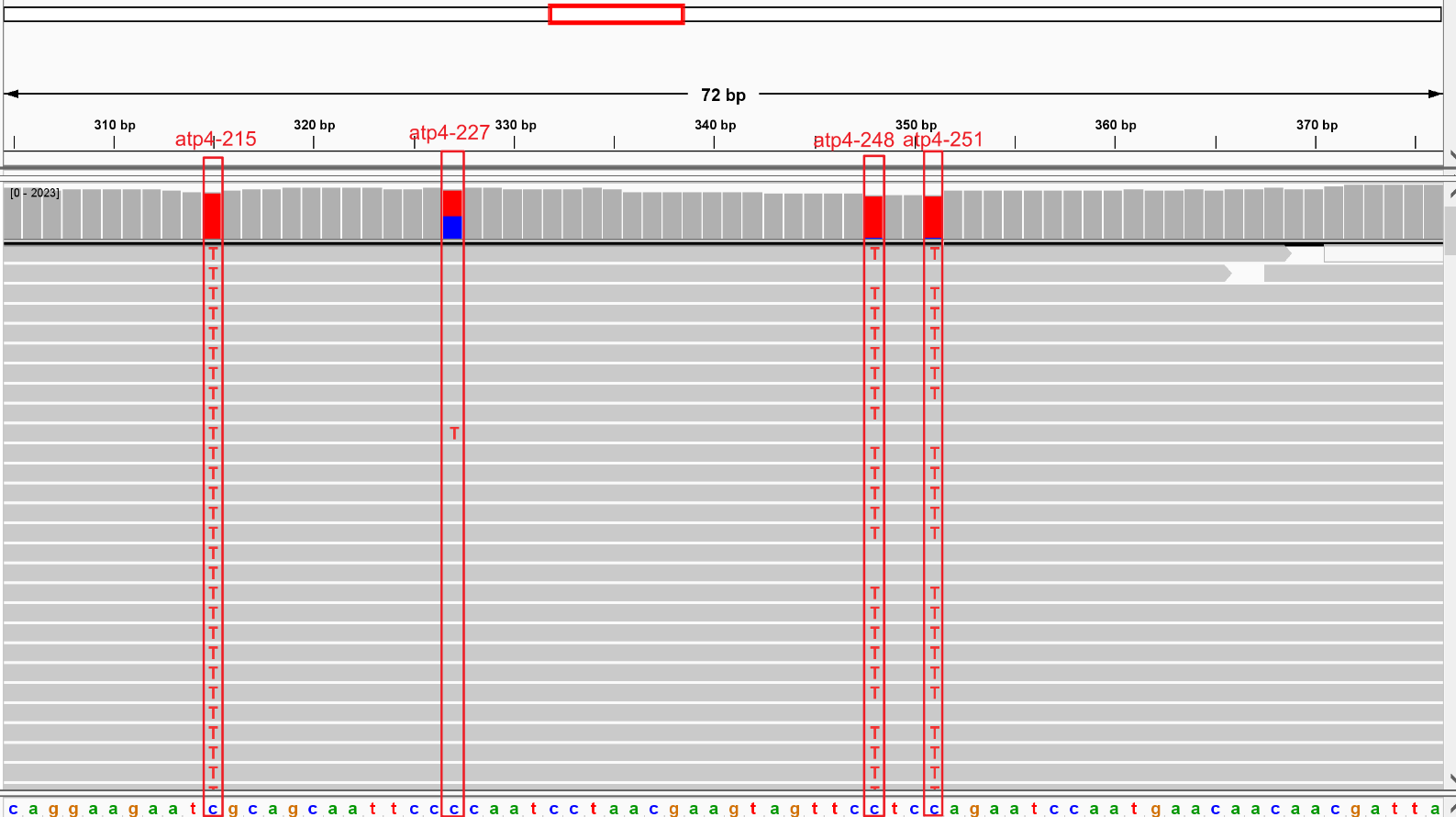

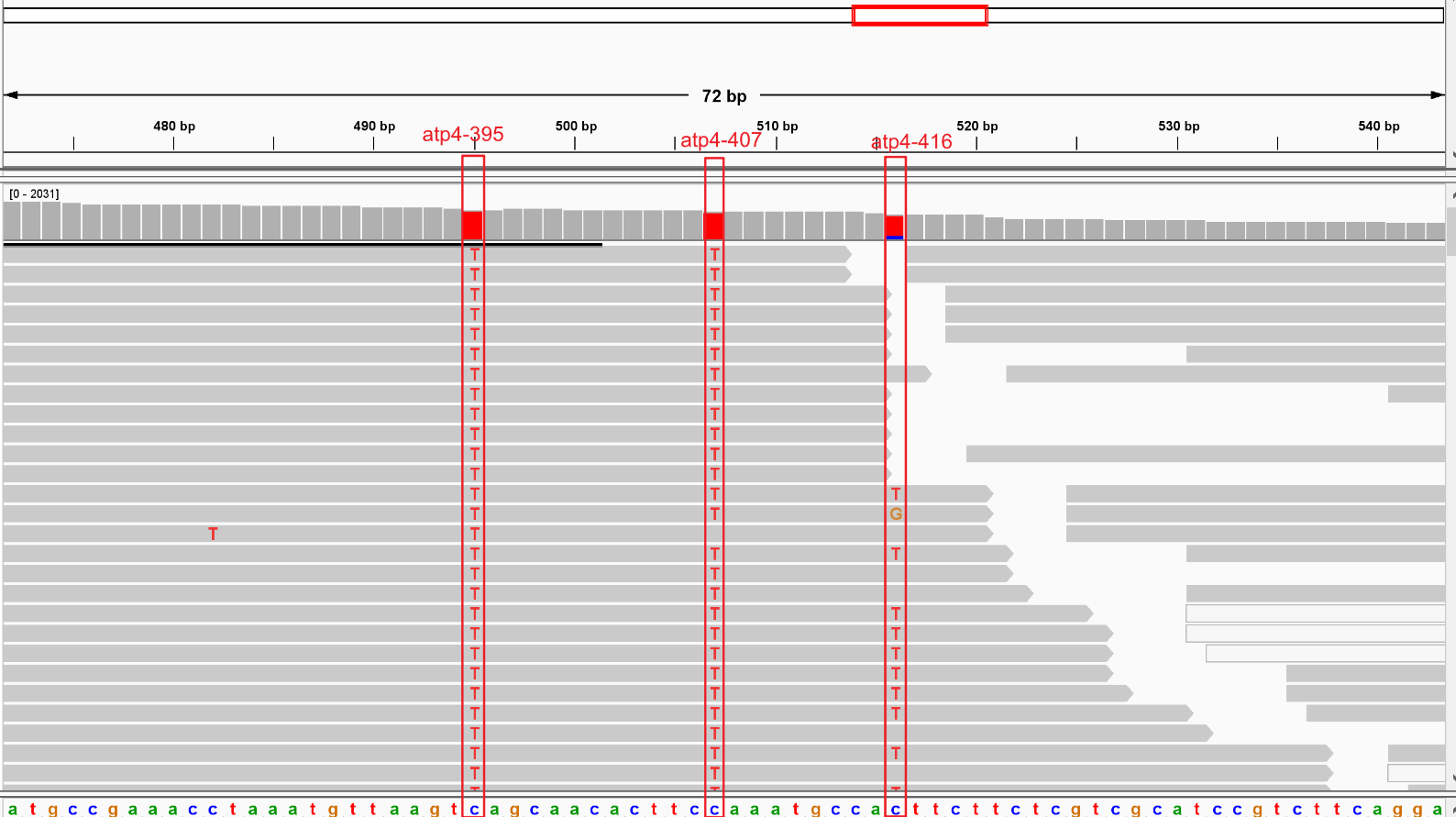


B alignment of RNA-seq reads to the coding sequence of *atp6*. Nine RNA-seq editing sites: atp6-218, 287, 305, 313, 452, 511, 514, and 639 are highlighted in red squares.


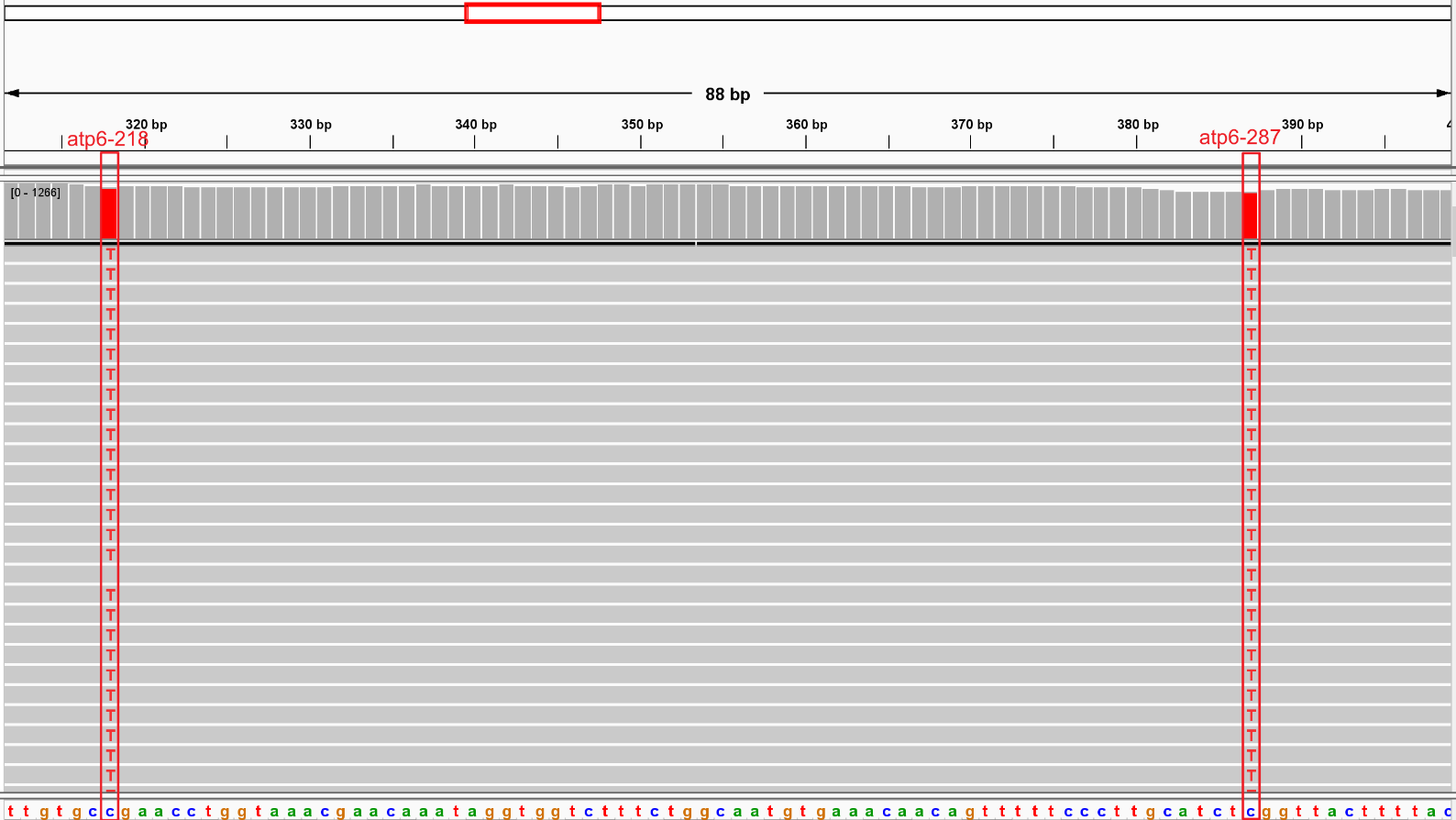

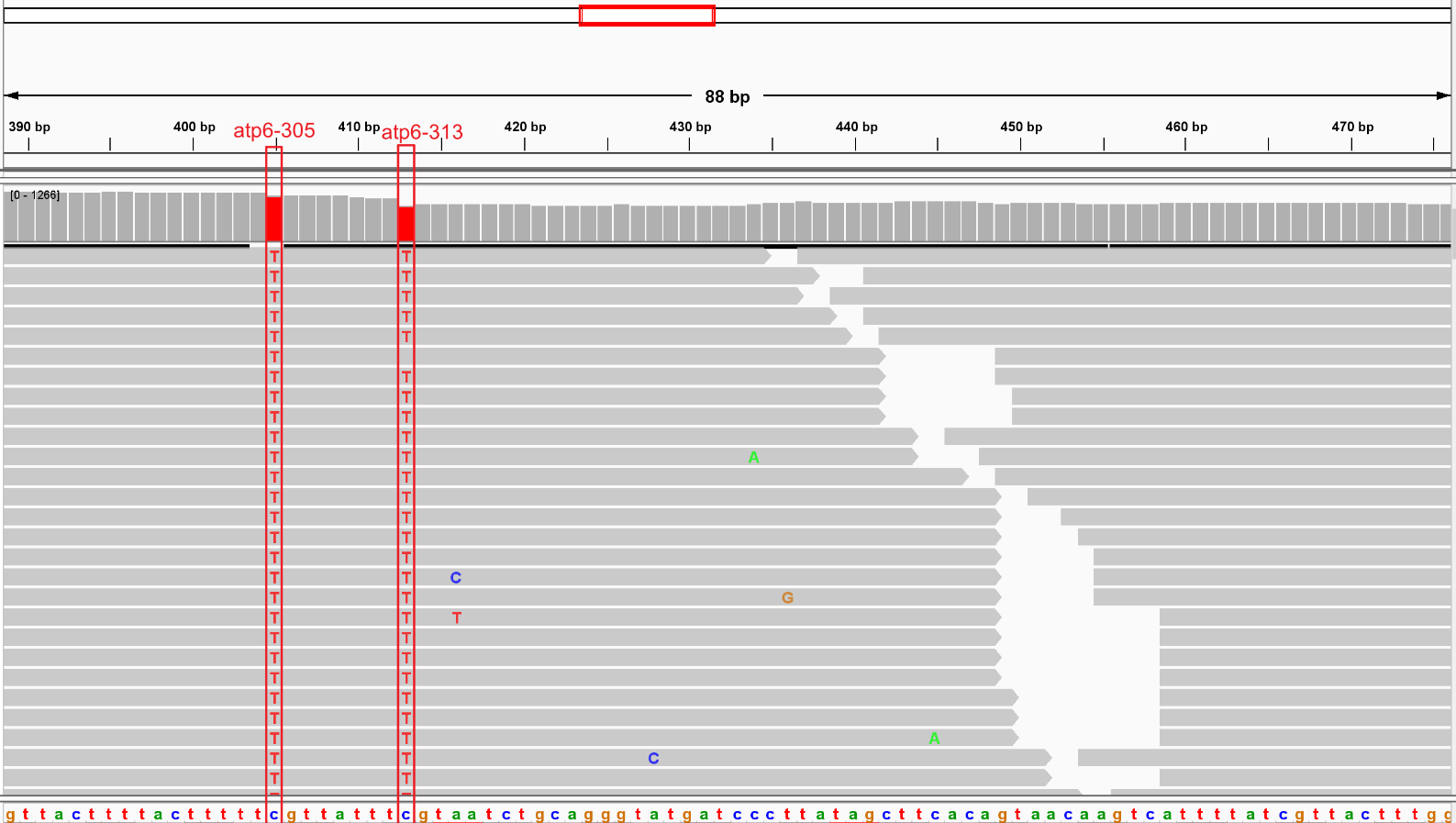


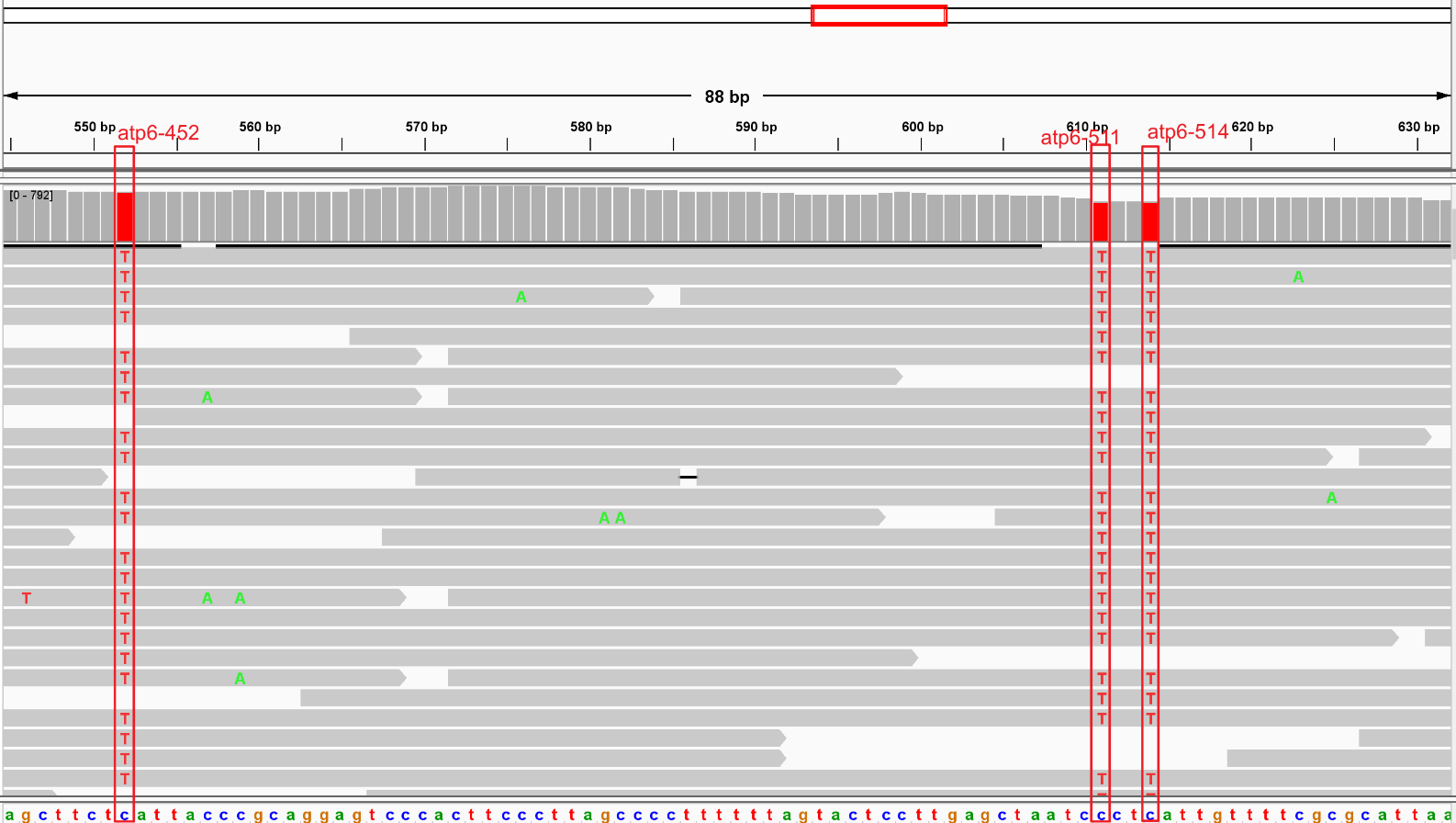


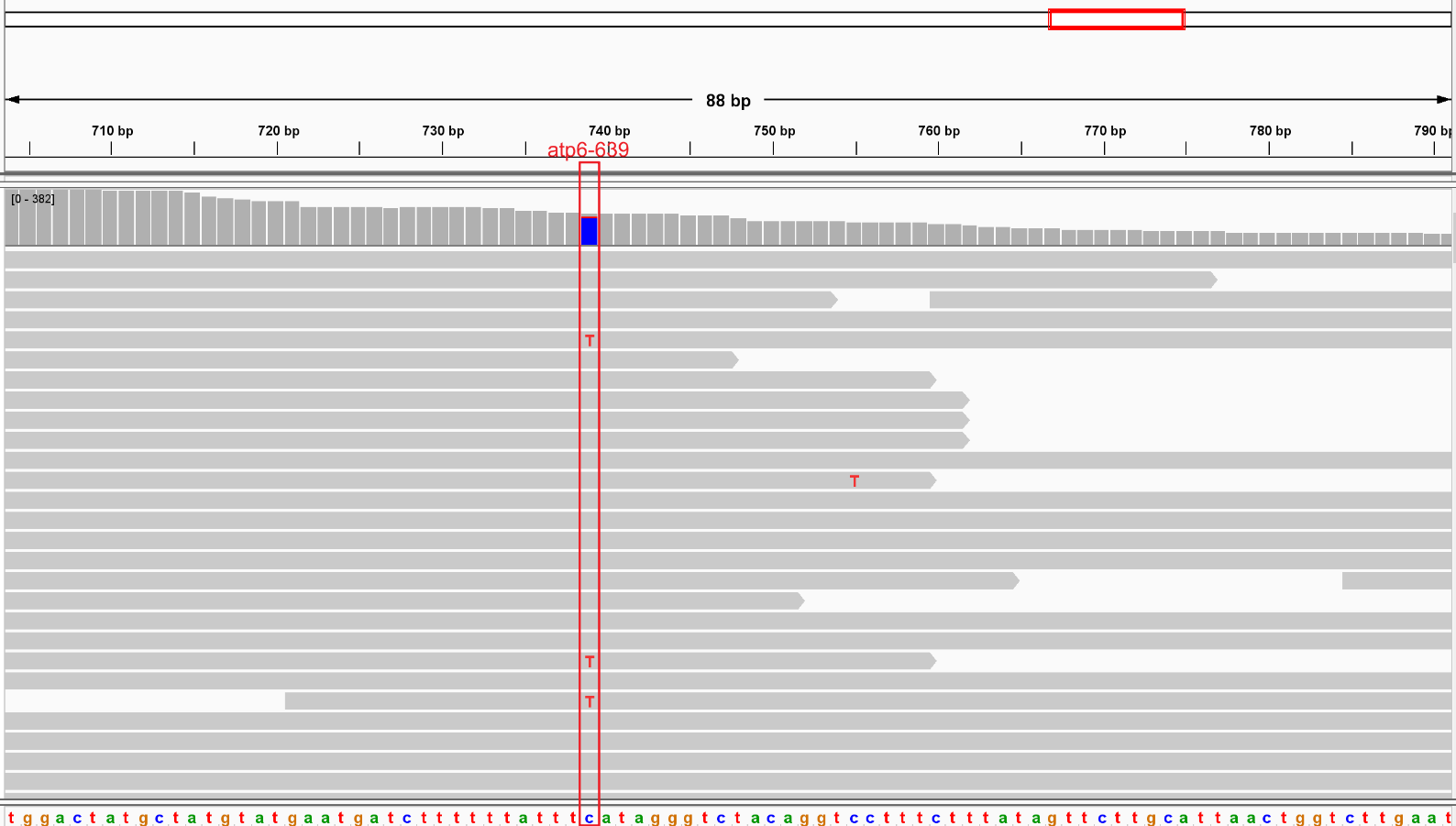


C alignment of RNA-seq reads to the coding sequence of *atp8*. Two RNA-seq editing sites: atp8-30 and 58 are highlighted in red squares.


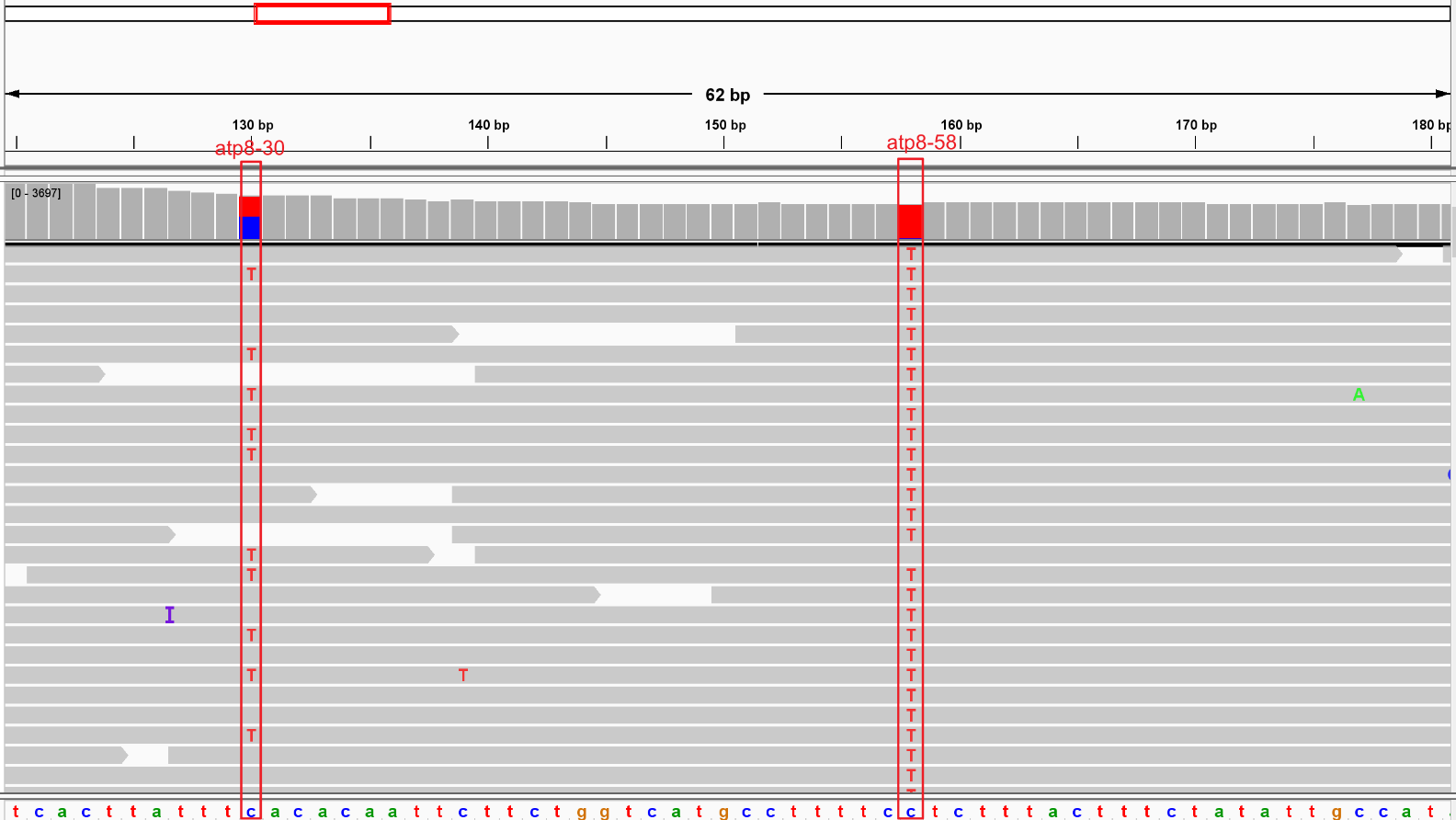


D alignment of RNA-seq reads to the coding sequence of *atp9*. One RNA-seq editing site: atp9-212 was highlighted in red squares.


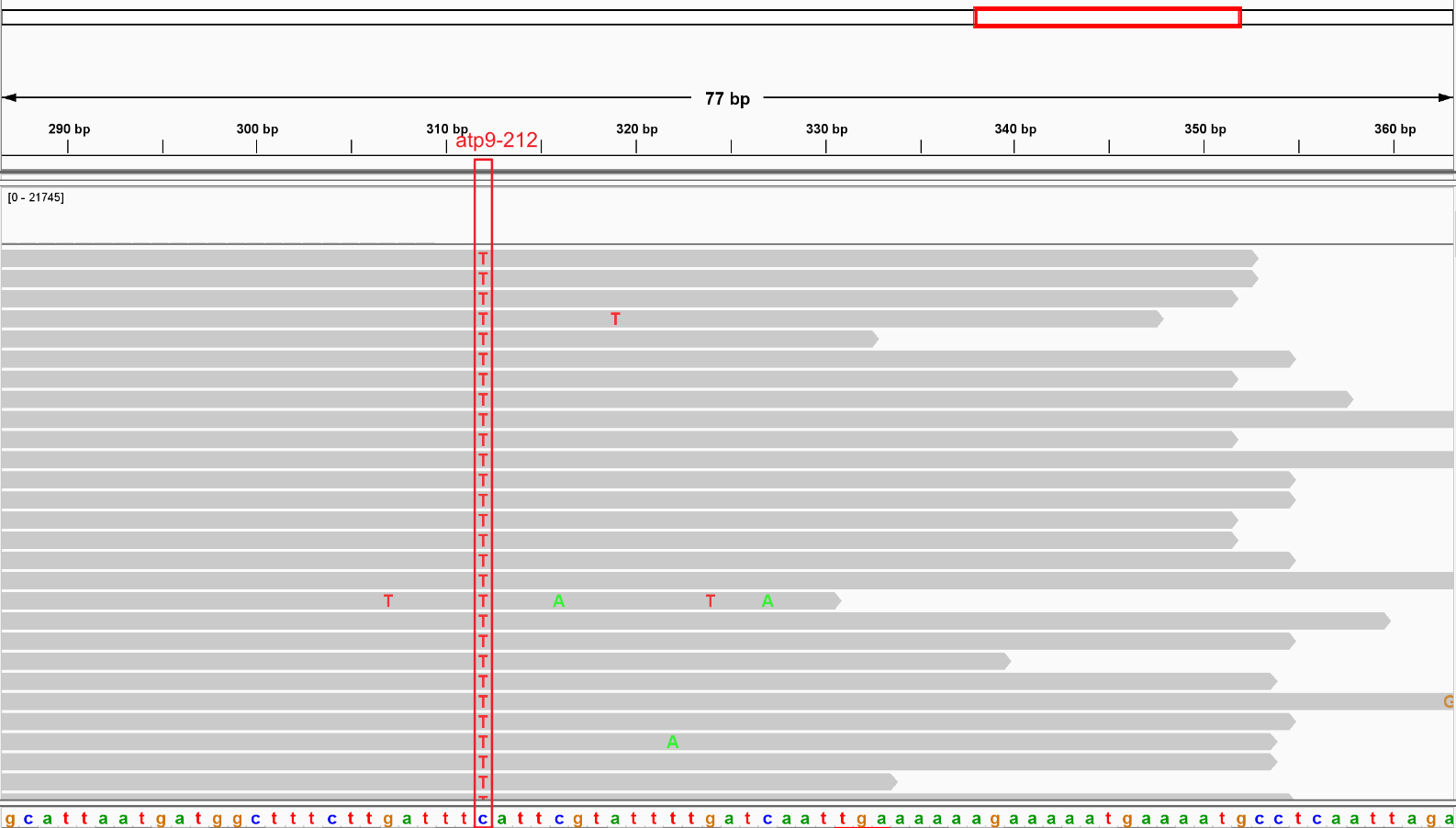


E alignment of RNA-seq reads to the coding sequence of *ccmB*. 26 RNA-seq editing sites: ccmB-28, 45, 80, 87, 128, 137, 160, 172, 193, 194, 286, 304, 313, 338, 367, 380, 424, 428, 467, 512, 551, 554, 566, 572, 596, and 611 are highlighted in red squares.


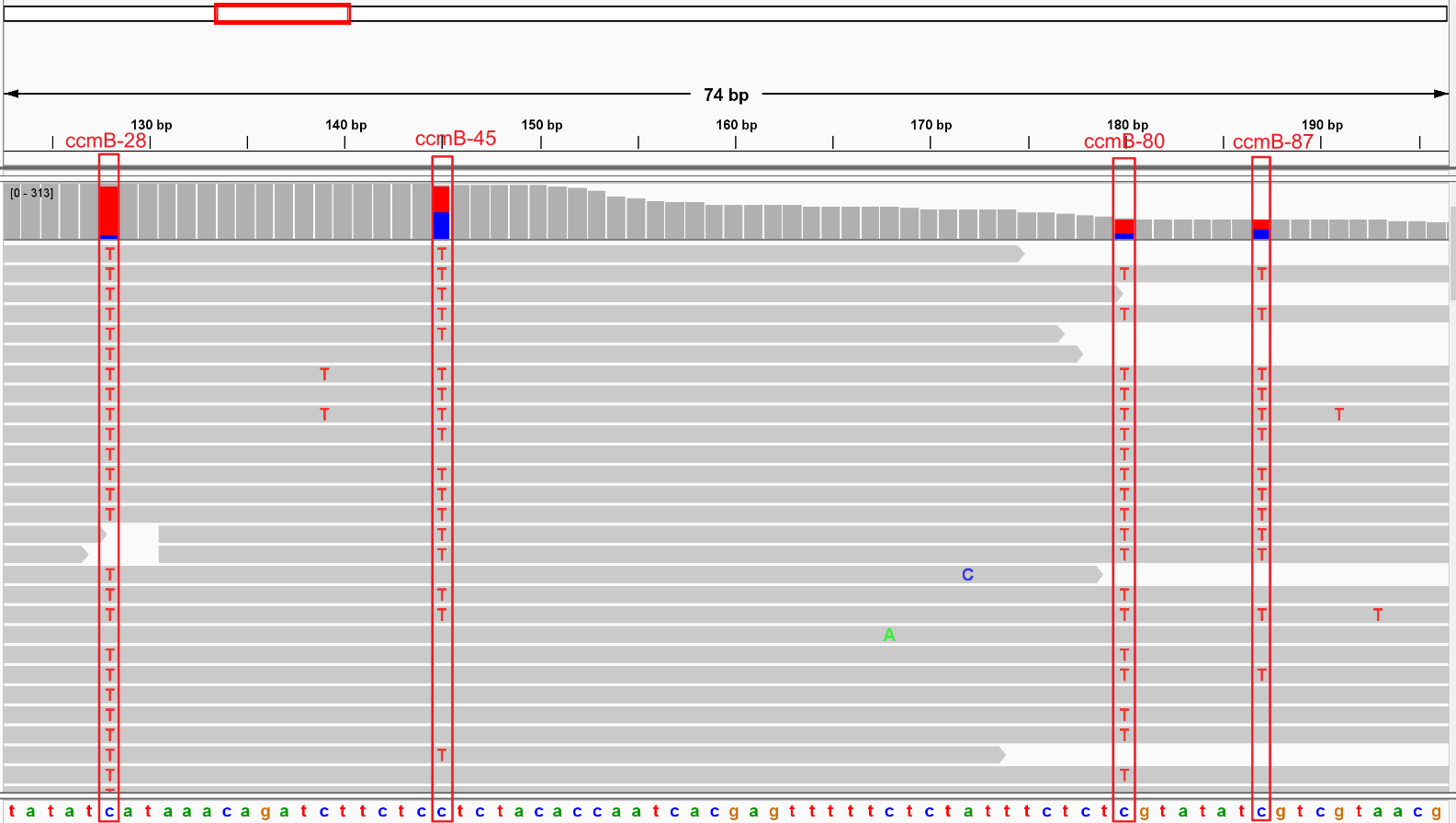

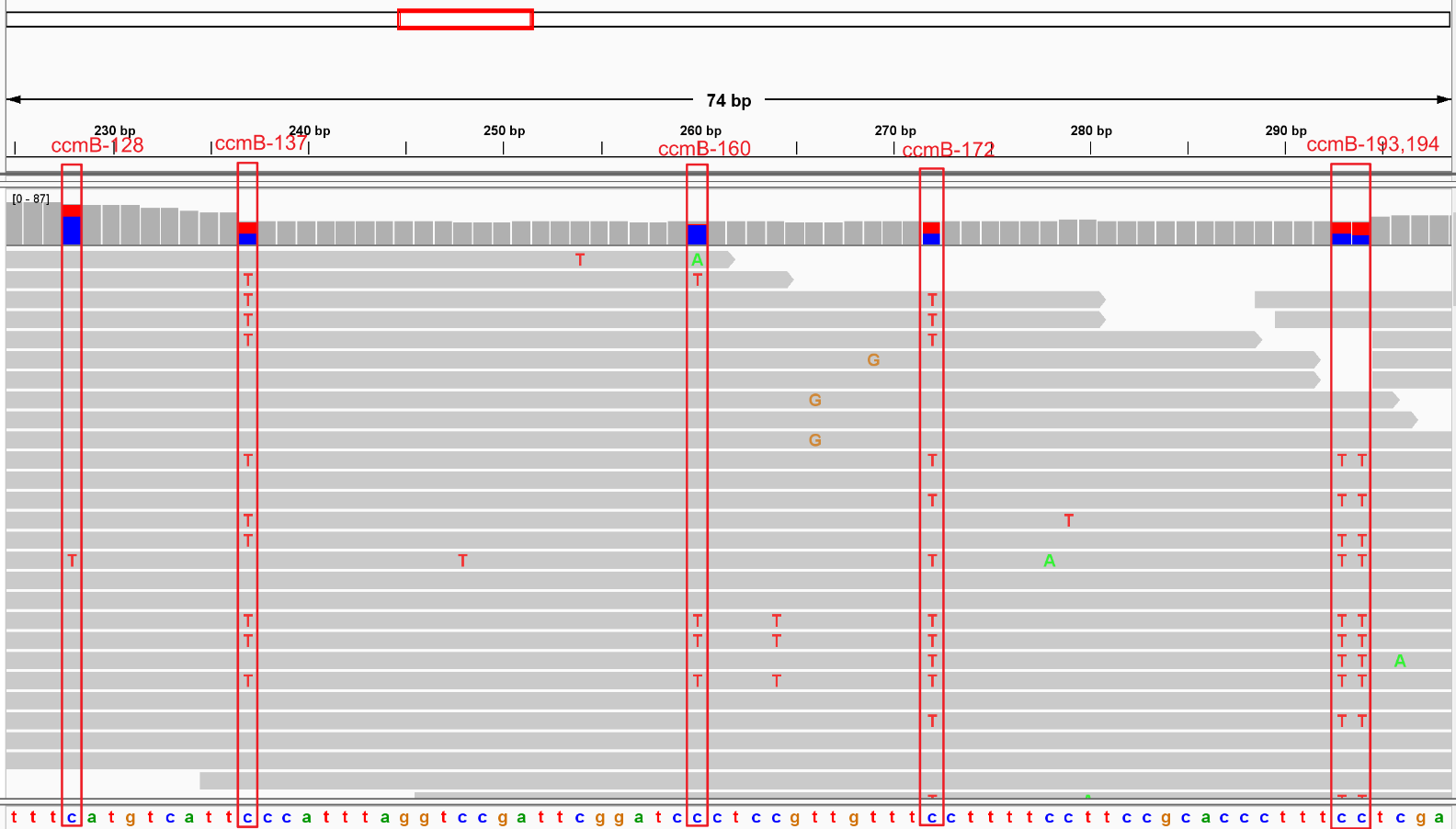


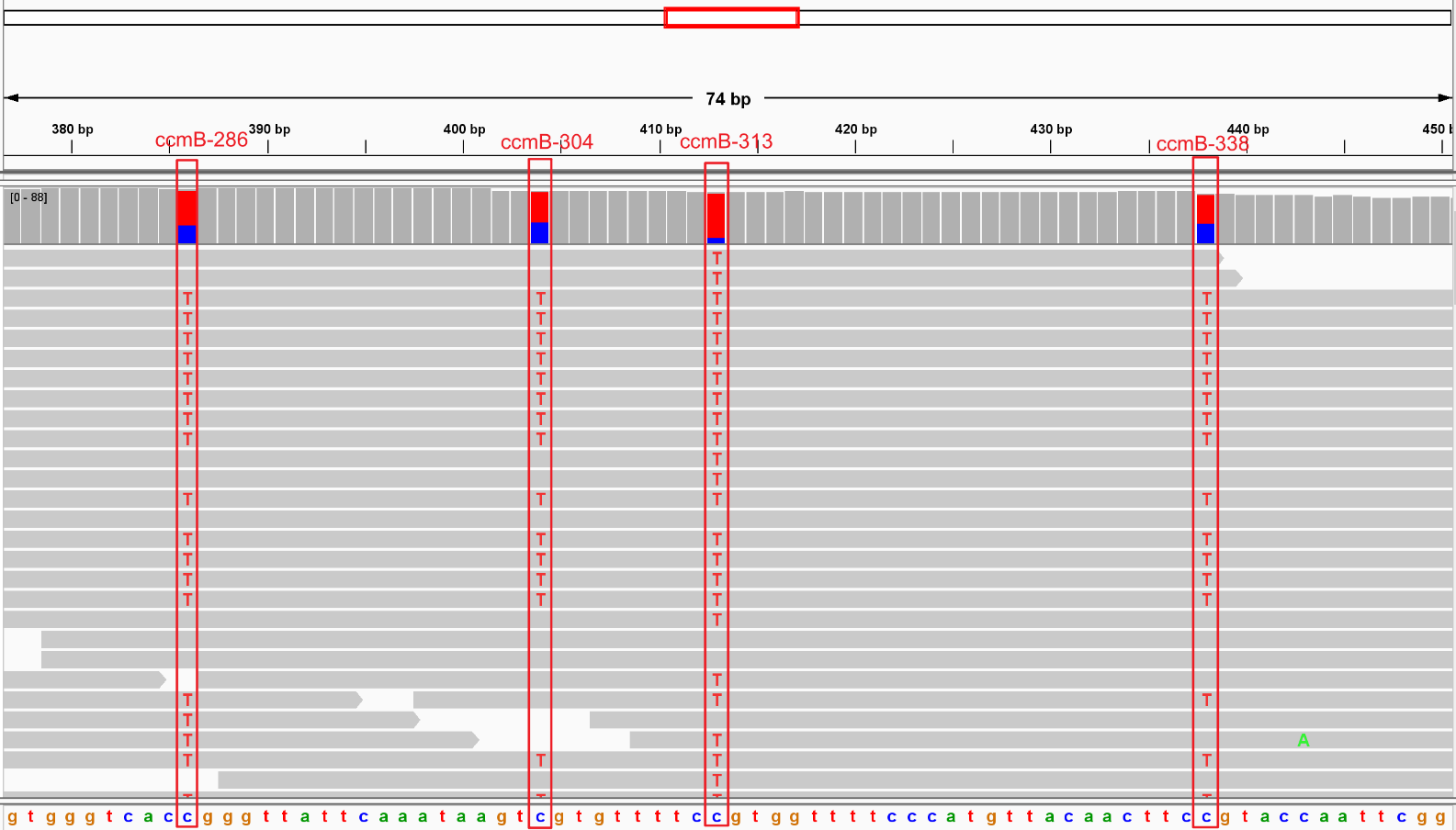

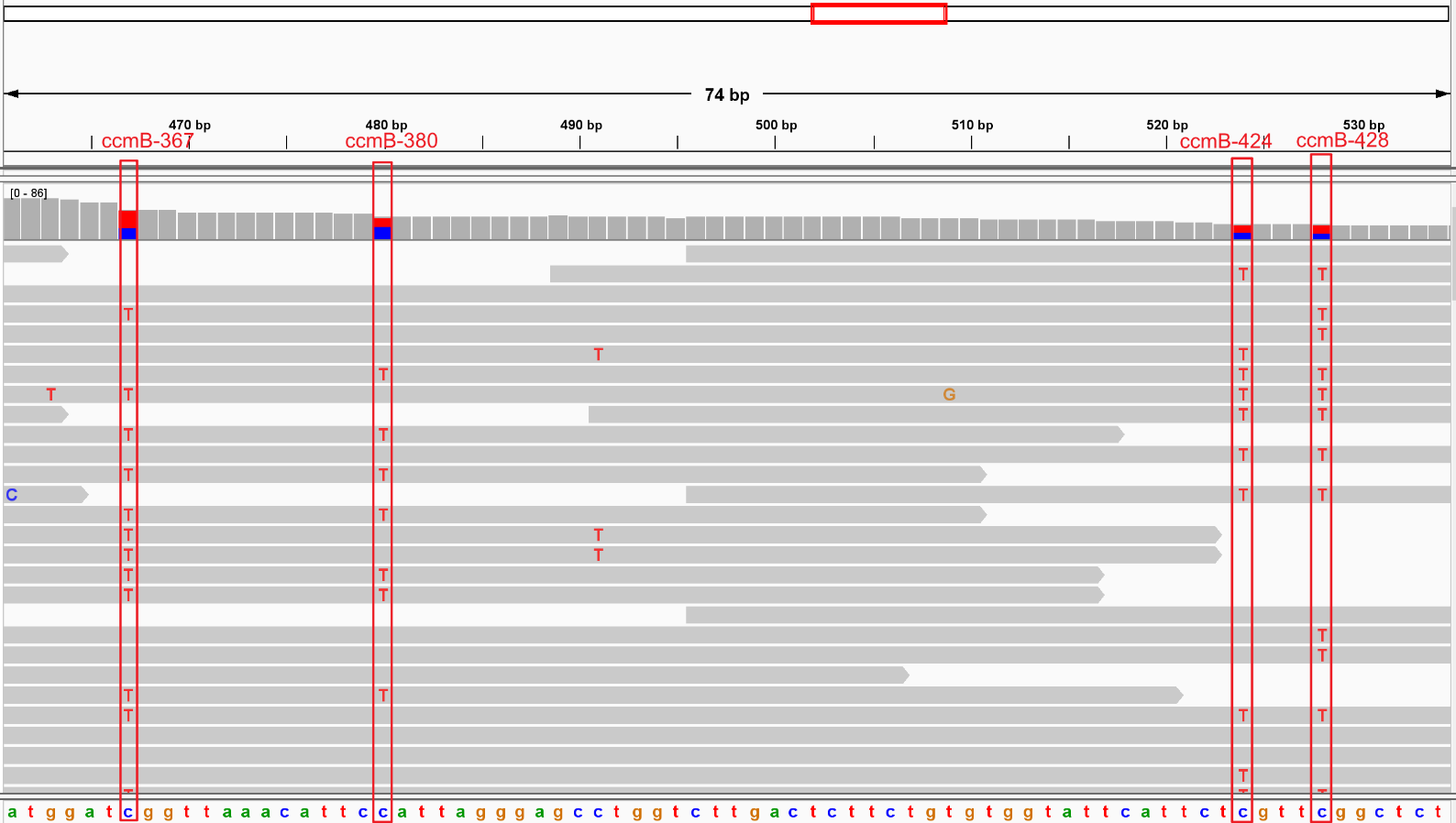

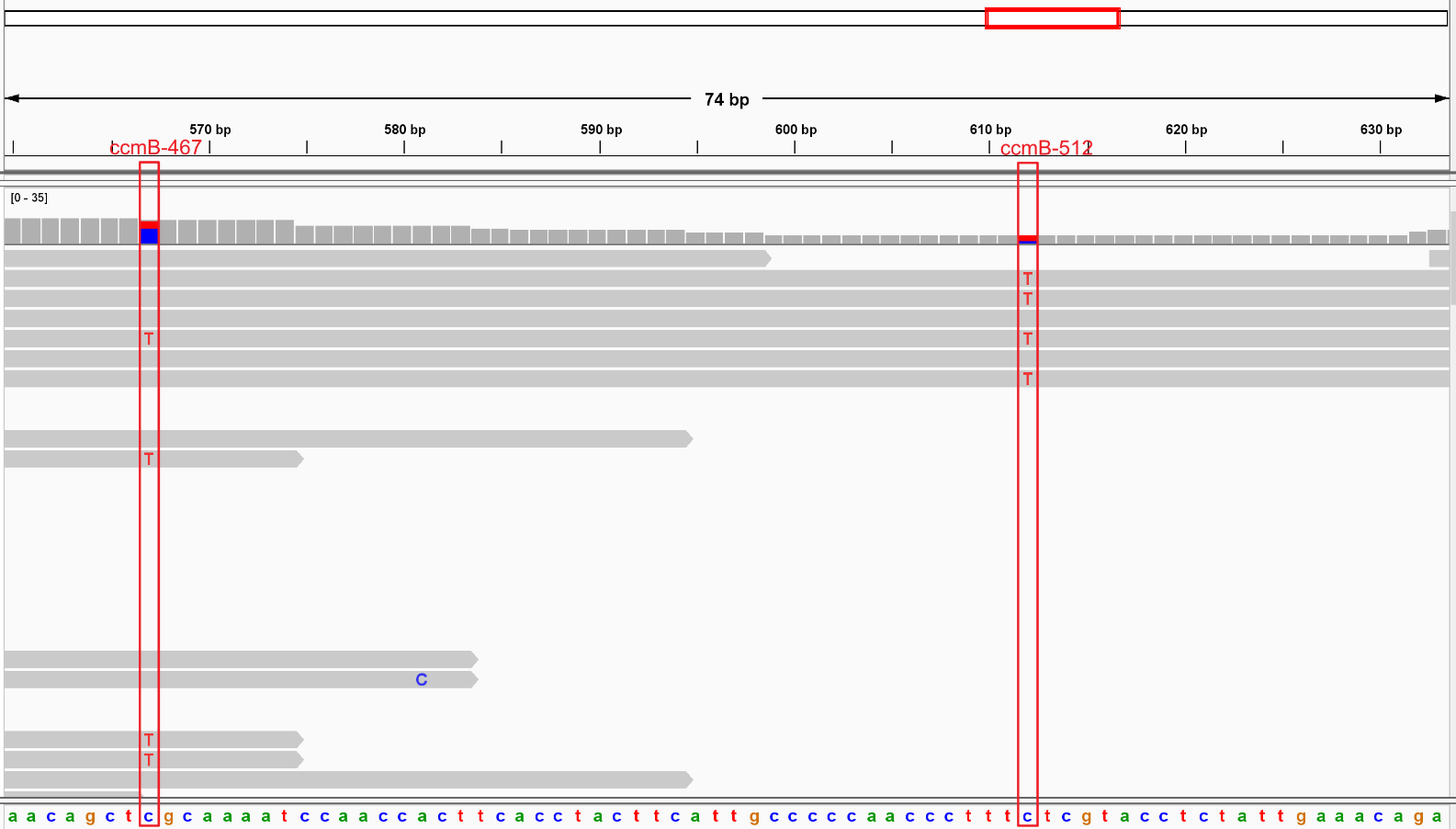

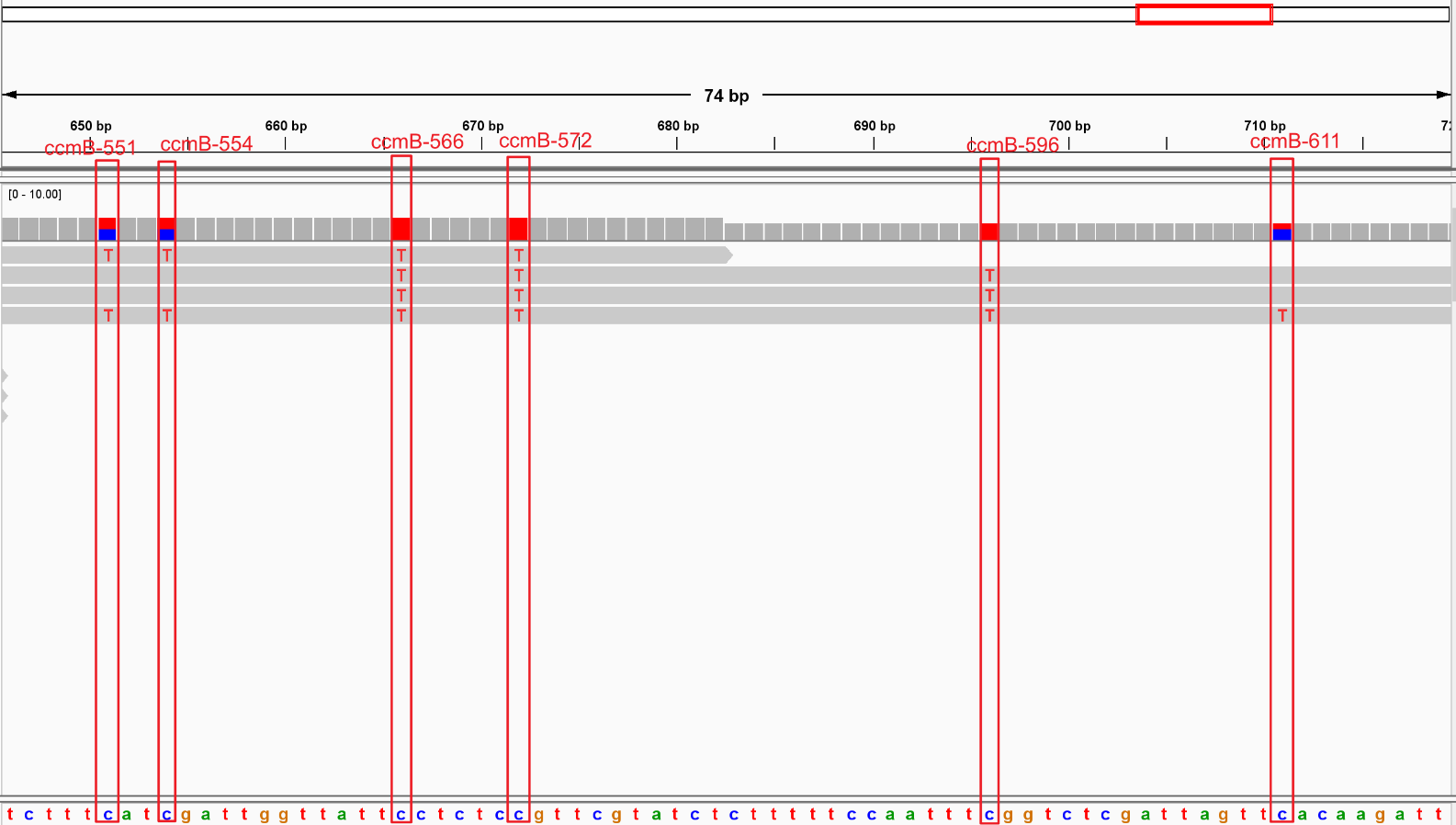


F alignment of RNA-seq reads to the coding sequence of *ccmC*. 23 RNA-seq editing sites: *ccmC*-38, 76, 103, 115, 133, 161, 179, 184, 281, 299, 331, 399, 400, 436, 446, 458, 497, 521, 575, 605, 608, 619, and 656 are highlighted in red squares.


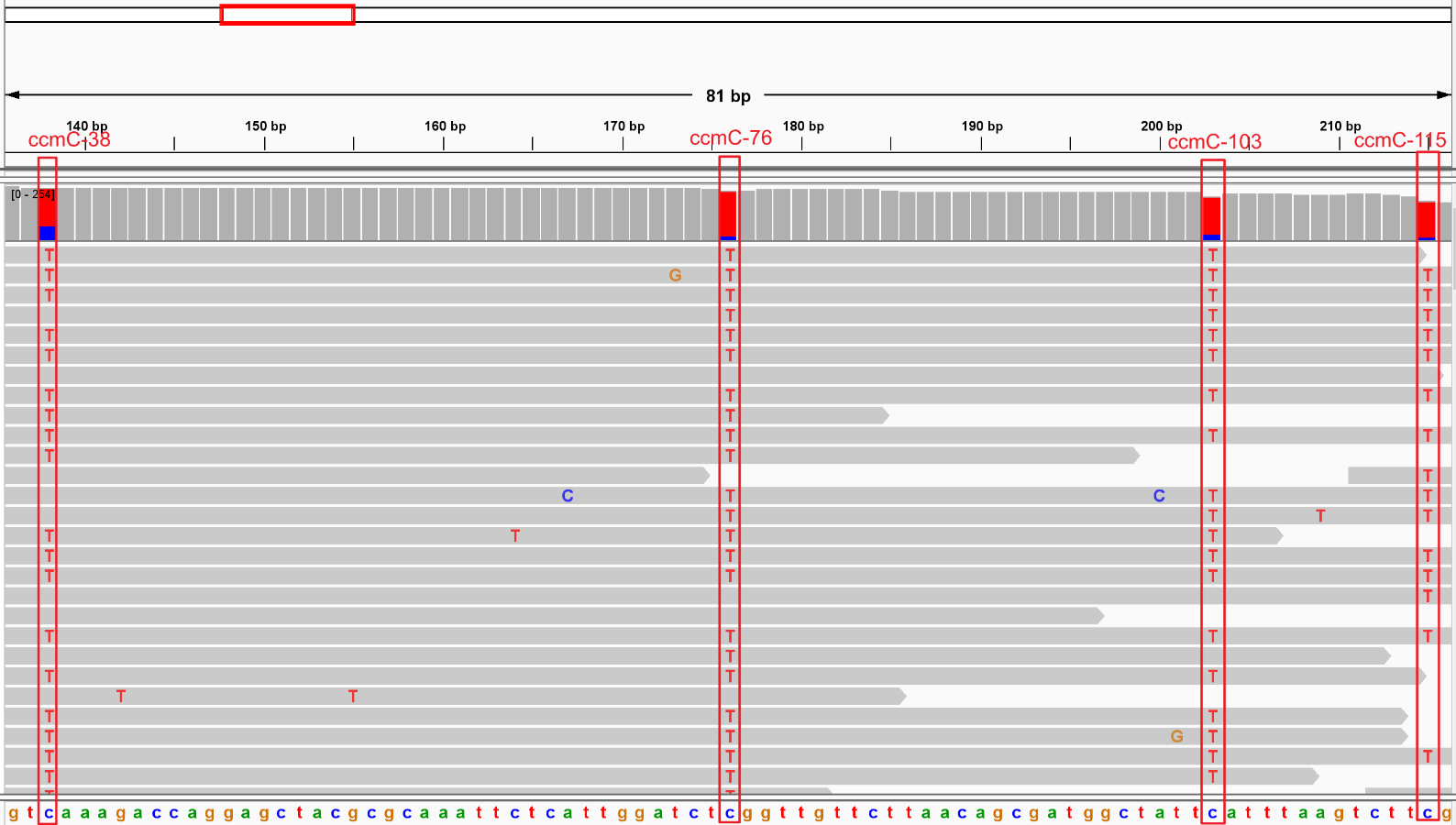

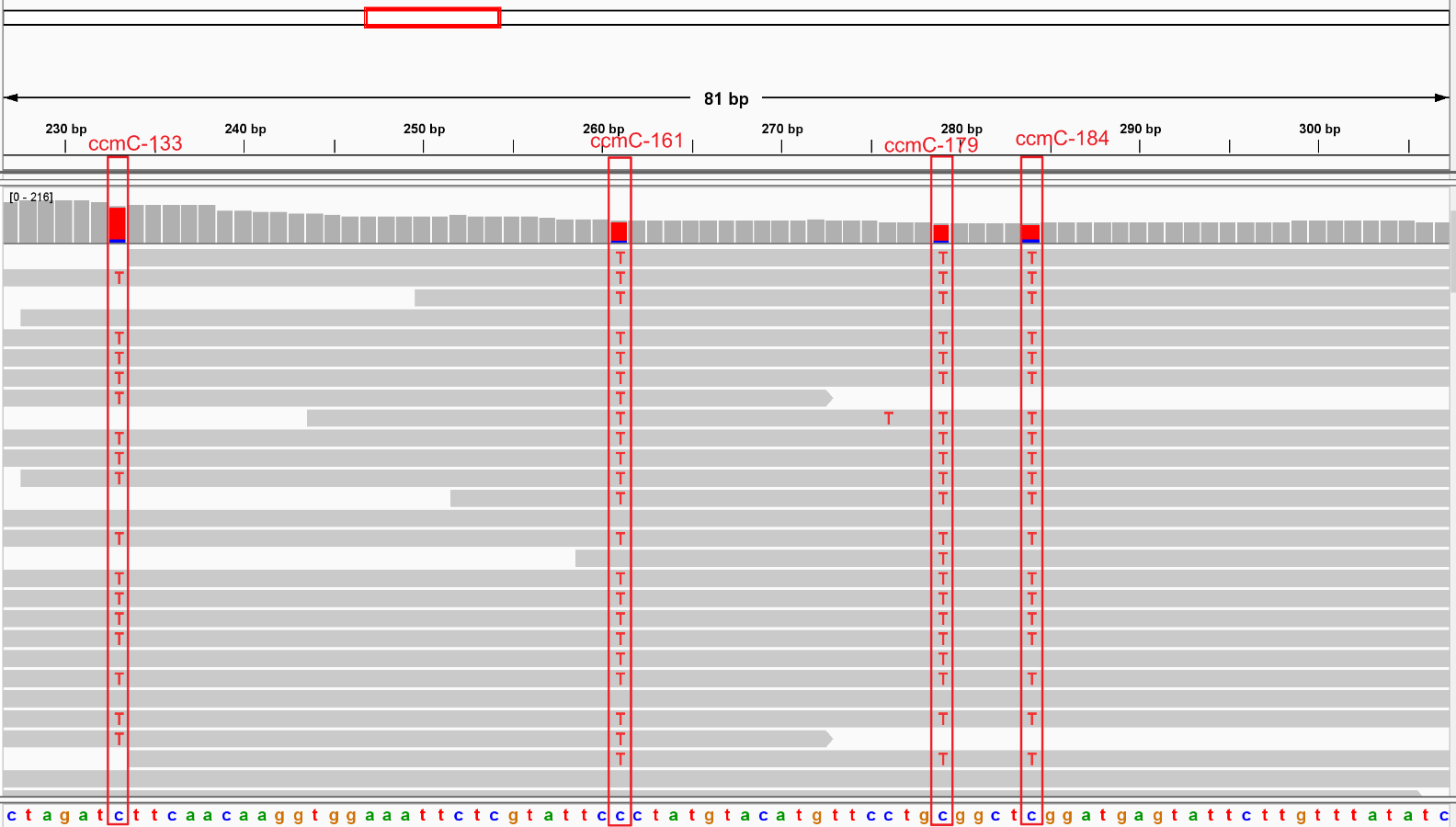

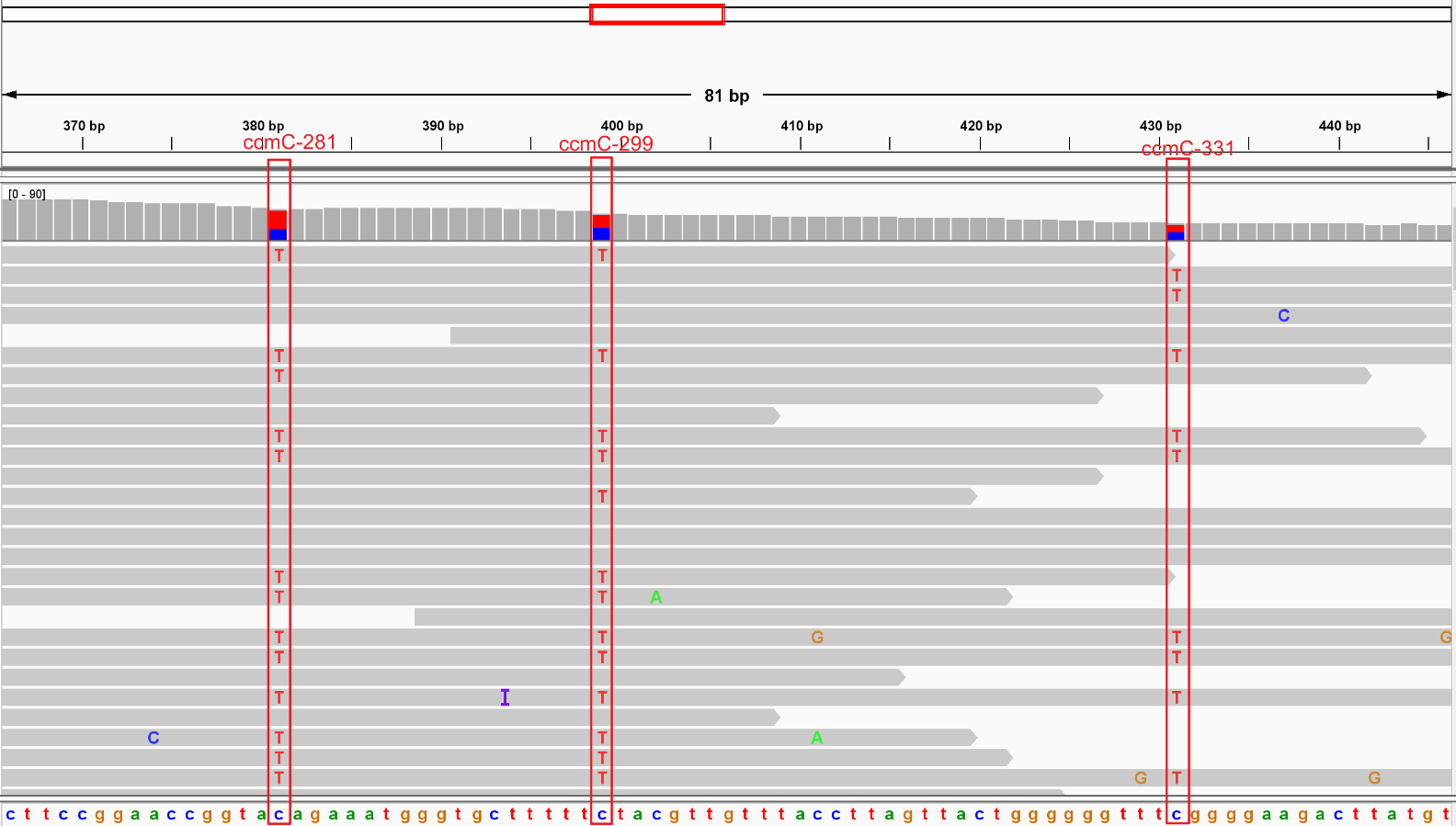

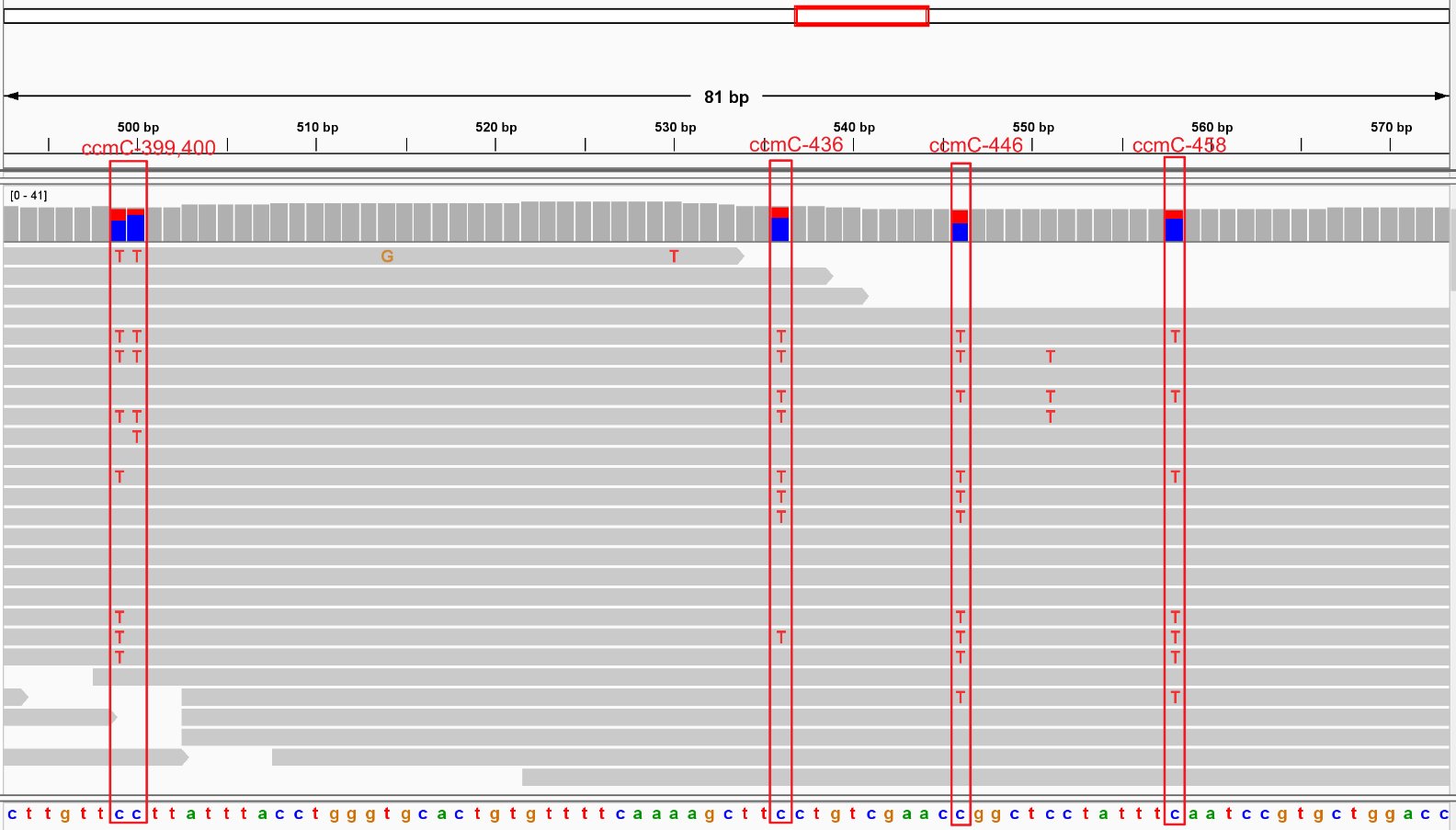

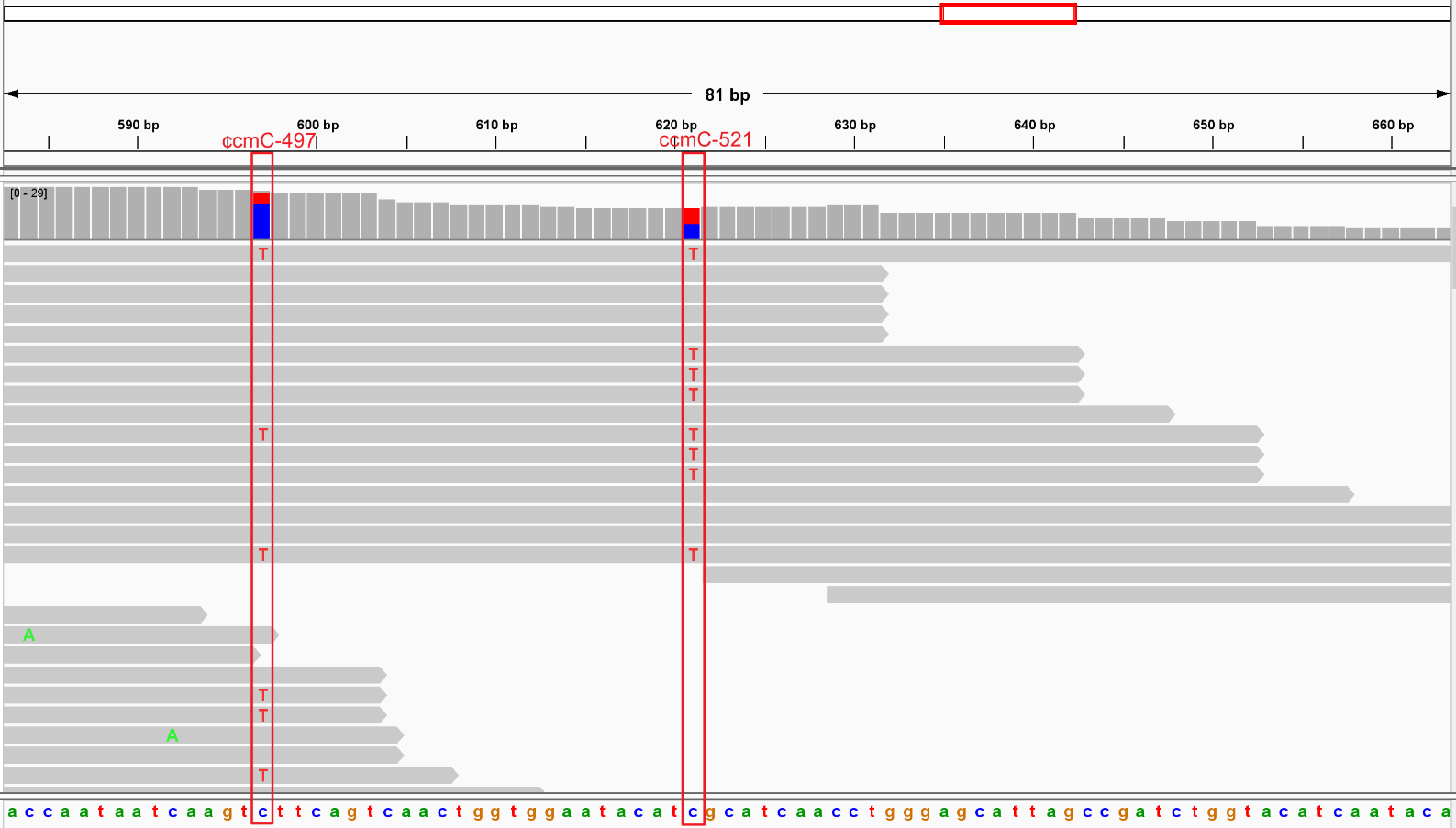

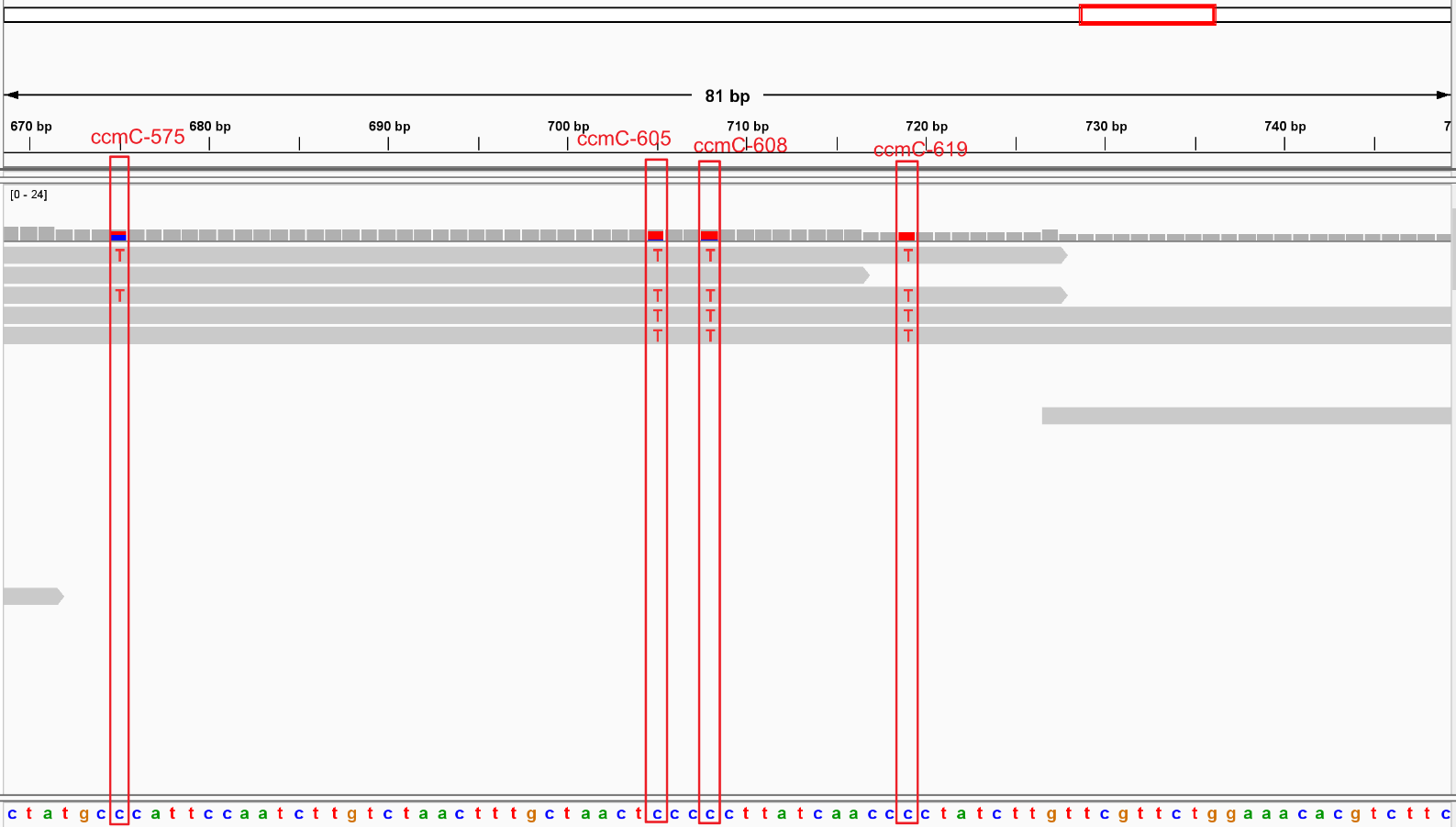

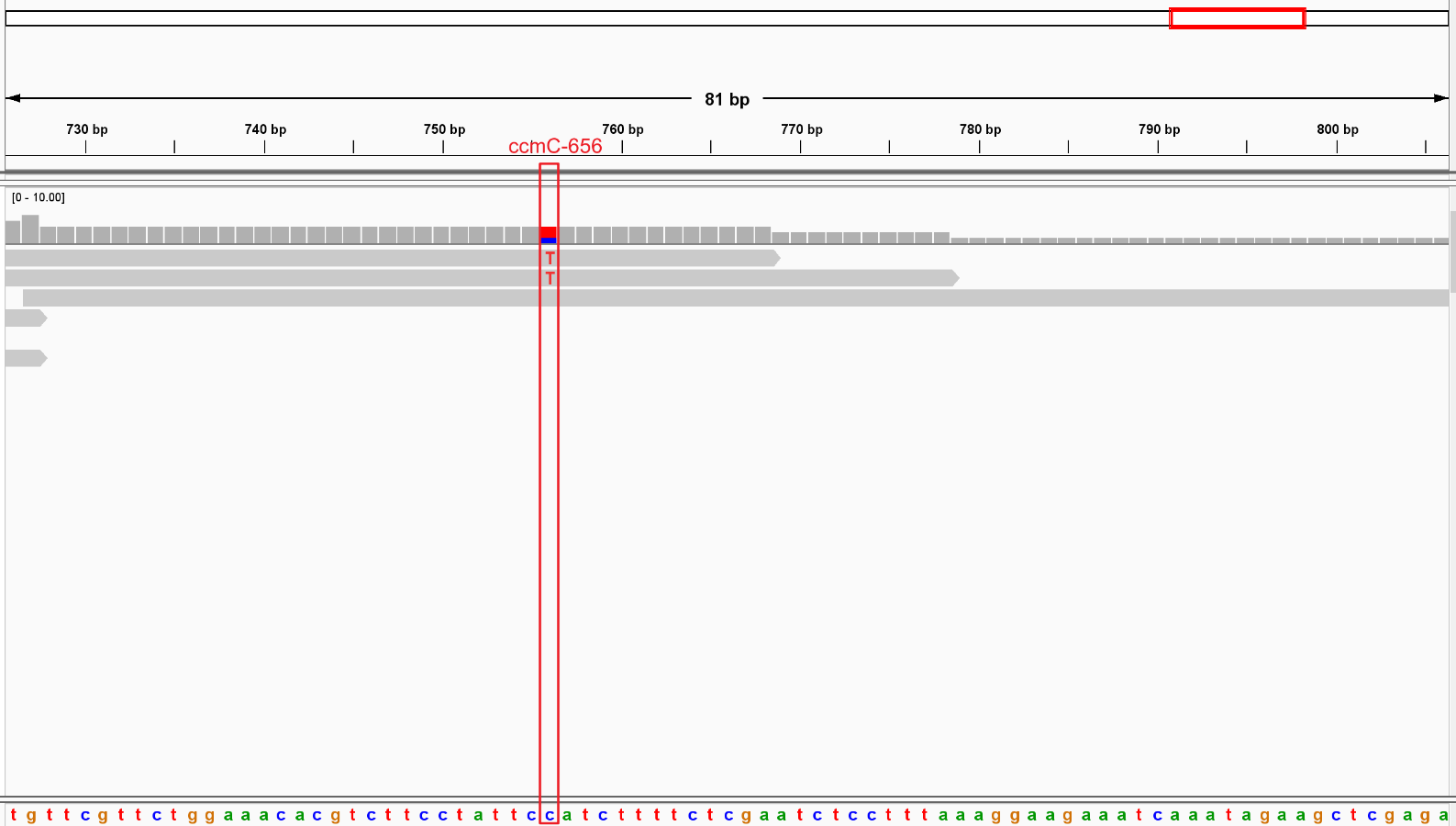


G alignment of RNA-seq reads to the coding sequence of *ccmFc*. 19 RNA-seq editing sites: ccmFc-38, 39, 50, 52, 103, 122, 146, 151, 155, 310, 334, 390, 406, 700, 701, 955, 1211, 1228, and 1233 are highlighted in red squares.


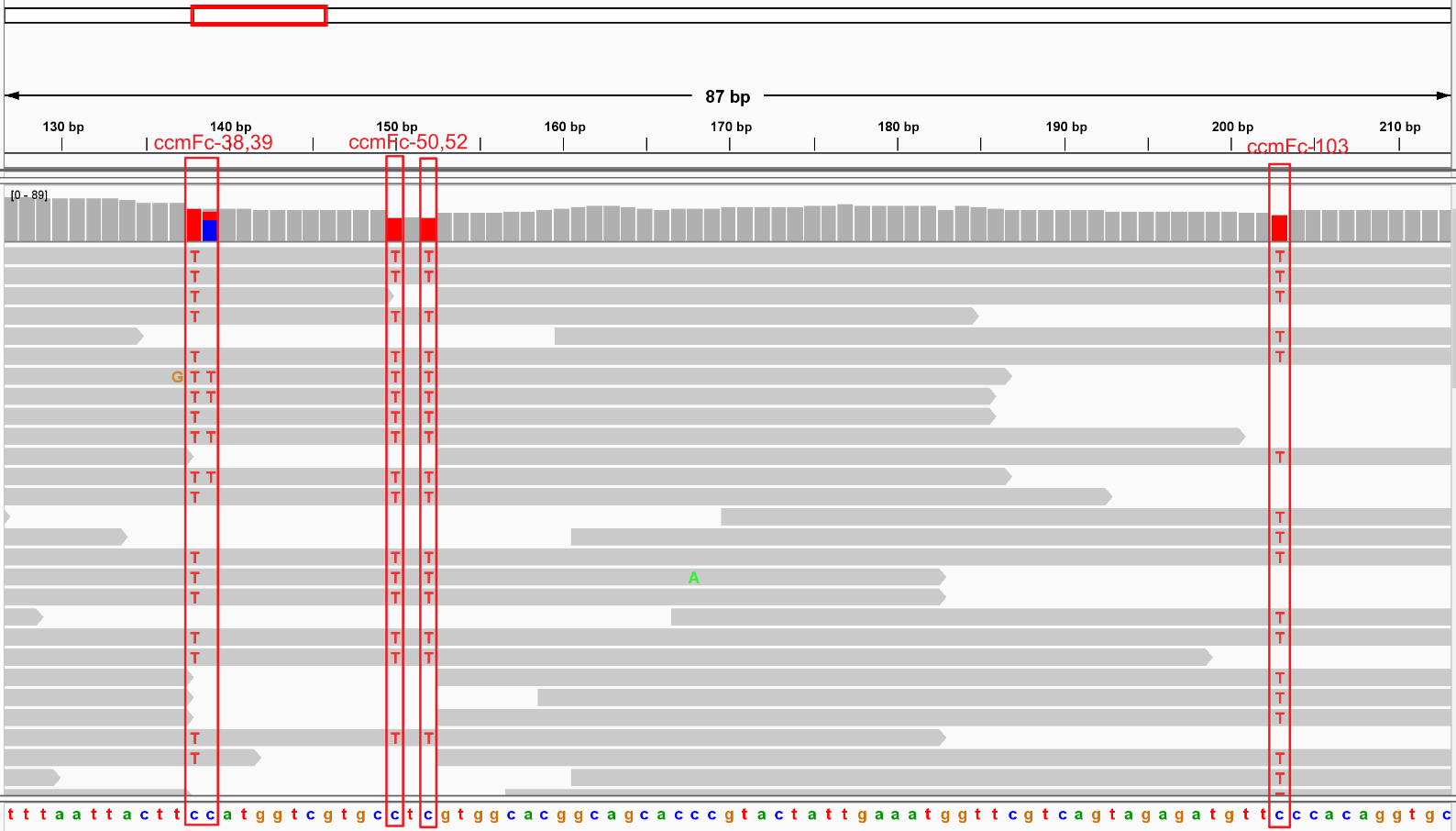


H alignment of RNA-seq reads to the coding sequence of *ccmFn*. 33 RNA-seq editing sites: ccmFn-38, 98, 137, 142, 151, 165, 248, 256, 283, 371, 378, 406, 713, 722, 732, 760, 782, 794, 809, 958, 1008, 1276, 1304, 1321, 1336, 1354, 1387, 1429, 1448, 1472, 1484, 1519, and 1747 are highlighted in red squares.

I alignment of RNA-seq reads to the coding sequence of *cob.* 11 RNA-seq editing sites: *cob*-298, 325, 358, 568, 853, 908, 982, 1015, 1084, 1101, and 1160 are highlighted in red squares.

J alignment of RNA-seq reads to the coding sequence of *cox1.* 16 RNA-seq editing sites: *cox1*-242, 254, 265, 452, 515, 551, 590, 715, 761, 1078, 1186, 1296, 1405, 1433, 1489, and 1499 are highlighted in red squares.

K alignment of RNA-seq reads to the coding sequence of *cox2.* 15 RNA-seq editing sites: *cox2*-32, 404, 422, 437, 505, 518, 580, 584, 586, 593, 594, 598, 615, 682, and 703 are highlighted in red squares.

L alignment of RNA-seq reads to the coding sequence of *cox3.* 13 RNA-seq editing sites: *cox3*-174, 245, 304, 311, 314, 419, 422, 566, 567, 651, 754, 764, and 885 are highlighted in red squares.

M alignment of RNA-seq reads to the coding sequence of *matR.* 19 RNA-seq editing sites: *matR*-32, 43, 166, 237, 326, 413, 474, 482, 1215, 1531, 1533, 1667, 1688, 1708, 1722, 1744, 1775, 1814, and 1832 are highlighted in red squares.

N alignment of RNA-seq reads to the coding sequence of *mttB.* 31 RNA-seq editing sites: *mttB*-16, 25, 26, 64, 100, 112, 128, 178, 188, 202, 236, 262, 328, 331, 344, 346, 373, 376, 379, 407, 472, 497, 505, 541, 554, 578, 610, 616, 667, 672, and 713 are highlighted in red squares.

O alignment of RNA-seq reads to the coding sequence of *nad1.* 23 RNA-seq editing sites: *nad1*-215, 265, 307, 308, 376, 401, 436, 490, 493, 500, 536, 635, 725, 734, 740, 743, 755, 779, 792, 823, 898, 909, 928 are highlighted in red squares.

P alignment of RNA-seq reads to the coding sequence of *nad2.* 30 RNA-seq editing sites: *nad2*-26, 89, 92, 109, 223, 252, 303, 308, 311, 356, 361, 367, 401, 428, 497, 741, 788, 800, 809, 928, 958, 1028, 1058, 1276, 1298, 1400, 1408, 1409, 1416, and 1457 are highlighted in red squares.

Q alignment of RNA-seq reads to the coding sequence of *nad3.* 16 RNA-seq editing sites: *nad3*-44, 62, 79, 80, 124, 146, 208, 209, 215, 230, 251, 266, 275, 317, 344, and 349 are highlighted in red squares.

R alignment of RNA-seq reads to the coding sequence of *nad4.* 32 RNA-seq editing sites: *nad4*-65, 68, 98, 149, 157, 188, 353, 359, 367, 407, 427, 440, 599, 650, 758, 847, 848, 878, 909, 997, 1001, 1007, 1100, 1120, 1123, 1142, 1163, 1298, 1346, 1364, 1408, and 1424 are highlighted in red squares.

S alignment of RNA-seq reads to the coding sequence of *nad4L.* 13 RNA-seq editing sites: *nad4L*-11, 17, 25, 56, 65, 70, 80, 101, 128, 149, 158, 167, and 251 are highlighted in red squares.

T alignment of RNA-seq reads to the coding sequence of *nad5.* 26 RNA-seq editing sites: *nad5*-155, 242, 359, 374, 398, 539, 548, 608, 609, 629, 676, 713, 725, 835, 1310, 1490, 1550, 1580, 1589, 1610, 1731, 1895, 1916, 1918, 1958, 1981 are highlighted in red squares.

U alignment of RNA-seq reads to the coding sequence of *nad6.* 11 RNA-seq editing sites: *nad6*-26, 88, 89, 95, 103, 161, 169, 191, 360, 463, 569 are highlighted in red squares.

V alignment of RNA-seq reads to the coding sequence of *nad7.* 20 RNA-seq editing sites: *nad7*-244, 251, 316, 335, 344, 383, 531, 578, 724, 739, 740, 769, 944, 963, 1050, 1057, 1103, 1124, 1137, and 1166 are highlighted in red squares.

W alignment of RNA-seq reads to the coding sequence of *nad9.* 9 RNA-seq editing sites: *nad9*-92, 167, 289, 298, 310, 328, 368, 398, and 439 are highlighted in red squares.

X alignment of RNA-seq reads to the coding sequence of *rpl5.* 7 RNA-seq editing sites: *rpl5*-35, 47, 160, 414, 441, 509, 512 are highlighted in red squares.

Y alignment of RNA-seq reads to the coding sequence of *rpl10.* Seven RNA-seq editing sites: *rpl10*-9, 101, 134, 174, 180, 330, 371 are highlighted in red squares.

Z alignment of RNA-seq reads to the coding sequence of *rpl16.* Two RNA-seq editing sites: *rpl16*-221 and 279 are highlighted in red squares.

a alignment of RNA-seq reads to the coding sequence of *rps3.* Eight RNA-seq editing sites: *rps3*-92, 512, 713, 1152, 1355, 1413, 1496, and 1582 are highlighted in red squares.

b alignment of RNA-seq reads to the coding sequence of *rps4.* 18 RNA-seq editing sites: *rps4*-176, 205, 219, 275, 287, 299, 316, 344, 449, 491, 504, 791, 935, 946, 956, 971, 1022, and 1036 were highlighted in red squares.

c alignment of RNA-seq reads to the coding sequence of *rps12.* Five RNA-seq editing sites: *rps12*-104, 159, 196, 221, 284 were highlighted in red squares.

d alignment of RNA-seq reads to the coding sequence of *rps13.* Three RNA-seq editing sites: *rps13*-56, 100, 287 were highlighted in red squares.

Fig. S6 Detection of polymorphic sites within the 12 mitochondrial marker for three individuals of *S. miltiorrhiza*, *S. officinalis*, and *S. splendens* using Sanger sequencing.

A Detection of 2 polymorphic sites within the cox1i12 marker for three individuals of *S. miltiorrhiza*, *S. officinalis*, and *S. splendens* using Sanger sequencing.

B Detection of 9 polymorphic sites within the cox2i12 marker for three individuals of *S. miltiorrhiza*, *S. officinalis*, and *S. splendens* using Sanger sequencing.

C Detection of 2 polymorphic sites within the nad1i23 marker for three individuals of *S. miltiorrhiza*, *S. officinalis*, and *S. splendens* using Sanger sequencing.

D Detection of 3 polymorphic sites within the nad2i12 marker for three individuals of *S. miltiorrhiza*, *S. officinalis*, and *S. splendens* using Sanger sequencing.

E Detection of 4 polymorphic sites within the nad2i34 marker for three individuals of *S. miltiorrhiza*, *S. officinalis*, and *S. splendens* using Sanger sequencing.

F Detection of 2 polymorphic sites within the nad2i45 marker for three individuals of *S. miltiorrhiza*, *S. officinalis*, and *S. splendens* using Sanger sequencing.

G Detection of 3 polymorphic sites within the nad4i12 marker for three individuals of *S. miltiorrhiza*, *S. officinalis*, and *S. splendens* using Sanger sequencing.

H Detection of 8 polymorphic sites within the nad4i34 marker for three individuals of *S. miltiorrhiza*, *S. officinalis*, and *S. splendens* using Sanger sequencing.

I Detection of 3 polymorphic sites within the nad5i45 marker for three individuals of *S. miltiorrhiza*, *S. officinalis*, and *S. splendens* using Sanger sequencing.

J Detection of 3 polymorphic sites within the nad7i12 marker for three individuals of *S. miltiorrhiza*, *S. officinalis*, and *S. splendens* using Sanger sequencing.

K Detection of 6 polymorphic sites within the nad7i23 marker for three individuals of *S. miltiorrhiza*, *S. officinalis*, and *S. splendens* using Sanger sequencing.

L Detection of 3 polymorphic sites within the nad7i34 marker for three individuals of *S. miltiorrhiza*, *S. officinalis*, and *S. splendens* using Sanger sequencing.
