# Supplementary figures and images for: Unveiling the mitochondrial genome of Salvia splendens insights into the evolutionary traits within the genus Salvia
Source: Sci Rep. 2025 Apr 17;15:13344. doi: 10.1038/s41598-025-96637-9 (PMC12006378; doi:10.1038/s41598-025-96637-9)

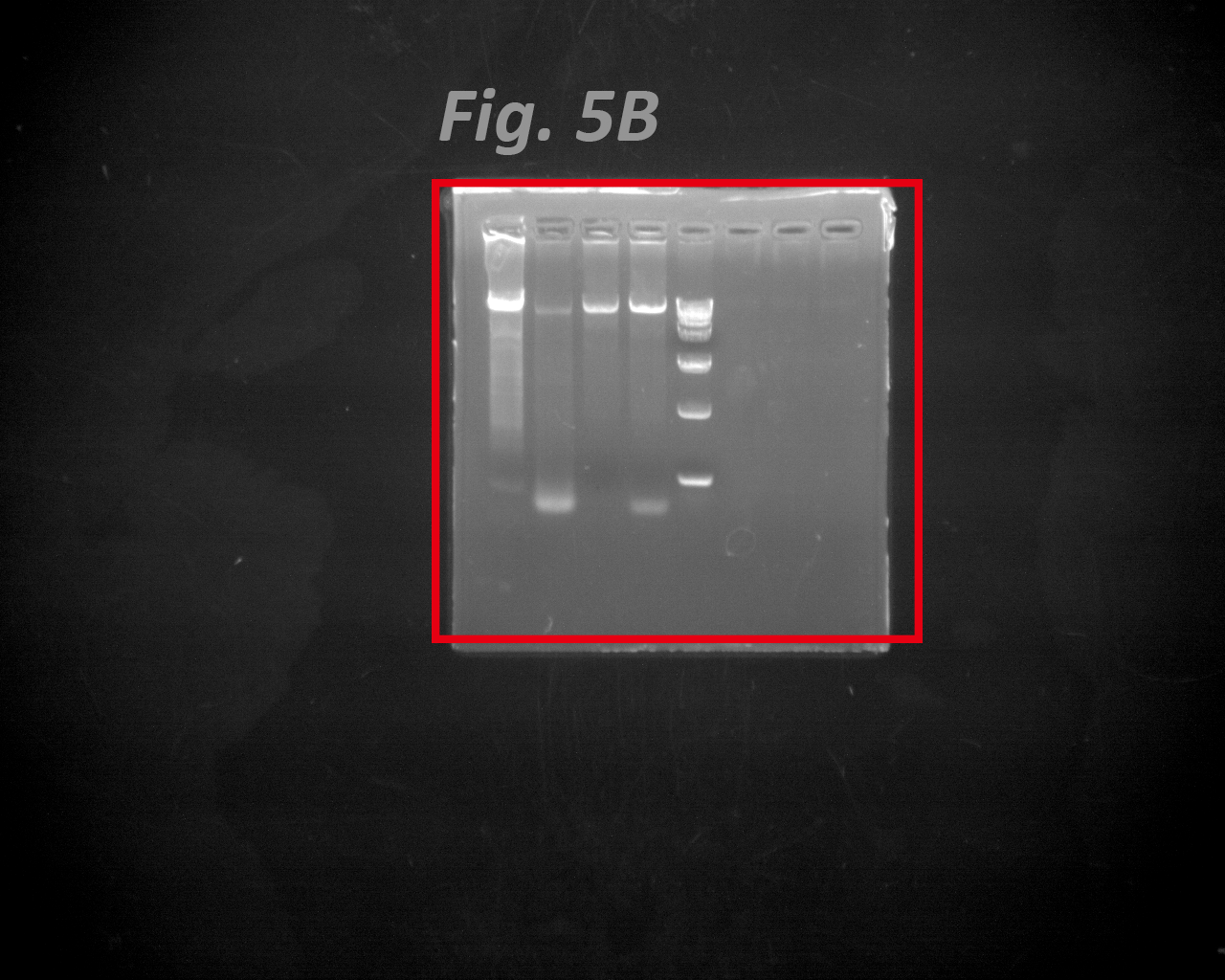

Supplement: Supplementary file 3 — Supplementary Information 3. [file 41598_2025_96637_MOESM3_ESM.tif]
